# The Catalytic Asymmetric Intermolecular Prins Reaction

**DOI:** 10.1021/jacs.1c10245

**Published:** 2021-11-30

**Authors:** C. David Díaz-Oviedo, Rajat Maji, Benjamin List

**Affiliations:** † Max-Planck-Institut für Kohlenforschung, Kaiser-Wilhelm-Platz 1, 45470 Mülheim an der Ruhr, Germany

## Abstract

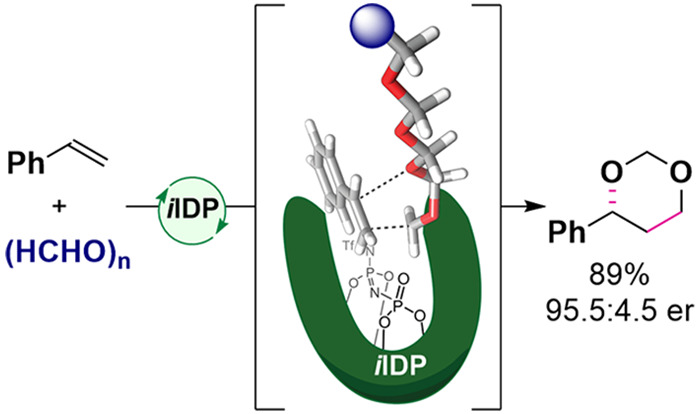

Despite their significant potential, catalytic asymmetric reactions of olefins with formaldehyde are rare and metal-free approaches have not been previously disclosed. Here we describe an enantioselective intermolecular Prins reaction of styrenes and paraformaldehyde to form 1,3-dioxanes, using confined imino-imidodiphosphate (*i*IDP) Brønsted acid catalysts. Isotope labeling experiments and computations suggest a concerted, highly asynchronous addition of an acid-activated formaldehyde oligomer to the olefin. The enantioenriched 1,3-dioxanes can be transformed into the corresponding optically active 1,3-diols, which are valuable synthetic building blocks.

The reactions of olefins and aldehydes have earned a coveted spot in the repertoire of the chemist due to the widespread occurrence of these two functional groups. The first report on this type of transformation by Kriewitz in 1899 describes the formation of unsaturated alcohols upon heating pinene with paraformaldehyde.^1^ However, the first comprehensive study on the acid-catalyzed reaction of olefins and aldehydes dates back to more than 100 years ago when Prins published a series of reports on the sulfuric acid-catalyzed reaction of several olefins (styrene, pinene, camphene, and anethole) with formaldehyde.^2^ During the subsequent decades, chemists have not only frequently used the Prins reaction but also aimed at unveiling its mechanism.^3^ The key step is considered to be the nucleophilic attack of the olefin to the activated carbonyl group (“carbonylonium ion”),^4^ producing a γ-hydroxycarbenium ion.^5^ The fate of this reactive species depends on the reaction conditions, leading to products such as unsaturated alcohols, 1,3-diols, and/or derivatives thereof (Figure 1A). Because it embodies a double bond functionalization *and* a carbon–carbon bond formation in a single step,^6^ the Prins reaction remains a key transformation in synthesis, providing direct access to products with common motifs in fragrances and bioactive molecules.^7^ Nevertheless, the possibility of side reactions (carbonyl-ene,^8^ carbonyl-olefin metathesis,^9^ and/or olefin polymerization, among others) can complicate the panorama. For these reasons, designing an efficient, catalytic variant of this reaction, surmounting the challenging control of product selectivity, is extremely desirable. Hitherto developed methodologies toward catalytic intermolecular Prins reactions entail the use of Brønsted acids, Lewis acids, iodine, ionic liquids, heteropolyacids, and heterogeneous catalysts (zeolites or solid-supported acids).^10−16^ However, the use of corrosive or toxic reagents represents a drawback of many of these procedures. It is also surprising that, despite the broad synthetic potential of the Prins reaction, a catalytic asymmetric intermolecular version remains unknown. For this reason, and encouraged by our previous studies on catalytic asymmetric Prins cyclizations,^17^ we became intrigued by the possibility to design an enantioselective intermolecular version of this type of olefin-aldehyde reaction from readily available substrates (Figure 1B). Here we report a highly enantioselective intermolecular Prins reaction of styrenes and paraformaldehyde to form 1,3-dioxanes, using confined imino-imidodiphosphate (*i*IDP) Brønsted acid catalysts.

**Figure 1:**
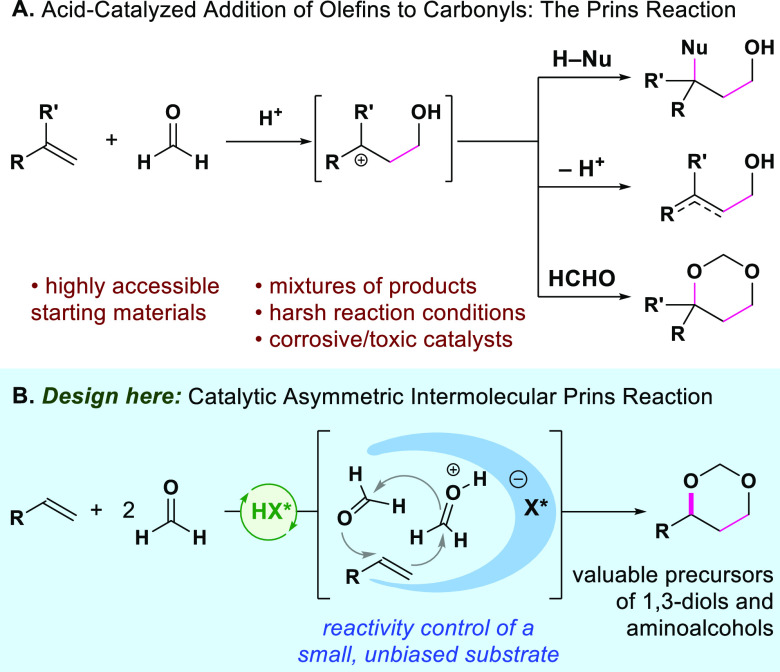
(A) The Prins reaction. (B) Our approach: chiral, confined Brønsted acid-catalyzed asymmetric intermolecular Prins reaction of styrenes and paraformaldehyde.

We began our investigation by exploring the reaction of styrene (**1a**) and paraformaldehyde (**2a**). Several established chiral Brønsted acid catalysts (phosphoric acids, disulfonimides, imidodiphosphates)^18−20^ did not lead to any conversion (see Supporting Information). The confined imino-imidodiphosphate (*i*IDP) **4a**, which performed superbly in the Prins cyclization,^17b^ afforded the corresponding 1,3-dioxane **3a** only in trace amounts, but with a promising er of 91:9 (Table 1, entry 1). This motivated us to test the effect of substituents on the catalyst skeleton. Fortunately, while the synthesis of *i*IDP catalysts previously involved multiple steps, a one-pot process, recently developed by our group,^21^ enabled the preparation and testing of several *i*IDPs. We found that the presence of electron-withdrawing groups (EWGs) at the 3,3′-aryl substituents of the BINOL backbone translates into a vast increase in reactivity. For example, 3,3′-(4-CF_3_-phenyl)-substituted catalyst **4b** provided product **3a** with good enantioselectivity (er = 91.5:8.5) and good yield (entry 2).

**Table 1:**
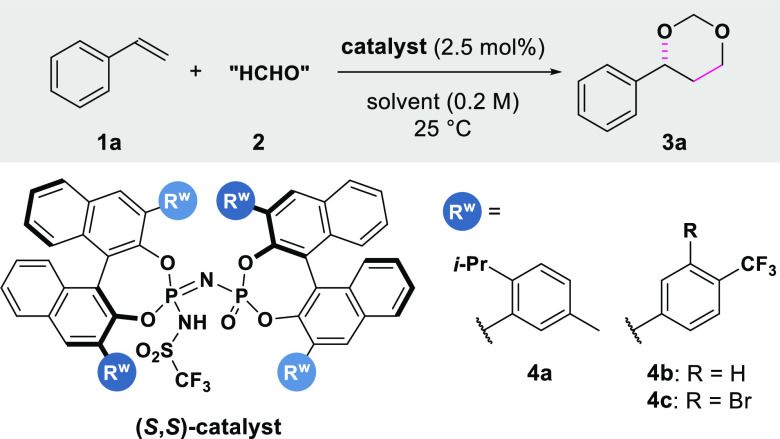
Reaction Development^a^

Entry	**catalyst** (mol %)	solvent	**HCHO** source	time (h)	**3a**, yield (%)^b^	er **3a**^c^
1	**4a** (5)	CHCl_3_	PF	24	<5	91:9
2	**4b** (2.5)	CHCl_3_	PF	24	74	91.5:8.5
3	**4b** (2.5)	CyH	PF	48	65	94.5:5.5
4	**4b** (2.5)	CyH	formalin^d^	48	19	93:7
5	**4b** (2.5)	CyH	trioxane^e^	48	11	92.5:7.5
6	**4c** (2.5)	CyH	PF	48	93	94:6
7^f^	**4c** (2.5)	**CyH**	**PF**	**72**	91 (90)^g^	95.5:4.5

a **1a** (25 μmol), HCHO **2** (2–3 equiv) and 2.5 mol % of catalyst **4**, in 125 μL of solvent (0.2 M), unless otherwise indicated.

b Determined by ^1^H NMR analysis (internal standard: Ph_3_CH).

c Determined by HPLC analysis using a chiral stationary phase.

d Formalin: HCHO 37 wt % (aq).

e 1,3,5-Trioxane.

f 0.1 M.

g Isolated yield. PF: paraformaldehyde. See the Supporting Information for further details.

Using cyclohexane as solvent (entry 3) led to product **3a** with an encouraging 94.5:5.5 er, but with a lower yield. Other formaldehyde sources, such as formalin or 1,3,5-trioxane, proved less reactive, although the enantioselectivity remained practically unchanged (entries 4–5). Introducing a bromine atom on the 3,3′-aryl ring (**4c**) led to enhanced reactivity (entry 6), and adjusting the concentration (0.1 M) allowed us to obtain **3a** with 90% isolated yield and 95.5:4.5 er (entry 7).

With these optimized conditions, we explored the reaction of various commercially available or easily accessible styrenes with paraformaldehyde (Table 2). Chloromethyl-substituted olefin **1b** provided the corresponding 1,3-dioxane in moderate yield and good enantioselectivity. Alkyl-substituted styrene **1c** displayed excellent reactivity, although with a slight decrease in enantioselectivity. The presence of electron-donating groups was tolerated, as was the case for pivalate **1d**. Similarly, thiopivalate **1e** proved also to be a suitable substrate for our methodology. Electron-deficient styrenes afforded the corresponding products with excellent enantioselectivity, as observed for the halogen-substituted substrates (**1f**–**1i**). However, to overcome the reactivity challenge posed by even more electron-deficient substrates, a more acidic *i*IDP catalyst with EWGs on the binaphthyl backbone was designed, considering the previous success of this strategy.^22^ Gratifyingly, 6,6′-(*i*-C_3_F_7_)_2_-substituted BINOL-derived *i*IDP **4b′** allowed the transformation of *m*-bromo-substituted styrene **1j** to the corresponding 1,3-dioxane with moderate yield and good enantioselectivity. Furthermore, other substitution patterns of the aromatic ring (**1k**–**1n**) were tolerated, allowing access to the desired 1,3-dioxanes with good enantioselectivity. 3,4-Dioxygenated substrate **1o** could also be successfully transformed, affording the corresponding 1,3-dioxane with good enantioselectivity.

**Table 2:**
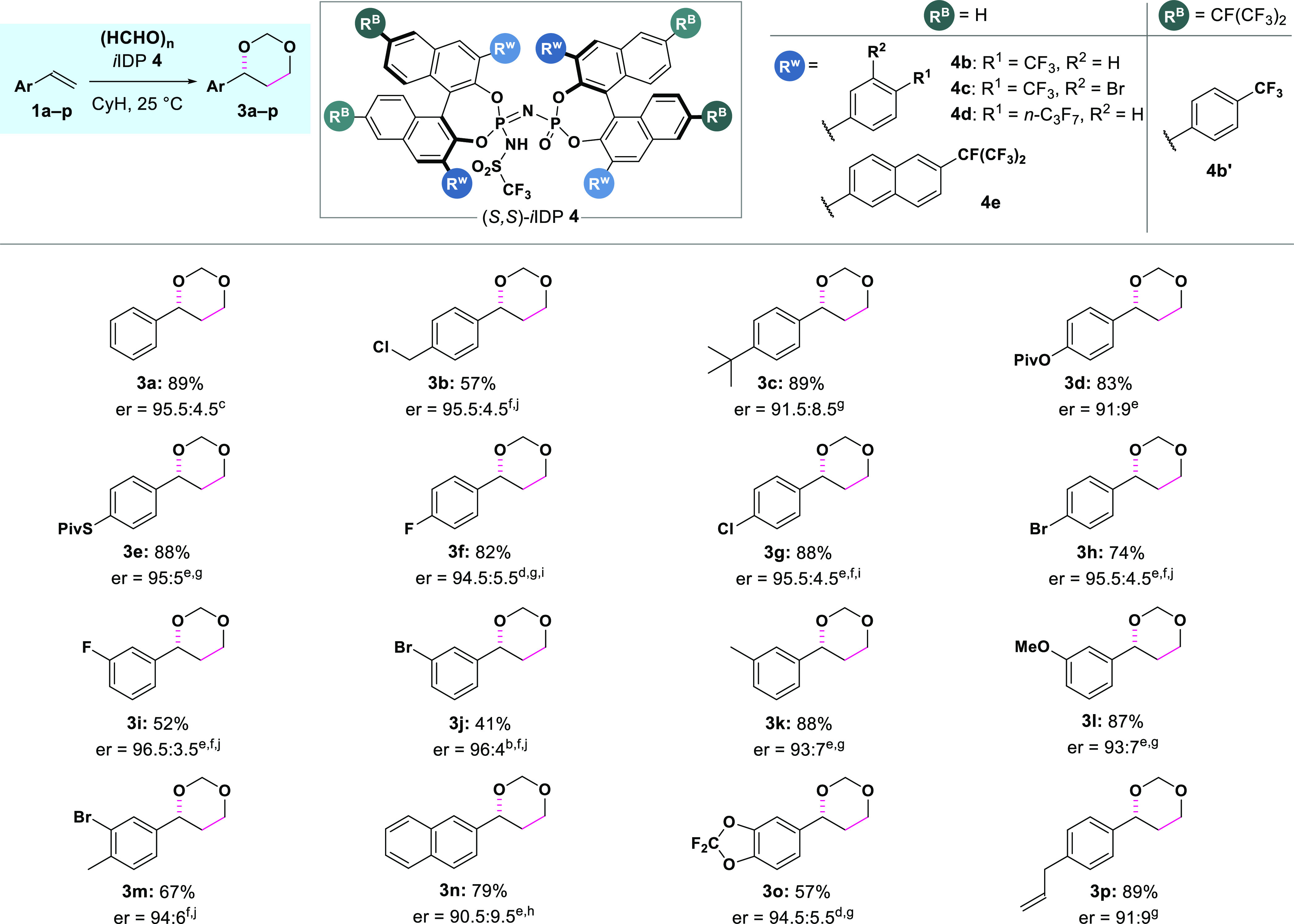
Substrate Scope^a^

a 0.25 mmol of substrate **1**, 2–3 equiv of paraformaldehyde **2a**, and 2.5 mol % of catalyst **4b**, in 2.5 mL of dry cyclohexane (0.1 M) at 25 °C for 72 h, unless otherwise stated.

b Using catalyst **4b′**.

c Using catalyst **4c**.

d Using catalyst **4d**.

e Using catalyst **4e**.

f Concentration: 0.2 M.

g Concentration: 0.05 M.

h Concentration: 0.025 M.

i Reaction for 96 h.

j Reaction for 5 days. See the Supporting Information for further details.

Using α-methylstyrene as substrate led to full conversion and complex mixtures. Internal styrenes, such as *trans*-β-methylstyrene or *trans*-anethole, also proved challenging, since they were less reactive and led to decreased enantioselectivity. These cases require further catalyst optimization, which is currently ongoing in our laboratory. Finally, our developed *i*IDP-catalyzed Prins reaction proved to be selective for aryl olefins, as observed in the reaction of alkyl olefin-substituted styrene **1p**. Alkyl-substituted alkenes (e.g., 1-octene or homoallylbenzene) were unreactive under the optimized reaction conditions (see Supporting Information for more details).

The presence of an acetal moiety in the prepared 1,3-dioxanes represents a potential use of our developed catalytic asymmetric Prins reaction as the key part of a direct synthesis of optically active 1,3-diols starting from styrenes. Gratifyingly, using the conditions reported by Fujioka for the ring opening of unsubstituted acetals (formals),^23^ the enantioenriched 1,3-dioxane **3a** (er = 95:5) could be readily transformed to the corresponding 1,3-diol **5** without erosion of enantiopurity (Figure 2A). Compound **5**, a common intermediate in the chemical syntheses of fluoxetine,^24^ atomoxetine,^24^ and dapoxetine,^25^ can now be prepared asymmetrically from styrene **1a** with our Prins reaction/ring-opening sequence (80% yield over two steps, er = 95:5) (Figure 2A). This discloses a potential application of our methodology for the preparation of pharmaceutically relevant compounds.

The *i*IDP-catalyzed Prins reaction could also be applied to the synthesis of optically active 1,3-dioxanes with different degrees of deuteration, starting from styrene β,β-*d*_2_ (**1a′**) and/or paraformaldehyde-*d*_2_ (**2a′**) (Figure 2B). This approach can be potentially utilized in asymmetric syntheses of deuterated analogs of APIs, which are interesting molecules for medicinal chemists.^26^

**Figure 2:**
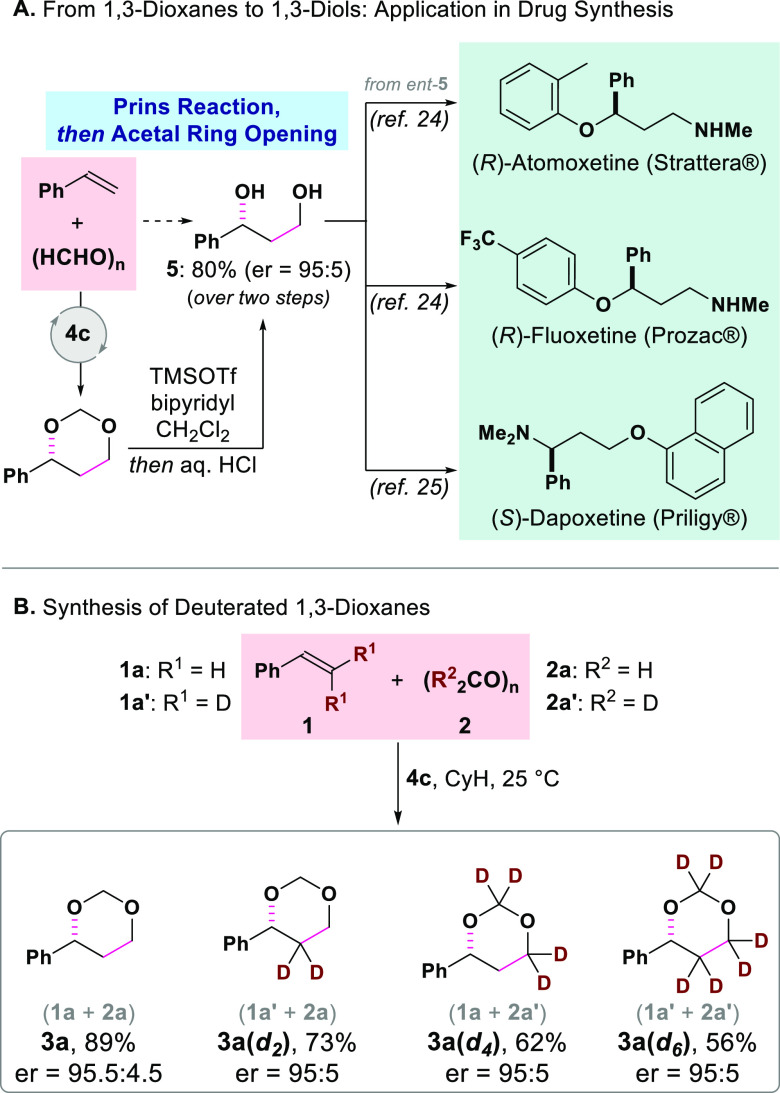
(A) From 1,3-dioxanes to 1,3-diols and potential application in drug synthesis. (B) Synthesis of deuterated 1,3-dioxanes.

We were intrigued if our catalytic, enantioselective methodology follows the stepwise pathway via a γ-hydroxy-carbocation, proposed in most of the reported mechanistic studies of the Prins reaction.^3^ This motivated us to gain a better understanding of the operating reaction pathway. To determine how the two formaldehyde units react with the olefin, we studied the reaction of styrene **1a** with mixtures of nondeuterated and deuterated paraformaldehyde ((HCHO)_*n*_**2a** and (DCDO)_*n*_**2a′**), using catalyst **4b**. We considered that, if a stepwise mechanism is proceeding, the two formaldehyde units should be attached to **1a** in different steps. This would translate into a reaction product with different ^1^H contents in the positions C-2 and C-6, with their ratio being quantifiable by ^1^H NMR (Figure 3A). Surprisingly, regardless of the **2a**/**2a′** ratio, the *i*IDP-catalyzed reaction provided in all cases **3a** with the same ^1^H content on C-2 and C-6 (^1^H content ratio C-2/C-6 close to 1). Contrastingly, the *p*-TsOH-catalyzed reaction formed a product with a ^1^H content ratio always different from 1 (Figure 3B). These results illustrated a striking difference in the reaction mechanism for the two catalysts.

**Figure 3:**
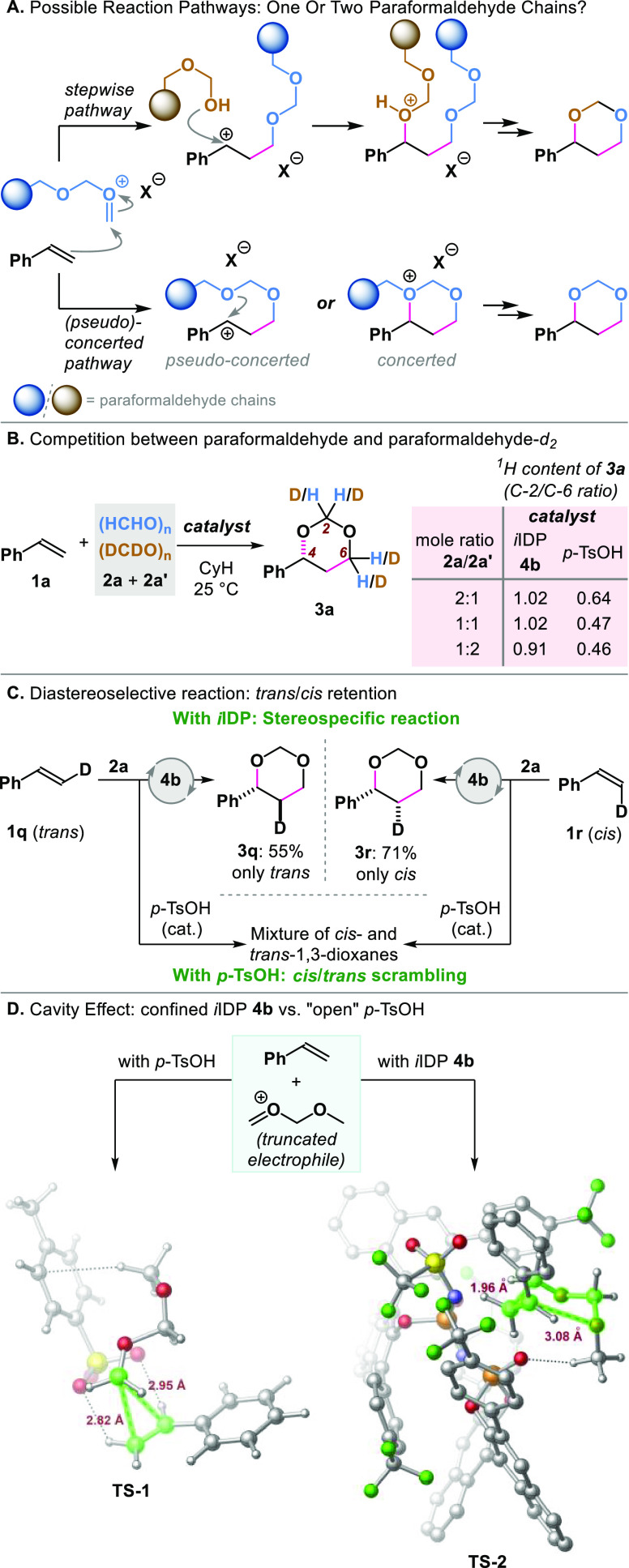
Mechanistic studies: (A) hypothesis on the possible reaction pathways, (B) competition experiment with deuterium-labeled paraformaldehyde, (C) stereospecificity experiment with styrene-β-*d*, (D) DFT-calculated transition states for the reaction of styrene and a “truncated electrophile” (for clarity, in **TS-2** hydrogen atoms bonded to aromatic rings were omitted; see the Supporting Information for further details).

To better understand this disparity, we subsequently studied the reaction of styrene-β-*d* (*trans*-: **1q**, and *cis*-: **1r**) with formaldehyde. With *i*IDP **4b** as catalyst, the *trans*-olefin produced exclusively the corresponding *trans*-product **3q**. The isomeric *cis*-olefin afforded only the *cis*-1,3-dioxane **3r** (Figure 3C) Contrarily, the *p*-TsOH-catalyzed reaction of β-deutero-styrenes (**1q**/**1r**) led in both cases to mixtures of *cis*/*trans*-1,3-dioxanes (Figure 3C), similar to the reported result for the H_2_SO_4_-catalyzed Prins reaction of **1q**.^3c^ These results suggest that the *i*IDP-catalyzed reaction proceeds via a (pseudo)-concerted pathway, probably due to the confined nature of the catalyst. In contrast, the *p*-TsOH-catalyzed reaction seems to proceed by a stepwise pathway involving a benzyl cation intermediate, which explains the observed *cis*/*trans* scrambling.

To rationalize these mechanistic dissimilarities, we resorted to DFT computations, considering a formaldehyde dimer-derived aldehydium ion as model reactive species (“truncated electrophile”), with a methoxy capping-group to resemble the polymeric chain of paraformaldehyde (Figure 3D). The optimized (PBE-D3/def2-SVP) transition state structure in the presence of the *p*-toluenesulfonate anion shows the electrophilic carbon approaching both carbon atoms of the olefin moiety, resembling a nonclassical “onium” ion (**TS-1**, Figure 3D). Furthermore, consistent with the report by Kupova,^3d,27^ the bond distance between the benzylic carbon and the remote oxygen was found to be 4.95 Å, ruling out any possibility of concerted cyclization. This arrangement suggests a stepwise operating pathway, where the benzylic carbocation intermediate rotates freely, leading to the observed *cis*/*trans* scrambling (Figure 3C). In contrast, within the confined *i*IDP anion cavity (catalyst **4b**), the corresponding transition state (**TS-2**, Figure 3D) adopts a chairlike geometry, where the C–C bond-formation event happens before the C–O bond formation. Notably, the benzylic carbon is significantly closer to the second oxygen atom of the formaldehyde dimer (distance C···O: 3.08 Å), which adopts an s-*cis* conformation due to a stabilizing CH···O interaction with the catalyst cavity. Hence, the transformation in this case follows a concerted pathway, explaining the observed stereospecificity with the deuterium-labeled styrenes (Figure 3C).

Next, we set out to understand the reason behind the observed stereoselectivity. The predicted enantioselectivity for the Prins reaction of **1a** with catalyst **4b** (e.r. 99:1 at the M06-2X/def2-TZVP+ CPCM(cyclohexane)//PBE-D3/def2-SVP level of theory) is in good agreement with the experimentally observed value (e.r. 94.5:5.5). To identify the source of stereoinduction, we conducted a distortion-interaction (DI) analysis.^28^ The main source of the calculated energy difference (ΔΔ*E*_gas_^‡^: 2.3 kcal mol^–1^) originates from distortion effects (ΔΔ*E*_dist_total_^‡^: 2.1 kcal mol^–1^), while much of the distortion (ΔΔ*E*_sub_^‡^: 1.8 kcal mol^–1^) is due to the twisted chairlike substrate arrangement in the TS leading to the minor stereoisomer (see Supporting Information for additional details).

With this mechanistic background information in hand, we propose a reaction mechanism (Figure 4) starting with the activation of paraformaldehyde by the Brønsted acid catalyst to produce the corresponding aldehydium/*i*IDP ion pair **I-1**. The subsequent nucleophilic attack by the olefin proceeds in an organized fashion (**TS-2**) via a concerted, highly asynchronous mechanism, as suggested by our experimental and computational results. Hence, the confined *i*IDP structure accommodates the incipient benzylic cation in close proximity to an oxygen atom of the poly(oxymethylene) chain, favoring the following cyclization step, producing **I-2**. In addition, this process occurs in an enantioselective fashion due to the chiral enantiopure nature of the *i*IDP anion. After cleavage of the remaining poly(oxymethylene) chain, the corresponding 1,3-dioxane product is obtained.

**Figure 4:**
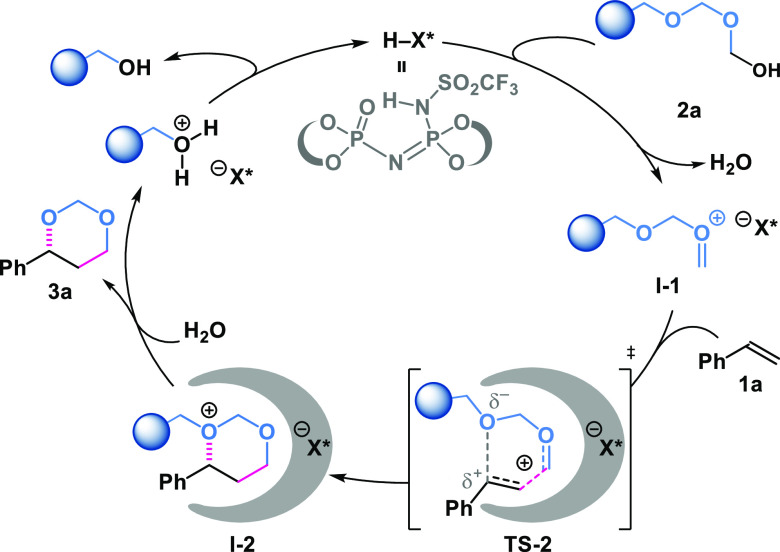
Proposed mechanism of the *i*IDP-catalyzed Prins reaction of aryl olefins and paraformaldehyde.

Here, we have reported an asymmetric intermolecular Prins reaction of aryl olefins with formaldehyde, catalyzed by chiral, confined imino-imidodiphosphates. By means of catalyst design, the reactivity of paraformaldehyde could be controlled. Diverse 1,3-dioxanes were obtained in good yields and good to excellent enantioselectivities, resulting in products of high utility for the chemical synthesis. Isotope labeling experiments and computational calculations suggest that the transformation proceeds via a concerted, highly asynchronous mechanism by addition of the olefin to a formaldehyde oligomer. This design allows now further exploration toward increasingly complex compounds, and extensions of this methodology to other aldehydes and olefins are currently ongoing in our laboratory.

## Supporting Information Available

Experimental details and analytical data for all new compounds, HPLC traces, NMR spectra, and computational studies (optimized structures, and Cartesian coordinates) (PDF)
