# Supplementary material for: The Catalytic Asymmetric Intermolecular Prins Reaction
Source: J Am Chem Soc. 2021 Nov 30;143(49):20598–604. doi: 10.1021/jacs.1c10245 (PMC8679094; doi:10.1021/jacs.1c10245)
Supplement: Supplementary file 1 — ja1c10245_si_001.pdf [file ja1c10245_si_001.pdf]

Supporting Information for

The Catalytic Asymmetric Intermolecular Prins Reaction

C. David Díaz-Oviedo, Rajat Maji, and Benjamin List*

Max-Planck-Institut für Kohlenforschung, Kaiser-Wilhelm-Platz 1, 45470 Mülheim an der Ruhr, Germany
*E-mail: list@kofo.mpg.de

1. Materials and Methods 2
2. Substrate Synthesis 4
3. Reaction Development 8
4. *i*IDP-Catalyzed Intermolecular Prins Reaction 10
5. Current Limitations 19
6. Mechanistic Studies 22
   6.1. Deuterium incorporation: $(HCHO)_n$ and $(DCDO)_n$ 22
   6.2. Stereospecificity: experiments with β-deutero-styrenes 24
   6.3. EWG on the catalyst structure: higher reactivity 25
7. Synthesis of BINOL-derived imino-imidodiphosphates (*i*IDPs) 26
   7.1. Synthesis of 3,3'-disubstituted BINOLs 29
   7.2. Synthesis of imino-imidodiphosphates 34
8. Computational Studies 38
   8.1. "Open" vs. "confined" acid: Understanding the "cavity effect" 38
   8.2. Understanding the Stereoselectivity 41
   8.3. Optimized Cartesian Coordinates (PBE-D3/def2-SVP) 43
9. Copies of NMR spectra 53
10. Copies of HPLC traces 119
11. References 143

1. Materials and Methods

Unless otherwise stated, all reactions were magnetically stirred and conducted in oven-dried (90 °C) or flame-dried glassware in anhydrous solvents under Ar, applying standard Schlenk techniques. Solvents and liquid reagents, as well as solutions of solid or liquid reagents were added via syringes, stainless steel or polyethylene cannulas through rubber septa or through a weak Ar counter-flow. Solid reagents were added through a weak Ar counter-flow. Cooling baths were prepared in Dewar vessels, filled with ice/water (0 °C), cooled acetone (> –78 °C) or dry ice/acetone (–78 °C). Heated oil baths were used for reactions requiring elevated temperatures. Solvents were removed under reduced pressure at 40 °C using a rotary evaporator, and unless otherwise stated, the remaining compound was dried in high vacuum (10^{-3} mbar) at ambient temperature. All given yields are isolated yields of chromatographically and NMR spectroscopically pure materials, unless otherwise stated.
Chemicals were purchased from commercial suppliers (including abcr, Acros, Alfa Aesar, Fluorochem, Merck, and TCI) and used without further purification unless otherwise stated. Et_3N was distilled from $LiAlH_4$ and stored under Ar prior to use. Pyridine was dried and stored over molecular sieves.
Solvents (CyH, CH_2Cl_2, Et_2O, THF, toluene) were dried by distillation from an appropriate drying agent in the technical department of the Max-Planck-Institut für Kohlenforschung and received in Schlenk flasks under Ar.[1] Other anhydrous solvents were purchased from commercial suppliers and used as received.
Reactions were monitored by thin layer chromatography (TLC) on silica gel pre-coated plastic sheets (0.2 mm, Macherey-Nagel). Visualization was accomplished by irradiation with UV light (254 nm and 366 nm) and/or phosphomolybdic acid (PMA) stain and/or Cerium Ammonium Molybdate (CAM) stain and/or permanganate stain.
Column chromatography was carried out using Merck silica gel (60 Å, 230−400 mesh, particle size 0.040−0.063 mm) or aluminum oxide (neutral, activated, Brockmann I, Sigma-Aldrich; activity adjustment individually specified) using technical grade solvents. Elution was accelerated using compressed air. Automated column chromatography was conducted on a Biotage® IsoleraTM ISO-4SW instrument, using SNAP Ultra HP-SphereTM 25 µm chromatography cartridges. All fractions containing a desired substance were combined and concentrated in vacuo, then redissolved in an appropriate solvent and filtered through cotton to remove silica residues.
^{1}H, ^{13}C, ^{11}B, ^{19}F, ^{31}P nuclear magnetic resonance (NMR) spectra were recorded on a Bruker AV-500, AV-400 or DPX-300 spectrometer in a suitable deuterated solvent. The solvent employed and respective measuring frequency are indicated for each experiment. Chemical shifts are reported with Me_4Si serving as a universal reference of all nuclides and with two or one digits after the comma. The resonance multiplicity is described as s (singlet), d (doublet), t (triplet), q (quadruplet), p (pentet), hept (heptet), m (multiplet), and b (broad). All spectra were recorded at 298 K unless otherwise noted, processed with the program MestReNova 11.0, and coupling constants are reported as observed. The residual deuterated solvent signal relative to Me_4Si was used as the internal reference in ^{1}H NMR spectra (e.g. $CDCl_3$ = 7.26 ppm) and are reported as follows: chemical shift in ppm (multiplicity, coupling constant *J* in Hz, number of protons). ^{11}B, ^{13}C, ^{19}F, ^{31}P NMR spectra were referenced according to Ξ-values (IUPAC recommendations 2008)[2] relative to the internal references set in ^{1}H NMR spectra (e.g. ^{13}C: Me_4Si, ^{19}F: CCl_3F, ^{31}P: H_3PO_4; each 0.00 ppm). All spectra are broadband decoupled unless otherwise noted.
Electron impact (EI) mass spectrometry (MS) was performed on a Finnigan MAT 8200 (70 eV) or MAT 8400 (70 eV) spectrometer. Electrospray ionization (ESI) mass spectrometry was conducted on a Bruker ESQ 3000 spectrometer. High resolution mass spectrometry (HRMS) was performed on a Finnigan MAT 95 (EI) or Bruker APEX III FTMS (7T magnet, ESI). The ionization method and mode of

detection employed is indicated for the respective experiment and all masses are reported in atomic units per elementary charge (*m/z*) with an intensity normalized to the most intense peak.
Specific rotations $[\alpha_D^T]$ were measured with a Rudolph RA Autopol IV Automatic Polarimeter at the indicated temperature (*T*) with a sodium lamp (sodium D line, λ = 589 nm). Measurements were performed in an acid resistant 1 mL cell (50 mm length) with concentrations (g/(100 mL)) reported in the corresponding solvent.
High-performance liquid chromatography (HPLC) was performed on Shimadzu LC-20AD liquid chromatograph (SIL-20AC auto sampler, CMB-20A communication bus module, DGU-20A5 degasser, CTO-20AC column oven, SPD-M20A diode array detector), Shimadzu LC-20AB liquid chromatograph (SIL-20ACHT auto sampler, DGU-20A5 degasser, CTO-20AC column oven, SPD-M20A diode array detector), or Shimadzu LC-20AB liquid chromatograph (reversed phase, SIL-20ACHT auto sampler, CTO-20AC column oven, SPD-M20A diode array detector) using Daicel columns with chiral stationary phases. All solvents used were HPLC-grade solvents, purchased from Merck. The column employed and respective solvent mixture are indicated for each experiment.
Gas chromatography (GC) analyses on a chiral stationary phase were performed on HP 6890 and 5890 series instruments (split-mode capillary injection system, flame ionization detector (FID), hydrogen carrier gas). All of these analyses were conducted in the GC department of the Max-Planck-Institut für Kohlenforschung. The conditions employed are described in detail for the individual experiments.
Liquid chromatography-mass spectrometry (LC-MS) was performed on Shimadzu LC-MS 2020 liquid chromatograph. All solvents used were HPLC-grade solvents purchased from Sigma-Aldrich. The column employed, the respective solvent mixture, and the MS parameters are indicated for each experiment.

2. Substrate Synthesis

Substrates **1a**–**c**, **1f**–**l** and **1n** were purchased from Sigma-Aldrich or abcr. Other substrates were prepared by methods indicated below.

General Procedure A: Wittig Olefination

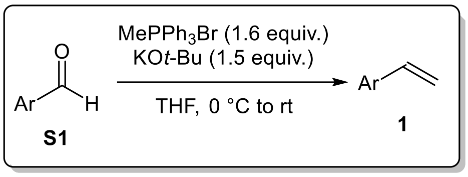

In an oven-dried RB flask, methyltriphenylphosphonium bromide (1.43 g, 4 mmol, 1.6 equiv.) was suspended in THF (9 mL) and cooled to 0 °C (ice/water bath). In one portion, potassium *tert*-butoxide (421 mg, 3.75 mmol, 1.5 equiv.) was added and the mixture was further stirred vigorously for 30 min. At 0 °C, a solution of the aldehyde **S1** (2.5 mmol, 1 equiv.) in THF (5 mL) was added dropwise to the formed phosphorus ylide. The mixture was further stirred at rt for 12 h. After checking full conversion (TLC monitoring), the mixture was diluted with MTBE (50 mL) and distilled water (50 mL), and the aqueous layer was extracted with MTBE (2 x 30 mL). The combined organic layers were washed with brine (1 x 30 mL), dried over anhydrous Na_2SO_4, filtered and concentrated under reduced pressure. Purification by flash column chromatography on silica gel (*n*-pentane/CH_2Cl_2 mixtures) afforded the corresponding olefin **1**.

General Procedure B: Aryl Halide Vinylation

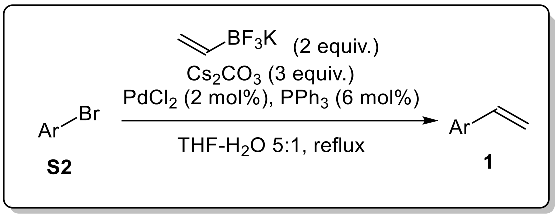

An oven-dried RB flask equipped with a reflux condenser was charged with aryl bromide **S2** (2.5 mmol, 1 equiv.) and THF (9.5 mL). To this was added potassium vinyltrifluoroborate (5 mmol, 2 equiv.), $PdCl_2$ (0.05 mmol, 2 mol%), PPh_3 (0.15 mmol, 6 mol%), Cs_2CO_3 (7.5 mmol, 3 equiv.), and distilled water (2.0 mL). The mixture was stirred at rt and degassed by bubbling Ar for 10 min, then heated to reflux under Ar. After allowing to cool to rt and checking full conversion (TLC monitoring), the mixture was filtered through a short pad of Celite®, washing with MTBE (30 mL). The filtrate was washed with distilled water (1 x 30 mL) and brine (1 x 30 mL), then dried over anhydrous Na_2SO_4, filtered and concentrated under reduced pressure. Purification by flash column chromatography on silica gel (*n*-pentane/CH_2Cl_2 mixtures) afforded the corresponding olefin **1**.

4-vinylphenyl pivalate (1d)

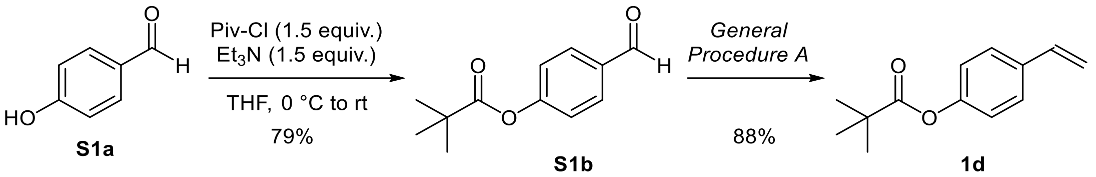

*4-formylphenyl pivalate (**S1b**): (Prepared following a reported procedure[3])* In an oven-dried RB flask, 4-hydroxybenzaldehyde **S1a** (1.22 g, 10 mmol, 1 equiv.) was dissolved in THF (10 mL) and cooled to 0 °C (ice/water bath). Under Ar, triethylamine (2.1 mL, 15 mmol, 1.5 equiv.) and pivaloyl chloride (1.8 mL, 15 mmol, 1.5 equiv., dropwise) were added and the mixture was further stirred at rt for 2 h. After checking full conversion (TLC monitoring), the mixture was treated with satd. aq. NH_4Cl (20 mL) and diluted with MTBE (30 mL), and the aqueous layer was extracted with MTBE (3 x 30 mL). The combined organic layers were washed with brine (1 x 30 mL), dried over anhydrous Na_2SO_4, filtered and concentrated under reduced pressure. Purification by flash column chromatography on silica gel (*i*-hexane/EtOAc 95:5 → 80:20) afforded the corresponding ester **S1b** as a white low-melting solid (1.63 g, 79%).
^1H NMR (501 MHz, $CDCl_3$): δ 9.99 (s, 1H), 7.91 (d, *J* = 8.6 Hz, 2H), 7.24 (d, *J* = 8.6 Hz, 2H), 1.37 (s, 9H). Spectroscopic data was consistent with the values reported in the literature.[4]

*4-vinylphenyl pivalate (**1d**):* Following *General Procedure A*, employing 4-formylphenyl pivalate (**S1b**, 516 mg, 2.5 mmol) as starting material. The crude product was purified by flash column chromatography (eluent: *n*-pentane/CH_2Cl_2 90:10 → 70:30) to give **1d** as a colorless liquid (448 mg, 88%).
^1H NMR (501 MHz, $CDCl_3$): δ 7.41 (d, *J* = 8.5 Hz, 2H), 7.02 (d, *J* = 8.6 Hz, 2H), 6.70 (dd, *J* = 17.6, 10.9 Hz, 1H), 5.70 (dd, *J* = 17.6, 0.9 Hz, 1H), 5.24 (dd, *J* = 10.9, 0.9 Hz, 1H), 1.36 (s, 9H).
^{13}C NMR (126 MHz, $CDCl_3$): δ 177.2 (C), 150.8 (C), 136.1 (CH), 135.3 (C), 127.2 (CH), 121.7 (CH), 114.0 (CH_2), 39.2 (C), 27.3 (CH_3).
ESI-HRMS: calculated for $C_{13}H_{16}O_2Na^+$ ($[M+Na]^+$): 227.1042, found: 227.1046.

***S*-(4-vinylphenyl) 2,2-dimethylpropanethioate (1e)**

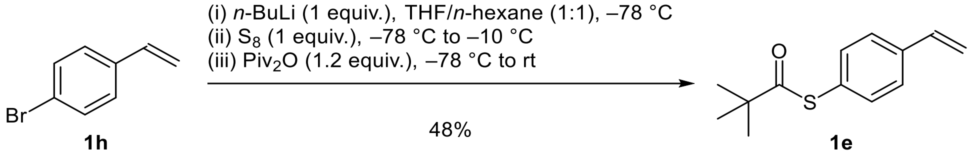

(Adapted from a reported procedure[5]): An oven-dried RB flask was charged with 4-bromostyrene (**1h**, 457 mg, 2.5 mmol, 1 equiv.), dry THF (6 mL) and dry *n*-hexane (6 mL). After cooling the mixture to –78 °C, *n*-BuLi (2.5 M in hexanes, 1.0 mL, 2.5 mmol, 1.0 equiv.) was added dropwise. After stirring at –78 °C for 1.5 h, sulfur powder (80 mg, 2.5 mmol, 1.0 equiv.) was added in one portion and the mixture was stirred at 0 °C for 30 min. Pivalic anhydride (0.56 mL, 2.75 mmol, 1.1 equiv.) was added after cooling the reaction mixture again to –78 °C. After stirring for 1 h at –78 °C, the mixture was slowly warmed up to rt and further stirred for 24 h. The reaction was quenched with 10 mL distilled water and extracted with MTBE (3 x 25 mL). Then, the combined organic phase was washed with brine (1 x 30 mL), dried over anhydrous Na_2SO_4, filtered and concentrated under reduced pressure. Purification by flash column chromatography on silica gel (*n*-pentane/CH_2Cl_2 95:5 → 70:30) afforded the corresponding thioester **1e** as a light-yellow liquid (264 mg, 48%).
^1H NMR (501 MHz, $CDCl_3$): δ 7.43 (d, *J* = 8.4 Hz, 2H), 7.35 (d, *J* = 8.4 Hz, 2H), 6.72 (dd, *J* = 17.5, 10.9 Hz, 1H), 5.79 (dd, *J* = 17.6, 0.8 Hz, 1H), 5.31 (dd, *J* = 10.9, 0.8 Hz, 1H), 1.32 (s, 9H).

^{13}C NMR (126 MHz, $CDCl_3$): δ 204.7 (C), 138.6 (C), 136.3 (CH), 135.2 (CH), 127.5 (C), 127.0 (CH), 115.3 (CH_2), 47.1 (C), 27.6 (CH_3).
ESI-HRMS: calculated for $C_{13}H_{16}OSNa^+$ ($[M+Na]^+$): 243.0814, found: 243.0818.

2-bromo-1-methyl-4-vinylbenzene (**1m**)

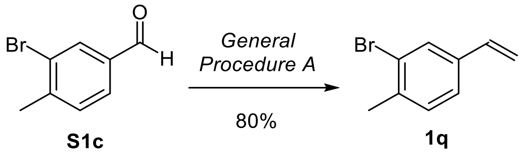

Following *General Procedure A*, employing 3-bromo-4-methylbenzaldehyde (**S1c**, 498 mg, 2.5 mmol) as starting material. The crude product was purified by flash column chromatography (eluent: *n*-pentane/CH_2Cl_2 100:0 → 70:30) to give **1m** as a colorless liquid (396 mg, 80%).
^1H NMR (501 MHz, $CDCl_3$): δ 7.58 (d, *J* = 1.8 Hz, 1H), 7.24 (dd, *J* = 7.8, 1.8 Hz, 1H), 7.18 (d, *J* = 7.8 Hz, 1H), 6.62 (dd, *J* = 17.6, 10.9 Hz, 1H), 5.71 (dd, *J* = 17.6, 0.7 Hz, 1H), 5.24 (d, *J* = 10.8 Hz, 1H), 2.39 (s, 3H).
^{13}C NMR (126 MHz, $CDCl_3$) δ 137.4 (C), 137.3 (C), 135.5 (CH), 130.9 (CH), 130.1 (CH), 125.25 (C), 125.19 (CH), 114.5 (CH_2), 22.8 (CH_3). Spectroscopic data was consistent with the values reported in the literature.[6]
APPI-HRMS: calculated for $C_9H_{10}Br^+$ ($[M+H]^+$): 196.9961, found: 196.9962.

2,2-difluoro-5-vinylbenzo[*d*][1,3]dioxole (**1o**)

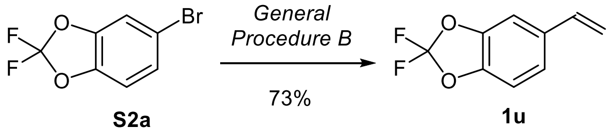

Following *General Procedure B*, employing 5-bromo-2,2-difluoro-1,3-benzodioxole (**S2a**, 593 mg, 2.5 mmol) as starting material. The crude product was purified by flash column chromatography (eluent: *n*-pentane/CH_2Cl_2 100:0 → 70:30) to give **1o** as a colorless liquid (337 mg, 73%).
^1H NMR (501 MHz, $CDCl_3$): δ 7.15 (d, *J* = 1.7 Hz, 1H), 7.07 (dd, *J* = 8.3, 1.7 Hz, 1H), 6.99 (d, *J* = 8.2 Hz, 1H), 6.66 (dd, *J* = 17.5, 10.9 Hz, 1H), 5.66 (d, *J* = 17.5 Hz, 1H), 5.25 (d, *J* = 10.9 Hz, 1H).
^{19}F NMR (471 MHz, $CDCl_3$): δ –50.24 (s, 2F).
^{13}C NMR (126 MHz, $CDCl_3$): δ 144.4 (C), 143.4 (C), 135.7 (CH), 134.3 (C), 131.8 (C, t, *J* = 255.1 Hz), 122.5 (CH), 114.3 (CH_2), 109.4 (CH), 106.6 (CH). Spectroscopic data was consistent with the values reported in the literature.[7]
APPI-HRMS: calculated for $C_9H_7F_2O_2^+$ ($[M+H]^+$): 185.0409, found: 185.0410.

1-allyl-4-vinylbenzene (**1p**)

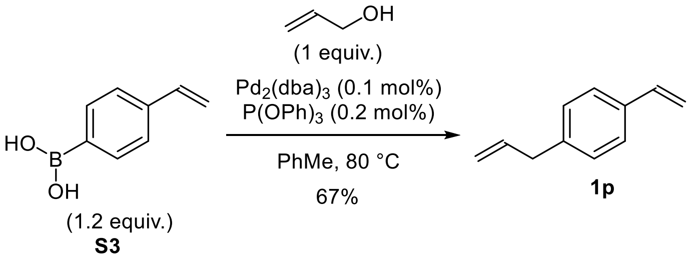

(Adapted from a reported procedure[8]): A two-necked RB flask under Ar atmosphere was charged with $Pd_2(dba)_3$ (4.6 mg, 5 µmol, 0.1 mol%), 4-vinylphenyl boronic acid (**S3**, 888 mg, 6 mmol, 1.2 equiv.) and 1,4-dioxane (6.5 mL). Triphenylphosphite (2.5 µL, 10 µmol, 0.2 mol%) and allyl alcohol (290 mg, 5 mmol, 1 equiv.) were added and the mixture was heated at 80 °C (oil bath) for 6 h. After cooling to rt,

the reaction mixture was diluted with MTBE (50 mL) and washed with brine (1 x 30 mL), dried over anhydrous Na_2SO_4, filtered and concentrated under reduced pressure. Purification by flash column chromatography on silica gel (*n*-pentane) afforded the corresponding olefin **1p** as a colorless liquid (480 mg, 67%).

^1H NMR (501 MHz, CD_2Cl_2): δ 7.35 (d, *J* = 8.1 Hz, 2H), 7.16 (d, *J* = 8.2 Hz, 2H), 6.71 (dd, *J* = 17.6, 10.9 Hz, 1H), 5.97 (ddt, *J* = 16.9, 10.1, 6.7 Hz, 1H), 5.72 (dd, *J* = 17.6, 1.0 Hz, 1H), 5.20 (dd, *J* = 10.9, 1.0 Hz, 1H), 5.13 – 5.03 (m, 2H), 3.38 (d, *J* = 6.8 Hz, 2H).

^{13}C NMR (126 MHz, CD_2Cl_2): δ 140.4 (C), 137.9 (CH), 137.0 (CH), 135.9 (C), 129.1 (CH), 126.6 (CH), 115.9 (CH_2), 113.3 (CH_2), 40.3 (CH_2). Spectroscopic data was consistent with the values reported in the literature.[9]

Styrene-β,β-*d*₂ (1a')

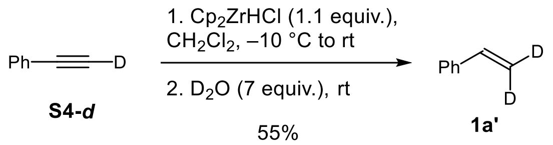

(Adapted from a reported procedure[10]): A two-necked RB flask under Ar atmosphere was charged with phenylacetylene-*d* (**S4-*d***)[10] (1.99 g, 19.5 mmol) and dry CH_2Cl_2 (50 mL). The flask was covered with aluminum foil and the mixture was cooled to –10 °C. Schwartz's Reagent (5.53 g, 21.5 mmol, 1.1 equiv.) was then added in three equal portions in rapid succession (over 2 min). The mixture was allowed to stir at –10 °C for 15 min, then the cold bath was removed and the stirring was continued at rt in the dark for 4 h. The flask was cooled to 0 °C, and the mixture was quenched with D_2O (2.5 mL, 99.9% D, 136.5 mmol, 7 equiv.) and stirred vigorously at rt for 12 h. The mixture was diluted with CH_2Cl_2 (50 mL), followed by the addition of anhydrous Na_2SO_4 and filtration, washing with CH_2Cl_2. The filtrate was concentrated under reduced pressure (400 mbar, water bath of rotavap at 25 °C; no heating, product is volatile) until 5–10 mL remained. *n*-Pentane (50 mL) was added and the mixture was filtered over a Celite® pad to remove the white precipitate; the filter cake was rinsed with *n*-pentane and the filtrate was again concentrated under reduced pressure (400 mbar, 25 °C). Purification by flash column chromatography on silica gel (*n*-pentane; removal of solvent on rotavap at 400 mbar, 25 °C) afforded the corresponding olefin **1a'** as a colorless liquid (1.13 g, 55%). Approx. 97% D-incorporation.

^1H NMR (501 MHz, $CDCl_3$) δ 7.45 – 7.40 (m, 2H), 7.37 – 7.31 (m, 2H), 7.26 (tt, *J* = 6.7, 1.3 Hz, 1H), 6.72 (d, *J* = 2.8 Hz, 1H). Spectroscopic data was consistent with the values reported in the literature.[11]

***trans*-Styrene-(β)-*d* (1q)** and ***cis*-Styrene-(β)-*d* (1r)**

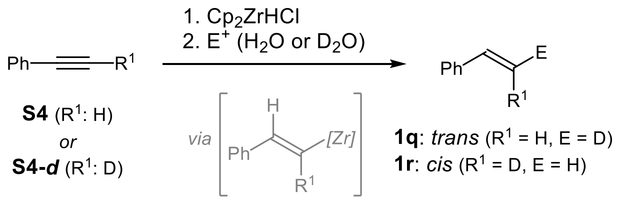

(Adapted from a reported procedure[10]): Prepared in a similar way to **1a'**, choosing between starting alkyne (**S4** or **S4-*d***) and electrophile quench (H_2O or D_2O).

Spectroscopic data was consistent with the values reported in the literature (*trans*-olefin[12] and *cis*-olefin[10]).

3. Reaction Development

At the onset of our studies, we tested the reaction of styrene (**1a**) with paraformaldehyde (**2a**) using as catalysts several chiral Brønsted acids available at that moment in our laboratory (Table S-1).

Table S-1. Initial screening of chiral Brønsted acids.[a]

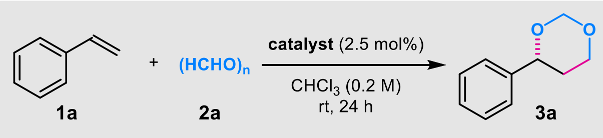

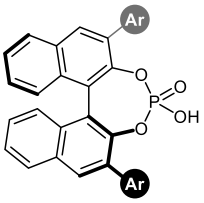

Ar = 4-*t*-Bu-C_6H_4, *or*
Ar = 3,5-$(CF_3)_2C_6H_3$,
CPA
no conversion

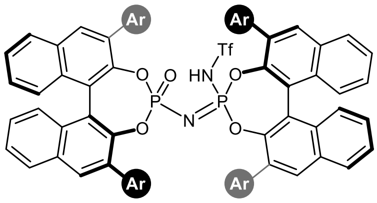

Ar = 2-*i*-Pr-5-Me-C_6H_3

***i*IDP**
2% yield (er = 91:9)

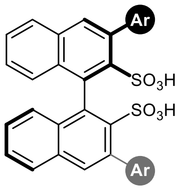

Ar = 3,5-$(CF_3)_2C_6H_3$

BINSA
72% yield (er = 47:53)

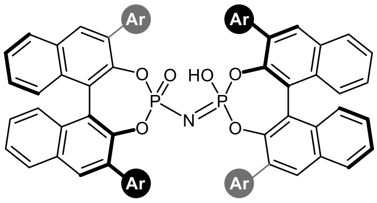

Ar = 2,4,6-Et_3-C_6H_2

IDP
no conversion

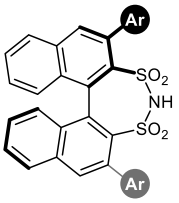

Ar = 3,5-$(CF_3)_2C_6H_3$

DSI
no conversion

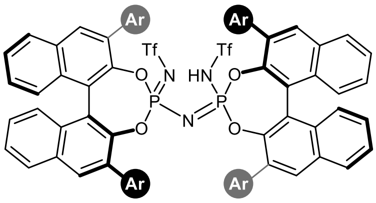

Ar = Ph

IDPi
57% yield (er = 56:44)

[a] 0.025 mmol **1a**, 3 equiv. **2a**, 5 mol% catalyst, in $CHCl_3$ (125 µL) at rt for 48 h. Yields were determined by ^{1}H NMR analysis using Ph_3CH as internal standard. Enantiomeric ratios were determined by HPLC analysis with a chiral stationary phase.

Using the confined imino-imidodiphosphate (*i*IDP) **4a**, which was an optimal catalyst for the Prins cyclization,[13] resulted in this case only in traces of product, yet with good enantioselectivity (82% ee). A more acidic, confined imidodiphosphorimidate (IDPi), as expected, provided the product in better yield (57%), but with a significant decrease in enantioselectivity (12% ee).
Therefore, we dedicated our efforts to increase the yield of the process by making a more acidic *i*IDP catalyst, but without modifying the inner active site. Introducing electron-withdrawing groups in the aryl substituent (at the 3,3' positions) increased the reactivity: catalyst **S11** (*prepared in our laboratory for another project, which will be reported in due course*) afforded **3a** in 24% yield after 24 h at rt, although with decreased enantioselectivity (79.5:20.5 er). Further exploration led to catalyst **4b**, with which (2.5 mol%), the product was obtained in 74% and promising 91.5:8.5 er.

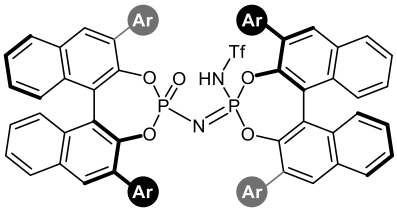

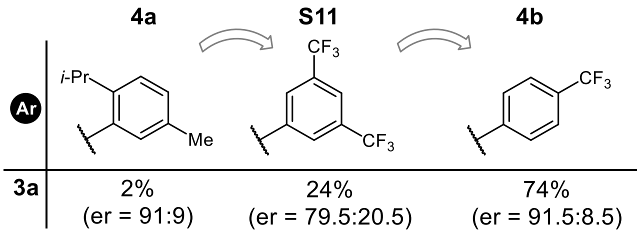

Table S-2. Reaction development for the iIDP-catalyzed Prins reaction of styrene (1a) and paraformaldehyde (2a).

entry	temperature (°C)	solvent	equiv. **2a**	concentration (M)	cat. loading (mol%)	%yield **3a**	er **3a**
1	50	$CHCl_3$	2.5	0.2	2.5	85	89:11
2	25	$CHCl_3$	2.5	0.2	2.5	75	91.5:8.5
3	0	$CHCl_3$	2.5	0.2	2.5	5	93.5:6.5
4	25	CH_2Cl_2	2.5	0.2	2.5	70	88:12
5	25	Et_2O	2.5	0.2	2.5	3	85.5:14.5
6	25	PhMe	2.5	0.2	2.5	61	88:12
7	25	PhH	2.5	0.2	2.5	50	89.5:10.5
8	25	MeCy	2.5	0.2	2.5	41	94:6
9	25	CyH	2.5	0.2	2.5	63	94.5:5.5
10	25	CyH	6	0.2	2.5	64	94:6
11	25	CyH	2.5	0.1	2.5	50	94:6
12	25	CyH	2.5	0.05	2.5	28	94.5:5.5
13	25	CyH	2.5	0.5	2.5	69	92:8
14	25	CyH	2.5	0.1	5.0	72	93:7
15	25	CyH	2.5	0.1	1.0	29	94.5:5.5

As can be extracted from Table S-2, performing the reaction at rt (25 °C), with cyclohexane as solvent, turned out to be the best conditions from the tested ones toward good enantioselectivity.

The absolute configuration of the produced 1,3-dioxane (**3a**) was determined by HPLC analysis, comparing with the retention times of pure enantiomers, which were prepared by acetalization of the commercially available enantiomers of 1-phenylpropane-1,3-diol.[14] Thus, when catalyst (*S*,*S*)-**4b** is used, the major enantiomer of the Prins product **3a** matches on HPLC with the (*R*)-1,3-dioxane.

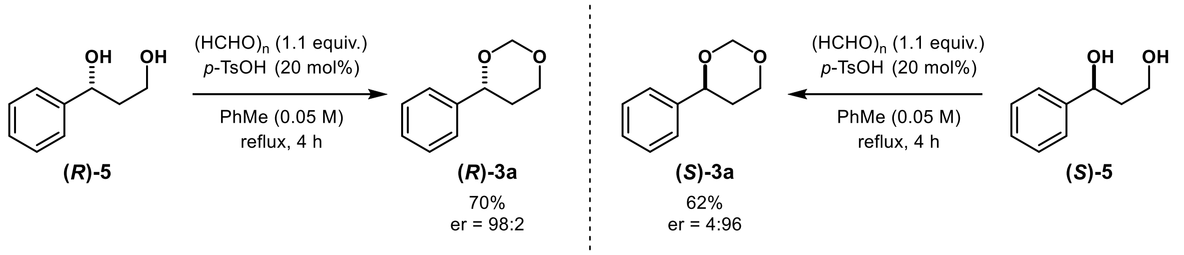

4. *i*IDP-Catalyzed Intermolecular Prins Reaction

General Procedure C: iIDP-Catalyzed Prins reaction of aryl olefins and paraformaldehyde

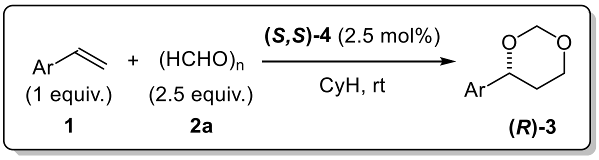

An oven-dried glass vial was charged with paraformaldehyde **2a** (18.8 mg, 0.625 mmol, 2.5 equiv.), (*S*,*S*)-*i*IDP catalyst **4** (62.5 µmol, 2.5 mol%), dry cyclohexane (depending on the concentration, indicated in each case), and olefin **1** (0.25 mmol, 1 equiv.) under Ar. Then, the mixture was vigorously stirred at rt for a certain time (indicated in each case). The reaction was stopped by adding distilled water (10 mL), followed by extraction with MTBE (3 x 20 mL). The combined organic layers were washed with brine (1 x 10 mL), then dried over anhydrous Na_2SO_4, filtered and concentrated under reduced pressure. Purification by flash column chromatography on silica gel (*n*-pentane/MTBE mixtures) afforded the corresponding enantioenriched 4-substituted-1,3-dioxane product **3**.
(for the synthesis of racemates, see General Procedure D at the end of this section)

(*R*)-4-phenyl-1,3-dioxane (**3a**)

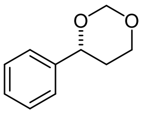

Prepared following *General Procedure C*, from styrene (**1a**, 29 µL, 0.25 mmol), paraformaldehyde **2a** (18.8 mg, 0.625 mmol, 2.5 equiv.), with catalyst (*S*,*S*)-**4c** (10.6 mg, 62.5 µmol, 2.5 mol%), in dry cyclohexane (2.5 mL, 0.1 M) for 72 h. Purification by flash column chromatography (eluent: *n*-pentane/MTBE 100:0 → 95:5) to give **3a** as a colorless liquid (36.4 mg, 89%, er = 95.5:4.5).
^{1}H NMR (501 MHz, $CDCl_3$) δ 7.40 – 7.35 (m, 4H), 7.32 – 7.28 (m, 1H), 5.23 (d, *J* = 6.3 Hz, 1H), 4.91 (d, *J* = 6.4 Hz, 1H), 4.66 (dd, *J* = 11.3, 2.5 Hz, 1H), 4.21 (dd, *J* = 11.5, 4.9 Hz, 1H), 3.89 (td, *J* = 11.9, 2.5 Hz, 1H), 2.11 (dddd, *J* = 13.5, 12.3, 11.3, 4.9 Hz, 1H), 1.76 – 1.70 (m, 1H).
^{13}C NMR (126 MHz, $CDCl_3$) δ 141.6 (C), 128.6 (CH), 128.0 (CH), 125.9 (CH), 94.3 (CH_2), 78.9 (CH), 67.1 (CH_2), 34.1 (CH_2). Spectroscopic data was consistent with the values reported in the literature.[15]
ESI-HRMS: calculated for $C_{10}H_{12}O_2Na^+$ ($[M+Na]^+$): 187.0729, found: 187.0732.
$[\alpha_D^{25}]$ = +32.1 (*c* = 0.746, $CHCl_3$).
HPLC (Chiralpak IB-3 column, Heptane/*i*-PrOH 95:5, 1 mL/min, 25 °C, 220 nm): t_R = 3.6 min (major), 4.5 min (minor).
Racemate synthesis: Following *General Procedure D* (condition A).

(*R*)-4-(4-(chloromethyl)phenyl)-1,3-dioxane (**3b**)

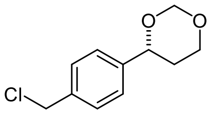

Prepared following *General Procedure C*, from 4-vinylbenzyl chloride (**1b**, 35 µL, 0.25 mmol), paraformaldehyde **2a** (18.8 mg, 0.625 mmol, 2.5 equiv.), with catalyst (*S*,*S*)-**4b** (8.6 mg, 62.5 µmol, 2.5 mol%), in dry cyclohexane (1.25 mL, 0.2 M) for 5 days. Purification by flash column chromatography (eluent: *n*-pentane/MTBE 100:0 → 90:10) to give **3b** as a white solid (30.1 mg, 57%, er = 95.5:4.5).
^{1}H NMR (501 MHz, $CDCl_3$) δ 7.39 (d, *J* = 8.4 Hz, 2H), 7.37 (d, *J* = 8.4 Hz, 2H), 5.22 (d, *J* = 6.4 Hz, 1H), 4.90 (d, *J* = 6.4 Hz, 1H), 4.66 (dd, *J* = 11.3, 2.6 Hz, 1H), 4.59 (s, 2H), 4.21 (dd, *J* = 11.5, 4.9 Hz, 1H), 3.88 (td, *J* = 11.8, 2.4 Hz, 1H), 2.07 (dddd, *J* = 13.4, 12.2, 11.3, 4.8 Hz, 1H), 1.76 – 1.69 (m, 1H).
^{13}C NMR (126 MHz, $CDCl_3$) δ 141.9 (C), 137.1 (C), 128.9 (CH), 126.3 (CH), 94.3 (CH_2), 78.4 (CH), 67.0 (CH_2), 46.1 (CH_2), 34.1 (CH_2).
^{13}C NMR (126 MHz, $CDCl_3$) δ 128.89, 126.26, 94.30, 78.46, 67.00, 46.09, 34.08.
ESI-HRMS: calculated for $C_{11}H_{13}ClO_2Na^+$ ($[M+Na]^+$): 235.0496, found: 235.0498.

[α_D^{25}] = +47.1 (*c* = 0.255, $CHCl_3$).
HPLC (Chiralpak IC-3 column, Heptane/*i*-PrOH 98:2, 1 mL/min, 25 °C, 220 nm): t_R = 7.0 min (minor), 8.0 min (major).
Racemate synthesis: Following *General Procedure D* (condition C).

(*R*)-4-(4-(*tert*-butyl)phenyl)-1,3-dioxane (**3c**)

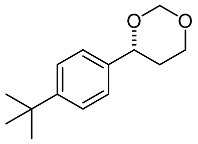

Prepared following *General Procedure C*, from 4-*tert*-butylstyrene (**1c**, 40 µL, 0.25 mmol), paraformaldehyde **2a** (18.8 mg, 0.625 mmol, 2.5 equiv.), with catalyst (*S*,*S*)-**4b** (8.6 mg, 62.5 µmol, 2.5 mol%), in dry cyclohexane (5 mL, 0.05 M) for 72 h. Purification by flash column chromatography (eluent: *n*-pentane/MTBE 100:0 → 95:5) to give **3c** as a white solid (49.1 mg, 89%, er = 91.5:8.5).
^{1}H NMR (501 MHz, $CDCl_3$) δ 7.39 (d, *J* = 8.4 Hz, 2H), 7.31 (d, *J* = 8.2 Hz, 2H), 5.21 (d, *J* = 6.3 Hz, 1H), 4.90 (d, *J* = 6.3 Hz, 1H), 4.63 (dd, *J* = 11.3, 2.5 Hz, 1H), 4.21 (dd, *J* = 11.4, 4.8 Hz, 1H), 3.88 (td, *J* = 11.9, 2.5 Hz, 1H), 2.14 (dddd, *J* = 13.4, 12.2, 11.3, 4.8 Hz, 1H), 1.76 – 1.68 (m, 1H), 1.32 (s, 9H).
^{13}C NMR (126 MHz, $CDCl_3$) δ 151.0 (C), 138.5 (C), 125.8 (CH), 125.5 (CH), 94.4 (CH_2), 78.8 (CH), 67.1 (CH_2), 34.7 (C), 33.8 (CH_2), 31.5 (CH_3). Spectroscopic data was consistent with the values reported in the literature.[15]
ESI-HRMS: calculated for $C_{14}H_{20}O_2Na^+$ ([M+Na]$^+$): 243.1355, found: 243.1358.
[α_D^{25}] = +29.8 (*c* = 0.450, $CHCl_3$).
HPLC (Chiralpak AD-3 column, Heptane/*i*-PrOH 97:3, 0.5 mL/min, 25 °C, 220 nm): t_R = 6.2 min (major), 6.9 min (minor).
Racemate synthesis: Following *General Procedure D* (condition A).

(*R*)-4-(1,3-dioxan-4-yl)phenyl pivalate (**3d**)

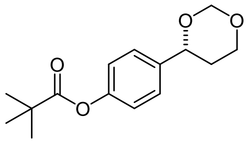

Prepared following *General Procedure C*, from 4-vinylphenyl pivalate (**1d**, 52 µL, 0.25 mmol), paraformaldehyde **2a** (18.8 mg, 0.625 mmol, 2.5 equiv.), with catalyst (*S*,*S*)-**4e** (12.4 mg, 62.5 µmol, 2.5 mol%), in dry cyclohexane (2.5 mL, 0.1 M) for 72 h. Purification by flash column chromatography (eluent: *n*-pentane/MTBE 100:0 → 80:20) to give **3d** as a white solid (54.9 mg, 83%, er = 91:9).
^{1}H NMR (501 MHz, $CDCl_3$) δ 7.38 (d, *J* = 8.5 Hz, 2H), 7.05 (d, *J* = 8.6 Hz, 2H), 5.21 (d, *J* = 6.4 Hz, 1H), 4.89 (d, *J* = 6.4 Hz, 1H), 4.65 (dd, *J* = 11.3, 2.5 Hz, 1H), 4.20 (ddt, *J* = 11.4, 5.0, 1.4 Hz, 1H), 3.87 (td, *J* = 11.9, 2.5 Hz, 1H), 2.07 (dddd, *J* = 13.5, 12.3, 11.3, 4.9 Hz, 1H), 1.75 – 1.68 (m, 1H), 1.35 (s, 9H).
^{13}C NMR (126 MHz, $CDCl_3$) δ 177.2 (C), 150.7 (C), 139.0 (C), 126.9 (CH), 121.6 (CH), 94.3 (CH_2), 78.3 (CH), 67.0 (CH_2), 39.2 (C), 34.2 (CH_2), 27.3 (CH_3).
ESI-HRMS: calculated for $C_{15}H_{20}O_4Na^+$ ([M+Na]$^+$): 287.1254, found: 287.1252.
[α_D^{25}] = +35.3 (*c* = 0.221, $CHCl_3$).
HPLC (Chiralpak IB-3 column, Heptane/*i*-PrOH 98:2, 1 mL/min, 25 °C, 220 nm): t_R = 4.9 min (minor), 5.4 min (major).
Racemate synthesis: Following *General Procedure D* (condition A).

(*R*)-*S*-(4-(1,3-dioxan-4-yl)phenyl) 2,2-dimethylpropanethioate (**3e**)

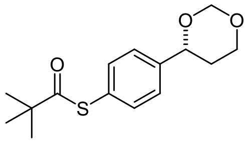

Prepared following *General Procedure C*, from *S*-(4-vinylphenyl) 2,2-dimethylpropanethioate (**1e**, 54 µL, 0.25 mmol), paraformaldehyde **2a** (18.8 mg, 0.625 mmol, 2.5 equiv.), with catalyst (*S*,*S*)-**4e** (12.4 mg, 62.5 µmol, 2.5 mol%), in dry cyclohexane (5 mL, 0.05 M) for 72 h. Purification by flash column chromatography (eluent: *n*-pentane/MTBE 100:0 → 85:15) to give **3e** as a white solid (61.7 mg, 88%, er = 95:5).

^{1}H NMR (501 MHz, $CDCl_3$) δ 7.41 (d, *J* = 8.4 Hz, 2H), 7.38 (d, *J* = 8.7 Hz, 2H), 5.22 (d, *J* = 6.3 Hz, 1H), 4.89 (d, *J* = 6.4 Hz, 1H), 4.67 (dd, *J* = 11.3, 2.6 Hz, 1H), 4.20 (dd, *J* = 11.5, 4.8 Hz, 1H), 3.88 (td, *J* = 11.9, 2.5 Hz, 1H), 2.06 (dddd, *J* = 13.4, 12.1, 11.2, 4.8 Hz, 1H), 1.77 – 1.70 (m, 1H), 1.32 (s, 9H).
^{13}C NMR (126 MHz, $CDCl_3$) δ 204.8 (C), 142.7 (C), 135.1 (CH), 127.5 (C), 126.5 (CH), 94.3 (CH_2), 78.3 (CH), 67.0 (CH_2), 47.1 (C), 34.1 (CH_2), 27.6 (CH_3).
ESI-HRMS: calculated for $C_{15}H_{20}O_3SNa^+$ ($[M+Na]^+$): 303.1025, found: 303.1024.
$[\alpha_D^{25}]$ = +42.1 (*c* = 0.826, $CHCl_3$).
HPLC (Chiralcel OJ-3 column, Heptane/*i*-PrOH 95:5, 1 mL/min, 25 °C, 220 nm): t_R = 12.4 min (minor), 21.2 min (major).
Racemate synthesis: Following *General Procedure D* (condition A).

(*R*)-4-(4-fluorophenyl)-1,3-dioxane (3f)

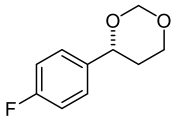

Prepared following *General Procedure C*, from 4-fluorostyrene (**1f**, 30 µL, 0.25 mmol), paraformaldehyde **2a** (18.8 mg, 0.625 mmol, 2.5 equiv.), with catalyst (*S*,*S*)-**4d** (11.2 mg, 62.5 µmol, 2.5 mol%), in dry cyclohexane (5 mL, 0.05 M) for 96 h. Purification by flash column chromatography (eluent: *n*-pentane/MTBE 100:0 → 95:5) to give **3f** as a yellow solid (37.4 mg, 82%, er = 94.5:5.5).
^{1}H NMR (501 MHz, $CDCl_3$) δ 7.35 (dd, *J* = 8.6, 5.5 Hz, 2H), 7.05 (t, *J* = 8.7 Hz, 2H), 5.21 (d, *J* = 6.3 Hz, 1H), 4.89 (d, *J* = 6.4 Hz, 1H), 4.63 (dd, *J* = 11.4, 2.5 Hz, 1H), 4.21 (dd, *J* = 11.6, 4.7 Hz, 1H), 3.87 (td, *J* = 11.9, 2.5 Hz, 1H), 1.74 – 1.67 (m, 1H).
^{19}F NMR (471 MHz, $CDCl_3$) δ –114.62 (s, 1F).
^{13}C NMR (126 MHz, $CDCl_3$) δ 162.5 (d, *J* = 245.8 Hz, C), 137.4 (d, *J* = 3.2 Hz, C), 127.6 (d, *J* = 8.1 Hz, CH), 115.5 (d, *J* = 21.3 Hz, CH), 94.3 (CH_2), 78.2 (CH), 67.0 (CH_2), 34.1 (CH_2). Spectroscopic data was consistent with the values reported in the literature.[16]
ESI-HRMS: calculated for $C_{10}H_{11}FO_2Na^+$ ($[M+Na]^+$): 205.0635, found: 205.0637.
$[\alpha_D^{25}]$ = +34.1 (*c* = 0.369, $CHCl_3$).
HPLC (Chiralpak IC-3 column, Heptane/*i*-PrOH 98:2, 1 mL/min, 25 °C, 220 nm): t_R = 4.5 min (minor), 4.9 min (major).
Racemate synthesis: Following *General Procedure D* (condition B).

(*R*)-4-(4-chlorophenyl)-1,3-dioxane (3g)

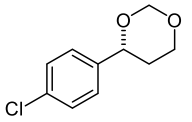

Prepared following *General Procedure C*, from 4-chlorostyrene (**1g**, 30 µL, 0.25 mmol), paraformaldehyde **2a** (18.8 mg, 0.625 mmol, 2.5 equiv.), with catalyst (*S*,*S*)-**4e** (12.4 mg, 62.5 µmol, 2.5 mol%), in dry cyclohexane (1.25 mL, 0.2 M) for 96 h. Purification by flash column chromatography (eluent: *n*-pentane/MTBE 100:0 → 95:5) to give **3g** as a colorless liquid (43.5 mg, 88%, er = 95.5:4.5).
^{1}H NMR (501 MHz, $CDCl_3$) δ 7.34 (d, *J* = 8.6 Hz, 2H), 7.30 (d, *J* = 8.6 Hz, 2H), 5.21 (d, *J* = 6.4 Hz, 1H), 4.88 (d, *J* = 6.4 Hz, 1H), 4.63 (dd, *J* = 11.3, 2.6 Hz, 1H), 4.20 (ddt, *J* = 11.5, 4.9, 1.4 Hz, 1H), 3.87 (td, *J* = 11.9, 2.5 Hz, 1H), 2.04 (dddd, *J* = 13.5, 12.3, 11.3, 4.9 Hz, 1H), 1.74 – 1.67 (m, 1H).
^{13}C NMR (126 MHz, $CDCl_3$) δ 140.2 (C), 133.6 (C), 128.8 (CH), 127.2 (CH), 94.3 (CH_2), 78.1 (CH), 66.9 (CH_2), 34.1 (CH_2). Spectroscopic data was consistent with the values reported in the literature.[15]
ESI-HRMS: calculated for $C_{10}H_{11}ClO_2Na^+$ ($[M+Na]^+$): 221.0340, found: 221.0342.
$[\alpha_D^{25}]$ = +45.1 (*c* = 0.532, $CHCl_3$).
HPLC (Chiralpak IC-3 column, Heptane/*i*-PrOH 97:3, 0.5 mL/min, 25 °C, 220 nm): t_R = 8.0 min (minor), 8.7 min (major).
Racemate synthesis: Following *General Procedure D* (condition B).

(*R*)-4-(4-bromophenyl)-1,3-dioxane (**3h**)

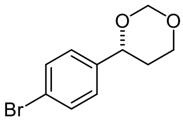

Prepared following *General Procedure C*, from 4-bromostyrene (**1h**, 33 µL, 0.25 mmol), paraformaldehyde **2a** (18.8 mg, 0.625 mmol, 2.5 equiv.), with catalyst (*S*,*S*)-**4e** (12.4 mg, 62.5 µmol, 2.5 mol%), in dry cyclohexane (1.25 mL, 0.2 M) for 5 days. Purification by flash column chromatography (eluent: *n*-pentane/MTBE 100:0 → 95:5) to give **3h** as a white solid (45.3 mg, 74%, er = 95.5:4.5).

^{1}H NMR (501 MHz, $CDCl_3$) δ 7.49 (d, *J* = 8.5 Hz, 2H), 7.25 (d, *J* = 8.4 Hz, 2H), 5.21 (d, *J* = 6.4 Hz, 1H), 4.88 (d, *J* = 6.4 Hz, 1H), 4.61 (dd, *J* = 11.3, 2.6 Hz, 1H), 4.20 (dd, *J* = 11.4, 4.8 Hz, 1H), 3.87 (td, *J* = 11.9, 2.4 Hz, 1H), 2.03 (dddd, *J* = 13.5, 12.3, 11.3, 4.9 Hz, 1H), 1.74 – 1.67 (m, 1H).

^{13}C NMR (126 MHz, $CDCl_3$) δ 140.7 (C), 131.7 (CH), 127.6 (CH), 121.7 (C), 94.3 (CH_2), 78.1 (CH), 66.9 (CH_2), 34.1 (CH_2).

ESI-HRMS: calculated for $C_{10}H_{11}BrO_2Na^+$ ($[M+Na]^+$): 264.9835, found: 264.9836.

$[\alpha_D^{25}]$ = +41.1 (*c* = 0.224, $CHCl_3$).

HPLC (Chiralpak IC-3 column, Heptane/*i*-PrOH 97:3, 0.5 mL/min, 25 °C, 220 nm): t_R = 8.3 min (minor), 9.2 min (major).

Racemate synthesis: Following *General Procedure D* (condition B).

(*R*)-4-(3-fluorophenyl)-1,3-dioxane (**3i**)

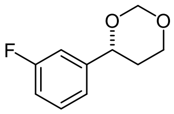

Prepared following *General Procedure C*, from 3-fluorostyrene (**1i**, 30 µL, 0.25 mmol), paraformaldehyde **2a** (18.8 mg, 0.625 mmol, 2.5 equiv.), with catalyst (*S*,*S*)-**4e** (12.4 mg, 62.5 µmol, 2.5 mol%), in dry cyclohexane (1.25 mL, 0.2 M) for 5 days. Purification by flash column chromatography (eluent: *n*-pentane/MTBE 100:0 → 95:5) to give **3i** as a colorless liquid (23.6 mg, 52%, er = 96.5:3.5).

^{1}H NMR (501 MHz, $CDCl_3$) δ 7.32 (td, *J* = 8.0, 5.9 Hz, 1H), 7.15 – 7.07 (m, 2H), 6.98 (tdd, *J* = 8.4, 2.6, 1.0 Hz, 1H), 5.22 (d, *J* = 6.4 Hz, 1H), 4.89 (d, *J* = 6.4 Hz, 1H), 4.65 (dd, *J* = 11.3, 2.6 Hz, 1H), 4.20 (ddt, *J* = 11.5, 4.9, 1.4 Hz, 1H), 3.87 (td, *J* = 11.9, 2.5 Hz, 1H), 2.05 (dddd, *J* = 13.5, 12.2, 11.3, 4.9 Hz, 1H), 1.77 – 1.71 (m, 1H).

^{19}F NMR (471 MHz, $CDCl_3$) δ –112.87 (s, 1F).

^{13}C NMR (126 MHz, $CDCl_3$) δ 163.1 (d, *J* = 245.9 Hz, C), 144.2 (d, *J* = 7.3 Hz, C), 130.1 (d, *J* = 8.0 Hz, CH), 121.3 (d, *J* = 2.8 Hz, CH), 114.7 (d, *J* = 20.8 Hz, CH), 112.9 (d, *J* = 22.4 Hz, CH), 94.2 (CH_2), 78.0 (d, *J* = 2.1 Hz, CH), 66.9 (CH_2), 34.0 (CH_2).

ESI-HRMS: calculated for $C_{10}H_{11}FO_2Na^+$ ($[M+Na]^+$): 205.0635, found: 205.0637.

$[\alpha_D^{25}]$ = +38.3 (*c* = 0.626, $CHCl_3$).

HPLC (Chiralcel OJ-3 column, Heptane/*i*-PrOH 95:5, 1 mL/min, 25 °C, 220 nm): t_R = 5.8 min (minor), 6.4 min (major).

Racemate synthesis: Following *General Procedure D* (condition C).

(*R*)-4-(3-bromophenyl)-1,3-dioxane (**3j**)

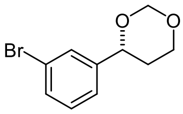

Prepared following *General Procedure C*, from 3-bromostyrene (**1j**, 33 µL, 0.25 mmol), paraformaldehyde **2a** (18.8 mg, 0.625 mmol, 2.5 equiv.), with catalyst (*S*,*S*)-**4b'** (12.9 mg, 62.5 µmol, 2.5 mol%), in dry cyclohexane (1.25 mL, 0.2 M) for 5 days. Purification by flash column chromatography (eluent: *n*-pentane/MTBE 100:0 → 95:5) to give **3j** as a colorless liquid (25.0 mg, 41%, er = 96:4).

^{1}H NMR (501 MHz, $CDCl_3$) δ 7.54 (t, *J* = 1.9 Hz, 1H), 7.42 (ddd, *J* = 7.8, 2.0, 1.2 Hz, 1H), 7.29 (d, *J* = 7.9 Hz, 1H), 7.23 (t, *J* = 7.8 Hz, 1H), 5.21 (d, *J* = 6.4 Hz, 1H), 4.88 (d, *J* = 6.4 Hz, 1H), 4.62 (dd, *J* = 11.3, 2.6 Hz, 1H), 4.20 (dd, *J* = 11.5, 4.8 Hz, 1H), 3.87 (td, *J* = 11.9, 2.4 Hz, 1H), 2.04 (dddd, *J* = 13.4, 12.1, 11.3, 4.8 Hz, 1H), 1.75 – 1.70 (m, 1H).

^{13}C NMR (126 MHz, $CDCl_3$) δ 143.9 (C), 131.0 (CH), 130.2 (CH), 129.0 (CH), 124.4 (CH), 122.8 (C), 94.2 (CH_2), 78.0 (CH), 66.9 (CH_2), 34.1 (CH_2).
ESI-HRMS: calculated for $C_{10}H_{11}BrO_2Na^+$ ($[M+Na]^+$): 264.9835, found: 264.9835.
$[\alpha_D^{25}]$ = +35.5 (*c* = 0.609, $CHCl_3$).
HPLC (Chiralpak IC-3 column, Heptane/*i*-PrOH 97:3, 0.5 mL/min, 25 °C, 220 nm): t_R = 8.0 min (minor), 8.6 min (major).
Racemate synthesis: Following *General Procedure D* (condition C).

(*R*)-4-(*m*-tolyl)-1,3-dioxane (**3k**)

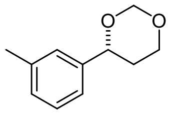

Prepared following *General Procedure C*, from 3-methylstyrene (**1k**, 33 µL, 0.25 mmol), paraformaldehyde **2a** (18.8 mg, 0.625 mmol, 2.5 equiv.), with catalyst (*S*,*S*)-**4e** (12.4 mg, 62.5 µmol, 2.5 mol%), in dry cyclohexane (5 mL, 0.05 M) for 72 h. Purification by flash column chromatography (eluent: *n*-pentane/MTBE 100:0 → 95:5) to give **3k** as a colorless liquid (39.4 mg, 88%, er = 93:7).
^1H NMR (501 MHz, $CDCl_3$) δ 7.25 (t, *J* = 7.6 Hz, 1H), 7.21 (s, 1H), 7.16 (d, *J* = 7.7 Hz, 1H), 7.11 (d, *J* = 7.6 Hz, 1H), 5.22 (d, *J* = 6.4 Hz, 1H), 4.90 (d, *J* = 6.3 Hz, 1H), 4.62 (dd, *J* = 11.3, 2.6 Hz, 1H), 4.21 (dd, *J* = 11.4, 4.9 Hz, 1H), 3.88 (td, *J* = 11.9, 2.5 Hz, 1H), 2.36 (s, 3H), 2.11 (dddd, *J* = 13.5, 12.3, 11.3, 4.8 Hz, 1H), 1.75 – 1.68 (m, 1H).
^{13}C NMR (126 MHz, $CDCl_3$) δ 141.5 (C), 138.3 (C), 128.7 (CH), 128.5 (CH), 126.6 (CH), 123.0 (CH), 94.4 (CH_2), 79.0 (CH), 67.1 (CH_2), 34.1 (CH_2), 21.6 (CH_3).
ESI-HRMS: calculated for $C_{11}H_{14}O_2Na^+$ ($[M+Na]^+$): 201.0886, found: 201.0887.
$[\alpha_D^{25}]$ = +40.5 (*c* = 0.430, $CHCl_3$).
HPLC (Chiralpak IB-3 column, Heptane/*i*-PrOH 95:5, 1 mL/min, 25 °C, 220 nm): t_R = 3.4 min (major), 4.3 min (minor).
Racemate synthesis: Following *General Procedure D* (condition A).

(*R*)-4-(3-methoxyphenyl)-1,3-dioxane (**3l**)

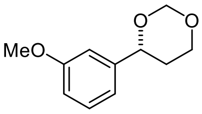

Prepared following *General Procedure C*, from 3-vinylanisole (**1l**, 34 µL, 0.25 mmol), paraformaldehyde **2a** (18.8 mg, 0.625 mmol, 2.5 equiv.), with catalyst (*S*,*S*)-**4e** (12.4 mg, 62.5 µmol, 2.5 mol%), in dry cyclohexane (5 mL, 0.05 M) for 72 h. Purification by flash column chromatography (eluent: *n*-pentane/MTBE 100:0 → 90:10) to give **3l** as a colorless oil (42.3 mg, 87%, er = 93:7).
^1H NMR (501 MHz, $CDCl_3$) δ 7.27 (t, *J* = 7.8 Hz, 1H), 6.97 – 6.91 (m, 2H), 6.84 (ddd, *J* = 8.3, 2.6, 1.0 Hz, 1H), 5.22 (d, *J* = 6.3 Hz, 1H), 4.90 (d, *J* = 6.3 Hz, 1H), 4.63 (dd, *J* = 11.3, 2.6 Hz, 1H), 4.20 (dd, *J* = 11.4, 4.8 Hz, 1H), 3.88 (td, *J* = 11.9, 2.5 Hz, 1H), 3.82 (s, 3H), 2.09 (dddd, *J* = 13.5, 12.2, 11.3, 4.9 Hz, 1H), 1.75 – 1.70 (m, 1H).
^{13}C NMR (126 MHz, $CDCl_3$) δ 159.9 (C), 143.2 (C), 129.6 (CH), 118.2 (CH), 113.6 (CH), 111.3 (CH), 94.3 (CH_2), 78.8 (CH), 67.1 (CH_2), 55.4 (CH_3), 34.1 (CH_2).
ESI-HRMS: calculated for $C_{11}H_{14}O_3Na^+$ ($[M+Na]^+$): 217.0835, found: 217.0837.
$[\alpha_D^{25}]$ = +42.2 (*c* = 0.465, $CHCl_3$).
HPLC (Chiralpak IB-3 column, Heptane/*i*-PrOH 95:5, 1 mL/min, 25 °C, 220 nm): t_R = 4.9 min (major), 6.4 min (minor).
Racemate synthesis: Following *General Procedure D* (condition A).

(*R*)-4-(3-bromo-4-methylphenyl)-1,3-dioxane (**3m**)

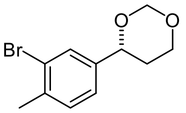

Prepared following *General Procedure C*, from 2-bromo-1-methyl-4-vinylbenzene (**1m**, 37 µL, 0.25 mmol), paraformaldehyde **2a** (18.8 mg, 0.625 mmol, 2.5 equiv.), with catalyst (*S*,*S*)-**4b** (8.6 mg, 62.5 µmol, 2.5 mol%), in dry cyclohexane (1.25 mL,

0.2 M) for 5 days. Purification by flash column chromatography (eluent: *n*-pentane/MTBE 100:0 → 95:5) to give **3m** as a colorless oil (43.2 mg, 67%, er = 94:6).
^{1}H NMR (501 MHz, $CDCl_3$) δ 7.56 (d, *J* = 1.6 Hz, 1H), 7.22 (d, *J* = 7.8 Hz, 1H), 7.19 (dd, *J* = 7.8, 1.6 Hz, 1H), 5.20 (d, *J* = 6.4 Hz, 1H), 4.87 (d, *J* = 6.4 Hz, 1H), 4.59 (dd, *J* = 11.3, 2.6 Hz, 1H), 4.19 (dd, *J* = 11.5, 4.9 Hz, 1H), 3.86 (td, *J* = 11.9, 2.4 Hz, 1H), 2.38 (s, 3H), 2.05 (dddd, *J* = 13.5, 12.2, 11.3, 4.9 Hz, 1H), 1.73 – 1.68 (m, 1H).
^{13}C NMR (126 MHz, $CDCl_3$) δ 141.1 (C), 137.4 (C), 130.9 (CH), 129.9 (CH), 125.1 (C), 124.7 (CH), 94.3 (CH_2), 77.8 (CH), 66.9 (CH_2), 34.0 (CH_2), 22.7 (CH_3).
ESI-HRMS: calculated for $C_{11}H_{13}BrO_2Na^+$ ($[M+Na]^+$): 278.9991, found: 278.9993.
$[\alpha_D^{25}]$ = +32.0 (*c* = 0.651, $CHCl_3$).
HPLC (Chiralcel OJ-3 column, Heptane/*i*-PrOH 95:5, 1 mL/min, 25 °C, 220 nm): t_R = 6.2 min (minor), 7.6 min (major).
Racemate synthesis: Following *General Procedure D* (condition C).

(*R*)-4-(naphthalen-2-yl)-1,3-dioxane (**3n**)

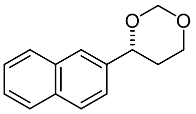

Prepared following *General Procedure C*, from 2-vinylnaphthalene (**1n**, 38.5 mg, 0.25 mmol), paraformaldehyde **2a** (18.8 mg, 0.625 mmol, 2.5 equiv.), with catalyst (*S*,*S*)-**4e** (12.4 mg, 62.5 µmol, 2.5 mol%), in dry cyclohexane (10 mL, 0.025 M) for 72 h. Purification by flash column chromatography (eluent: *n*-pentane/MTBE 100:0 → 90:10) to give **3n** as a white solid (42.1 mg, 79%, er = 90.5:9.5).
^{1}H NMR (501 MHz, $CDCl_3$) δ 7.88 – 7.81 (m, 4H), 7.53 – 7.44 (m, 3H), 5.29 (d, *J* = 6.4 Hz, 1H), 4.97 (d, *J* = 6.4 Hz, 1H), 4.83 (dd, *J* = 11.3, 2.6 Hz, 1H), 4.25 (dd, *J* = 11.4, 4.8 Hz, 1H), 3.94 (td, *J* = 11.8, 2.5 Hz, 1H), 2.25 – 2.13 (m, 1H), 1.85 – 1.79 (m, 1H).
^{13}C NMR (126 MHz, $CDCl_3$) δ 139.0 (C), 133.4 (C), 133.2 (C), 128.4 (CH), 128.2 (CH), 127.8 (CH), 126.3 (CH), 126.1 (CH), 124.6 (CH), 124.0 (CH), 94.4 (CH_2), 78.9 (CH), 67.1 (CH_2), 34.2 (CH_2). Spectroscopic data was consistent with the values reported in the literature.[15]
ESI-HRMS: calculated for $C_{14}H_{14}O_2Na^+$ ($[M+Na]^+$): 237.0886, found: 237.0886.
$[\alpha_D^{25}]$ = +21.0 (*c* = 0.591, $CHCl_3$).
HPLC (Chiralpak IB-3 column, Heptane/*i*-PrOH 80:20, 1 mL/min, 25 °C, 274 nm): t_R = 3.8 min (minor), 5.6 min (major).
Racemate synthesis: Following *General Procedure D* (condition A).

(*R*)-5-(1,3-dioxan-4-yl)-2,2-difluorobenzo[*d*][1,3]dioxole (**3o**)

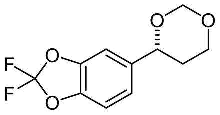

Prepared following *General Procedure C*, from 2,2-difluoro-5-vinylbenzo[*d*][1,3]dioxole (**1o**, 37 µL, 0.25 mmol), paraformaldehyde **2a** (18.8 mg, 0.625 mmol, 2.5 equiv.), with catalyst (*S*,*S*)-**4d** (11.2 mg, 62.5 µmol, 2.5 mol%), in dry cyclohexane (5 mL, 0.05 M) for 72 h. Purification by flash column chromatography (eluent: *n*-pentane/MTBE 100:0 → 90:10) to give **3o** as a colorless liquid (35.0 mg, 57%, er = 94.5:5.5).
^{1}H NMR (501 MHz, $CDCl_3$) δ 7.14 (d, *J* = 1.6 Hz, 1H), 7.06 (dd, *J* = 8.2, 1.6 Hz, 1H), 7.02 (d, *J* = 8.2 Hz, 1H), 5.20 (d, *J* = 6.4 Hz, 1H), 4.88 (d, *J* = 6.4 Hz, 1H), 4.63 (dd, *J* = 11.3, 2.6 Hz, 1H), 4.20 (dd, *J* = 11.5, 4.9 Hz, 1H), 3.86 (td, *J* = 11.9, 2.5 Hz, 1H), 2.03 (dddd, *J* = 13.4, 12.1, 11.2, 4.9 Hz, 1H), 1.74 – 1.68 (m, 1H).
^{19}F NMR (471 MHz, $CDCl_3$) δ –50.03 (d, *J* = 3.5 Hz, 2F).
^{13}C NMR (126 MHz, $CDCl_3$) δ 144.1 (C), 143.3 (C), 138.1 (C), 131.8 (t, *J* = 254.9 Hz, C), 121.0 (CH), 109.3 (CH), 107.6 (CH), 94.3 (CH_2), 78.1 (CH), 66.9 (CH_2), 34.3 (CH_2).
EI-HRMS: calculated for $C_{11}H_{10}F_2O_4^+$ ($[M]^+$): 244.0540, found: 244.0542.
$[\alpha_D^{25}]$ = +34.5 (*c* = 0.522, $CHCl_3$).

HPLC (Chiralcel OJ-3 column, Heptane/*i*-PrOH 95:5, 1 mL/min, 25 °C, 220 nm): t_R = 5.2 min (major), 5.6 min (major).
Racemate synthesis: Following *General Procedure D* (condition A).

(*R*)-4-(4-allylphenyl)-1,3-dioxane (**3p**)

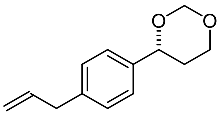

Prepared following *General Procedure C*, from 1-allyl-4-vinylbenzene (**1p**, 40 µL, 0.25 mmol), paraformaldehyde **2a** (18.8 mg, 0.625 mmol, 2.5 equiv.), with catalyst (*S*,*S*)-**4b** (8.6 mg, 62.5 µmol, 2.5 mol%), in dry cyclohexane (5 mL, 0.05 M) for 72 h. Purification by flash column chromatography (eluent: *n*-pentane/MTBE 100:0 → 95:5) to give **3p** as a colorless liquid (45.3 mg, 89%, er = 91:9).
^{1}H NMR (501 MHz, $CDCl_3$) δ 7.31 (d, *J* = 8.1 Hz, 2H), 7.19 (d, *J* = 8.2 Hz, 2H), 5.96 (ddt, *J* = 16.9, 10.1, 6.7 Hz, 1H), 5.21 (d, *J* = 6.3 Hz, 1H), 5.09 (dq, *J* = 9.5, 1.7 Hz, 1H), 5.06 (p, *J* = 1.6 Hz, 1H), 4.90 (d, *J* = 6.3 Hz, 1H), 4.63 (dd, *J* = 11.3, 2.5 Hz, 1H), 4.21 (dd, *J* = 11.4, 4.9 Hz, 1H), 3.88 (td, *J* = 11.8, 2.4 Hz, 1H), 3.39 (d, *J* = 6.7 Hz, 2H), 2.11 (dddd, *J* = 13.4, 12.2, 11.3, 4.8 Hz, 1H), 1.73 – 1.68 (m, 1H).
^{13}C NMR (126 MHz, $CDCl_3$) δ 139.8 (C), 139.4 (C), 137.5 (CH), 128.8 (CH), 126.1 (CH), 116.0 (CH_2), 94.4 (CH_2), 78.8 (CH), 67.1 (CH_2), 40.0 (CH_2), 34.0 (CH_2).
ESI-HRMS: calculated for $C_{13}H_{16}O_2Na^+$ ([M+Na]$^+$): 227.1042, found: 227.1041.
[α_D^{25}] = +29.4 (*c* = 0.639, $CHCl_3$).
HPLC (Chiralpak IC-3 column, Heptane/*i*-PrOH 97:3, 0.5 mL/min, 25 °C, 220 nm): t_R = 9.4 min (minor), 10.4 min (major).
Racemate synthesis: Following *General Procedure D* (condition A).

(*R*)-4-phenyl-1,3-dioxane-5,5-*d*$_2$ (**3a(*d*$_2$)**)

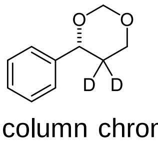

Prepared following *General Procedure C*, from styrene-β,β-*d*$_2$ (**1a'**, 32 µL, 0.25 mmol), paraformaldehyde **2a** (18.8 mg, 0.625 mmol, 2.5 equiv.), with catalyst (*S*,*S*)-**4c** (10.6 mg, 62.5 µmol, 2.5 mol%), in dry cyclohexane (2.5 mL, 0.1 M) for 72 h. Purification by flash column chromatography (eluent: *n*-pentane/MTBE 100:0 → 95:5) to give **3a(*d*$_2$)** as a colorless liquid (30.4 mg, 73%, er = 95:5).
^{1}H NMR (501 MHz, $CDCl_3$) δ 7.41 – 7.33 (m, 4H), 7.33 – 7.26 (m, 1H), 5.23 (d, *J* = 6.3 Hz, 1H), 4.90 (d, *J* = 6.4 Hz, 1H), 4.65 (s, 1H), 4.21 (d, *J* = 11.4 Hz, 1H), 3.88 (dt, *J* = 11.5, 1.9 Hz, 1H).
^{13}C NMR (126 MHz, $CDCl_3$) δ 141.6 (C), 128.6 (CH), 128.0 (CH), 125.9 (CH), 94.3 (CH_2), 78.8 (CH), 67.0 (CH_2). *The carbon atom from the CD$_2$ group was not visible.*
EI-HRMS: calculated for $C_{10}H_{10}D_2O_2^+$ ([M]$^+$): 166.0957, found: 166.0958.
[α_D^{25}] = +27.5 (*c* = 0.793, $CHCl_3$).
HPLC (Chiralpak IB-3 column, Heptane/*i*-PrOH 95:5, 1 mL/min, 25 °C, 220 nm): t_R = 3.6 min (major), 4.5 min (minor).
Racemate synthesis: Following *General Procedure D* (condition A).

(*R*)-4-phenyl-1,3-dioxane-2,2,6,6-*d*$_4$ (**3a(*d*$_4$)**)

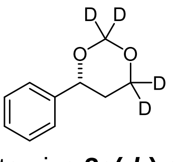

Prepared following *General Procedure C*, from styrene (**1a**, 29 µL, 0.25 mmol), paraformaldehyde-*d*$_2$ **2a'** (20.0 mg, 0.625 mmol, 2.5 equiv.), with catalyst (*S*,*S*)-**4c** (10.6 mg, 62.5 µmol, 2.5 mol%), in dry cyclohexane (2.5 mL, 0.1 M) for 72 h. Purification by flash column chromatography (eluent: *n*-pentane/MTBE 100:0 → 95:5) to give **3a(*d*$_4$)** as a colorless liquid (26.1 mg, 62%, er = 95:5).
^{1}H NMR (501 MHz, $CDCl_3$) δ 7.40 – 7.34 (m, 4H), 7.33 – 7.28 (m, 1H), 4.66 (dd, *J* = 11.3, 2.6 Hz, 1H), 2.09 (ddt, *J* = 13.2, 11.2, 1.8 Hz, 1H), 1.72 (dd, *J* = 13.5, 2.6 Hz, 1H).

^{13}C NMR (126 MHz, $CDCl_3$) δ 141.6 (C), 128.6 (CH), 128.0 (CH), 125.9 (CH), 78.8 (CH), 33.9 (CH_2). *The carbon atoms from CD_2 groups were not visible.*
EI-HRMS: calculated for $C_{10}H_8D_4O_2^+$ ([M]$^+$): 168.1083, found: 168.1084.
$[\alpha_D^{25}]$ = +43.5 (*c* = 0.409, $CHCl_3$).
HPLC (Chiralpak IB-3 column, Heptane/*i*-PrOH 95:5, 1 mL/min, 25 °C, 220 nm): t_R = 3.6 min (major), 4.5 min (minor).
Racemate synthesis: Following *General Procedure D* (condition A).

(*R*)-4-phenyl-1,3-dioxane-2,2,5,5,6,6-*d*$_6$ (3a(*d*$_6$))

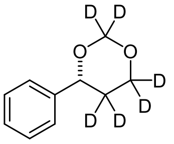

Prepared following *General Procedure C*, from styrene-β,β-*d*$_2$ (**1a'**, 32 µL, 0.25 mmol), paraformaldehyde-*d*$_2$ **2a'** (20.0 mg, 0.625 mmol, 2.5 equiv.), with catalyst (*S*,*S*)-**4c** (10.6 mg, 62.5 µmol, 2.5 mol%), in dry cyclohexane (2.5 mL, 0.1 M) for 72 h. Purification by flash column chromatography (eluent: *n*-pentane/MTBE 100:0 → 95:5) to give **3a(*d*$_6$)** as a colorless liquid (23.8 mg, 56%, er = 95:5).
^{1}H NMR (501 MHz, $CDCl_3$) δ 7.40 – 7.34 (m, 4H), 7.32 – 7.28 (m, 1H), 4.65 (s, 1H).
^{13}C NMR (126 MHz, $CDCl_3$) δ 141.6 (C), 128.6 (CH), 128.0 (CH), 125.9 (CH), 78.7 (CH). *The carbon atoms from CD_2 groups were not visible.*
ESI-HRMS: calculated for $C_{10}H_6D_6O_2Na^+$ ([M+Na]$^+$): 193.1106, found: 193.1110.
$[\alpha_D^{25}]$ = +44.2 (*c* = 0.371, $CHCl_3$).
HPLC (Chiralpak IB-3 column, Heptane/*i*-PrOH 95:5, 1 mL/min, 25 °C, 220 nm): t_R = 3.6 min (major), 4.5 min (minor).
Racemate synthesis: Following *General Procedure D* (condition A).

General Procedure D: Synthesis of racemates

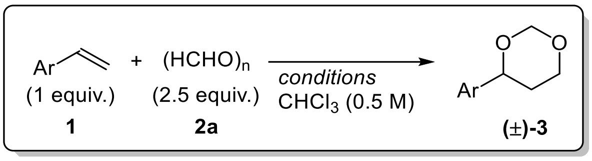

condition A: *p*-TsOH (20 mol%), rt
condition B: 2,4-DNBSA (20 mol%), 50 °C
condition C: Amberlyst 15 H$^+$ (0.1 g/mmol), 60 °C
2,4-DNBSA: 2,4-dinitrobenzenesulfonic acid

An oven-dried glass vial was charged with paraformaldehyde **2a** (18.8 mg, 0.625 mmol, 2.5 equiv.), and with catalyst:

-either 20 mol% *p*-toluenesulfonic acid *(condition A)*,
-or 20 mol% 2,4-dinitrobenzensulfonic acid hydrate *(condition B)*,
-or 25 mg Amberlyst 15 H$^+$ *(condition C)*.

Then to it was added dry chloroform (0.5 mL), and olefin **1** (0.25 mmol, 1 equiv.). The mixture was vigorously stirred, either at rt *(condition A)* or at 60 °C *(conditions B–C)* for 24–72 h. The reaction was stopped by adding distilled water (10 mL), followed by extraction with MTBE (3 x 20 mL). The combined organic layers were washed with brine (1 x 10 mL), then dried over anhydrous Na_2SO_4, filtered and concentrated under reduced pressure. Purification by flash column chromatography on silica gel (*n*-pentane/MTBE mixtures) afforded the corresponding racemic 1,3-dioxane product **3**.

*1,3-Dioxane Ring Opening: Preparation of 1,3-Diol **5***

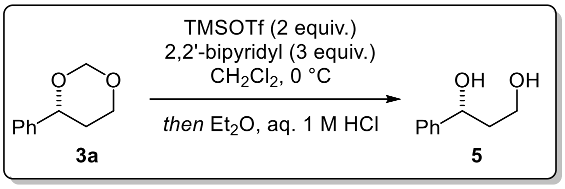

(Adapted from a reported procedure[17]): An oven-dried RB flask was charged with **3a** (164 mg, 1 mmol, er = 95.5:4.5), dry CH_2Cl_2 (2 mL) and 2,2'-bipyridyl (469 mg, 3 mmol, 3 equiv.). After cooling the mixture at 0 °C, TMSOTf (0.36 mL, 2 mmol, 2 equiv.) was added dropwise followed by further stirring at 0 °C for 2 h. After checking full conversion of **3a** by TLC, Et_2O (10 mL) and aq. 1 M HCl (10 mL, 10 mmol, 10 equiv.) were added in one portion at 0 °C and the reaction mixture was stirred for 1.5 h. The mixture was then extracted with EtOAc (3 x 30 mL) and the combined organic phase was washed with satd. aq. $NaHCO_3$ (1 x 30 mL), dried over anhydrous Na_2SO_4, filtered and concentrated under reduced pressure. Purification by flash column chromatography on silica gel (*i*-hexane/EtOAc 90:10 → 60:40) afforded the corresponding 1,3-diol **5** as a colorless oil (134 mg, 88%, er = 95:5).

^{1}H NMR (501 MHz, $CDCl_3$) δ 7.39 – 7.33 (m, 4H), 7.32 – 7.25 (m, 1H), 4.96 (dd, J = 8.8, 3.7 Hz, 1H), 3.89 – 3.83 (m, 2H), 2.08 – 1.89 (m, 2H). Spectroscopic data was consistent with the values reported in the literature.[18]

HPLC (Chiralpak IC-3 column, Heptane/*i*-PrOH 95:5, 1 mL/min, 25 °C, 220 nm): t_R = 16.3 min (minor), 22.8 min (major).

5. Current Limitations

Several substrates proved to be challenging, since they are at the edges of the reactivity: some of them are either too reactive, whereas others display very low reactivity. Other substrates present moderate to good reactivity, but the enantioselectivity observed with the tested catalysts was not satisfactory. In any case, these substrates require further studies and optimization.

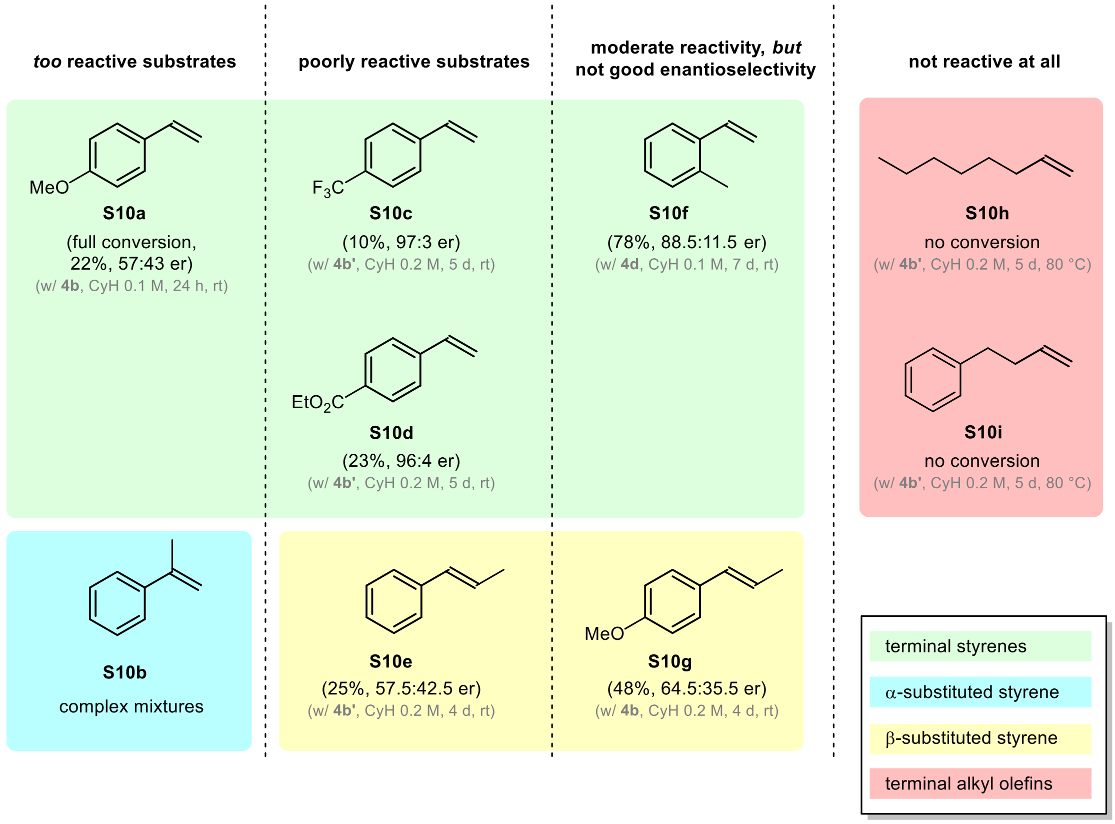

Figure S-1. Current substrate limitations of the iIDP-catalyzed Prins reaction.

Terminal Styrenes

Terminal styrenes containing strong EDG or EWG on the aromatic ring remained a challenge, although in opposite ways.

- 4-Vinylanisole (**S10a**) was fully consumed when either of the catalysts (**4b**, **4c**, **4d**, **4e**) were used, forming complex mixtures from which the corresponding 1,3-dioxane was obtained only in moderate yields (e.g., with catalyst **4b**, in CyH, 0.1 M for 24 h: full conversion, 22% yield, 57:43 er).
- 4-Trifluoromethylstyrene (**S10c**) and ethyl 4-vinylbenzoate (**S10d**) require the more acidic catalyst **4b'** (2.5 mol%) to show some conversion. After 5 days at rt (in CyH, 0.2 M), these olefins generate the corresponding 1,3-dioxanes in low yields, but with excellent enantioselectivity: 10% yield (97:3 er) and 23% yield (96:4 er), respectively.
- 2-Methylstyrene (**S10f**) reacts with paraformaldehyde in the presence of 2.5 mol% of catalyst **4d** (in CyH, 0.1 M), to produce 78% of the corresponding 1,3-dioxane after 7 days, but with moderate enantioselectivity (88.5:11.5 er).

α-/β-Substituted Styrenes

- α-Methylstyrene (**S10b**) proved to be highly reactive with all the tested *i*IDP catalysts (2.5 mol%), giving always high conversion (>90%) after 24–48 h (in CyH, 0.1 M, at rt); however,

complex mixtures were obtained and the corresponding 1,3-dioxane could not be detected by ^1H NMR. Neither lower catalyst loadings (1 mol%) nor lower temperatures (0 °C) were helpful: the reaction showed then lower conversion, but still forming complex mixtures.

- *trans*-β-Methylstyrene (**S10e**) proved not reactive with the standard *i*IDP catalysts (no 1,3-dioxane detected by ^1H NMR after 5 days at rt). The more acidic *i*IDP **4b'** as catalyst provided 25% of *trans*-5-methyl-4-phenyl-1,3-dioxane, although with low enantioselectivity (er = 57.5:42.5).
- In an attempt to improve the reactivity of internal olefins, *trans*-anethole (**S10g**) was tested as substrate. Gratifyingly, utilizing catalyst **4b** resulted in 48% yield of the corresponding *trans*-1,3-dioxane after 4 days at rt (CyH, 0.2 M), although only with modest enantioselectivity (er = 64.5:35.5).
- Interestingly, the *i*IDP-catalyzed reactions with the internal olefins (**S10e** or **S10g**) displayed a high degree of diastereospecificity (*trans*-olefins produce *trans*-1,3-dioxanes). This goes in line with the outcome from the experiments using styrene-β-*d* (section 6.2 of this Supponrting Information file).

<u>**ALKYL OLEFINS**</u>

Despite all the efforts devoted to obtain reactivity from alkyl olefins, such as 1-octene (**S10h**), none of the tested catalysts provided any substrate conversion, neither at rt nor at higher temperatures (50 °C or 80 °C). Considering that aromatic rings could act as a recognition element for the *i*IDP catalysts, 4-phenyl-1-butene (**S10i**) was tested as substrate, but unfortunately no reactivity was observed.

(*R*)-4-(4-methoxyphenyl)-1,3-dioxane (**S11a**)

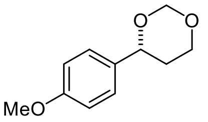

Prepared following *General Procedure C*, from 4-vinylanisole (**S10a**, 33 µL, 0.25 mmol), paraformaldehyde **2a** (18.8 mg, 0.625 mmol, 2.5 equiv.), with catalyst (*S*,*S*)-**4b** (8.6 mg, 62.5 µmol, 2.5 mol%), in dry cyclohexane (2.5 mL, 0.1 M) for 72 h. Purification by flash column chromatography (eluent: *n*-pentane/MTBE 100:0 → 80:20) to give **S11a** as a white solid (10.7 mg, 22%, er = 57:43).

^1H NMR (501 MHz, $CDCl_3$) δ 7.30 (d, *J* = 8.6 Hz, 2H), 6.90 (d, *J* = 8.8 Hz, 2H), 5.20 (d, *J* = 6.3 Hz, 1H), 4.89 (d, *J* = 6.3 Hz, 1H), 4.59 (dd, *J* = 11.2, 2.5 Hz, 1H), 4.20 (ddt, *J* = 11.5, 4.9, 1.4 Hz, 1H), 3.87 (td, *J* = 11.8, 2.5 Hz, 1H), 3.81 (s, 3H), 2.11 (dddd, *J* = 13.5, 12.3, 11.3, 4.9 Hz, 1H), 1.71 – 1.66 (m, 1H).

^{13}C NMR (126 MHz, $CDCl_3$) δ 159.4 (C), 133.8 (C), 127.3 (CH), 114.0 (CH), 94.4 (CH_2), 78.6 (CH), 67.1 (CH_2), 55.4 (CH_3), 34.0 (CH_2). Spectroscopic data was consistent with the values reported in the literature.[19]

ESI-HRMS: calculated for $C_{11}H_{14}O_3Na^+$ ($[M+Na]^+$): 217.0835, found: 217.0836.

HPLC (Chiralpak AD-3 column, Heptane/*i*-PrOH 97:3, 0.5 mL/min, 25 °C, 220 nm): t_R = 11.2 min (major), 13.2 min (minor).

<u>Racemate synthesis:</u> Following *General Procedure D* (condition A).

(*R*)-4-(4-(trifluoromethyl)phenyl)-1,3-dioxane (**S11c**)

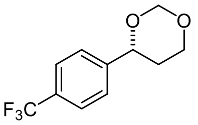

Prepared following *General Procedure C*, from 4-(trifluoromethyl)styrene (**S10c**, 37 µL, 0.25 mmol), paraformaldehyde **2a** (18.8 mg, 0.625 mmol, 2.5 equiv.), with catalyst (*S*,*S*)-**4b'** (12.9 mg, 62.5 µmol, 2.5 mol%), in dry cyclohexane (1.25 mL, 0.2 M) for 5 days. Purification by flash column chromatography (eluent: *n*-pentane/MTBE 100:0 → 95:5) to give **S11c** as a white solid (5.8 mg, 10%, er = 97:3).

^{1}H NMR (501 MHz, $CDCl_3$) δ 7.62 (d, *J* = 8.1 Hz, 2H), 7.49 (d, *J* = 8.1 Hz, 2H), 5.24 (d, *J* = 6.4 Hz, 1H), 4.90 (d, *J* = 6.4 Hz, 1H), 4.72 (dd, *J* = 11.3, 2.6 Hz, 1H), 4.25 – 4.18 (m, 1H), 3.89 (td, *J* = 11.9, 2.5 Hz, 1H), 2.04 (dddd, *J* = 13.5, 12.2, 11.3, 4.9 Hz, 1H), 1.78 – 1.73 (m, 1H).
^{19}F NMR (471 MHz, $CDCl_3$) δ –62.54 (s, 3F).
^{13}C NMR (126 MHz, $CDCl_3$) δ 145.6 (C), 130.1 (q, *J* = 32.5 Hz, C), 126.1 (CH), 125.6 (q, *J* = 3.8 Hz, CH), 124.2 (q, *J* = 271.8 Hz, C), 94.2 (CH_2), 78.0 (CH), 66.9 (CH_2), 34.1 (CH_2).
ESI-HRMS: calculated for $C_{11}H_{11}F_3O_2Na^+$ ($[M+Na]^+$): 255.0603, found: 255.0602.
$[\alpha_D^{25}]$ = +40.2 (*c* = 0.249, $CHCl_3$).
HPLC (Kromasil Amycoat RP column, CH_3CN/H_2O 50:50, 1 mL/min, 25 °C, 220 nm): t_R = 7.7 min (major), 8.5 min (minor).
Racemate synthesis: Following *General Procedure D* (condition C).

ethyl (*R*)-4-(1,3-dioxan-4-yl)benzoate (**S11d**)

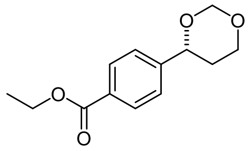

Prepared following *General Procedure C*, from ethyl 4-vinylbenzoate (**S10d**, 43 µL, 0.25 mmol), paraformaldehyde **2a** (18.8 mg, 0.625 mmol, 2.5 equiv.), with catalyst (*S*,*S*)-**4b'** (12.9 mg, 62.5 µmol, 2.5 mol%), in dry cyclohexane (1.25 mL, 0.2 M) for 5 days. Purification by flash column chromatography (eluent: *n*-pentane/MTBE 100:0 → 85:15) to give **S11d** as a white solid (13.4 mg, 23%, er = 96:4).
^{1}H NMR (501 MHz, $CDCl_3$) δ 8.04 (d, *J* = 8.4 Hz, 2H), 7.44 (d, *J* = 8.2 Hz, 2H), 5.24 (d, *J* = 6.4 Hz, 1H), 4.90 (d, *J* = 6.4 Hz, 1H), 4.71 (dd, *J* = 11.3, 2.6 Hz, 1H), 4.38 (q, *J* = 7.1 Hz, 2H), 4.21 (dd, *J* = 11.5, 4.8 Hz, 1H), 3.89 (td, *J* = 11.9, 2.5 Hz, 1H), 2.04 (dddd, *J* = 13.4, 12.2, 11.3, 4.8 Hz, 1H), 1.78 – 1.72 (m, 1H), 1.39 (t, *J* = 7.1 Hz, 3H).
^{13}C NMR (126 MHz, $CDCl_3$) δ 166.5 (C), 146.5 (C), 130.0 (C), 129.9 (CH), 125.6 (CH), 94.2 (CH_2), 78.3 (CH), 66.9 (CH_2), 61.1 (CH_2), 34.1 (CH_2), 14.5 (CH_3).
ESI-HRMS: calculated for $C_{13}H_{16}O_4Na^+$ ($[M+Na]^+$): 259.0941, found: 259.0944.
$[\alpha_D^{25}]$ = +28.5 (*c* = 0.548, $CHCl_3$).
HPLC (Chiralpak AD-3 column, Heptane/*i*-PrOH 95:5, 1 mL/min, 25 °C, 220 nm): t_R = 7.1 min (major), 8.4 min (minor).
Racemate synthesis: Following *General Procedure D* (condition C).

(*R*)-4-(*o*-tolyl)-1,3-dioxane (**S11f**)

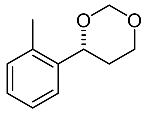

Prepared following *General Procedure C*, from 2-methylstyrene (**S10f**, 32 µL, 0.25 mmol), paraformaldehyde **2a** (18.8 mg, 0.625 mmol, 2.5 equiv.), with catalyst (*S*,*S*)-**4d** (11.2 mg, 62.5 µmol, 2.5 mol%), in dry cyclohexane (2.5 mL, 0.1 M) for 7 days. Purification by flash column chromatography (eluent: *n*-pentane/MTBE 100:0 → 95:5) to give **S11f** as a colorless liquid (23.1 mg, 78%, er = 88.5:11.5).
^{1}H NMR (501 MHz, $CDCl_3$) δ 7.50 (dd, *J* = 7.6, 1.7 Hz, 1H), 7.25 (t, *J* = 8.4 Hz, 1H), 7.20 (td, *J* = 7.4, 1.5 Hz, 1H), 7.15 (d, *J* = 7.4 Hz, 1H), 5.24 (d, *J* = 6.3 Hz, 1H), 4.93 (d, *J* = 6.3 Hz, 1H), 4.83 (dd, *J* = 11.2, 2.4 Hz, 1H), 4.22 (dd, *J* = 11.4, 4.8 Hz, 1H), 3.89 (td, *J* = 11.9, 2.4 Hz, 1H), 2.35 (s, 3H), 2.09 (dddd, *J* = 13.6, 12.3, 11.2, 4.8 Hz, 1H), 1.73 – 1.66 (m, 1H).
^{13}C NMR (126 MHz, $CDCl_3$) δ 139.5 (C), 134.4 (C), 130.5 (CH), 127.7 (CH), 126.5 (CH), 125.9 (CH), 94.5 (CH_2), 76.2 (CH), 67.2 (CH_2), 32.8 (CH_2), 19.1 (CH_3).
ESI-HRMS: calculated for $C_{11}H_{14}O_2Na^+$ ($[M+Na]^+$): 201.0886, found: 201.0888.
$[\alpha_D^{25}]$ = +37.6 (*c* = 0.622, $CHCl_3$).
HPLC (Chiralpak IC-3 column, Heptane/*i*-PrOH 97:3, 0.5 mL/min, 25 °C, 220 nm): t_R = 9.1 min (minor), 10.1 min (major).
Racemate synthesis: Following *General Procedure D* (condition B).

6. Mechanistic Studies

The acid-catalyzed intermolecular Prins reaction (olefins + formaldehyde) is thought to proceed via a stepwise mechanism (as depicted in Scheme S-1), starting with a nucleophilic attack of the olefin to an activated molecule of formaldehyde **I-1** to form an intermediate γ-hydroxybenzyl cation **I-2**. The fate of this species explains the several products commonly obtained (unsaturated alcohols, 1,3-glycols and esters thereof, and 1,3-dioxanes, among others). For example, the formation of the 1,3-dioxane product **3** can be explained if the intermediate **I-2** further reacts with a second molecule of formaldehyde, forming a cationic hemiformal **I-3** that readily undergoes cyclization.

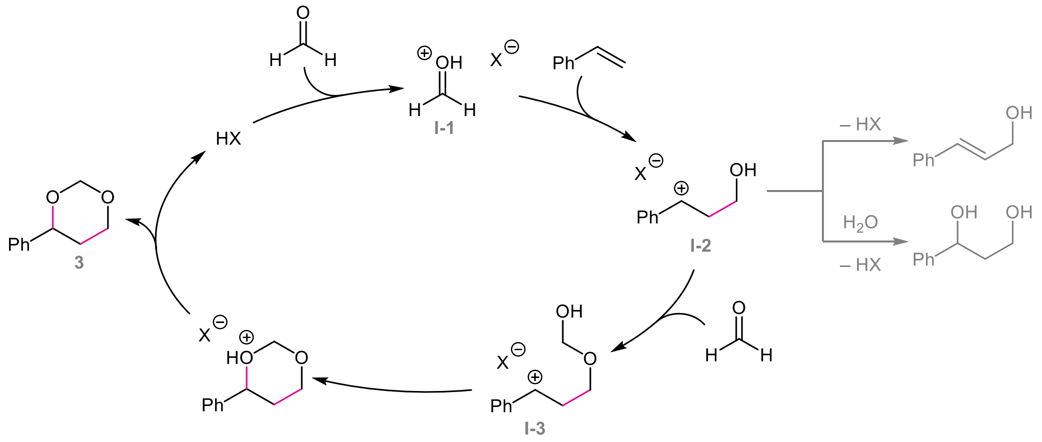

Scheme S-1. Proposed mechanistic cycle for an acid-catalyzed intermolecular Prins reaction via a stepwise pathway.

Now, considering that our *i*IDP-catalyzed Prins reaction proceeds with paraformaldehyde as HCHO source, the polymeric nature of this reagent should be taken into account when proposing a reaction mechanism. In order to do so, we wanted to gain a better understanding on how the two formaldehyde units get bonded to the olefin moiety.

The two HCHO units could come:

- either from different polymeric $(CH_2O)_n$ chains, or
- from the same polymeric $(CH_2O)_n$ chain:
    - if so: do the two HCHO units react with the olefin at the same time or in different steps?

By addressing these issues, we could know if the operating mechanistic pathway is stepwise or rather a (pseudo)-concerted one (see Figure 3A, in the main text).

6.1. Deuterium incorporation: (HCHO)ₙ and (DCDO)ₙ

To determine if the two formaldehyde units that are bonded to the olefin moiety during the Prins reaction could come from the same polymeric chain of paraformaldehyde, an experiment was designed, where styrene **1a** reacts with mixtures of "protio"-paraformaldehyde ($(HCHO)_n$, **2a**) and deutero-paraformaldehyde ($(DCDO)_n$, **2a'**), both commercially available.
Two possible scenarios were predicted:

- If the two formaldehyde units are bonded to the olefin in different steps, a statistical mixture of 1,3-dioxane products should be obtained, where the amount of ^1H (and also of ^2H) on positions C-2 and C-6 would be different from each other.
- Contrarily, if both formaldehyde units were to come from the same paraformaldehyde chain, every produced molecule of 1,3-dioxane should contain the same isotope (H or D) on both positions C-2 and C-6. This would be reflected in a C-2/C-6 ^1H content ratio close to 1.

The relative content of ^{1}H of positions C-2 and C-6 can be determined using ^{1}H NMR, since the integral values are proportional to the amount of ^{1}H atoms.

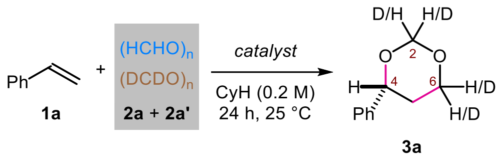

An oven-dried glass vial was charged with a mixture of paraformaldehyde **2a** and paraformaldehyde-*d*$_2$ **2a'** (3 equiv. in total, see below), catalyst (either 2.5 mol% of (*S*,*S*)-*i*IDP catalyst **4b**, or 15 mol% *p*-TsOH·H_2O), dry cyclohexane (0.125 mL, 0.2 M), and styrene **1a** (3 µL, 0.025 mmol, 1 equiv.), under Ar. Then, the mixture was vigorously stirred at rt for 24 h. The reaction was stopped by adding one drop of Et_3N, and the mixture was diluted with $CDCl_3$ and analyzed by ^{1}H NMR**.**
The following experiments were performed:

- Only $(HCHO)_n$: 3 equiv. **2a** (2.25 mg, 75 µmol)
- $(HCHO)_n/(DCDO)_n$: 2:1: 2 equiv. **2a** (1.50 mg, 50 µmol) + 1 equiv. **2a'** (0.80 mg, 25 µmol)
- $(HCHO)_n/(DCDO)_n$: 1:1: 1.5 equiv. **2a** (1.50 mg, 37.5 µmol) + 1.5 equiv. **2a'** (0.80 mg, 37.5 µmol)
- $(HCHO)_n$ / $(DCDO)_n$: 1:2: 2 equiv. **2a** (1.50 mg, 25 µmol) + 1 equiv. **2a'** (0.80 mg, 50 µmol)
- Only $(DCDO)_n$: 3 equiv. **2a'** (2.40 mg, 75 µmol)

For the ^{1}H NMR analysis, the signal corresponding to the hydrogen atom of the product on C-4 (4.65 ppm) is used in all cases as integration reference (integral value of 1.00), since that atom is not expected to be replaced by deuterium during the reaction.

*Table S-3. ^{1}H NMR analysis of the Prins reaction of styrene **1a** with mixtures of paraformaldehyde (**2a**) and paraformaldehyde-d$_2$ (**2a'**).*

Catalyst	2a/2a'	^{1}H NMR Integrals							Ratio H-2/H-6
		H-4	H-2a	H-2b	H-6a	H-6b	H-2: H-2a + H-2b	H-6: H-6a + H-6b	
*i*IDP **4b**	Only **2a**	1.00	0.96	1.11	1.02	1.03	2.07	2.05	1.01
	2:1	1.00	0.80	0.90	0.84	0.83	1.70	1.67	**1.02**
	1:1	1.00	0.70	0.80	0.73	0.73	1.49	1.47	**1.02**
	1:2	1.00	0.44	0.54	0.54	0.54	0.97	1.08	**0.90**
	Only **2a'**	1.00	0.00	0.00	0.00	0.00	0.00	0.00	n/a
p-TsOH	Only **2a**	1.00	0.98	1.08	0.98	0.99	2.06	1.97	1.05
	2:1	1.00	0.61	0.65	0.98	1.00	1.26	1.98	**0.64**
	1:1	1.00	0.44	0.47	0.96	0.99	0.91	1.95	**0.47**
	1:2	1.00	0.31	0.34	0.70	0.71	0.65	1.41	**0.46**
	Only **2a'**	1.00	0.00	0.00	0.00	0.00	0.00	0.00	n/a

As can be seen in Table S-3, the experiments with *i*IDP **4b** as catalyst led to a 1,3-dioxane product with very similar content of ^{1}H on positions C-2 and C-6, regardless of the **2a**/**2a'** ratio. This points towards a (pseudo)-concerted reaction pathway, where both formaldehyde units come from the same paraformaldehyde chain.
Conversely, the use of *p*-TsOH as catalyst resulted in the formation of a product with different ^{1}H contents on positions C-2 and C-6, which could be understood as the reaction proceeding rather via a stepwise pathway. Also, when **2a**/**2a'** mixtures were used, the lower ^{1}H incorporation on position C-2

in comparison to C-6 might indicate a stronger kinetic isotope effect for the acetalization step than for the olefin-aldehyde C–C bond-forming step.

6.2. Stereospecificity: experiments with β-deutero-styrenes

Two extreme scenarios can be considered for the mechanism of the *i*IDP-catalyzed Prins reaction: either a stepwise reaction pathway (involving the intermediacy of a benzyl cation), or a (pseudo)-concerted pathway. The previously shown experiments with $(HCHO)_n/(DCDO)_n$ mixtures (see Section 6.1 of this Supporting Information file) point towards the latter case being the operative pathway.

We considered that this could be further confirmed by studying the reaction with β-deuterostyrenes (both *trans*- and *cis*- isomers: **1q** and **1r**, respectively) as substrates. Similarly to the $(HCHO)_n/(DCDO)_n$ experiment, either *i*IDP **4b** or *p*-TsOH were used as catalysts.

For the *i*IDP-catalyzed reactions, the stereochemistry of the starting olefin was translated to the 1,3-dioxane product, as could be seen on the ^{1}H NMR spectra (H-4 and H-5, see Scheme S-2): the *cis*-olefin was transformed solely to a *cis*-product; the *trans*-olefin resulted in the exclusive formation of a *trans*-product.

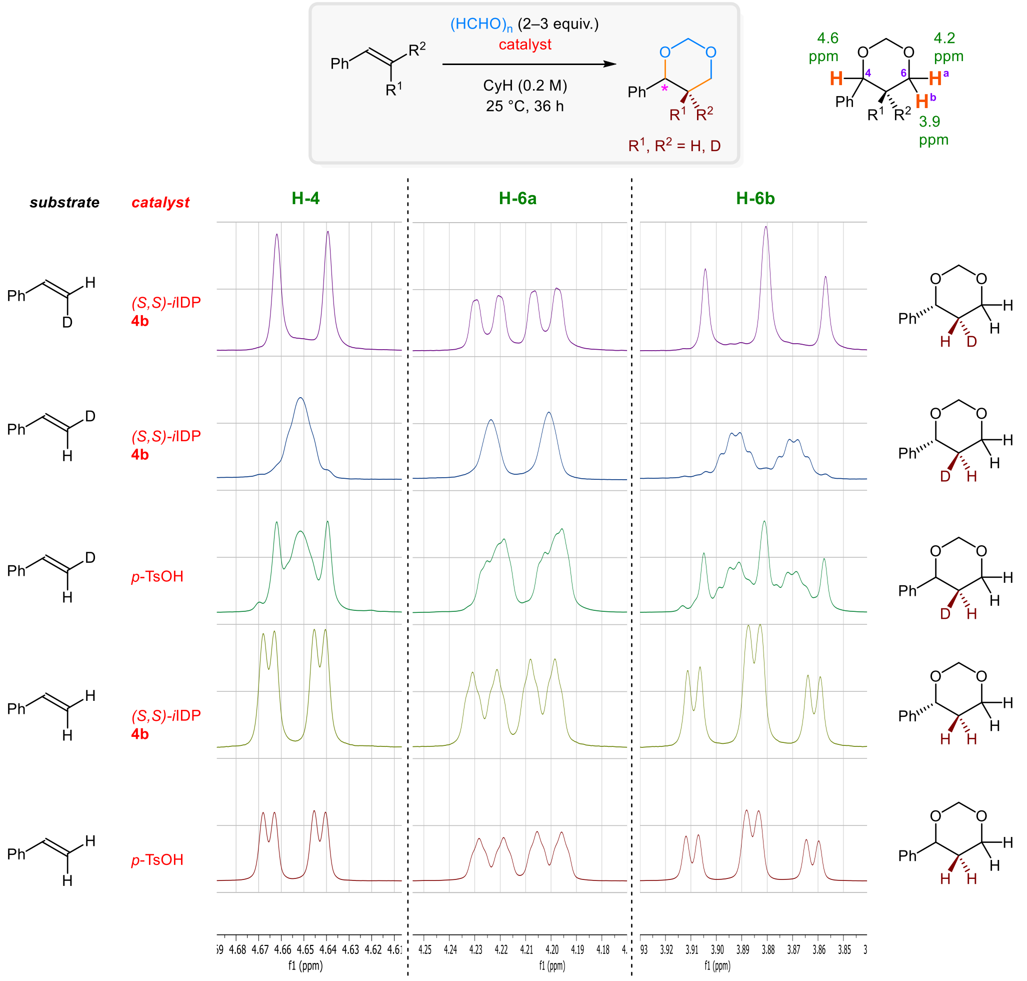

*Scheme S-2. ^{1}H NMR analysis of the Prins reaction of β-deutero-styrenes (**1w**, **1x**) with paraformaldehyde (**2a**).*

However, when the reaction was catalyzed with *p*-TsOH, the obtained product corresponded rather to an equimolar mixture of *cis*- and *trans*-1,3-dioxanes. These differences in the outcome of the reaction are in concordance with the previous mechanistic studies, underlining the different operating pathways depending on the type of catalyst used.

The results from the reaction with β-deutero-styrenes can be understood like this:

- When *p*-TsOH is used as catalyst, the reaction proceeds via a benzyl cation intermediate, which can rotate around the C–C bond, giving rise to the mixture of *cis*- and *trans*-products, regardless of the stereochemistry from the starting olefin.
- The *i*IDP-catalyzed reaction is probably proceeding rather via a concerted pathway, which makes the stereoinformation of the starting olefin to be translated into the formed 1,3-dioxane.

6.3. EWG on the catalyst structure: higher reactivity

To better understand the effect of the electron-withdrawing groups introduced in the catalyst structure (from **4a** to **4b**, and then to **4b’**), we conducted a ^{1}H NMR monitoring of the Prins reaction of styrene (**1a**) with paraformaldehyde (**2a**) toward 4-phenyl-1,3-dioxane (**3a**), using each of the mentioned *i*IDP catalysts (2.5 mol%), in $CDCl_3$ at rt, in the presence of Ph_3CH as internal standard for quantification.
As can be seen in Figure S-2, exchanging the 3,3’-substituent of the catalyst (R^W) from an alkyl-substituted to an EWG-substituted phenyl ring translates into a significant increase in reactivity. Further introduction of EWGs on the structure, now on the 6,6’-positions of the BINOL backbone (R^B), results in higher reactivity.

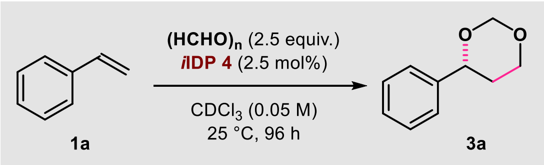

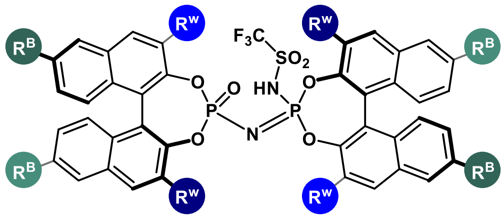

(*S*,*S*)-***i*IDP**

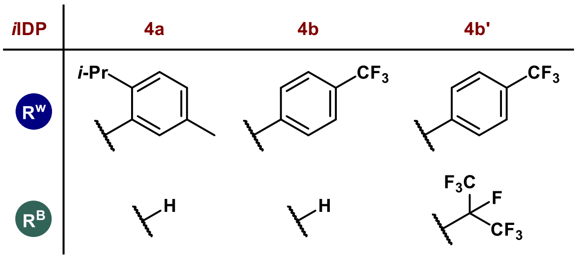

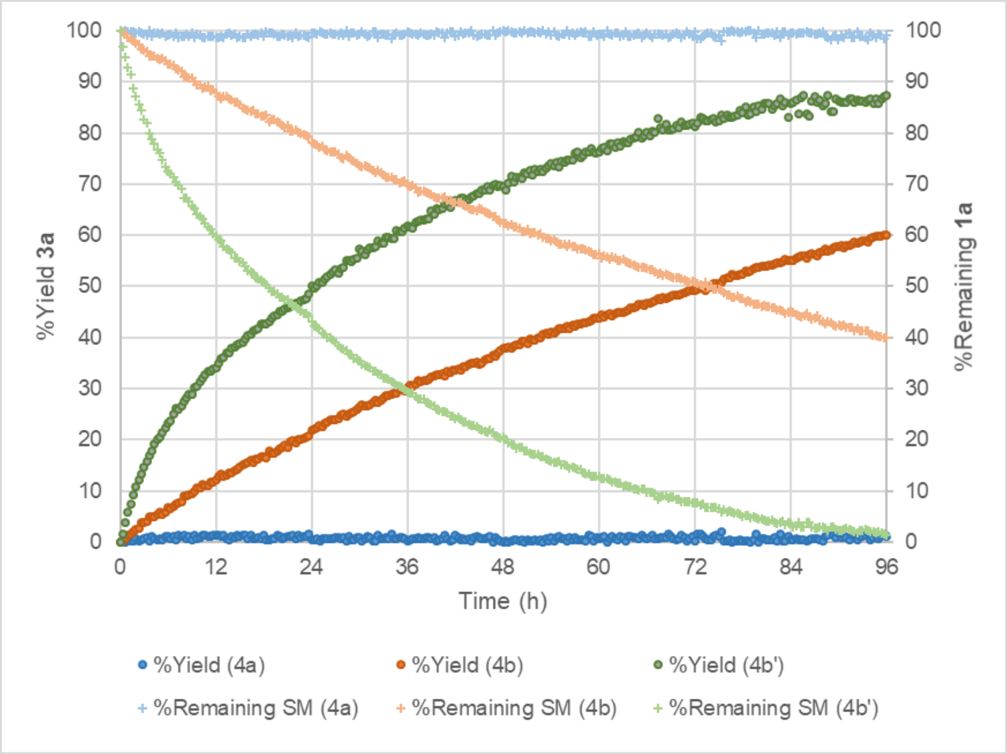

*Figure S-2. ^{1}H NMR monitoring for the model Prins reaction using catalysts **4a**, **4b** and **4b'** (in $CDCl_3$ at 25 °C).*

7. Synthesis of BINOL-derived imino-imidodiphosphates (*i*IDPs)

The synthesis of BINOL-derived imino-imidodiphosphates requires 3,3'-disubstituted BINOLs, which are prepared by means of a palladium-catalyzed Suzuki cross coupling reactions: the boronate resides on the BINOL moiety and the 3,3' substituents are inserted as (pseudo)halides, whose preparation is described below:

2-bromo-4-iodo-1-(trifluoromethyl)benzene (**S5**)

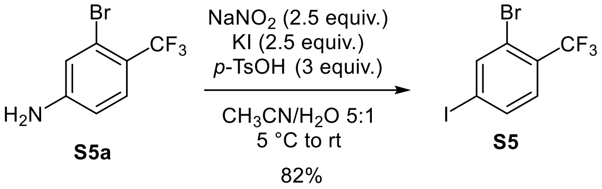

(Adapted from a reported procedure[20]) A RB flask under air was charged with 3-bromo-4-(trifluoromethyl)aniline **S5a** (3.0 g, 15 mmol, 1 equiv.), acetonitrile (65 mL) and *p*-toluenesulfonic acid monohydrate (7.13 g, 45 mmol, 3 equiv.), and the mixture was cooled to 0–5 °C (ice-water bath). A solution of sodium nitrite (1.72 g, 30 mmol, 2 equiv.) and potassium iodide (5.19 g, 37.5 mmol, 2.5 equiv.) in distilled water (12 mL) is added dropwise over 5 min. The mixture was allowed to reach rt and further stirred for 30 min, then poured onto distilled water (30 mL), neutralized with satd. aq. $NaHCO_3$ (20 mL) and extracted with MTBE (3 x 30 mL). The combined organic phases were washed with satd. aq. $NaHCO_3$ (1 x 20 mL), aq. $Na_2S_2O_3$ 10% (1 x 20 mL), water (1 x 20 mL) and brine (1 x 20 mL), successively; then dried over anhydrous Na_2SO_4, filtered and concentrated under reduced pressure. Purification by flash column chromatography on silica gel (*n*-pentane) afforded the corresponding aryl iodide **S5** as a colorless liquid (4.32 g, 82%).

^{1}H NMR (501 MHz, $CDCl_3$) δ 8.09 (d, *J* = 0.9 Hz, 1H), 7.76 (dd, *J* = 8.3, 0.9 Hz, 1H), 7.38 (d, *J* = 8.3 Hz, 2H).

^{19}F NMR (471 MHz, $CDCl_3$) δ –62.90 (s, 3F).

^{13}C NMR (126 MHz, $CDCl_3$) δ 143.2 (CH), 136.7 (CH), 130.0 (d, *J* = 31.9 Hz, C), 129.0 (q, *J* = 5.2 Hz, CH), 123.0 (q, *J* = 273.4 Hz, C), 121.0 (C), 98.9 (C). Spectroscopic data was consistent with the values reported in the literature.[21]

EI-HRMS: calculated for $C_7H_3BrF_3I^+$ ($[M]^+$): 349.8410, found: 349.8409.

6-(perfluoropropan-2-yl)naphthalen-2-yl trifluoromethanesulfonate (**S6**)

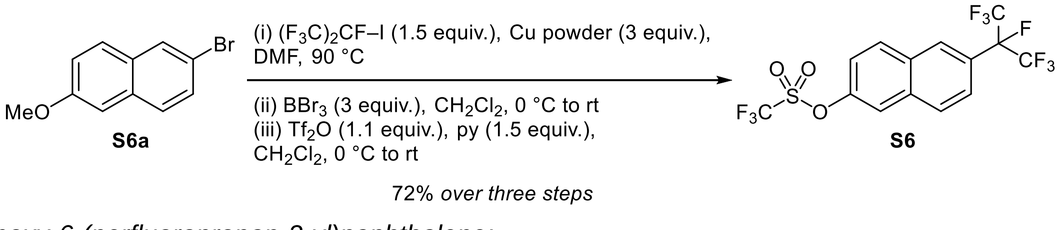

2-methoxy-6-(perfluoropropan-2-yl)naphthalene:

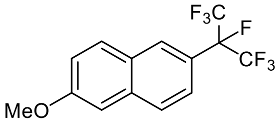

An oven-dried Schlenk flask under Ar atmosphere was charged with Cu powder (2.86 g, 45 mmol, 3 equiv.; freshly activated with aq. HCl 2 M, washed with ethanol and acetone, and dried under vacuum overnight), 6-bromo-2-methoxynaphthalene (**S6a**, 3.56 g, 15 mmol, 1 equiv.), and dry DMF (25 mL). After degassing the mixture by bubbling Ar for 5 min, perfluoroisopropyl iodide (3.2 mL, 22.5 mmol, 1.5 equiv.) was added in one portion and the flask was closed under Ar. The mixture was stirred vigorously at 90 °C (oil bath) for 24 h. After cooling to rt, the mixture was diluted with MTBE (100 mL), treated with water (1 mL dropwise, then 20 mL in one portion) and vigorously stirred for 10

min at rt. Afterwards, the mixture was passed through a Celite® pad, washing with MTBE. The aqueous layer was further extracted with MTBE (3 x 50 mL) and the combined organic layers were washed with aq. HCl 2 M (1 x 30 mL), water (1 x 100 mL) and brine (1 x 50 mL). After drying over anhydrous Na_2SO_4, the organic phase was filtered and concentrated under reduced pressure, affording a yellow liquid, which was used directly for the next reaction.

^{1}H NMR (501 MHz, $CDCl_3$): δ 8.04 (s, 1H), 7.83 (t, *J* = 9.4 Hz, 2H), 7.59 (d, *J* = 8.1 Hz, 1H), 7.24 (dd, *J* = 8.9, 2.6 Hz, 1H), 7.17 (d, *J* = 2.5 Hz, 1H), 3.95 (s, 3H).

^{19}F NMR (471 MHz, $CDCl_3$) δ –75.51 (d, *J* = 7.3 Hz, 6F), –181.74 (hept, *J* = 7.4 Hz, 1F).

6-(perfluoropropan-2-yl)naphthalen-2-ol:

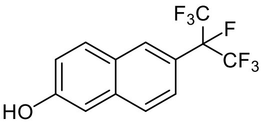

An oven-dried RB flask was charged with the product from the previous Ullmann coupling and dry CH_2Cl_2 (300 mL). This solution was cooled to –10 °C (salt/ice) and, using an addition funnel, BBr_3 (45 mL, 1 M in CH_2Cl_2, 45 mmol, 3 equiv.) was added dropwise over 10 min. The mixture was vigorously stirred at –10 °C for 30 min, and then at rt for 6 h. After cooling to 0 °C, the reaction was stopped by dropwise addition of MeOH (50 mL, with the addition funnel) and further diluted with distilled water (200 mL). The aqueous layer was extracted with CH_2Cl_2 (2 x 50 mL), and the combined organic phases were washed with brine (1 x 100 mL). After drying over anhydrous Na_2SO_4, the organic phase was filtered and concentrated under reduced pressure, affording an off-white solid, which was used directly for the next reaction.

^{1}H NMR (501 MHz, CD_2Cl_2) δ 8.18 (s, 1H), 8.01 (d, *J* = 8.9 Hz, 1H), 7.98 (d, *J* = 8.9 Hz, 1H), 7.69 (d, *J* = 8.8 Hz, 1H), 7.58 (d, *J* = 2.5 Hz, 1H), 7.34 (dd, *J* = 8.9, 2.4 Hz, 1H).

^{19}F NMR (471 MHz, CD_2Cl_2) δ –75.76 (d, *J* = 6.8 Hz, 6F), –182.06 (hept, *J* = 7.1 Hz, 1F).

*6-(perfluoropropan-2-yl)naphthalen-2-yl trifluoromethanesulfonate (**S6**):*

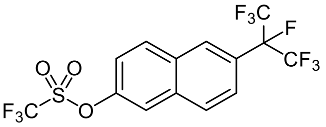

An oven-dried RB flask was charged with the product from the previous deprotection and dry CH_2Cl_2 (40 mL). This solution was cooled to 0 °C (ice/water) and treated with pyridine (1.8 mL, 22.5 mmol, 1.5 equiv.). Trifluoromethanesulfonic anhydride (2.8 mL, 16.5 mmol, 1.1 equiv.) was added dropwise at 0 °C and the mixture was stirred vigorously at rt overnight. The reaction was stopped by dropwise addition of aq. HCl 1 M (50 mL) and further diluted with MTBE (100 mL). The aqueous layer was extracted with MTBE (2 x 30 mL), and the combined organic phases were washed subsequently with satd. aq. $NaHCO_3$ (1 x 50 mL), water (1 x 30 mL), and brine (1 x 30 mL). After drying over anhydrous Na_2SO_4, the organic phase was filtered and concentrated under reduced pressure. Purification by flash column chromatography on silica gel (*n*-pentane) afforded the corresponding aryl triflate **S6** as a light yellow oil that solidified upon standing as white solid (4.78 g, 72% over three steps).

^{1}H NMR (501 MHz, CD_2Cl_2) δ 8.25 (s, 1H), 8.09 (d, *J* = 9.1 Hz, 1H), 8.07 (d, *J* = 8.9 Hz, 1H), 7.88 (d, *J* = 2.5 Hz, 1H), 7.77 (d, *J* = 8.8 Hz, 1H), 7.53 (dd, *J* = 9.0, 2.5 Hz, 1H).

^{19}F NMR (471 MHz, CD_2Cl_2) δ –73.10 (s, 3F), –75.73 (d, *J* = 7.4 Hz, 6F), –182.12 (hept, *J* = 7.2 Hz, 1F).

^{13}C NMR (126 MHz, CD_2Cl_2) δ 149.0, 134.7, 132.2, 132.0 (d, *J* = 2.3 Hz), 130.0 (d, *J* = 2.4 Hz), 127.0 (d, *J* = 12.2 Hz), 125.8 (d, *J* = 20.5 Hz), 123.7 (d, *J* = 9.7 Hz), 122.2 (d, *J* = 28.1 Hz), 121.6, 119.92 (d, *J* = 27.5 Hz), 119.63, 119.2 (d, *J* = 320.9 Hz).

EI-HRMS: calculated for $C_{14}H_6F_{10}O_3S^+$ ($[M]^+$): 443.9873, found: 443.9880.

4-(perfluoropropyl)phenyl trifluoromethanesulfonate (**S7**)

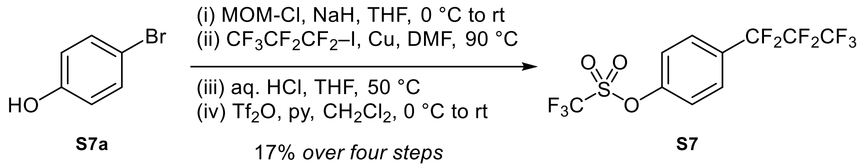

1-bromo-4-(methoxymethoxy)benzene:

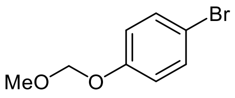

(Adapted from a reported procedure[22]) An oven-dried RB flask was charged with 4-bromophenol (**S7a**, 3.46 g, 20 mmol, 1 equiv.) and dry THF (65 mL). To the cooled mixture at 0 °C, NaH (960 mg of 60% suspension in oil, 24 mmol, 1.2 equiv.) was added portionwise and, after stirring 15 min, MOM-Cl (1.8 mL, 22 mmol, 1.1 equiv.) was added in one portion. The mixture was slowly warmed up to rt and further stirred for 24 h. The reaction was quenched by carefully adding satd. aq. NH_4Cl, diluted with 50 mL distilled water and extracted with MTBE (3 x 25 mL). Then, the combined organic phase was washed with brine (1 x 30 mL), dried over anhydrous Na_2SO_4, filtered and concentrated under reduced pressure. The obtained crude was directly used for the next step, without further purification.

^1H NMR (501 MHz, CD_2Cl_2) δ 7.39 (d, J = 9.0 Hz, 2H), 6.93 (d, J = 9.0 Hz, 2H), 5.14 (s, 2H), 3.44 (s, 3H).

^{13}C NMR (126 MHz, CD_2Cl_2) δ 156.9, 132.6, 118.6, 114.3, 95.0, 56.3.

Spectroscopic data was consistent with the values reported in the literature.[22]

1-(methoxymethoxy)-4-(perfluoropropyl)benzene:

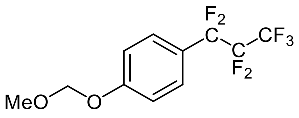

An oven-dried Schlenk flask under Ar atmosphere was charged with Cu powder (1.90 g, 30 mmol, 6 equiv.; freshly activated with aq. HCl 2 M, washed with ethanol and acetone, and dried under vacuum overnight), crude 1-bromo-4-(methoxymethoxy)benzene (1.08 g, 5 mmol, 1 equiv.), and dry DMF (10 mL). After degassing the mixture by bubbling Ar for 5 min, perfluoropropyl iodide (2.2 mL, 15 mmol, 3 equiv.) was added in one portion and the flask was closed under Ar. The mixture was stirred vigorously at 90 °C (oil bath) for 2.5 days. After cooling to rt, the mixture was diluted with MTBE (30 mL), treated with water (1 mL dropwise, then 5 mL in one portion) and vigorously stirred for 10 min at rt. Afterwards, the mixture was passed through a Celite® pad, washing with MTBE. The aqueous layer was further extracted with MTBE (3 x 30 mL) and the combined organic layers were washed with water (1 x 100 mL) and brine (1 x 50 mL). After drying over anhydrous Na_2SO_4, the organic phase was filtered and concentrated under reduced pressure, affording a light yellow liquid, which was used directly for the next reaction.

^1H NMR (501 MHz, CD_2Cl_2) δ 7.51 (d, J = 8.8 Hz, 2H), 7.15 (d, J = 8.9 Hz, 2H), 5.23 (s, 2H), 3.47 (s, 3H).

^{19}F NMR (471 MHz, CD_2Cl_2) δ –80.42 (t, J = 10.0 Hz, 3F), –110.96 (q, J = 10.2 Hz, 2F), –126.75 (s, 2F).

^{13}C NMR (126 MHz, CD_2Cl_2) δ 160.6 (C), 128.7 (t, J = 6.6 Hz, CH), 116.6 (CH), 94.7 (CH_2), 56.5 (CH_3).

4-(perfluoropropyl)phenol:

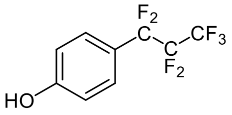

A RB flask was charged with the crude product from the previous Ullmann coupling (1.18 g, ~3.8 mmol), 1,4-dioxane (8 mL), MeOH (1 mL), and aq. HCl 6 M (3 mL, 18 mmol, ~5 equiv.). The mixture was heated to 90 °C overnight and, after cooling to rt, it was diluted with CH_2Cl_2 (50 mL) and distilled water (50 mL). The aqueous layer was further extracted with CH_2Cl_2 (3 x 20 mL) and the combined organic layers were washed with brine (1 x 30 mL). After drying over anhydrous Na_2SO_4, the organic phase was

filtered and concentrated under reduced pressure, affording an orange oil, which was used directly for the next reaction.
^{1}H NMR (501 MHz, CD_2Cl_2) δ 7.47 (d, *J* = 8.6 Hz, 2H), 6.96 (d, *J* = 8.8 Hz, 2H), 5.57 (s, 1H).
^{19}F NMR (471 MHz, CD_2Cl_2) δ –80.44 (t, *J* = 9.9 Hz, 3F), –110.85 (q, *J* = 9.9 Hz, 2F), –126.83 (s, 2F).
^{13}C NMR (126 MHz, CD_2Cl_2) δ 159.3 (C), 129.0 (t, *J* = 6.5 Hz, CH), 116.0 (CH).

*4-(perfluoropropyl)phenyl trifluoromethanesulfonate (**S7**):*

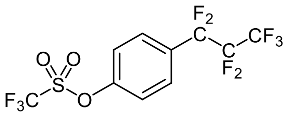

An oven-dried RB flask was charged with crude 4-(perfluoropropyl)phenol (856 mg, ~3.25 mmol, 1 equiv.) and dry CH_2Cl_2 (7 mL). This solution was cooled to 0 °C (ice/water) and treated with pyridine (0.36 mL, 4.25 mmol, 1.3 equiv.). Trifluoromethanesulfonic anhydride (0.60 mL, 3.6 mmol, 1.1 equiv.) was added dropwise at 0 °C and the mixture was stirred vigorously at rt overnight. The reaction was stopped by dropwise addition of aq. HCl 1 M (10 mL) and further diluted with MTBE (25 mL). The aqueous layer was extracted with MTBE (2 x 20 mL), and the combined organic phases were washed subsequently with satd. aq. $NaHCO_3$ (1 x 30 mL), water (1 x 30 mL), and brine (1 x 30 mL). After drying over anhydrous Na_2SO_4, the organic phase was filtered and concentrated under reduced pressure. Purification by flash column chromatography on silica gel (*n*-pentane) afforded the corresponding aryl triflate **S7** as a colorless liquid (442 mg, 72% over three steps).
^{1}H NMR (501 MHz, CD_2Cl_2) δ 7.74 (d, *J* = 8.8 Hz, 2H), 7.48 (d, *J* = 8.8 Hz, 2H).
^{19}F NMR (471 MHz, CD_2Cl_2) δ –73.06 (s, 3F), –80.35 (t, *J* = 10.0 Hz, 3F), –111.86 (q, *J* = 10.0 Hz, 2F), –126.53 (s, 2F).
^{13}C NMR (126 MHz, CD_2Cl_2) δ 152.4 (C), 129.7 (t, *J* = 6.5 Hz, CH), 122.5 (CH), 119.1 (q, *J* = 320.7 Hz, C).
APCI-HRMS: calculated for $C_{10}H_4F_{10}O_3S^+$ ([M]$^+$): 393.9716, found: 393.9716.

7.1. Synthesis of 3,3'-disubstituted BINOLs

General Procedure E: Suzuki Coupling and MOM Deprotection

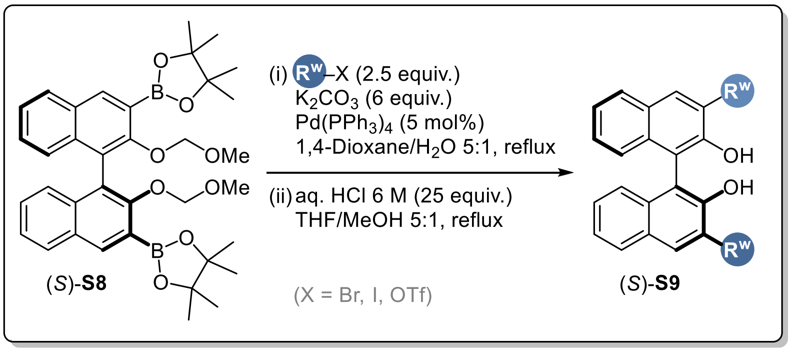

In a flask under Ar, (*S*)-2,2'-(2,2'-bis(methoxymethoxy)-[1,1'-binaphthalene]-3,3'-diyl)bis(4,4,5,5-tetramethyl-1,3,2-dioxaborolane) **S8**[23] (313 mg, 0.50 mmol, 1 equiv.), the corresponding aryl (pseudo)halide (2–3 equiv.), and solid K_2CO_3 (415 mg, 3 mmol, 6 equiv.) were dissolved in a 4:1 *v/v* mixture of 1,4-dioxane/water (5 mL). After degassing the mixture by bubbling Ar for 5 min, $Pd(PPh_3)_4$ (29 mg, 0.025 mmol, 5 mol%) was added and the resulting mixture was heated to reflux for 18–24 h. The reaction mixture was cooled to rt and filtered through a short pad of Celite®, washing with CH_2Cl_2 (30 mL). The filtrate was washed with water (1 x 20 mL) and brine (1 x 20 mL), then dried over anhydrous Na_2SO_4, filtered and concentrated under reduced pressure.
The obtained crude was redissolved in a mixture of THF/MeOH 3:1 *v/v* (10 mL), treated with aq. HCl 6 M (2 mL, 12 mmol, 24 equiv.) and heated to 50 °C overnight. After cooling to rt, the mixture was

diluted with CH_2Cl_2 (20 mL) and water (20 mL), and the aqueous phase was further extracted with CH_2Cl_2 (3 x 20 mL). The combined organic phases were washed with brine (1 x 30 mL) and, after drying over anhydrous Na_2SO_4, the extract was filtered and concentrated under reduced pressure. Purification by flash column chromatography on silica gel (*n*-pentane/CH_2Cl_2 or *n*-pentane/MTBE mixtures) afforded the corresponding 3,3'-disubstituted BINOL (*S*)-**S9**.

(*S*)-3,3'-bis(4-(trifluoromethyl)phenyl)-[1,1'-binaphthalene]-2,2'-diol (**S9a**)

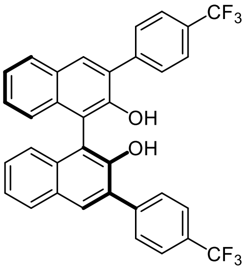

Following *General Procedure E*, employing (*S*)-**S8** (940 mg, 1.5 mmol) and 4-bromobenzotrifluoride (1012 mg, 4.5 mmol, 3 equiv.) as coupling partners. The crude product was purified by flash column chromatography (eluent: *n*-pentane/MTBE 100:0 → 90:10) to give **S9a** as an off-white solid (793 mg, 92%).

^{1}H NMR (501 MHz, $CDCl_3$) δ 8.06 (s, 2H), 7.96 (d, *J* = 8.3 Hz, 2H), 7.87 (d, *J* = 8.0 Hz, 4H), 7.74 (d, *J* = 7.9 Hz, 4H), 7.44 (ddd, *J* = 8.2, 6.8, 1.3 Hz, 2H), 7.37 (ddd, *J* = 8.2, 6.8, 1.3 Hz, 2H), 7.23 (d, *J* = 8.4 Hz, 2H), 5.31 (s, 2H).
^{19}F NMR (471 MHz, $CDCl_3$) δ –62.49 (s, 6F).
^{13}C NMR (126 MHz, $CDCl_3$) δ 150.2 (C), 141.3 (C), 133.2 (C), 132.2 (CH), 130.1 (CH), 125.4 (q, *J* = 32.2 Hz, C), 129.6 (C), 129.5 (C), 128.9 (CH), 128.2 (CH), 125.4 (q, *J* = 3.8 Hz, CH), 125.0 (CH), 124.2 (CH), 112.1 (C). Spectroscopic data was consistent with the values reported in the literature.[24]
$[\alpha_D^{25}]$ = –65.3 (*c* = 0.44, $CHCl_3$); literature (for (*R*)-enantiomer): $[\alpha_D^{23}]$ = +48.1 (*c* = 0.21, $CHCl_3$).[24]

(*S*)-3,3'-bis(3-bromo-4-(trifluoromethyl)phenyl)-[1,1'-binaphthalene]-2,2'-diol (**S9b**)

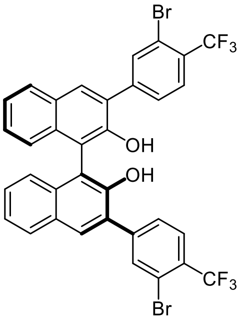

Following *General Procedure E*, employing (*S*)-**S8** (626 mg, 1 mmol) and 2-bromo-4-iodo-1-(trifluoromethyl)benzene (**S5**, 633 mg, 2.3 mmol, 2.3 equiv.) as coupling partners. The crude product was purified by flash column chromatography (eluent: *n*-pentane/CH_2Cl_2 100:0 → 70:30) to give **S9b** as a white foamy solid (467 mg, 64%).

^{1}H NMR (501 MHz, CD_2Cl_2) δ 8.16 (s, 2H), 8.10 (s, 2H), 8.00 (d, *J* = 7.3 Hz, 2H), 7.84 (d, *J* = 8.2 Hz, 2H), 7.81 (d, *J* = 8.4 Hz, 2H), 7.46 (ddd, *J* = 8.1, 6.8, 1.2 Hz, 2H), 7.39 (ddd, *J* = 8.2, 6.8, 1.3 Hz, 2H), 7.19 (d, *J* = 8.4 Hz, 2H), 5.44 (s, 2H).
^{19}F NMR (471 MHz, CD_2Cl_2) δ –62.59 (s, 6F).
^{13}C NMR (126 MHz, CD_2Cl_2) δ 150.5 (C), 143.4 (C), 136.3 (CH), 133.8 (C), 132.7 (CH), 129.9 (C), 129.2 (CH), 129.00 (C), 128.97 (CH), 128.7 (CH), 128.2 (C), 128.1 (q, *J* = 5.4 Hz, CH), 125.3 (CH), 124.4 (CH), 123.6 (d, *J* = 272.9 Hz, C), 120.0 (C), 112.4 (C).
ESI-HRMS: calculated for $C_{34}H_{17}Br_2F_6O_2^-$ ([M–H]$^-$): 728.9505, found: 728.9514.
$[\alpha_D^{25}]$ = –37.6 (*c* = 0.20, $CHCl_3$).

(*S*)-3,3'-bis(4-(perfluoropropyl)phenyl)-[1,1'-binaphthalene]-2,2'-diol (**S9c**)

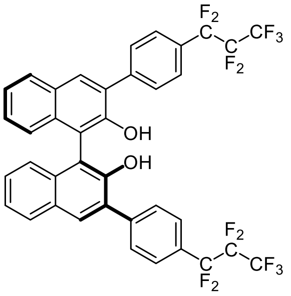

Following *General Procedure E*, employing (*S*)-**S8** (626 mg, 1 mmol) and 4-(perfluoropropyl)phenyl trifluoromethanesulfonate (**S7**, 633 mg, 2.3 mmol, 2.3 equiv.) as coupling partners. The crude product was purified by flash column chromatography (eluent: *n*-pentane/CH_2Cl_2 100:0 → 70:30) to give **S9c** as a white foamy solid (467 mg, 64%).

^{1}H NMR (501 MHz, CD_2Cl_2) δ 8.11 (s, 2H), 7.99 (d, *J* = 8.2 Hz, 2H), 7.93 (d, *J* = 8.5 Hz, 4H), 7.72 (d, *J* = 8.3 Hz, 4H), 7.45 (ddd, *J* = 8.0, 6.8, 1.2 Hz, 2H), 7.37 (ddd, *J* = 8.2, 6.8, 1.3 Hz, 2H), 7.21 (d, *J* = 8.5 Hz, 2H), 5.45 (s, 2H).
^{19}F NMR (471 MHz, CD_2Cl_2) δ –80.32 (t, *J* = 10.0 Hz, 6F), –111.71 (q, *J* = 9.8 Hz, 4F), –126.50 (s, 4F).
^{13}C NMR (126 MHz, CD_2Cl_2) δ 150.6, 142.1, 133.7, 132.6, 130.4, 130.0, 129.7, 129.1, 128.3, 128.0, 127.1 (t, *J* = 6.4 Hz), 125.1, 124.4, 112.5.
ESI-HRMS: calculated for $C_{38}H_{19}F_{14}O_2^-$ ([M–H]$^-$): 773.1167, found: 773.1172.
$[\alpha_D^{25}]$ = –37.1 (*c* = 0.18, $CHCl_3$).

(*S*)-6,6'''-bis(perfluoropropan-2-yl)-[2,2':4',1'':3'',2'''-quaternaphthalene]-2'',3'-diol (**S9d**)

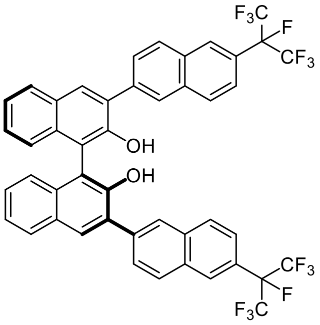

Following *General Procedure E*, employing (*S*)-**S8** (313 mg, 0.5 mmol) and 6-(perfluoropropan-2-yl)naphthalen-2-yl trifluoromethanesulfonate (**S6**, 489 mg, 1.1 mmol, 2.2 equiv.) as coupling partners. The crude product was purified by flash column chromatography (eluent: *n*-pentane/MTBE 100:0 → 90:10) to give **S9d** as a light yellow solid (410 mg, 94%).

^{1}H NMR (501 MHz, CD_2Cl_2) δ 8.31 (s, 2H), 8.24 (s, 2H), 8.19 (s, 2H), 7.71 (d, *J* = 8.8 Hz, 2H), 7.46 (ddd, *J* = 8.1, 6.8, 1.2 Hz, 2H), 7.39 (ddd, *J* = 8.3, 6.8, 1.4 Hz, 2H), 7.28 (dd, *J* = 8.4, 1.1 Hz, 2H), 5.53 (s, 2H).
^{19}F NMR (471 MHz, CD_2Cl_2) δ –75.72 (d, *J* = 7.3 Hz), –181.95 (hept, *J* = 7.5 Hz).
^{13}C NMR (126 MHz, CD_2Cl_2) δ 150.9, 138.0, 134.6, 133.7, 132.5 (CH), 132.2 (d, *J* = 2.1 Hz), 130.5, 130.1, 129.68 (CH), 129.66 (CH), 129.1 (CH), 129.0 (CH), 128.6 (CH), 128.1 (CH), 126.7 (d, *J* = 11.8 Hz, CH), 125.0 (CH), 124.5 (CH), 124.4, 122.5, 122.3 (q, *J* = 9.4 Hz, CH), 120.1 (d, *J* = 28.6 Hz), 112.8.
ESI-HRMS: calculated for $C_{46}H_{23}O_2F_{14}^-$ ([M–H]$^-$): 873.1480, found: 873.1480.
$[\alpha_D^{25}]$ = +36.3 (*c* = 0.25, $CHCl_3$).

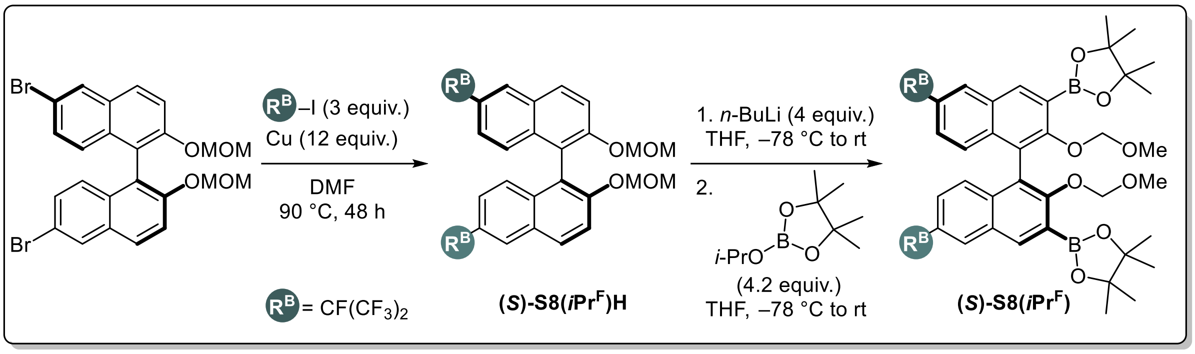

(*S*)-2,2'-bis(methoxymethoxy)-6,6'-bis(perfluoropropan-2-yl)-1,1'-binaphthalene (S8(*i*Pr^F)H)

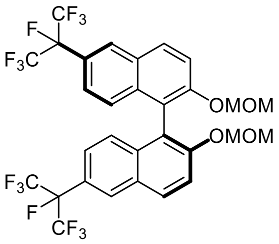

<u>*Activation of copper:*</u> Copper powder (5 g) was stirred for 30 min in aq. HCl 1 M (100 mL), and then filtered with a Büchner funnel, washing with distilled water (2 x 100 mL), ethanol (1 x 50 mL), acetone (1 x 50 mL) and Et_2O (1 x 50 mL). The obtained solid is transferred to a flask and dried under vacuum (10^{-3} mbar) overnight.

<u>*Ullmann coupling:*</u> An oven-dried Schlenk flask, equipped with a magnetic stir bar, was charged under Ar with freshly activated copper powder (1.70 g, 26.7 mmol, 6 equiv.), DMF (10 mL), (*S*)-6,6'-dibromo-2,2'-bis(methoxymethoxy)-1,1'-binaphthalene[25] (1.19 g, 2.23 mmol), and heptafluoro-2-iodopropane (0.95 mL, 6.68 mmol, 3 equiv.). The mixture was degassed by bubbling Ar for 10 min, then the flask was closed and the mixture was heated under stirring at 90 °C for 48 h. After allowing to cool to rt and checking full conversion (TLC monitoring), the mixture was filtered through a pad of Celite®, washing with MTBE (150 mL). The filtrate was washed with distilled water (2 x 100 mL) and brine (1 x 50 mL), then dried over anhydrous Na_2SO_4, filtered and concentrated under reduced pressure. Purification by flash column chromatography on silica gel (*n*-pentane/MTBE 90:10 *v/v*) afforded the corresponding product **S8(*i*Pr^F)H** as a yellow solid (1.10 g, 70% yield).

^{1}H NMR (501 MHz, CD_2Cl_2) δ 8.21 (s, 2H), 8.10 (d, *J* = 9.1 Hz, 2H), 7.73 (d, *J* = 9.1 Hz, 2H), 7.40 (d, *J* = 9.1 Hz, 2H), 7.25 (d, *J* = 9.1 Hz, 2H), 5.14 (d, *J* = 6.9 Hz, 2H), 5.09 (d, *J* = 7.0 Hz, 2H), 3.17 (s, 6H).
^{19}F NMR (471 MHz, CD_2Cl_2) δ –75.84 (d, *J* = 7.0 Hz, 12F), –182.07 (hept, *J* = 7.8, 7.2 Hz, 2F).
^{13}C NMR (126 MHz, CD_2Cl_2) δ 154.8 (C), 135.1 (C), 131.0 (CH), 129.12 (C), 129.11 (C), 127.0 (d, *J* = 11.8 Hz, CH), 126.6 (d, *J* = 2.3 Hz, CH), 122.5 (d, *J* = 9.8 Hz, CH), 122.3 (d, *J* = 20.6 Hz, C), 120.2 (C), 119.9 (C), 118.2 (CH), 95.3 (CH_2), 56.2 (CH_3).
ESI-HRMS: calculated for $C_{30}H_{20}F_{14}O_4Na^+$ ($[M+Na]^+$): 733.1030, found: 733.1034.
$[\alpha_D^{25}]$ = –41.2 (*c* = 0.61, CH_2Cl_2).

(*S*)-2,2'-(2,2'-bis(methoxymethoxy)-6,6'-bis(perfluoropropan-2-yl)-[1,1'-binaphthalene]-3,3'-diyl)bis(4,4,5,5-tetramethyl-1,3,2-dioxaborolane) (S8(*i*Pr^F))

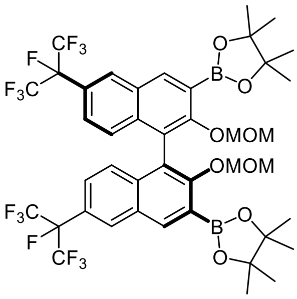

An oven-dried RB flask, equipped with a magnetic stir bar, was charged under Ar with **S8(*i*Pr^F)H** (1.00 g, 1.41 mmol) and THF (15 mL). After cooling the mixture at –78 °C, *n*-BuLi (2.5 M in hexanes, 2.3 mL, 5.8 mmol, 4 equiv.) was added dropwise over 5 min. After the addition was completed, the mixture was stirred for 30 min at –78 °C and then for further 3 h at rt. The mixture was cooled again to –78 °C and 2-isopropoxy-4,4,5,5-tetramethyl-1,3,2-dioxaborolane (1.2 mL, 5.91 mmol, 4.2 equiv.) was added dropwise over 2 min, followed by stirring at rt for 16 h (overnight). The reaction was quenched at 0 °C by adding 1 mL MeOH, followed by dilution with 100 mL distilled water. The mixture was extracted with MTBE (3 x 50 mL), and the combined organic

phase was washed with brine (1 x 30 mL), dried over anhydrous Na_2SO_4, filtered and concentrated under reduced pressure. Purification by recrystallization (with hexanes) afforded the corresponding bis-boronate **S8(*i*Pr^F)** as a yellow solid (989 mg, 73%).
^{1}H NMR (501 MHz, CD_2Cl_2) δ 8.59 (s, 2H), 8.26 (d, *J* = 2.2 Hz, 2H), 7.48 (dd, *J* = 9.1, 2.0 Hz, 2H), 7.36 (d, *J* = 9.1 Hz, 2H), 4.92 (d, *J* = 6.4 Hz, 2H), 4.82 (d, *J* = 6.4 Hz, 2H), 2.21 (s, 6H), 1.40 (s, 24H).
^{11}B NMR (161 MHz, CD_2Cl_2) δ 30.20.
^{19}F NMR (471 MHz, CD_2Cl_2) δ –75.86 (d, *J* = 7.2 Hz, 12F), –182.12 (hept, *J* = 7.4 Hz, 2F).
^{13}C NMR (126 MHz, CD_2Cl_2) δ 159.7 (C), 140.7 (CH), 136.9 (C), 131.2 (C), 129.7 (d, *J* = 2.1 Hz, C), 127.9 (d, *J* = 2.3 Hz, CH), 127.4 (d, *J* = 11.3 Hz, CH), 127.1 (C), 125.4 (C), 124.7 (C), 123.2 (d, *J* = 10.4 Hz, CH), 123.1 (C), 123.0 (C), 122.3 (d, *J* = 28.1 Hz, C), 120.0 (d, *J* = 28.0 Hz, C), 117.9 (C), 100.8 (CH_2), 95.4 (C), 93.3 (C), 93.0 (C), 92.7 (C), 91.6 (C), 91.4 (C), 91.1 (C), 84.6 (C), 55.7 (CH_3), 25.08 (CH_3), 25.06 (CH_3).
ESI-HRMS: calculated for $C_{42}H_{42}B_2F_{14}O_8Na^+$ ($[M+Na]^+$): 985.2734, found: 985.2738.
$[\alpha_D^{25}]$ = –31.6 (*c* = 0.72, CH_2Cl_2).

(*S*)-6,6'-bis(perfluoropropan-2-yl)-3,3'-bis(4-(trifluoromethyl)phenyl)-[1,1'-binaphthalene]-2,2'-diol (**S9e**)

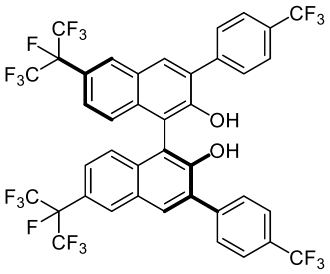

Following *General Procedure E*, but using (*S*)-**S8(*i*Pr^F)** as modified BINOL-diboronate (150 mg, 0.15 mmol, 1 equiv.), and 4-bromobenzotrifluoride (88 mg, 0.39 mmol, 2.5 equiv.) as coupling partner. The crude product was purified by flash column chromatography (eluent: *n*-pentane/MTBE 100:0 → 95:5) to give **S9e** as a light yellow solid (132 mg, 93%).
^{1}H NMR (501 MHz, CD_2Cl_2) δ 8.30 (s, 2H), 8.21 (s, 2H), 7.90 (d, *J* = 8.1 Hz, 4H), 7.80 (d, *J* = 8.1 Hz, 4H), 7.56 (d, *J* = 9.0 Hz, 2H), 7.37 (d, *J* = 9.0 Hz, 2H), 5.60 (s, 2H).
^{19}F NMR (471 MHz, CD_2Cl_2) δ –62.90 (s, 6F), –75.79 (h, *J* = 6.4 Hz, 12F), –182.09 (hept, *J* = 6.9 Hz, 2F).
^{13}C NMR (126 MHz, CD_2Cl_2) δ 152.5 (C), 141.0 (C), 134.6 (C), 133.4 (CH), 131.5 (C), 130.5 (CH), 130.4 (q, *J* = 32.5 Hz, C), 129.1 (d, *J* = 2.1 Hz, C), 128.0 (C), 127.7 (d, *J* = 11.8 Hz, CH), 125.9 (q, *J* = 3.7 Hz, CH), 125.6 (d, *J* = 2.2 Hz, CH), 124.1 (d, *J* = 9.5 Hz, CH), 123.7 (C), 123.2 (d, *J* = 20.4 Hz, C), 122.3 (d, *J* = 27.6 Hz, C), 121.5 (C), 120.0 (d, *J* = 28.1 Hz, C), 112.2 (C), 93.0 (hept, *J* = 33.1 Hz, C), 91.4 (hept, *J* = 33.2 Hz, C).
ESI-HRMS: calculated for $C_{40}H_{17}F_{20}O_2^-$ ($[M-H]^-$): 909.0915, found: 909.0927.
$[\alpha_D^{25}]$ = –61.4 (*c* = 0.17, $CHCl_3$).

7.2. Synthesis of imino-imidodiphosphates

General Procedure F: One-pot synthesis of iIDPs

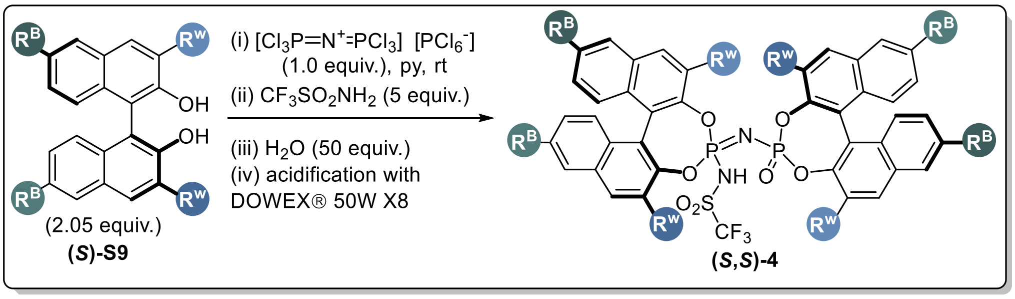

This is a slight adaptation from a reported procedure by our group.[26]

A- Synthesis and purification

An oven-dried Schlenk flask was charged with hexachlorobisphosphazonium hexachlorophosphate[26] (107 mg, 0.20 mmol, 1 equiv.) and the corresponding (*S*)-BINOL (**S9**, 0.42 mmol, 2.1 equiv.) under Ar. After drying the mixture under vacuum (10^{-3} mbar) for 30 min at rt and switching back to Ar atmosphere, pyridine (2 mL) was added in one portion and the mixture was vigorously stirred for 2 h. Trifluoromethanesulfonamide (149 mg, 1.0 mmol, 5 equiv.) was added in one portion and the mixture was further stirred at rt overnight, followed by the addition of distilled water (0.18 mL, 10 mmol, 50 equiv.) and additional stirring for 2 h. The reaction was stopped by diluting the mixture with CH_2Cl_2 (10 mL) and adding aq. HCl 3 M (10 mL); after stirring for 30 min, the aqueous phase was extracted with CH_2Cl_2 (3 x 30 mL). The combined organic phases were washed with distilled water (1 x 30 mL) and brine (1 x 30 mL), then dried over anhydrous Na_2SO_4, filtered and concentrated under reduced pressure. Purification by flash column chromatography on silica gel (*n*-hexane/EtOAc mixtures) afforded the corresponding *i*IDPs as salts.

B- Acidification

DOWEX® 50W X8 (approx. 5 g) was suspended in aq. H_2SO_4 0.5 M, transferred to a glass column with stopcock, and washed thoroughly with more acid (approximately 20 times the volume of the DOWEX pad) until the eluate was colorless. Then, the resin pad was washed with distilled water (until the eluate is neutral to pH indicator paper), ethanol and Et_2O, in that order. For each of these washing operations, ~10–20 times of pad volume was used as volume of the washing agent.

The purified *i*IDP salt (after column, dissolved in 5–10 mL Et_2O) was added to the DOWEX pad (packed in Et_2O) and the eluate was collected in test tubes (adding more Et_2O to not let the DOWEX pad run dry). The eluted fractions were re-added to the DOWEX pad to ensure full acidification: the collection and re-acidification was done 3x in total. After washing the DOWEX pad with Et_2O, the eluate was concentrated under reduced pressure to give a light yellow solid. This solid was re-dissolved in a small amount of Et_2O (1–2 mL) and treated with *n*-pentane (20 mL). After removing the solvents under reduced pressure, the obtained solid was further freeze-dried with liquid N_2 (3x) and dried overnight under high-vacuum, affording thus the acidified imino-imidodiphosphate **4**.

1,1,1-trifluoro-*N*-((11b*S*)-4-(((11b*S*)-4-oxido-2,6-bis(4-(trifluoromethyl)phenyl)dinaphtho[2,1-*d*:1',2'-*f*][1,3,2]dioxaphosphepin-4-yl)imino)-2,6-bis(4-(trifluoromethyl)phenyl)-4λ⁵-dinaphtho[2,1-*d*:1',2'-*f*][1,3,2]dioxaphosphepin-4-yl)methanesulfonamide (*i*IDP **4b**)

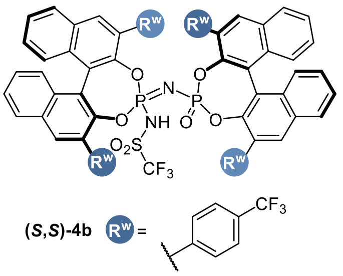

Following *General Procedure F*, employing (*S*)-BINOL **S9a** (603 mg, 1.0 mmol) as starting material. The crude product was purified by flash column chromatography (eluent: *n*-pentane/CH_2Cl_2 100:0 → 70:30) and acidified with DOWEX® 50W X8, to give **4b** as an off-white solid (530 mg, 77%).

^{1}H NMR (501 MHz, CD_2Cl_2) δ 8.22 (d, *J* = 8.2 Hz, 2H), 8.12 (d, *J* = 17.0 Hz, 2H), 8.07 (dd, *J* = 8.3, 5.3 Hz, 2H), 7.84 (dddd, *J* = 12.5, 8.1, 5.8, 2.2 Hz, 2H), 7.72 – 7.57 (m, 12H), 7.52 – 7.39 (m, 8H), 7.03 (d, *J* = 8.1 Hz, 2H), 6.98 (d, *J* = 8.1 Hz, 2H), 6.78 (d, *J* = 8.0 Hz, 2H), 6.72 (d, *J* = 8.0 Hz, 2H).
^{19}F NMR (471 MHz, CD_2Cl_2) δ –62.58 (s, 3F), –62.73 (s, 3F), –62.77 (s, 3F), –63.16 (s, 3F), –80.03 (s, 3F).
^{31}P NMR (203 MHz, CD_2Cl_2) δ –2.35 (d, *J* = 111.5 Hz), –7.81 (d, *J* = 111.4 Hz).
^{13}C NMR (126 MHz, CD_2Cl_2) δ 144.3, 144.2, 143.9, 143.8, 143.7, 140.4, 140.1, 140.0, 132.94, 132.87, 132.8, 132.7 (CH), 132.5, 132.33, 132.27, 132.1 (CH), 131.6 (CH), 131.1 (CH), 130.8 (CH), 130.3, 130.2 (CH), 130.1 (CH), 130.0 (CH), 129.8, 129.6, 129.4 (CH), 129.3 (CH), 129.2 (CH), 128.5 (CH), 128.2 (CH), 127.9 (CH), 127.8 (CH), 127.7 (CH), 127.5 (CH), 127.42 (CH), 127.36 (CH), 127.22 (CH), 127.19 (CH), 127.1 (CH), 127.0 (CH), 125.84 (CH), 125.81 (CH), 125.11 (CH), 125.07 (CH), 125.00 (CH), 124.97 (CH), 124.73 (CH), 124.69 (CH), 124.0, 123.94, 123.91, 122.7, 122.5.
ESI-HRMS: calculated for $C_{69}H_{36}F_{15}N_2O_7P_2S^-$ ([M–H]⁻): 1383.1485, found: 1383.1483.
[α_D^{25}] = +314.0 (*c* = 0.23, $CHCl_3$).

1,1,1-trifluoro-*N*-((11b*S*)-4-(((11b*S*)-4-oxido-2,6-bis(3-bromo-4-(trifluoromethyl)phenyl)dinaphtho[2,1-*d*:1',2'-*f*][1,3,2]dioxaphosphepin-4-yl)imino)-2,6-bis(3-bromo-4-(trifluoromethyl)phenyl)-4λ⁵-dinaphtho[2,1-*d*:1',2'-*f*][1,3,2]dioxaphosphepin-4-yl)methanesulfonamide (*i*IDP **4c**)

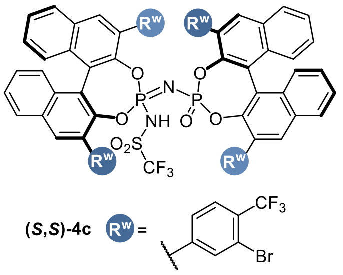

Following *General Procedure F*, employing (*S*)-BINOL **S9b** (384 mg, 0.52 mmol) as starting material. The crude product was purified by flash column chromatography (eluent: *n*-pentane/CH_2Cl_2 100:0 → 70:30) and acidified with DOWEX® 50W X8, to give **4c** as an off-white solid (286 mg, 67%).

^{1}H NMR (501 MHz, CD_2Cl_2) δ 8.22 (s, 1H), 8.15 (s, 1H), 8.09 (t, *J* = 6.7 Hz, 4H), 7.86 (dd, *J* = 13.5, 6.0 Hz, 3H), 7.80 (d, *J* = 8.6 Hz, 1H), 7.76 (d, *J* = 8.5 Hz, 1H), 7.73 – 7.64 (m, 5H), 7.61 (d, *J* = 7.3 Hz, 2H), 7.48 – 7.28 (m, 9H), 7.25 (s, 1H), 6.99 (t, *J* = 9.0 Hz, 2H), 6.69 (d, *J* = 8.2 Hz, 1H), 6.59 (d, *J* = 8.2 Hz, 1H).
^{19}F NMR (471 MHz, CD_2Cl_2) δ –62.24 (s, 3F), –62.51 (s, 3F), –62.64 (s, 3F), –63.04 (s, 3F), –80.13 (s, 3F).
^{31}P NMR (203 MHz, CD_2Cl_2) δ –3.53 (d, *J* = 111.3 Hz), –7.76 (d, *J* = 111.3 Hz).
^{13}C NMR (126 MHz, CD_2Cl_2) δ 144.1, 144.0, 143.9, 143.7, 143.6, 142.7, 141.7, 141.6, 141.5, 141.4, 136.2, 135.6, 135.5, 135.4, 132.8, 132.7, 132.61, 132.55, 132.4, 132.3, 132.2, 131.94, 131.90, 131.7, 131.4, 131.3, 131.00, 130.97, 130.78, 130.76, 129.7, 129.6, 129.5, 129.4, 129.2, 128.9, 128.8, 128.64,

128.55, 128.2, 128.0, 127.92, 127.88, 127.84, 127.81, 127.75, 127.5, 127.4, 127.3, 127.2, 127.0, 124.7, 124.52, 124.46, 124.3, 124.02, 124.00, 123.84, 123.82, 122.8, 122.7, 122.5, 122.4, 122.1, 120.2, 120.0, 119.8, 119.7.
ESI-HRMS: calculated for $C_{69}H_{32}Br_4F_{15}N_2O_7P_2S^-$ ([M–H]⁻): 1694.7906, found: 1694.7904.
$[\alpha_D^{25}]$ = +279.3 (*c* = 0.23, $CHCl_3$).

1,1,1-trifluoro-*N*-((11b*S*)-4-(((11b*S*)-4-oxido-2,6-bis(4-(perfluoropropyl)phenyl)dinaphtho[2,1-*d*:1',2'-*f*][1,3,2]dioxaphosphepin-4-yl)imino)-2,6-bis(4-(perfluoropropyl)phenyl)-4λ⁵-dinaphtho[2,1-*d*:1',2'-*f*][1,3,2]dioxaphosphepin-4-yl)methanesulfonamide (*i*IDP **4d**)

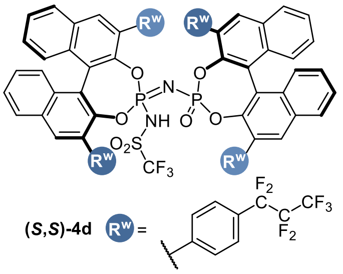

Following *General Procedure F*, employing (*S*)-BINOL **S9c** (119 mg, 0.15 mmol) as starting material. The crude product was purified by flash column chromatography (eluent: *n*-pentane/CH_2Cl_2 100:0 → 70:30) and acidified with DOWEX® 50W X8, to give **4d** as an off-white solid (132 mg, 98%).

^{1}H NMR (501 MHz, CD_2Cl_2) δ 8.22 (dd, *J* = 8.3, 5.6 Hz, 2H), 8.16 (s, 1H), 8.12 (s, 1H), 8.07 (t, *J* = 8.6 Hz, 2H), 7.88 – 7.79 (m, 2H), 7.73 (s, 1H), 7.67 – 7.57 (m, 9H), 7.53 (dt, *J* = 16.7, 8.4 Hz, 5H), 7.46 – 7.35 (m, 5H), 7.00 (d, *J* = 8.1 Hz, 2H), 6.93 (d, *J* = 8.1 Hz, 2H), 6.78 (d, *J* = 8.2 Hz, 2H), 6.74 (d, *J* = 8.2 Hz, 2H).
^{19}F NMR (471 MHz, CD_2Cl_2) δ –79.96 (s, 3F), –80.35 – –80.54 (m, 12F), –110.29 – –112.62 (m, 8F), –126.48 – –126.70 (m, 8F).
^{31}P NMR (203 MHz, CD_2Cl_2) δ -1.53 (d, *J* = 107.7 Hz), -7.14 (d, *J* = 108.4 Hz).
^{13}C NMR (151 MHz, CD_2Cl_2) δ 144.3, 144.2, 143.9, 143.8, 143.02, 142.96, 140.6, 140.3, 140.2, 133.03, 133.01, 132.9, 132.84, 132.83, 132.78, 132.77, 132.7, 132.60, 132.58, 132.49, 132.48, 132.4, 132.3, 132.24, 132.21, 132.20, 132.1, 132.02, 132.00, 131.7, 131.2, 130.7, 130.5, 130.1, 129.99, 129.91, 129.4, 129.3, 129.2, 128.6, 128.4, 128.3, 128.1, 127.91, 127.86, 127.8, 127.7, 127.5, 127.40, 127.36, 127.32, 127.28, 127.19, 127.16, 127.04, 126.97, 126.7, 126.63, 126.58, 126.53, 126.49, 126.25, 126.21, 126.16, 124.00, 123.98, 123.97, 123.95, 122.74, 122.72, 122.5, 121.6, 121.3, 121.1, 120.70, 120.68, 119.7, 119.4, 119.24, 119.19, 119.02, 118.96, 118.6, 117.7, 117.5, 117.3, 117.2, 117.1, 117.0, 116.4, 115.8, 115.7, 115.6, 114.2, 114.0, 111.05, 110.99, 110.8, 110.7, 110.54, 110.49, 109.5, 109.3, 109.2, 109.05, 108.99, 108.8, 108.7, 108.5, 107.6, 107.5, 107.3, 107.2, 107.0.
ESI-HRMS: calculated for $C_{77}H_{36}F_{31}N_2O_7P_2S^-$ ([M–H]⁻): 1783.1229, found: 1783.1237.
$[\alpha_D^{25}]$ = +336.0 (*c* = 0.17, $CHCl_3$).

1,1,1-trifluoro-*N*-((11b*S*)-4-(((11b*S*)-4-oxido-2,6-bis(6-(perfluoropropan-2-yl)naphthalen-2-yl)dinaphtho[2,1-*d*:1',2'-*f*][1,3,2]dioxaphosphepin-4-yl)imino)-2,6-bis(6-(perfluoropropan-2-yl)naphthalen-2-yl)-4λ⁵-dinaphtho[2,1-*d*:1',2'-*f*][1,3,2]dioxaphosphepin-4-yl)methanesulfonamide (*i*IDP **4e**)

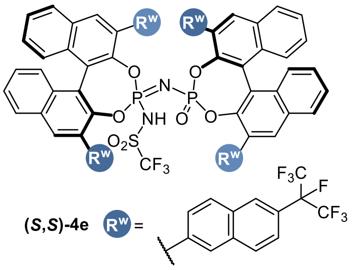

Following *General Procedure F*, employing (*S*)-BINOL **S9d** (350 mg, 0.40 mmol) as starting material. The crude product was purified by flash column chromatography (eluent: *i*-hexane/EtOAc 97:3 → 80:20) and acidified with DOWEX® 50W X8, to give **4e** as an off-light yellow solid (261 mg, 69%).

^{1}H NMR (501 MHz, CD_2Cl_2) δ 8.22 (t, *J* = 7.0 Hz, 2H), 8.14 – 8.04 (m, 5H), 7.98 – 7.87 (m, 7H), 7.80 (dt, *J* = 19.9, 6.9 Hz, 2H), 7.71 (dd, *J* = 8.9, 5.3 Hz, 2H), 7.66 – 7.41 (m, 18H), 7.39 (d, *J* = 8.7 Hz, 2H), 7.36 (s, 1H), 7.32 (s, 1H), 6.85 (dd, *J* = 8.7, 1.9 Hz, 1H), 6.77 (dd, *J* = 8.7, 1.9 Hz, 1H), 6.42 – 6.33 (m, 2H).
^{19}F NMR (471 MHz, CD_2Cl_2) δ –75.69 – –76.12 (m, 24F), –80.84 (s, 3F), –181.95 (dhept, *J* = 21.5, 7.1 Hz, 2F), –182.19 (dhept, *J* = 21.9, 7.1 Hz, 2F).
^{31}P NMR (203 MHz, CD_2Cl_2) δ –4.69 (d, *J* = 120.7 Hz), –9.79 (d, *J* = 120.3 Hz).
^{13}C NMR (126 MHz, CD_2Cl_2) δ 144.4, 144.1, 136.3, 136.1, 135.8, 134.3, 134.2, 134.1, 134.0, 133.6, 133.5, 133.18, 133.15, 133.1, 133.0, 132.6, 132.5, 132.43, 132.38, 132.3, 132.12, 132.06, 132.0, 131.9, 131.7, 131.6, 129.8, 129.72, 129.67, 129.6, 129.5, 129.4, 129.3, 129.2, 129.1, 128.8, 128.7, 128.6, 128.5, 128.4, 128.2, 128.1, 128.0, 127.84, 127.77, 127.64, 127.56, 127.5, 127.3, 127.1, 126.9, 126.4, 126.2, 126.1, 124.8, 124.7, 124.6, 124.5, 124.4, 124.24, 124.17, 124.15, 124.1, 123.92, 123.88, 123.86, 122.72, 122.70, 122.4, 122.2, 121.9, 121.7, 121.6.
ESI-HRMS: calculated for $C_{93}H_{44}F_{31}N_2O_7P_2S^-$ ([M–H]$^-$): 1983.1855, found: 1983.1863.
$[\alpha_D^{25}]$ = +232.7 (*c* = 0.21, $CHCl_3$).

1,1,1-trifluoro-*N*-((11b*S*)-4-(((11b*S*)-4-oxido-9,14-bis(perfluoropropan-2-yl)-2,6-bis(4-(trifluoromethyl)phenyl)dinaphtho[2,1-*d*:1',2'-*f*][1,3,2]dioxaphosphepin-4-yl)imino)-9,14-bis(perfluoropropan-2-yl)-2,6-bis(4-(trifluoromethyl)phenyl)-4λ⁵-dinaphtho[2,1-*d*:1',2'-*f*][1,3,2]dioxaphosphepin-4-yl)methanesulfonamide (*i*IDP **4b'**)

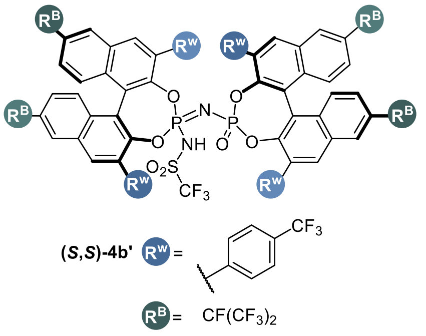

Following *General Procedure F*, employing (*S*)-BINOL **S9e** (326 mg, 0.35 mmol) as starting material. The crude product was purified by flash column chromatography (eluent: *n*-pentane/CH_2Cl_2 100:0 → 70:30) and acidified with DOWEX® 50W X8, to give **4b'** as a light yellow solid (326 mg, 91%).

^{1}H NMR (501 MHz, CD_2Cl_2) δ 8.55 (s, 2H), 8.39 (s, 2H), 8.25 (d, *J* = 7.0 Hz, 2H), 7.91 – 7.81 (m, 4H), 7.76 (t, *J* = 9.0 Hz, 2H), 7.69 – 7.59 (m, 7H), 7.56 (d, *J* = 9.2 Hz, 1H), 7.47 (t, *J* = 8.8 Hz, 4H), 6.92 – 6.83 (m, 6H), 6.80 (d, *J* = 8.1 Hz, 2H).
^{19}F NMR (471 MHz, CD_2Cl_2) δ –62.86 (s, 6F), –62.88 (s, 3F), –63.27 (s, 3F), –75.52 – –75.59 (m, 16F), –75.66 (dp, *J* = 20.8, 8.2 Hz, 8F), –79.94 (s, 3F), –182.13 (q, *J* = 6.9 Hz, 3F), –182.24 (p, *J* = 7.3 Hz, 1F).
^{31}P NMR (203 MHz, CD_2Cl_2) δ –1.75 (d, *J* = 101.0 Hz), –5.93 (d, *J* = 100.8 Hz).
^{13}C NMR (126 MHz, CD_2Cl_2) δ 139.8, 139.3, 139.1, 134.7, 134.5, 134.4, 133.7, 133.6, 133.5, 133.2, 133.0, 132.3, 131.9, 131.5, 130.8, 130.2, 130.1, 129.9, 128.3, 128.0, 127.9, 126.1, 125.5, 125.32, 125.29, 124.9, 124.7, 123.8, 123.7, 123.2, 122.2, 120.1.
ESI-HRMS: calculated for $C_{81}H_{32}F_{43}N_2O_7P_2S^-$ ([M–H]$^-$): 2055.0725, found: 2055.0740.
$[\alpha_D^{25}]$ = +227.7 (*c* = 0.19, $CHCl_3$).

8. Computational Studies

METHOD

All calculations presented in this paper were carried out with a development version of the ORCA suite of programs base on version 4.2.[27] Molecular geometries were optimized in the gas phase using the PBE functional[28] in conjunction with the D3 version of Grimme's dispersion correction with Becke-Johnson damping function,[29] using the resolution of identity approximation. The def2-SVP basis set was used for all atoms with matching auxiliary basis.[30] Analytic frequency calculations were performed to verify the nature of all stationary points (minima and transition states) and to calculate free energies and enthalpies at 298 K by using the rigid-rotor harmonic oscillator (RRHO) approximation. Solvation effect has been accounted by using CPCM (cyclohexane) solvation model,[31] as implemented in ORCA. An exhaustive manual conformational search was performed for possible catalyst-substrate orientations. Transition state (TS) structures were verified by the presence of a single imaginary vibrational frequency. Single-point energies are calculated at M06-2X/def2-TZVP[32] level of theory. Distortion-Interaction analysis[33] has been performed to determine the reason behind the stereoinduction. Molecular structures were generated using CYLview program[34] and VMD.[35]

The main objectives of our computational analysis are the following:

-to understand the confinement effect ("cavity effect"): what causes such a different result during the deuterium-labelling experiments in the presence of achiral *p*-TsOH, and chiral, enantiopure (*S*,*S*)-*i*IDP catalyst (Figures 3B and 3C of main text).

-to probe the origin of stereoselectivity.

8.1. "Open" vs. "confined" acid: Understanding the "cavity effect"

The results from the experiments with deuterium-containing reagents (Sections 6.1 and 6.2) showed a significant difference in the reaction pathways, when either *p*-TsOH or an *i*IDP are utilized as catalysts. Inspired by the work of Kupova and coworkers,[36] we conducted several computational calculations to study the addition of styrene (**1a**) to a formaldehyde-derived aldehydium ion ("*truncated electrophile*"), in the presence of the anion from the deprotonated catalyst (either *p*-TsOH or *i*IDP, Scheme S-3). It is worth to mention that, in contrast to the study from Kupova, our proposed truncated electrophile does not contain a terminal -OH group, in order to describe more accurately the oligomeric structure of paraformaldehyde, as well as to avoid the participation of hydrogen-bond interactions in the calculations.

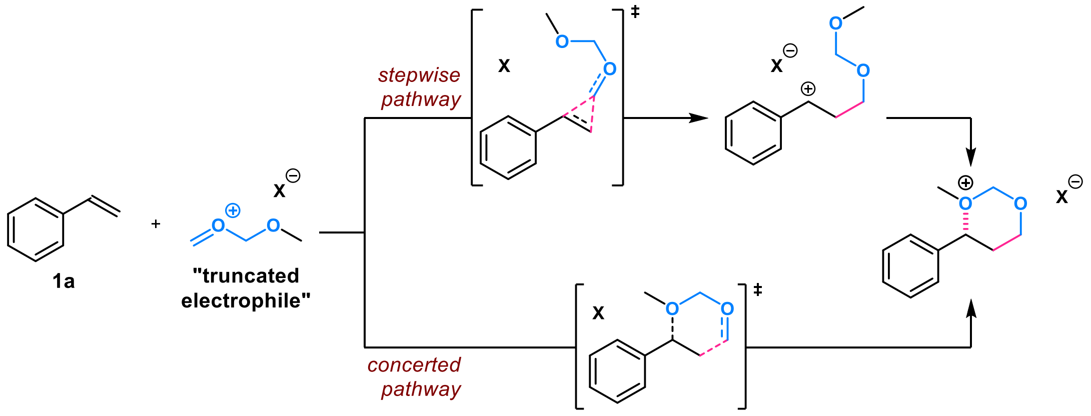

Scheme S-3. Effect of confinement on the operative reaction pathway.

The results from the computations were the following (see Table S-4):

(a) With the *p*-toluenesulfonate anion:

A transition-state assembly **TS-1** was found, resembling the structure of an "-onium cation", where both carbon atoms from the olefin approach the electrophile (distances C···C: 2.82 Å and 2.95 Å, respectively). Notably, the bond distance between the benzylic carbon and the remote oxygen found to be 4.95 Å, ruling out any possibility of a concerted cyclization. This indicates that a stepwise mechanism is operative here via the stable benzyl cation as intermediate. Furthermore, changing the displacement variable along the imaginary vibration mode indicates that the observed TS indeed leads to a more stable non-cyclized intermediate **C1**. The intermediacy of such a carbocation matches both the observed differences in deuterium incorporation for the reaction with $(HCHO)_n$/$(DCDO)_n$ mixtures, and the *cis*-/*trans*-scrambling in the Prins reaction of β-deuterostyrenes.

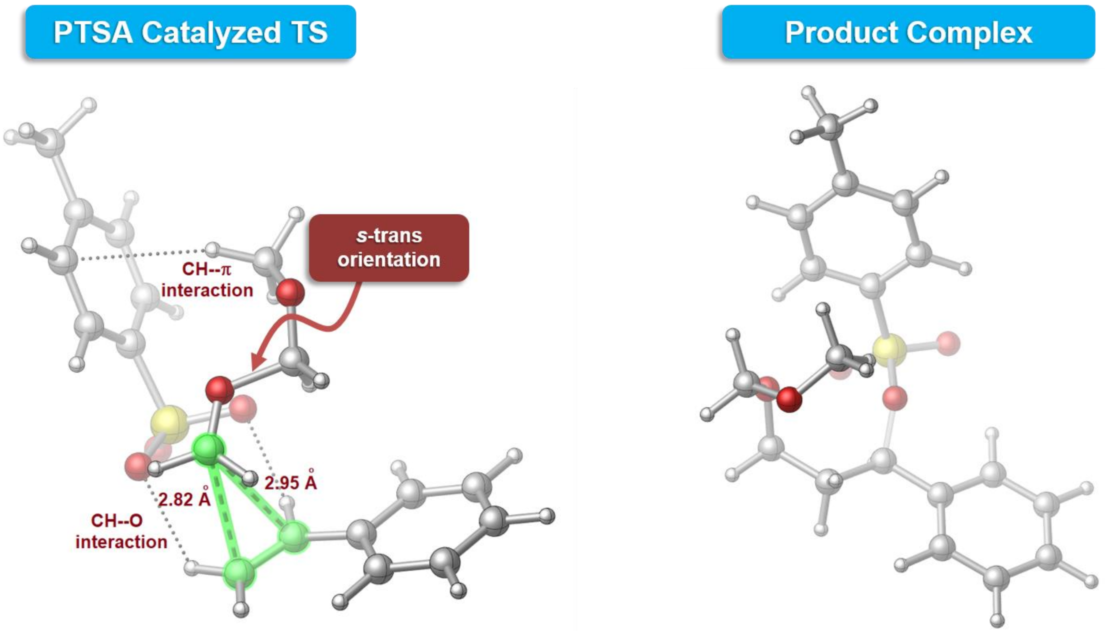

*Figure S-3. **TS-1** (left) and subsequent intermediate **C1** (right), computed at the PBE-D3/def2-SVP level of theory.*

(b) With the *i*IDP anion:

The nature of the TS is strikingly different in the presence of a confined, enantiopure (*S*,*S*)-*i*IDP anion (catalyst **4b**). In this case, we could only observe **TS-2**, where the distance between the atoms forming the C–C bond is around 2 Å (Figure S-4). A highly asynchronous, concerted chair-like TS structures was obtained where C–C bond formation happens prior to the O–C bond formation. Furthermore, the confined cavity induces the dimeric electrophile to adopt a conformation where the nucleophilic oxygen atom stays closer to the benzylic center (C···O distance = 3.02 Å). By slight displacement from the TS structure toward product and subsequent optimization, a quick collapse of the TS to the formation of the C–O bond is observed, that leads to direct cyclization (intermediate **C2**). This implies that the carbocationic intermediate is virtually non-existent within the catalyst cavity and the transformation follows rather a dynamically concerted pathway. This result is in agreement with the observed high stereospecificity for the *i*IDP-catalyzed Prins reaction with β-deutero-styrenes.

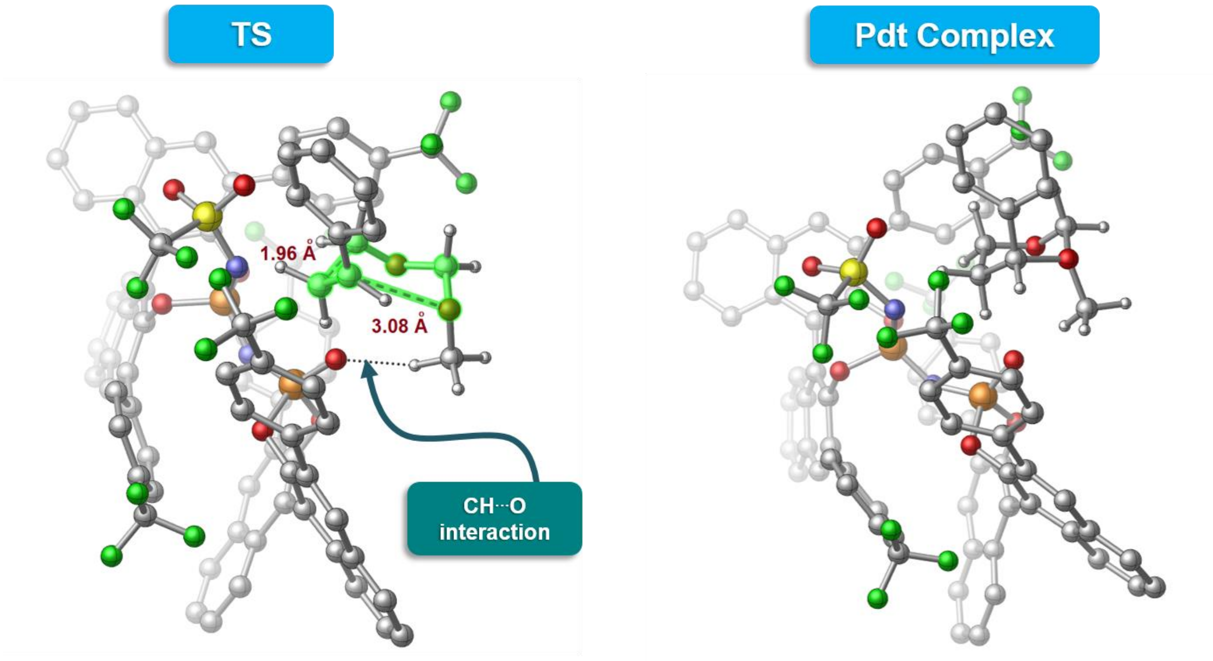

*Figure S-4. **TS-2** (left) and subsequent intermediate **C2** (right), computed at the PBE-D3/def2-SVP level of theory.*

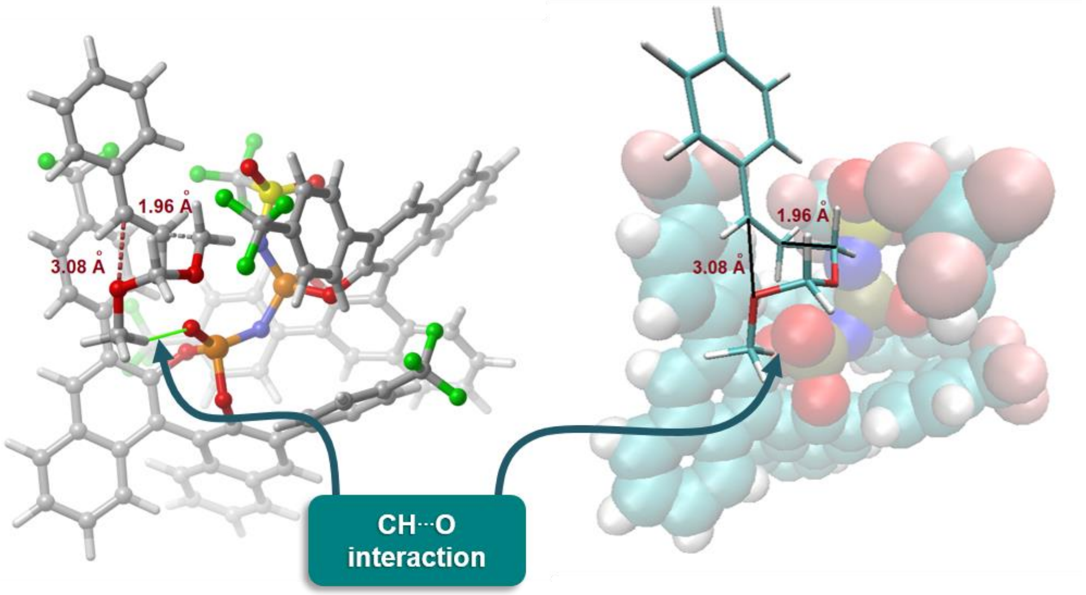

*Figure S- 5. A different perspective of **TS-2** (by rotating 180 degrees). The additional CH···O interaction within the confined cavity promotes an s-cis configuration of the electrophile.*

Table S-4. Comparison of p-TsOH and iIDP: TS energetics at the M06-2X/def2-TZVP+ CPCM(cyclohexane)//PBE-D3/def2-SVP level of theory.

TS No.	PBE RRHO corrections	M06-2X/def2-TZVP single point *(solvent: CyH)*	Imaginary Freq.	ΔG(TS) Final
Catalyst: *p*-TsOH				
TS-1	0.08029937	–1473.3172402	–118.36	–1473.2369
Catalyst: *i*IDP **4b**				
TS-2	0.20778449	–6444.62259	- 218.51	–6444.4148

8.2. *Understanding the Stereoselectivity*

Next, we turned our attention to understand the reason for the enantioselectivity in presence of catalyst **4b**, modeling the transition states toward the formation of each product enantiomer. Both TS structures adopt a chair-like conformation, where the initial C–C bond formation precedes the subsequent C–O collapse (Figure S-6). Based on the free energy difference of the optimized TS structures at 298 K (Table S-5), the predicted enantioselectivity (er = 99:1, at the M06-2X/def2-TZVP+ CPCM(cyclohexane)//PBE-D3/def2-SVP level of theory) is in good agreement with the experimental observation (er = 94.5:5.5). Moreover, the Ph group in the stereodetermining TS is projected outside the cavity, explaining the high substrate group tolerance observed for this transformation. Employing the Distortion-Interaction (DI) analysis, we observed that the energy difference between two enantiodifferentiating TS structures (**TS-2** and **TS-3**) originates from unfavorable distortion effects (see Table S-6). Presumably, such a distortion might cause due to the gain in additional CH···F interaction during the distorted structure. Notably, DI analysis revealed that much of the distortion was resulting from the twisted chair like substrate arrangement in the TS leading to the minor stereoisomer

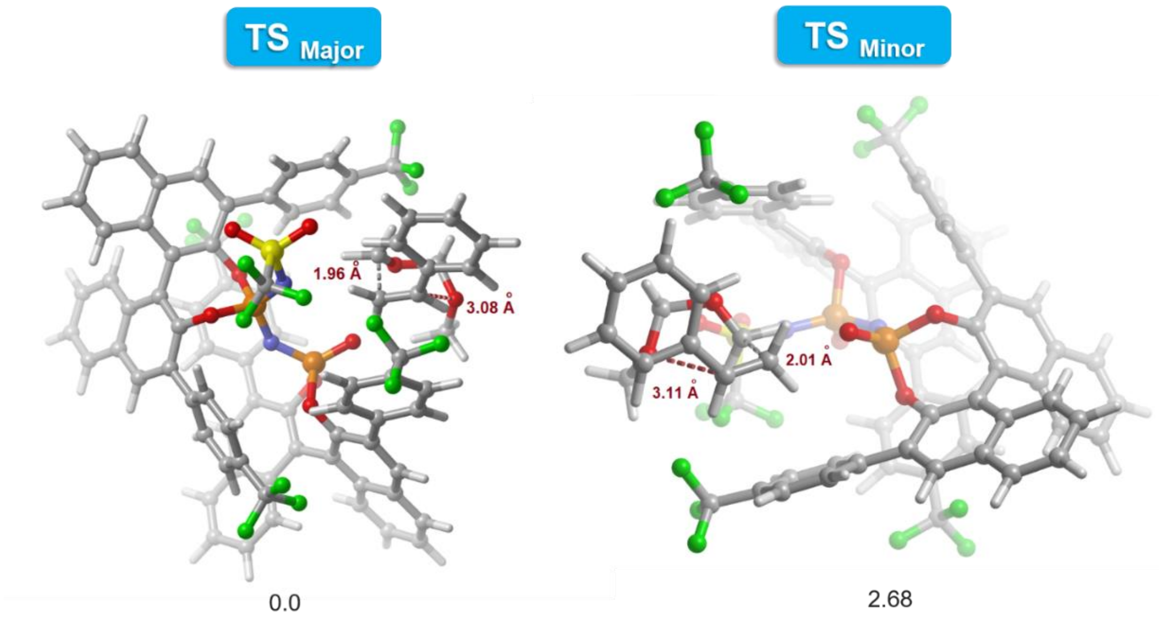

Figure S-6. Enantio-determining TS structures: highly asynchronous, concerted chair-like TS structures where C–C bond formation happens prior to the O–C bond formation (ΔΔG‡ in kcal/mol, computed at the M06-2X/def2-TZVP+ CPCM(cyclohexane)//PBE-D3/def2-TZVP+ CPCM(cyclohexane)//PBE-D3/def2-SVP level of theory).

Notably, the catalyst counteranion leading to the minor isomer of the TS ((*S*)-enantiomer of product) is more distorted to accommodate the substrate (Table S-6). This also highlights the importance of suitable cavity size in achieving the required stereoinduction.

Table S-5. TS energetics for the formation of each enantiomer of the Prins product, at the M06-2X/def2-TZVP+ CPCM(cyclohexane)//PBE-D3/def2-SVP level of theory.

**TS No.**	**PBE RRHO corrections**	**M06-2X/def2-TZVP single point *(solvent: CyH)***	**Imaginary Freq.**	**ΔG(TS) Final**	**Relative TS Energy (kcal/mol)**
Major enantiomer: (*R*)-**3a**					
TS-2	0.20723631	–6444.626477	–303.94	–6444.4191	0.0
Minor enantiomer: (*S*)-**3a**					
TS-3	0.20778449	–6444.62259	- 218.51	–6444.4148	2.68

Based on the optimized structures, the predicted enantioselectivity at the M06-2X/def2-TZVP+ CPCM(cyclohexane)//PBE-D3/def2-SVP level of theory at 298 K (er = 99:1) is in good agreement with the experimentally observed enantioselectivity (er = 94.5:5.5).

Table S-6. Distortion-Interaction analysis to understand the selectivity for the intermolecular Prins reaction.

**TS No.**	**M06-2X/def2-TZVP single point (gas phase)**	**Relative Energy ($\Delta\Delta E_{gas}^{‡}$, kcal/mol)**
TS-2	–6444.604631	2.3
TS-3	–6444.600944	
Substrates Only ($\Delta\Delta E_{sub}^{‡}$)		
Subst_TS-2	–578.2826397	1.8
Subst_TS-3	–578.2797785	
Catalyst Only ($\Delta\Delta E_{sub}^{‡}$)		
Cat_TS-2	–5866.1760818	0.31
Cat_TS-3	–5866.1755331	
Total net Distortion in TS leading to minor enantiomer ($\Delta\Delta E_{dist\_total}^{‡}$)		2.11
Total Interaction		0.2

8.3. Optimized Cartesian Coordinates (PBE-D3/def2-SVP)

TS-1

Imaginary frequency = –118.36

6	0.309765654	2.168394803	-4.772646504
6	-3.210091370	2.445729570	-3.813448554
6	-3.878021551	1.263455240	-3.887938443
6	-1.363069515	0.363249689	-4.812650625
1	-2.600244514	2.621436679	-2.908428959
1	-1.716191108	-0.606101640	-4.424772065
1	-1.898678693	0.934241161	-5.594539834
1	-3.794768531	0.549184652	-3.054697460
1	-4.505487574	0.987669131	-4.753220614
6	-3.131247154	3.489231942	-4.843029242
6	-2.395916903	4.665985368	-4.557559103
6	-3.715564084	3.363627105	-6.127972461
6	-2.250269814	5.677933697	-5.515206722
1	-1.938630721	4.772728775	-3.561098725
6	-3.567799065	4.373823326	-7.085928996
1	-4.299705516	2.464190313	-6.377186331
6	-2.834350208	5.535086418	-6.784553947
1	-1.678042390	6.585705581	-5.270521004
1	-4.031078087	4.258379705	-8.077737089
1	-2.720488873	6.327834172	-7.539276926
1	-0.191970100	2.768726722	-3.987697593
8	1.642528572	2.133900196	-4.726747662
8	-0.234364225	0.751602790	-4.400134999
1	-0.045678090	2.358552746	-5.806026537
6	2.231642092	2.129213312	-3.405952143
1	1.519341999	2.530010610	-2.656681391
1	2.486754755	1.088820139	-3.120519308
1	3.152599698	2.739063418	-3.460383396
6	2.326861454	-1.596067410	-1.736840092
6	3.249564435	-0.988350780	-0.862354280
6	2.810385934	0.114355028	-0.094109592
6	1.502364593	0.598911362	-0.202145865
6	0.606896061	-0.013388563	-1.096015320
6	1.011705431	-1.117797506	-1.854646775
1	2.644863080	-2.464907348	-2.335614475
1	3.511871720	0.597507366	0.605350303
1	1.159639655	1.452028553	0.402244434
1	0.286164081	-1.592061289	-2.530451357
6	4.656585436	-1.511910775	-0.719529056
1	4.792268666	-2.032136517	0.252810354
1	4.908373766	-2.233998579	-1.520369513
1	5.402153722	-0.691090569	-0.748735630
16	-1.034628695	0.717816856	-1.306169216
8	-1.601204191	0.895102297	0.052595099
8	-0.730012780	2.031415180	-2.014959988
8	-1.798983758	-0.242888708	-2.196740597

Pdt Complex of TS-1

6	1.904935274	1.195780359	-5.945826078
6	-2.183595069	1.154219433	-5.129353783
6	-1.253111596	1.703414690	-6.207701061
6	-0.274302118	0.685445063	-6.807152178
1	-2.740560205	0.290061751	-5.556596905
1	-0.805885348	-0.259588630	-7.039992011
1	0.128521349	1.099687811	-7.762331168

1	-1.894370561	2.069380016	-7.037648722
1	-0.685675830	2.575477599	-5.825107441
6	-3.187900814	2.142490524	-4.552826482
6	-3.988352427	1.732608812	-3.465148112
6	-3.355546101	3.442529915	-5.064972567
6	-4.938475020	2.600308470	-2.912683112
1	-3.847799487	0.725010946	-3.043617506
6	-4.309829269	4.311953195	-4.510559824
1	-2.740180448	3.793340895	-5.905195887
6	-5.104693671	3.894512102	-3.434417855
1	-5.554759600	2.263240303	-2.065141320
1	-4.426712400	5.325343587	-4.924149778
1	-5.851341005	4.576454887	-2.999678989
1	2.163807085	1.518466170	-6.982047268
8	1.715357775	2.381852630	-5.225252903
8	0.810359440	0.314866041	-5.963317723
1	2.741249382	0.599838696	-5.505807392
6	1.610408945	2.187864580	-3.819687721
1	1.636032172	3.189625457	-3.349583114
1	0.664933970	1.676000750	-3.543589184
1	2.464791579	1.584220105	-3.430614221
6	1.526954847	-0.915004104	-0.977531512
6	2.647355114	-1.361074767	-1.709227781
6	2.467858367	-1.715072631	-3.065256748
6	1.217140900	-1.617461421	-3.684401405
6	0.128620291	-1.165222708	-2.924668974
6	0.262368720	-0.819090299	-1.573187949
1	1.647235112	-0.642322389	0.082417858
1	3.331120276	-2.071330689	-3.649431145
1	1.075571504	-1.858501363	-4.745764663
1	-0.616911332	-0.485508090	-1.004037618
6	4.006387335	-1.468069231	-1.067633498
1	3.993147792	-1.123151777	-0.016282516
1	4.371011624	-2.516424849	-1.078232410
1	4.757126907	-0.863524877	-1.617624029
16	-1.467235256	-0.994315406	-3.698227065
8	-2.517393727	-1.210431526	-2.683126637
8	-1.383384438	0.644006025	-4.014930723
8	-1.514684905	-1.731984988	-4.977291949

Major Isomer

TS-2

Imaginary frequency = –303.94

15	-0.350462660	-0.707857142	-0.915583153
8	1.304920371	-0.442088428	-0.875304737
8	-0.401092100	-2.158833370	-0.094144834
6	2.106661865	-1.471763661	-1.324712369
6	2.683255150	-1.377551937	-2.634508182
6	3.492737395	-2.432592931	-3.052841452
6	3.703150434	-3.592599035	-2.260868885
6	4.492995495	-4.681615483	-2.736651511
6	4.647773043	-5.832884973	-1.981073810
6	4.005526864	-5.940784647	-0.718856508
6	3.246274670	-4.892060319	-0.219904400
6	3.089977215	-3.682149334	-0.959096556
6	2.323483310	-2.564666583	-0.476138952
6	1.737057841	-2.541124537	0.892434573
6	2.553742004	-2.670314868	2.070551533
6	3.976887007	-2.773352330	2.020174177

6	4.727537645	-2.880975063	3.182560684
6	4.094613691	-2.893525644	4.453983059
6	2.717266728	-2.765889413	4.539005165
6	1.917061276	-2.632419317	3.364651662
6	0.515134020	-2.411367190	3.449674139
6	-0.277308375	-2.216997190	2.320120299
6	0.364696808	-2.310863199	1.042226951
6	2.461080860	-0.211489797	-3.526637446
1	3.997439749	-2.371286599	-4.028826066
1	4.968653634	-4.592350653	-3.725870999
1	5.255136887	-6.667998591	-2.361177780
1	4.107351672	-6.866217840	-0.132055071
1	2.748013989	-4.989427657	0.754255394
1	4.479643601	-2.737912185	1.044422425
1	5.823900682	-2.948319489	3.116980909
1	4.700108194	-2.987199328	5.367913649
1	2.215965761	-2.748616390	5.519432872
1	0.047714876	-2.381164301	4.445784560
6	-1.712729870	-1.862142053	2.448619521
6	2.365701138	1.113045322	-3.033535867
6	2.257352348	2.199721432	-3.905925300
6	2.239877083	1.996696787	-5.297521404
6	2.298570430	0.687305555	-5.804498068
6	2.397426061	-0.399004175	-4.926986327
6	-2.112106174	-0.968008863	3.467919673
6	-3.448018934	-0.590684128	3.615944492
6	-4.419472363	-1.089245049	2.731579761
6	-4.043958059	-1.990934483	1.722683368
6	-2.705088318	-2.373973265	1.582843829
1	2.384741186	1.291451104	-1.952508604
1	2.420192450	-1.419694635	-5.336329071
1	-1.354627071	-0.525281164	4.130331718
1	-2.422853415	-3.073632498	0.786366311
7	-0.924325763	0.407850240	0.080496172
15	-1.763561981	1.730126608	-0.077984057
8	-1.172715720	2.928965534	0.900848551
8	-3.211788544	1.428272560	0.676338291
6	-1.200882747	2.710447001	2.271776657
6	0.012545470	2.308853780	2.916342821
6	-0.036614436	2.094097713	4.291511365
6	-1.243302290	2.217194416	5.033788122
6	-1.282812001	1.933683782	6.432131552
6	-2.475927448	1.977972327	7.135864605
6	-3.683301451	2.299801165	6.460377762
6	-3.676915996	2.599050231	5.105263292
6	-2.461677956	2.588839683	4.356596592
6	-2.414755513	2.871199387	2.945409133
6	-3.629808846	3.256505261	2.177413767
6	-4.413566319	4.414472702	2.519438850
6	-4.034118255	5.329096723	3.545990088
6	-4.821360715	6.430656858	3.849212493
6	-6.027657635	6.674506518	3.141118613
6	-6.409588161	5.821745237	2.117867615
6	-5.614086571	4.690238365	1.769704692
6	-5.962938678	3.854249097	0.675615898
6	-5.171350075	2.779339754	0.288741133
6	-4.006811551	2.498366764	1.066135978
6	1.262045536	2.080179010	2.148995572
1	0.884617383	1.811340432	4.823606711
1	-0.343265948	1.663254527	6.938956796

1	-2.493084182	1.749822795	8.212147093
1	-4.634801257	2.303165051	7.013015177
1	-4.619793299	2.817490176	4.586872802
1	-3.097361598	5.159001367	4.093739965
1	-4.503310026	7.124890908	4.641701088
1	-6.646822699	7.547910964	3.395294334
1	-7.329347246	6.012320543	1.543303078
1	-6.876635613	4.081732729	0.105717839
6	-5.486901177	2.006926538	-0.937302043
6	1.689239079	2.961639788	1.131753548
6	2.860118344	2.706623630	0.408464735
6	3.629219166	1.566088344	0.692496017
6	3.229669991	0.694068140	1.719293788
6	2.055792327	0.946388200	2.432352813
6	-5.811451605	2.700143695	-2.125301513
6	-6.141846271	2.005652859	-3.292674225
6	-6.147730590	0.598930790	-3.294160104
6	-5.806757127	-0.103586947	-2.125406965
6	-5.476094862	0.594755579	-0.959431701
1	1.095071811	3.853450746	0.899497003
1	1.723923607	0.225716314	3.193679844
1	-5.753165088	3.797436607	-2.137564398
1	-5.214350921	0.040090347	-0.050564018
7	-1.918198301	2.334350986	-1.569375602
16	-2.385677135	3.810531546	-2.016244120
8	-2.934120134	3.733303535	-3.398092214
8	-3.099875997	4.620755386	-1.006640865
6	-0.728341443	4.710209123	-2.263681236
9	-0.155982786	4.315017831	-3.421571863
9	-0.945115443	6.027182989	-2.321438645
1	2.266718503	0.521722777	-6.890682519
1	2.185928478	3.217830041	-3.498463526
1	3.823023473	-0.202680751	1.945143603
1	3.176023455	3.396553982	-0.386852078
1	-6.373401022	2.560225237	-4.213832373
1	-5.794364117	-1.202018138	-2.132394581
1	-4.805658880	-2.393609697	1.040124749
1	-3.731880121	0.116215403	4.407643893
6	-3.180301401	-1.474585316	-5.381383840
6	-0.941325495	1.047382112	-5.874785185
6	-1.088437394	1.124442680	-4.485766939
6	-2.961851932	0.607993836	-4.230427050
1	-0.689418770	0.060181057	-6.299283540
1	-2.993785173	1.096203588	-3.239193642
1	-0.669683501	0.327304039	-3.844303487
1	-1.156522553	2.116184995	-4.018675188
6	-1.195809302	2.100416836	-6.822601396
6	-0.956055614	1.846091934	-8.201175438
6	-1.670064317	3.383384026	-6.426253405
6	-1.155522331	2.845806914	-9.153957970
1	-0.592143873	0.851862394	-8.503225942
6	-1.880218536	4.371560925	-7.388627942
1	-1.888824992	3.594660901	-5.368312530
6	-1.617803601	4.110416127	-8.746984095
1	-0.953322610	2.646945501	-10.216659061
1	-2.249863766	5.360395042	-7.079653466
1	-1.776554870	4.900973721	-9.496425638
8	-0.876735121	-0.949958273	-2.300807036
1	-3.851358098	-2.329474001	-5.144598075
8	-1.917799466	-1.874041361	-5.763340684

8	-3.149086014	-0.708125553	-4.152806823
1	-3.606500519	-0.852018146	-6.195724613
6	-1.314170861	-2.838849167	-4.886601581
1	-1.965907543	-3.737572537	-4.796254580
1	-0.356820021	-3.132957770	-5.355627032
1	-1.124496677	-2.405473772	-3.882128196
9	0.129568549	4.447705949	-1.264338275
6	-6.531419440	-0.129542244	-4.553123926
6	-5.846066200	-0.631150102	2.886937636
6	2.212298570	3.185928453	-6.223559345
6	4.876108269	1.244374190	-0.090080188
9	-5.836393230	0.341233100	-5.638877749
9	-7.841407022	0.019637122	-4.855074847
9	-6.284277761	-1.462361938	-4.483810018
9	-6.646436605	-1.089204477	1.891991107
9	-6.386853230	-1.041294605	4.061055423
9	-5.935956026	0.728653375	2.875044934
9	5.041844667	2.061932598	-1.157633103
9	5.993756415	1.343279013	0.672441512
9	4.846765496	-0.033219726	-0.565469212
9	1.922980613	2.834045801	-7.503732680
9	1.290292174	4.105943740	-5.839617708
9	3.408720423	3.823686356	-6.249632691
1	-3.468708274	1.138792599	-5.059631103

Pdt Complex of TS-2

15	-0.225277632	-0.680068078	-0.972679886
8	1.424072934	-0.421800409	-0.813249345
8	-0.322946432	-2.146897613	-0.182876001
6	2.269932429	-1.432871618	-1.213977968
6	2.953455641	-1.302545907	-2.469223410
6	3.824488687	-2.328276617	-2.831523459
6	3.988272189	-3.500061593	-2.045662681
6	4.841246129	-4.562779105	-2.469302561
6	4.946965509	-5.729804176	-1.729638571
6	4.189999008	-5.881116397	-0.537353238
6	3.367472927	-4.858163472	-0.087678376
6	3.258960529	-3.632184791	-0.808629614
6	2.433591980	-2.538470610	-0.369455765
6	1.743065288	-2.542798130	0.950191087
6	2.470936236	-2.688785815	2.184773797
6	3.893363222	-2.801079455	2.240878365
6	4.555134185	-2.926388178	3.454479786
6	3.830130506	-2.947017761	4.675363324
6	2.451315172	-2.809463705	4.658625667
6	1.741725104	-2.658938924	3.429391795
6	0.339157605	-2.427952689	3.414482133
6	-0.368167560	-2.223113074	2.232074687
6	0.362873934	-2.310624377	1.002447038
6	2.738983662	-0.137513499	-3.363726364
1	4.412075351	-2.238109041	-3.757730276
1	5.406077294	-4.440620459	-3.406784867
1	5.604156215	-6.544156514	-2.069781134
1	4.253503517	-6.819487958	0.034072674
1	2.781924773	-4.987880932	0.832746254
1	4.469169718	-2.762605119	1.306550995
1	5.652802373	-3.002468580	3.469049360
1	4.365456567	-3.054508847	5.630622749
1	1.877917144	-2.798302155	5.598784033
1	-0.199042455	-2.399403558	4.374211789

6	-1.809451905	-1.869848451	2.263874409
6	2.495774356	1.164719381	-2.866473656
6	2.300131565	2.243965892	-3.732047023
6	2.347746900	2.056824816	-5.123964541
6	2.608621408	0.774593244	-5.640396343
6	2.795912994	-0.304667699	-4.768531655
6	-2.273219179	-0.970299179	3.250303942
6	-3.619374126	-0.608419313	3.321434461
6	-4.536260319	-1.129994495	2.393528119
6	-4.094719936	-2.029549904	1.409489135
6	-2.744764724	-2.394924373	1.344513152
1	2.454983732	1.335861407	-1.785781023
1	2.980183408	-1.306228719	-5.185520729
1	-1.557529526	-0.513035638	3.948313500
1	-2.409368700	-3.093558245	0.567681092
7	-0.854783023	0.419505446	0.001976848
15	-1.644365151	1.776167289	-0.128092666
8	-1.042237636	2.919414868	0.909749969
8	-3.115351669	1.477462032	0.585546313
6	-1.096932490	2.649366211	2.268795247
6	0.101734820	2.210036131	2.916302273
6	0.027769256	1.940244969	4.280759255
6	-1.189510204	2.047566481	5.007942238
6	-1.254883242	1.709358600	6.393089814
6	-2.458109941	1.742343816	7.079934019
6	-3.650093391	2.108435638	6.399868896
6	-3.618132273	2.460909024	5.057952443
6	-2.392006957	2.462122369	4.327031698
6	-2.319094472	2.801230822	2.929239938
6	-3.516349039	3.242178324	2.162512449
6	-4.279565327	4.400284185	2.548007004
6	-3.890270924	5.263397834	3.614663804
6	-4.658573001	6.366330957	3.958831513
6	-5.854977005	6.662766613	3.254045527
6	-6.245728579	5.860946668	2.193606502
6	-5.469072602	4.730074224	1.803240330
6	-5.828092537	3.944435973	0.676025727
6	-5.054478388	2.872256335	0.245308140
6	-3.896420151	2.541039398	1.014767442
6	1.364219610	2.006051976	2.163364040
1	0.936599741	1.626140210	4.816430851
1	-0.326937840	1.406382910	6.902885741
1	-2.495219653	1.472385123	8.145985039
1	-4.609698996	2.105372100	6.938333900
1	-4.548719315	2.720400901	4.535506369
1	-2.960687811	5.052828063	4.160576588
1	-4.332763626	7.020859485	4.781385113
1	-6.459135193	7.536669731	3.540553500
1	-7.157813852	6.092802835	1.621962785
1	-6.739416767	4.207707904	0.118032622
6	-5.398377352	2.150469562	-1.004067341
6	1.809117734	2.914861691	1.176762673
6	3.001677315	2.687320090	0.480310845
6	3.772183435	1.544703357	0.754755803
6	3.350087721	0.642511374	1.744993107
6	2.157307595	0.869219823	2.434717111
6	-5.779730440	2.891945650	-2.145813649
6	-6.148342768	2.248362093	-3.329966096
6	-6.131839127	0.843520661	-3.400684290
6	-5.737916635	0.092904433	-2.280258004

6	-5.372374326	0.740813808	-1.094980724
1	1.212155466	3.805503560	0.945070408
1	1.810861561	0.126152468	3.167285396
1	-5.733566889	3.989368720	-2.111539891
1	-5.060407919	0.146776102	-0.228490820
7	-1.743381766	2.437930144	-1.591894117
16	-2.227826098	3.920413526	-2.002615507
8	-2.867922916	3.879744508	-3.343719374
8	-2.854285587	4.728711464	-0.934247168
6	-0.564640749	4.772114911	-2.346787848
9	-0.089377548	4.389225548	-3.551532183
9	-0.734038048	6.096748392	-2.354398595
1	2.669163603	0.626305439	-6.728832378
1	2.107278709	3.243346267	-3.320740526
1	3.938779933	-0.260097084	1.958612801
1	3.331804121	3.399070613	-0.289735899
1	-6.418376782	2.842076908	-4.215603029
1	-5.703515175	-1.003337493	-2.341907644
1	-4.813200218	-2.445000142	0.688697111
1	-3.952511558	0.103782108	4.089010538
6	-3.074280988	-0.773892804	-5.670178958
6	-1.162794733	0.868690271	-5.925601682
6	-1.459499534	1.228000349	-4.482971029
6	-2.887867879	0.897482706	-4.071478010
1	-3.003476336	1.009827985	-2.983301748
1	-0.767004016	0.696249282	-3.803569311
1	-1.279293696	2.307114442	-4.336107847
6	-1.846600958	1.657021202	-7.014167121
6	-1.803629563	1.192740231	-8.346344879
6	-2.474740806	2.887960655	-6.733277895
6	-2.397666580	1.931558912	-9.377012771
1	-1.306350355	0.237381616	-8.573549360
6	-3.058765264	3.629037899	-7.771679150
1	-2.504930555	3.284599396	-5.707152082
6	-3.028206405	3.153778485	-9.090831881
1	-2.361607511	1.556051910	-10.410748875
1	-3.543387522	4.589149938	-7.540118889
1	-3.491775057	3.737958526	-9.900026729
8	-0.656892667	-0.900433313	-2.394659575
1	-3.298933528	-1.834425926	-5.885241449
8	-1.577310368	-0.617773769	-6.130950894
8	-3.178924112	-0.503701848	-4.368119587
1	-3.574221500	-0.065617761	-6.362535660
6	-0.686745429	-1.569701480	-5.446087957
1	-0.986928689	-2.570458928	-5.804335376
1	0.337040164	-1.325357706	-5.778243676
1	-0.777500469	-1.469960273	-4.343798840
9	0.352873057	4.451582942	-1.414616174
1	-3.645161299	1.523984196	-4.581854542
6	-5.977051139	-0.697930122	2.479488752
6	2.053264926	3.207068326	-6.051731330
6	-6.512866958	0.173687253	-4.692672201
6	5.048613794	1.261291501	0.005774605
9	-6.087429073	0.659911423	2.480239202
9	-6.569185831	-1.132119969	3.620110247
9	-6.717548186	-1.157425393	1.440723503
9	5.174829829	2.026530934	-1.105753725
9	5.111002828	-0.040204551	-0.392849189
9	6.147201026	1.481624068	0.771218263
9	-5.838826429	0.723078913	-5.756417605

9	-7.829081094	0.309792451	-4.976289757
9	-6.232857796	-1.152380092	-4.703080983
9	2.458118351	4.390738299	-5.547199532
9	2.639054176	3.047766239	-7.262820388
9	0.708117959	3.317610120	-6.284662801
1	-0.071652148	0.818995950	-6.109600953

TS-3
Imaginary frequency = –218.51

15	0.253838804	-0.454155665	-0.961983513
8	1.896310758	-0.267044521	-0.685069088
8	0.068537367	-2.002785687	-0.371781025
6	2.767344764	-1.256077453	-1.084121068
6	3.606149452	-1.014353934	-2.223563299
6	4.501633952	-2.020067913	-2.583419239
6	4.553186828	-3.270014912	-1.911892945
6	5.435412045	-4.306525181	-2.340449890
6	5.439076607	-5.542658614	-1.714342600
6	4.547930052	-5.791008911	-0.636499365
6	3.691982210	-4.797434008	-0.183497660
6	3.682320211	-3.505342853	-0.787525345
6	2.825563682	-2.440464094	-0.338384582
6	2.011970474	-2.553519085	0.903139187
6	2.622453815	-2.825652239	2.179561161
6	4.033389354	-2.958515047	2.354197242
6	4.579979119	-3.204632007	3.606029316
6	3.745467437	-3.332388086	4.748000168
6	2.374421048	-3.178156848	4.618360531
6	1.781888264	-2.903946164	3.349150487
6	0.387738123	-2.655160262	3.227322600
6	-0.205440681	-2.325606089	2.010916081
6	0.636060689	-2.303063477	0.851075921
6	3.530566758	0.242696054	-3.009209242
1	5.199564755	-1.850251361	-3.416994114
1	6.106090956	-4.107045097	-3.190818055
1	6.119575492	-6.336115277	-2.057978365
1	4.532613633	-6.782149644	-0.158470438
1	3.002351307	-5.002168664	0.646819844
1	4.693124155	-2.833391945	1.485660386
1	5.671571468	-3.292035924	3.713998068
1	4.190175957	-3.535609633	5.733702546
1	1.716932140	-3.248276948	5.499055344
1	-0.237956685	-2.714170887	4.131007489
6	-1.642765413	-1.961494770	1.947388296
6	3.233208493	1.494479564	-2.420132431
6	3.158715755	2.657224358	-3.194518925
6	3.390474092	2.603952086	-4.578848701
6	3.716029250	1.373812106	-5.180150589
6	3.772758586	0.212530891	-4.405113105
6	-2.194073854	-1.152903791	2.967165045
6	-3.541708956	-0.788703615	2.949982733
6	-4.371011238	-1.216953778	1.899984586
6	-3.842829803	-2.030204938	0.883531456
6	-2.491570938	-2.395670478	0.904954055
1	3.048923292	1.560198015	-1.342557284
1	3.982828364	-0.748453765	-4.896369148
1	-1.543955459	-0.765549026	3.765024273
1	-2.086606997	-3.023924332	0.101286700
7	-0.385154424	0.541678701	0.119557104

15	-1.236279475	1.861645793	0.018794157
8	-0.800302892	2.933625221	1.202968111
8	-2.753275401	1.449023179	0.549791281
6	-1.035589665	2.571484590	2.520278792
6	0.072460705	2.091738764	3.288967473
6	-0.176018758	1.729119261	4.610342342
6	-1.480512533	1.779573625	5.174743119
6	-1.723079619	1.347411165	6.513252078
6	-3.007522283	1.319201307	7.032778319
6	-4.105668075	1.714559656	6.223486164
6	-3.902582167	2.158798796	4.924486349
6	-2.589829977	2.227724319	4.367798910
6	-2.338921633	2.662693335	3.018057513
6	-3.436340182	3.118483342	2.120366199
6	-4.288092195	4.229225726	2.449904246
6	-4.089031465	5.041388148	3.604841417
6	-4.941642826	6.098040923	3.890389881
6	-6.035930802	6.395375263	3.035546630
6	-6.239956854	5.642853114	1.890101673
6	-5.371293891	4.561159485	1.557800876
6	-5.535871720	3.830011600	0.351629322
6	-4.668852847	2.806733811	-0.020964375
6	-3.627935536	2.467495462	0.899190538
6	1.416101899	1.920940681	2.681782583
1	0.658206869	1.380209355	5.238091448
1	-0.865436226	1.020811182	7.121960940
1	-3.180766232	0.976518368	8.063930216
1	-5.128468308	1.661368184	6.625789559
1	-4.761169119	2.438034884	4.299306153
1	-3.239553670	4.827371195	4.267647971
1	-4.763784949	6.714794884	4.784324603
1	-6.709214950	7.231714884	3.276478349
1	-7.070270417	5.877571136	1.206030630
1	-6.371843195	4.096191013	-0.312675995
6	-4.791582081	2.146431442	-1.343793559
6	1.985012175	2.887174208	1.822409000
6	3.239174140	2.674992838	1.238263796
6	3.950196379	1.492759153	1.503771747
6	3.406314216	0.533409678	2.374310328
6	2.151352834	0.744816963	2.949440029
6	-5.141455915	2.918003754	-2.474945571
6	-5.282944625	2.329705509	-3.735086642
6	-5.047597154	0.954408890	-3.899108756
6	-4.684712607	0.174699263	-2.787288622
6	-4.565058963	0.762215084	-1.525023505
1	1.435430895	3.809696853	1.598099496
1	1.710908313	-0.039765951	3.581479687
1	-5.258941803	4.005529994	-2.368797546
1	-4.283290405	0.140653563	-0.668139414
7	-1.247739962	2.649130525	-1.391144480
16	-1.631178624	4.193167440	-1.637011259
8	-2.214324507	4.345995238	-2.996303269
8	-2.252121399	4.920752660	-0.511368247
6	0.092033677	4.968326808	-1.821085542
9	0.922460689	4.553152639	-0.847968786
9	0.637578041	4.617991227	-3.011323827
1	3.910891269	1.323312105	-6.261845819
1	2.913396570	3.614395417	-2.717185401
1	3.950772962	-0.398157973	2.581045288
1	-5.549948150	2.947453209	-4.604433596

1	-4.487739713	-0.900080613	-2.910636782
1	-4.494297476	-2.374672055	0.067768297
1	-3.942732207	-0.143395936	3.743804201
6	-2.387211068	3.054157502	-5.739330826
6	0.313784752	0.939949736	-6.426654098
6	0.202670891	0.352573382	-5.177913561
6	-0.917788719	1.780108952	-4.296148711
1	-0.892126373	1.127729435	-3.398136586
1	-0.233633617	2.643212489	-4.346657202
1	-0.502836694	-0.469150505	-4.984311509
1	1.049705367	0.387377019	-4.477809176
8	-0.125709585	-0.454859001	-2.415401245
1	-2.985544460	3.733656814	-5.098726943
8	-1.247501811	3.632158687	-6.233883394
8	-2.095118331	1.894372792	-4.856399060
1	-2.994323888	2.595578744	-6.543026582
6	-0.982986998	4.994408587	-5.863614176
1	-1.290523707	5.192613720	-4.818070621
1	0.106911520	5.143579431	-5.967646544
1	-1.513752002	5.686254325	-6.553196154
9	-0.003386088	6.300492011	-1.777592517
6	-5.225372892	0.307575789	-5.244550559
6	-5.814207842	-0.783004790	1.888450875
6	3.247445996	3.829243847	-5.439620531
9	-6.467046626	-0.211357347	-5.406224720
9	-4.350562043	-0.718202087	-5.444121130
9	-5.040033965	1.189889903	-6.269232000
9	2.105191025	3.757229846	-6.211390164
9	3.152360705	4.968131912	-4.725877371
9	4.272703066	3.965310412	-6.311783671
9	-6.531049669	-1.379747900	2.874226234
9	-6.428967949	-1.068327994	0.714991341
9	-5.929710353	0.559693485	2.086177223
6	-0.668772161	0.867871831	-7.482611554
6	-0.504265785	1.690015271	-8.629044596
6	-1.836749278	0.066905712	-7.374300709
6	-1.473787101	1.710093804	-9.633417020
1	0.388126402	2.330216490	-8.698810491
6	-2.809935993	0.099271700	-8.375706826
1	-1.996792318	-0.560929277	-6.487892980
6	-2.629855796	0.917622005	-9.505631328
1	-1.341281083	2.353467978	-10.515862459
1	-3.720805807	-0.506067556	-8.267266551
1	-3.399094421	0.940397595	-10.292650412
1	1.155026804	1.631976978	-6.595754831
1	3.665623723	3.430813180	0.563522529
6	5.292573236	1.226921050	0.872961492
9	5.574495778	2.097311203	-0.127282654
9	5.353300387	-0.026796273	0.342036445
9	6.302912498	1.310551115	1.774764466

9. Copies of NMR spectra

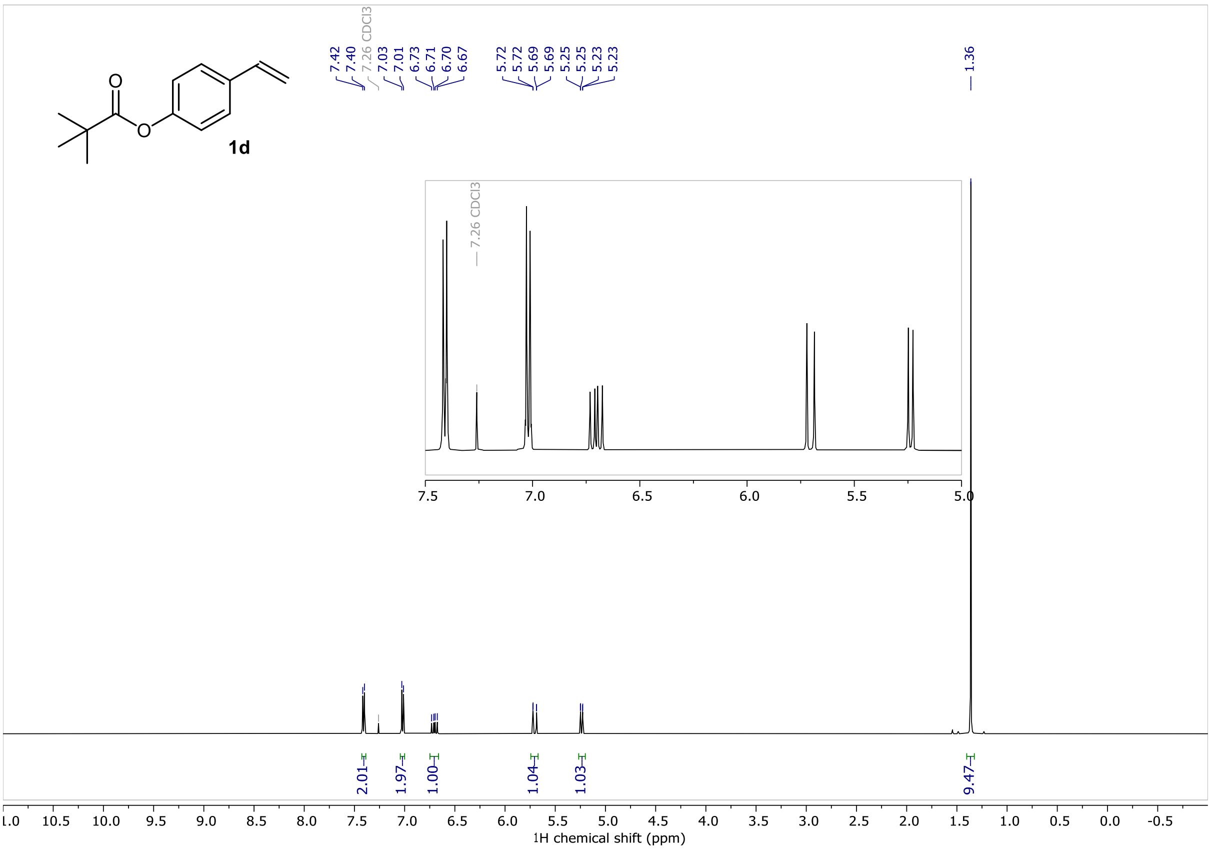

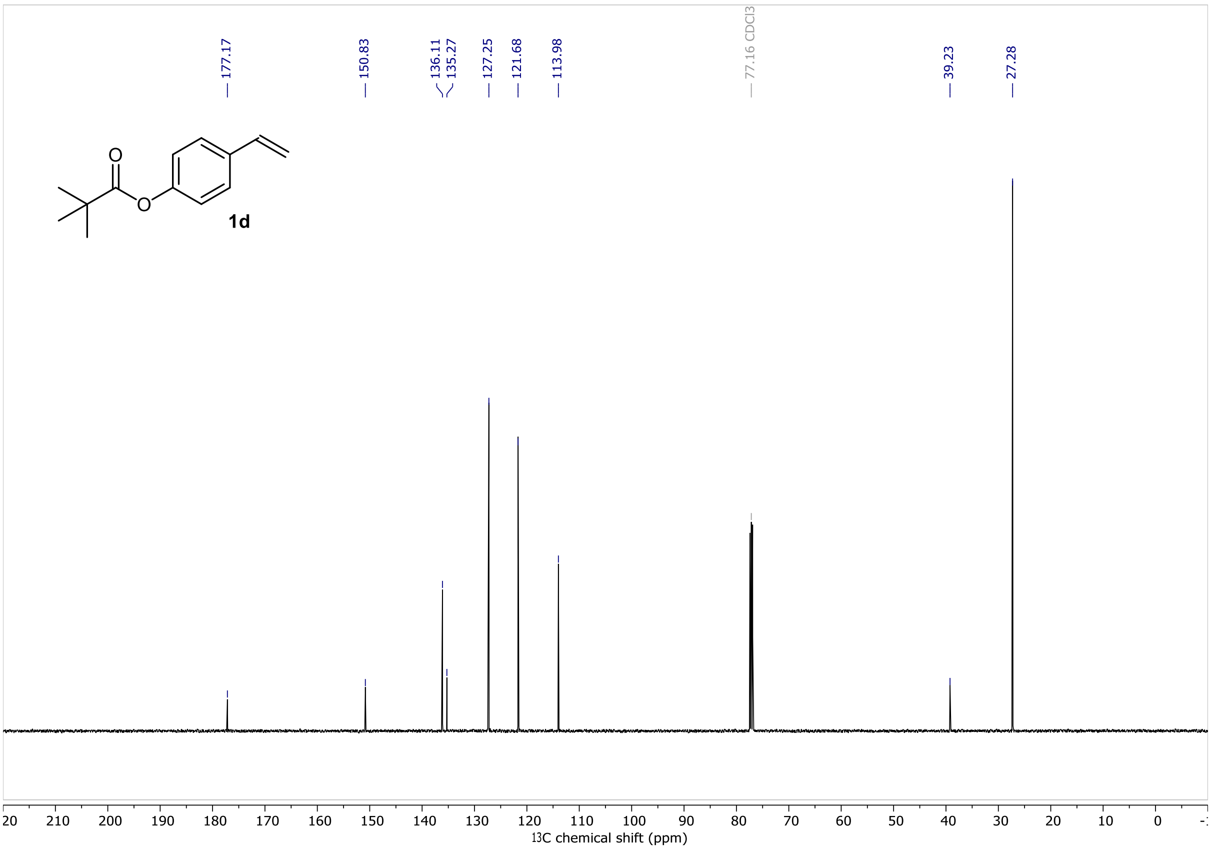

NMR spectra for compound **1d**: ^{1}H (501 MHz) and ^{13}C (126 MHz), in $CDCl_3$.

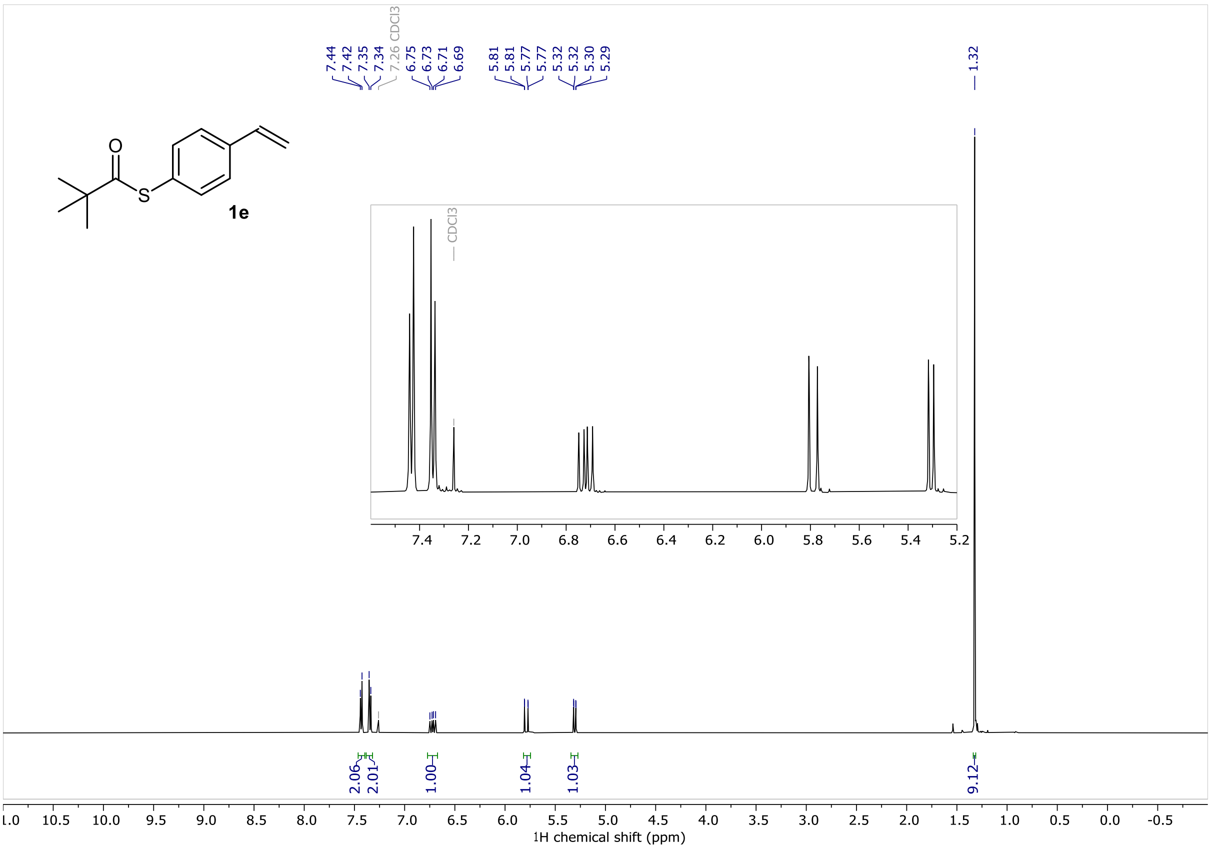

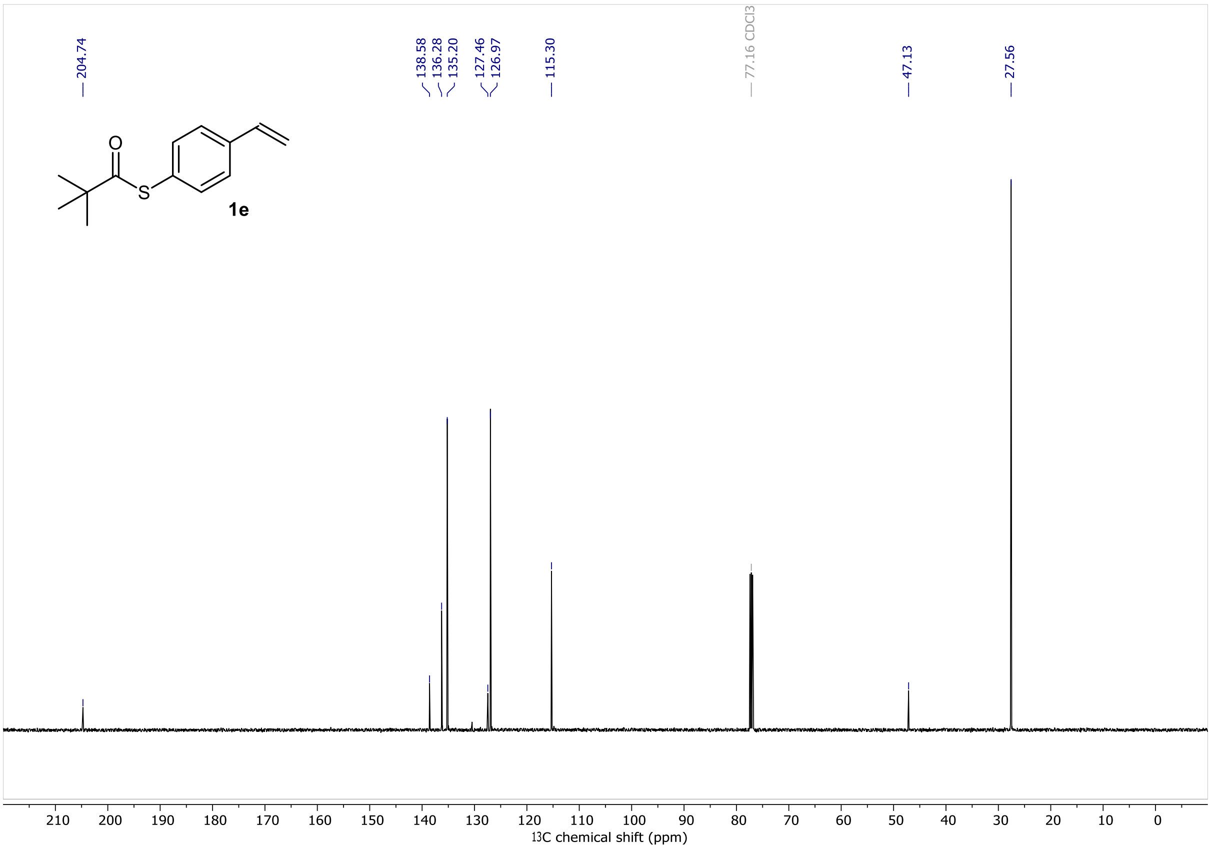

NMR spectra for compound **1e**: ^{1}H (501 MHz) and ^{13}C (126 MHz), in $CDCl_3$.

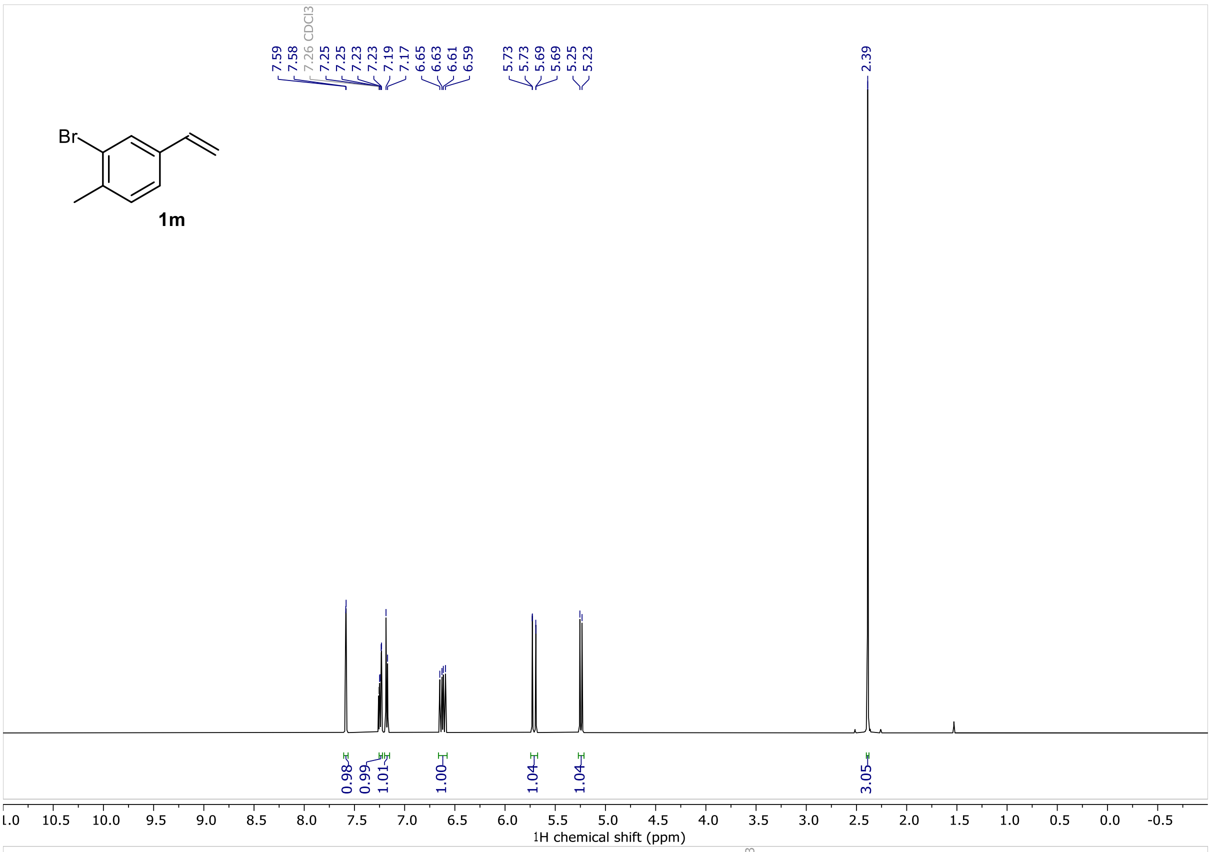

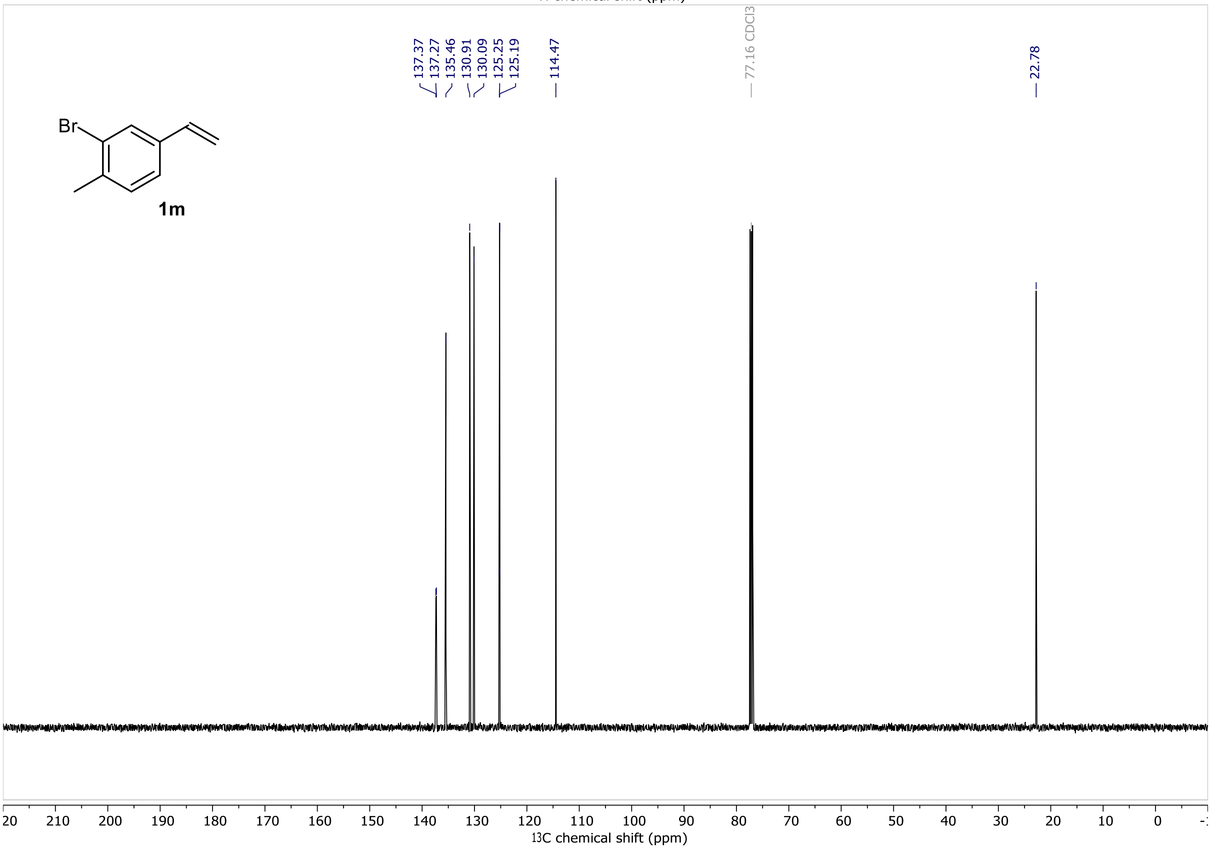

NMR spectra for compound **1m**: ^{1}H (501 MHz) and ^{13}C (126 MHz), in $CDCl_3$.

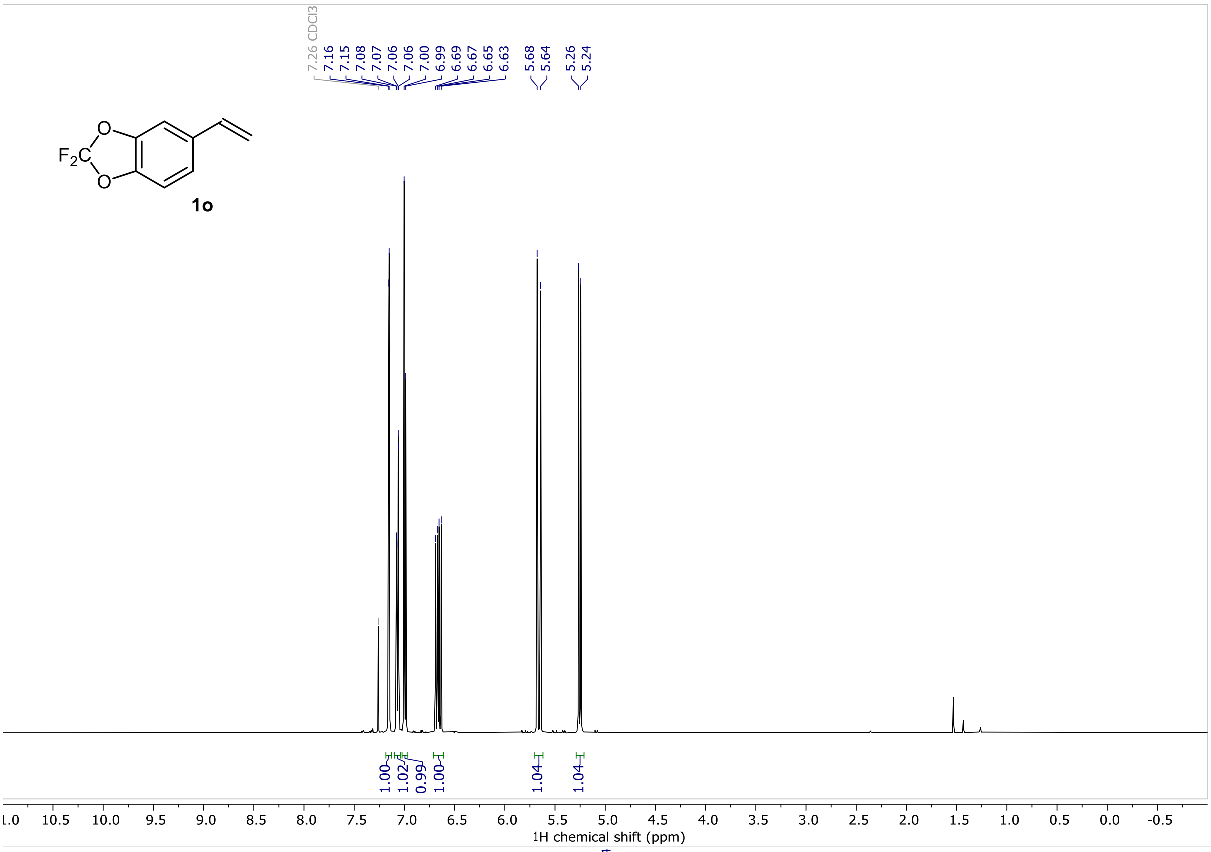

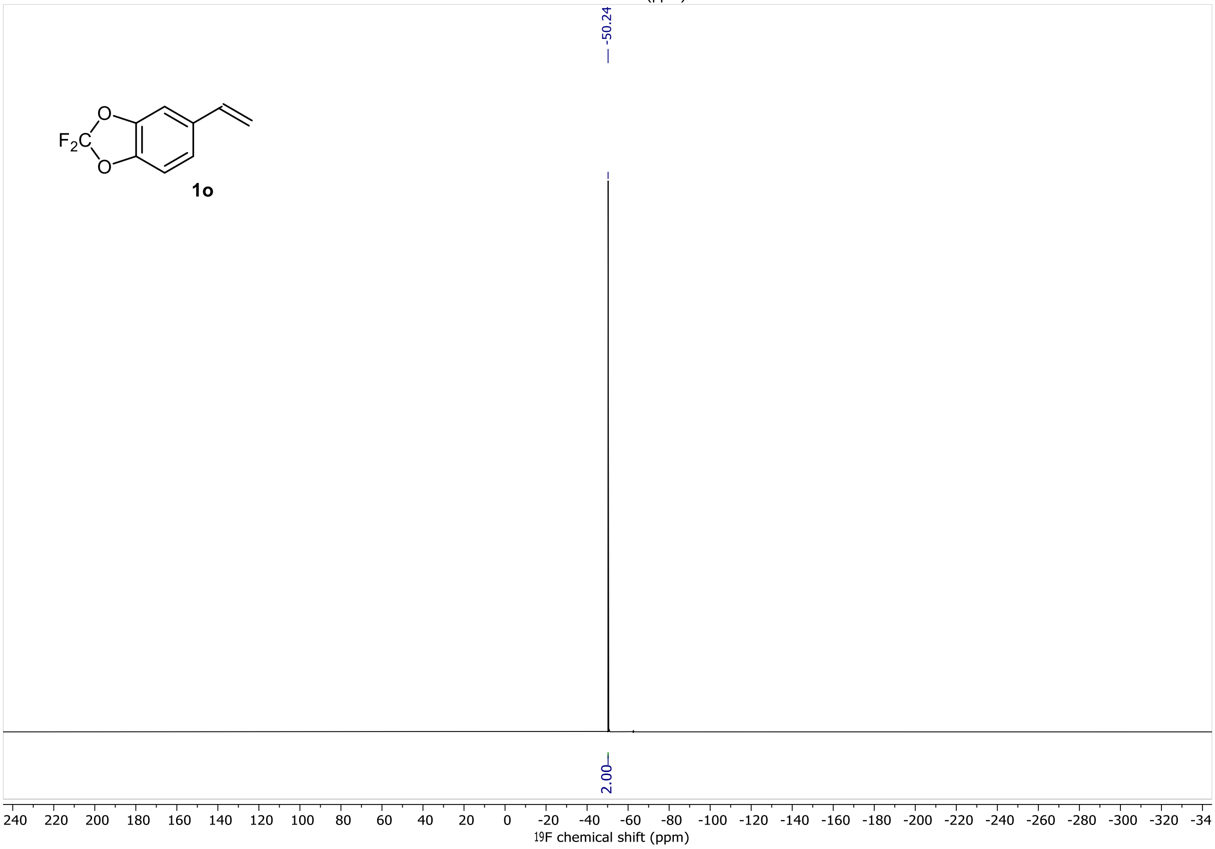

NMR spectra for compound **1o**: ^{1}H (501 MHz) and ^{19}F (471 MHz), in $CDCl_3$.

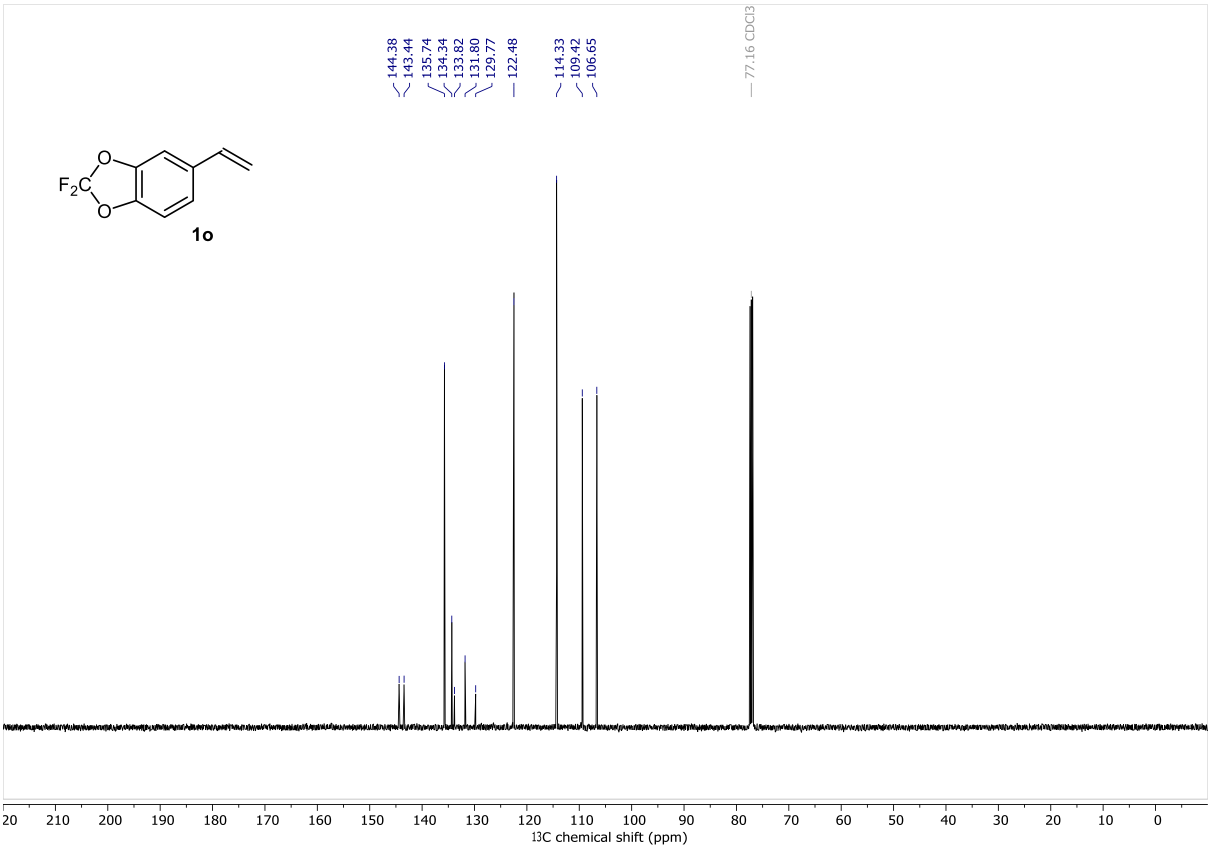

NMR spectra for compound **1o** *(continuation)*: ^{13}C (126 MHz), in $CDCl_3$.

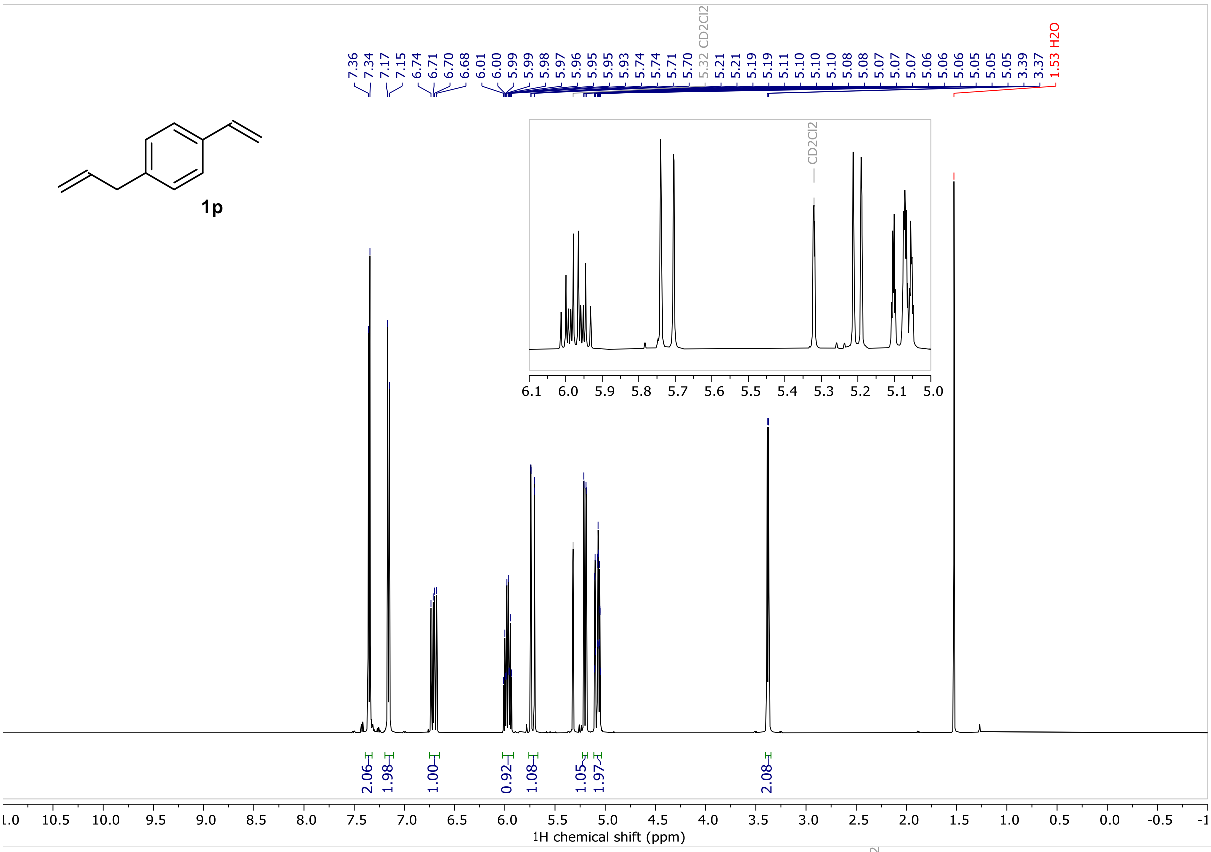

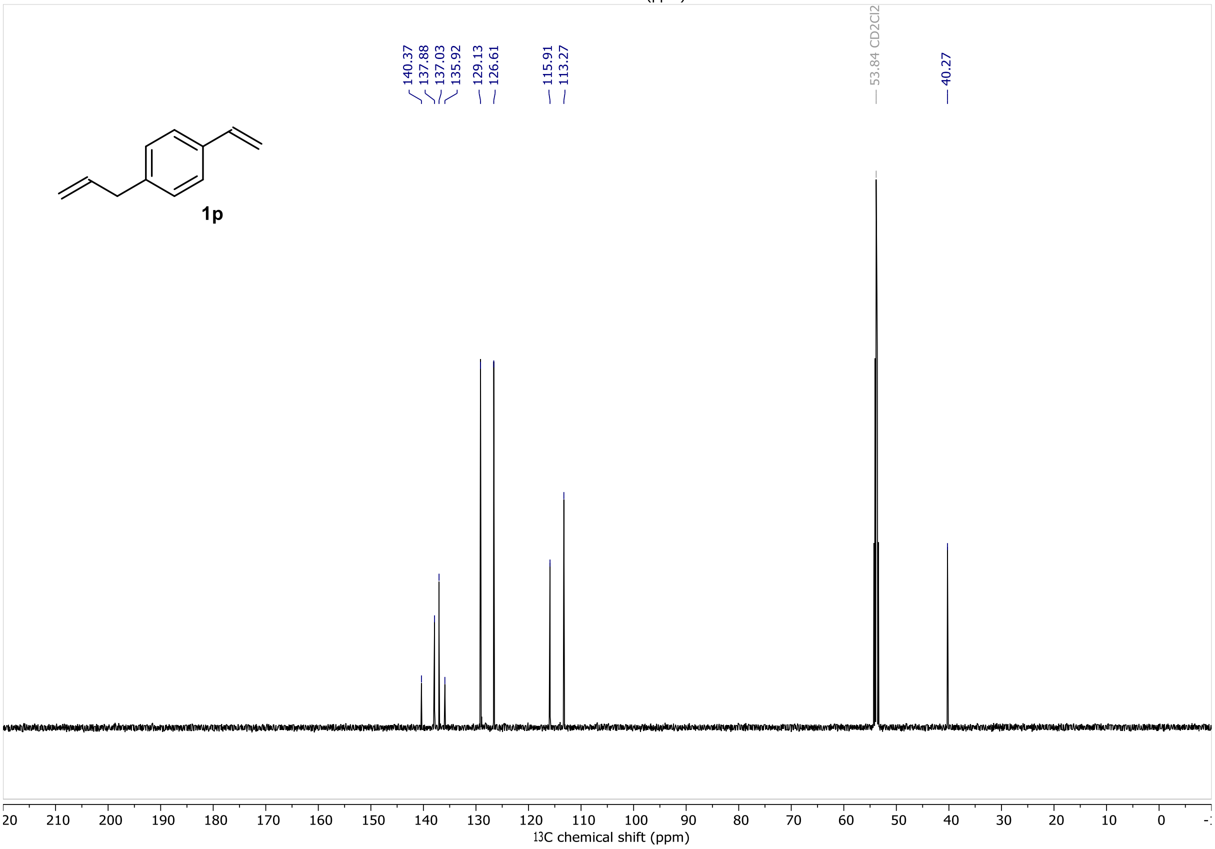

NMR spectra for compound **1p**: ^{1}H (501 MHz) and ^{13}C (126 MHz), in CD_2Cl_2

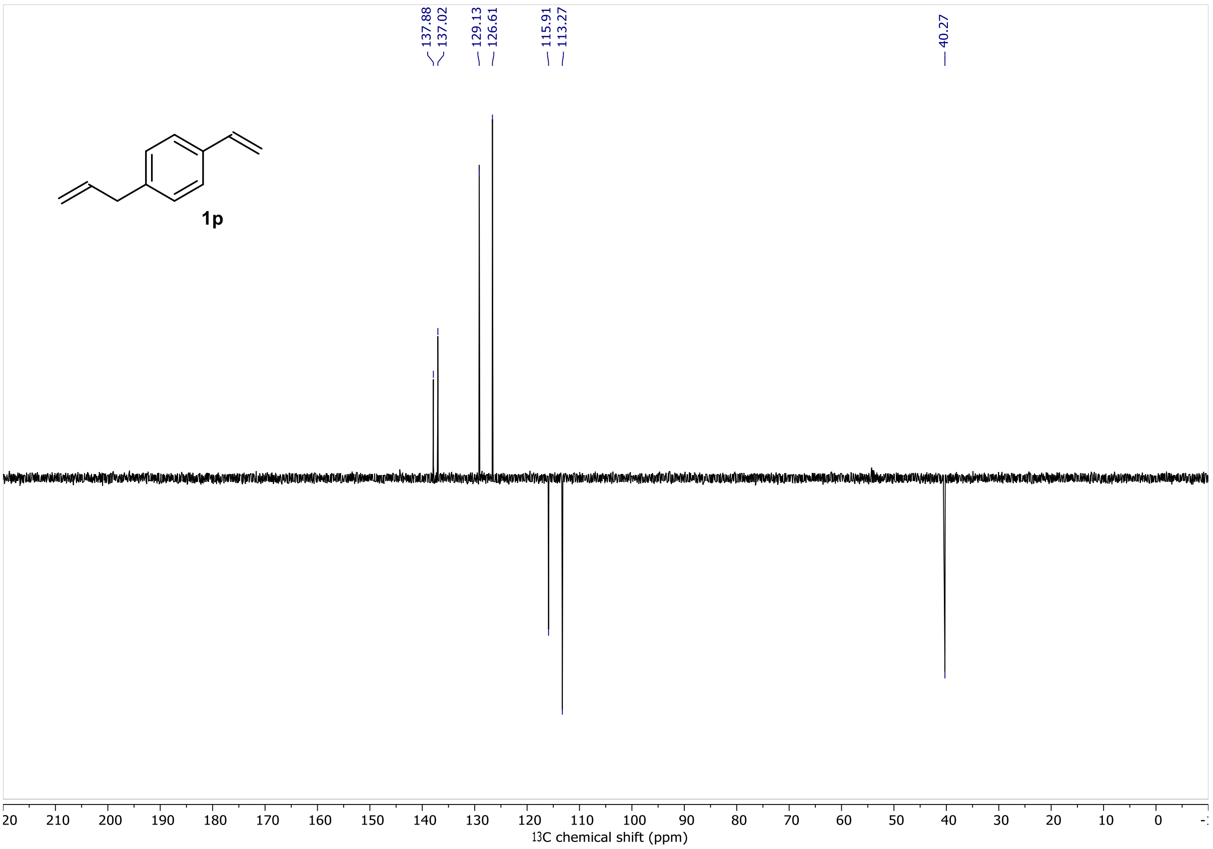

NMR spectra for compound **1p** *(continuation)*: DEPT-135 (126 MHz), in CD_2Cl_2.

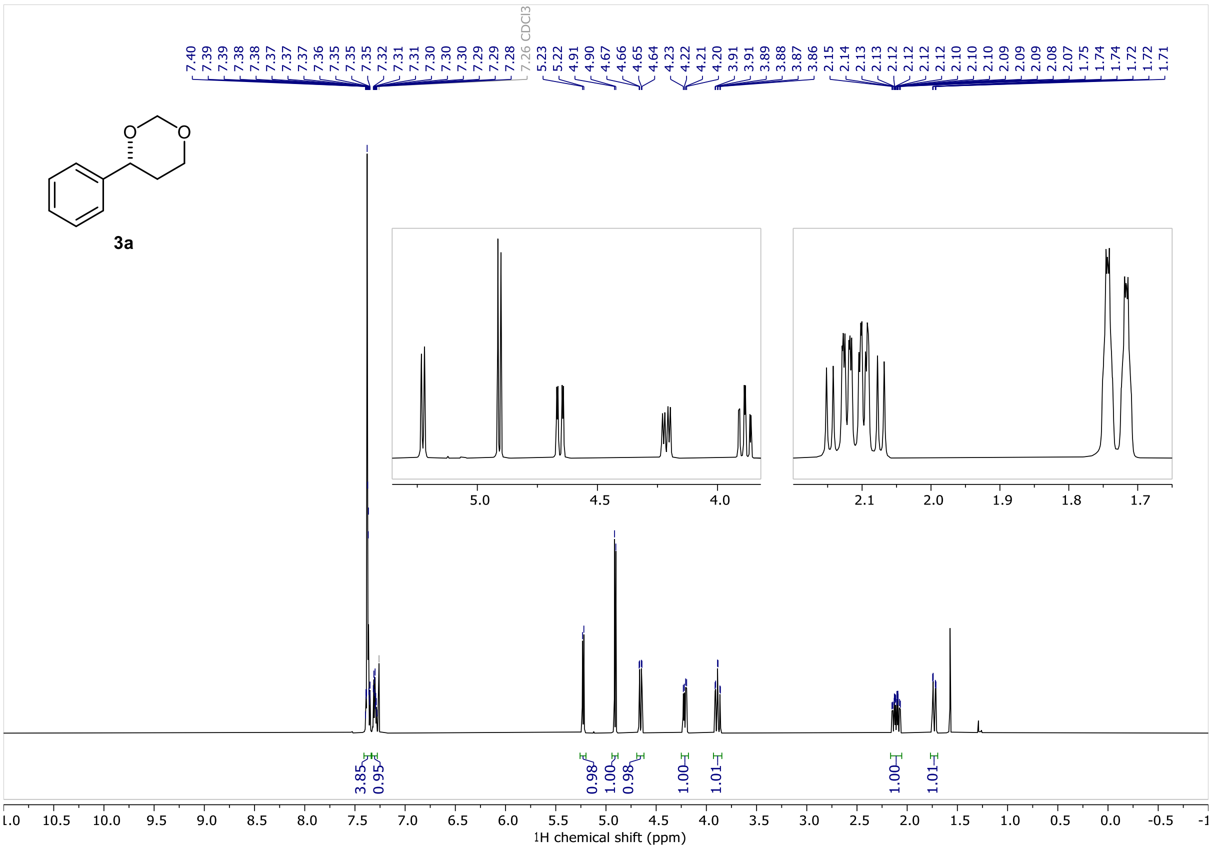

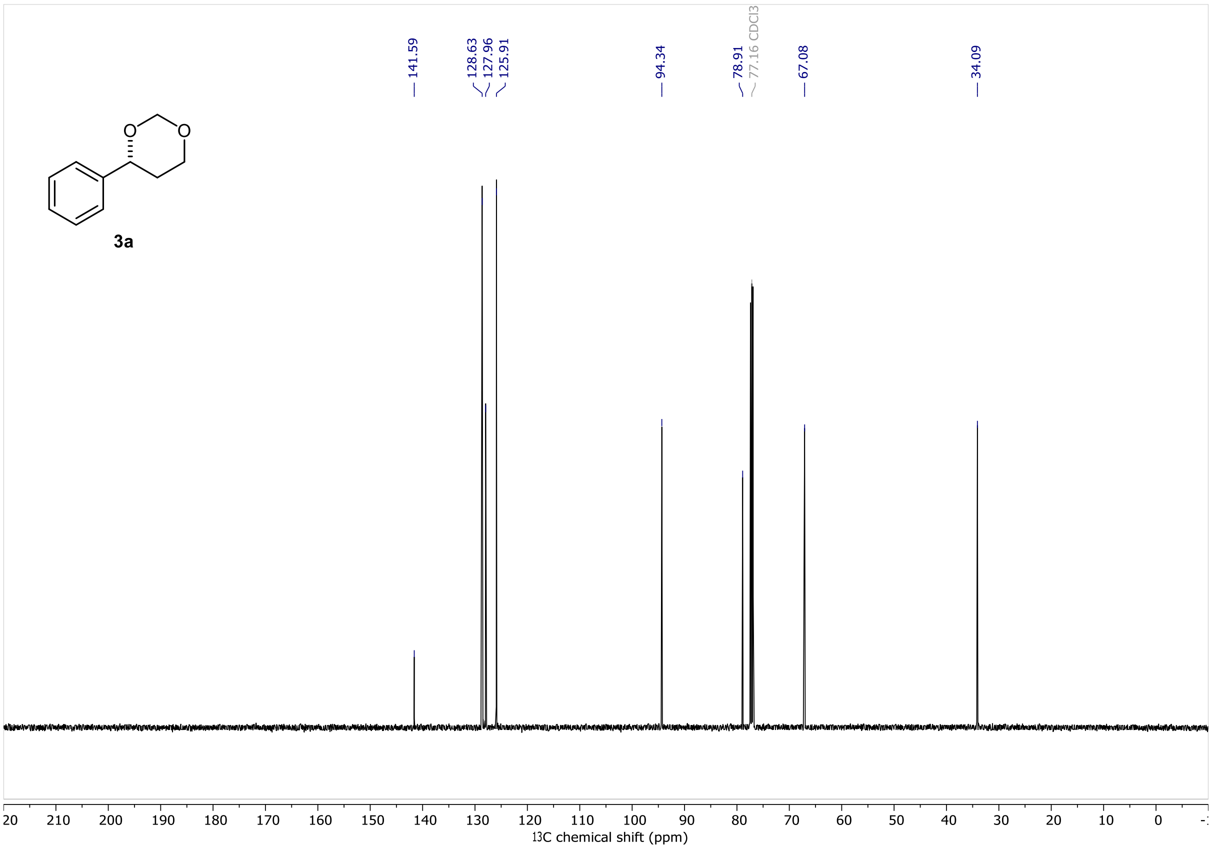

NMR spectra for compound **3a**: ^{1}H (501 MHz) and ^{13}C (126 MHz), in $CDCl_3$.

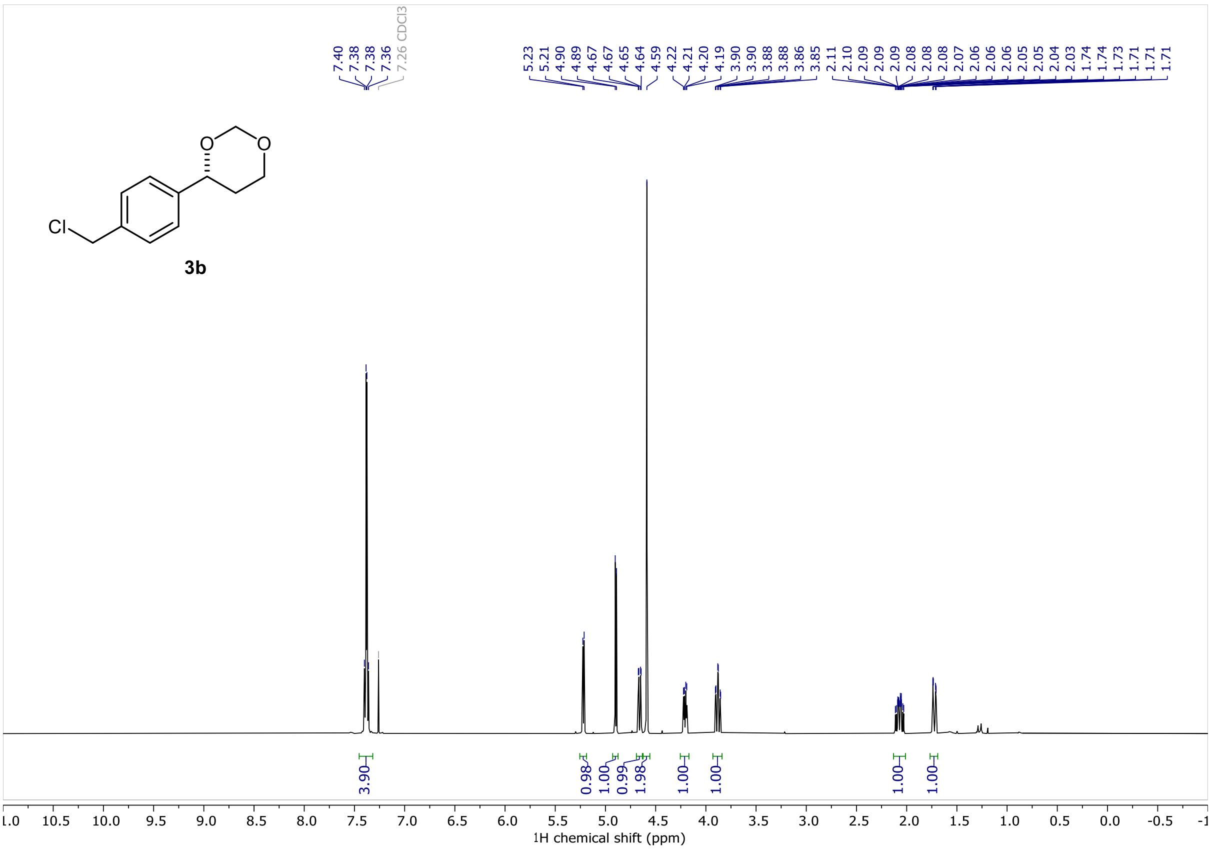

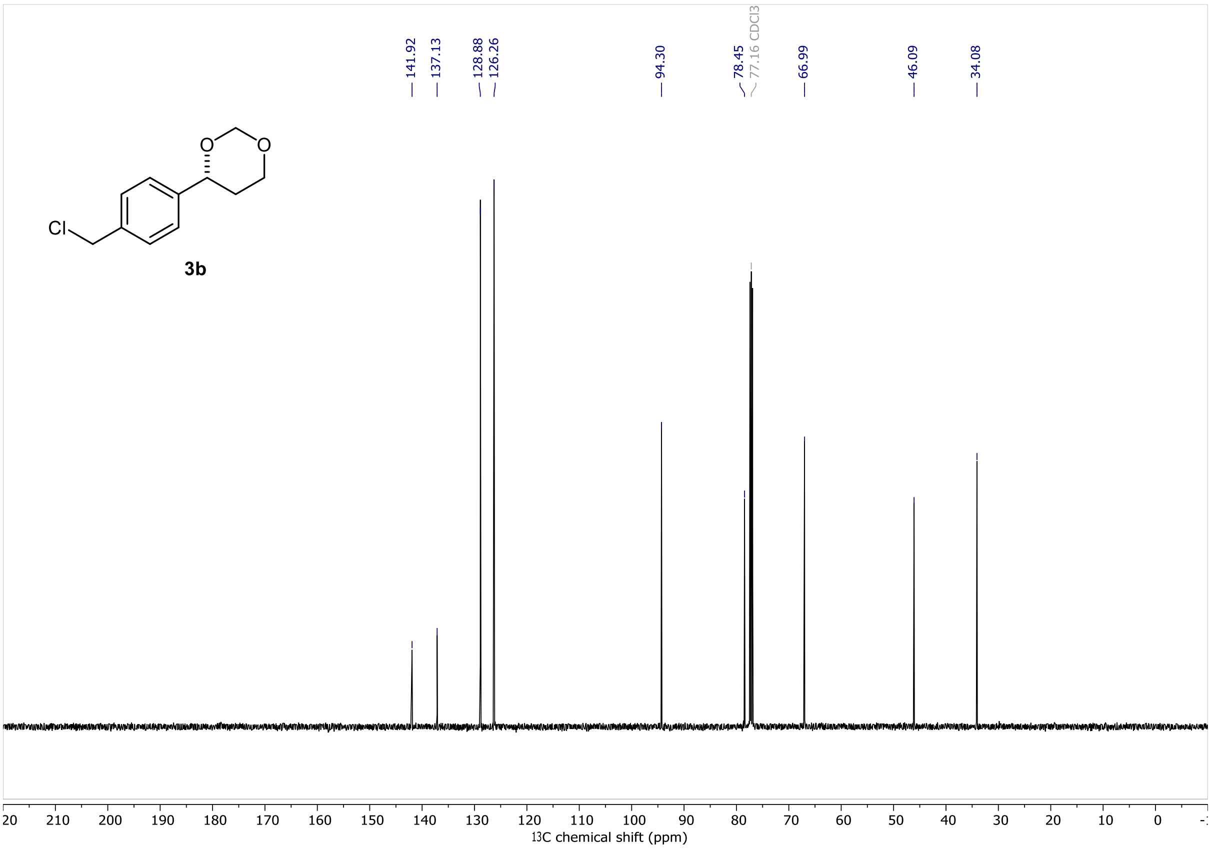

NMR spectra for compound **3b**: ^{1}H (501 MHz) and ^{13}C (126 MHz), in $CDCl_3$.

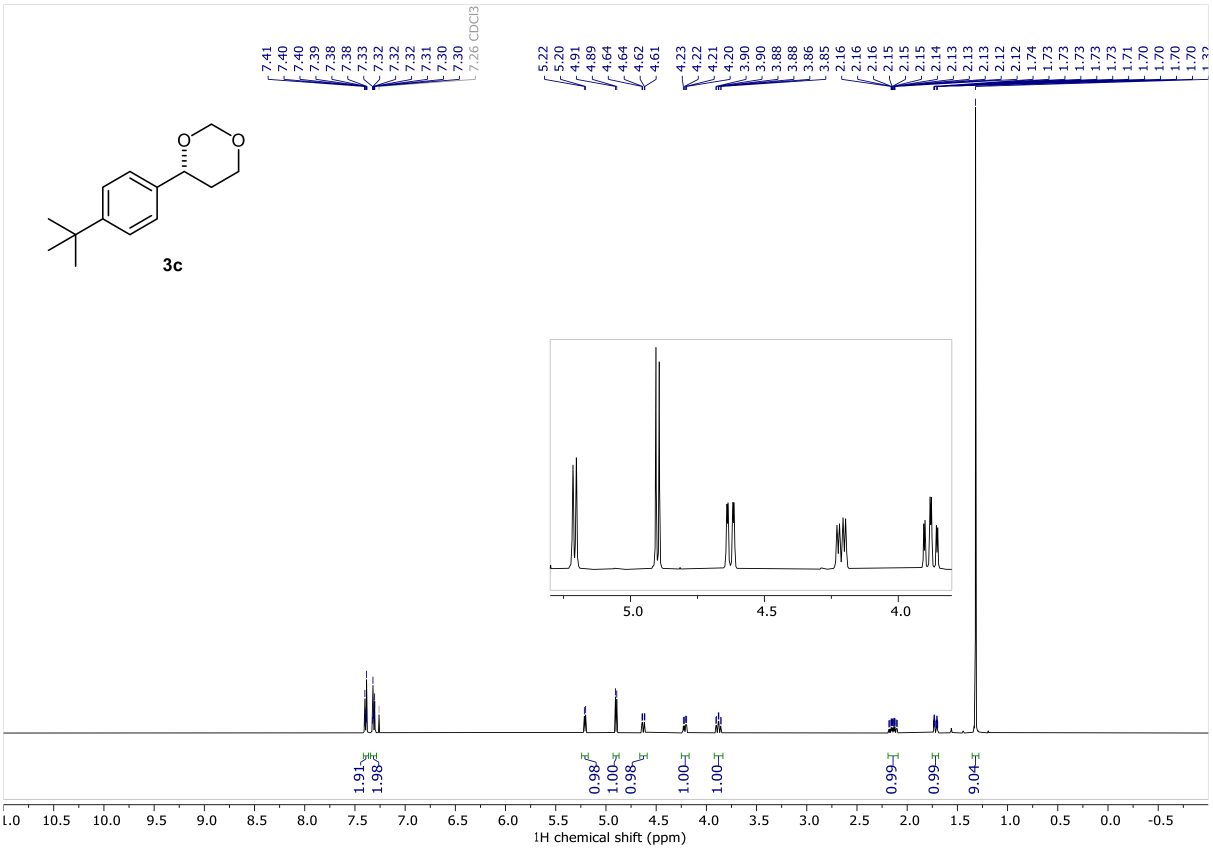

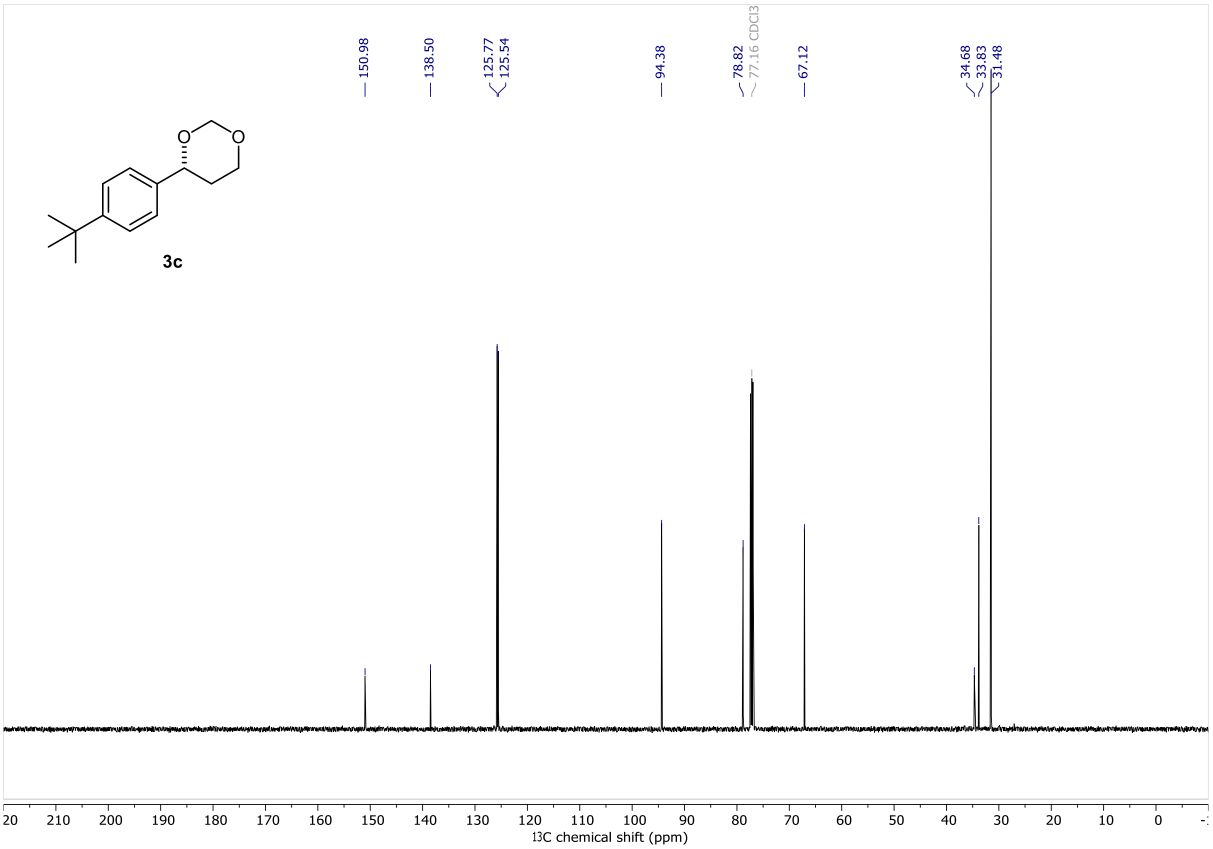

NMR spectra for compound **3c**: ^{1}H (501 MHz) and ^{13}C (126 MHz), in $CDCl_3$.

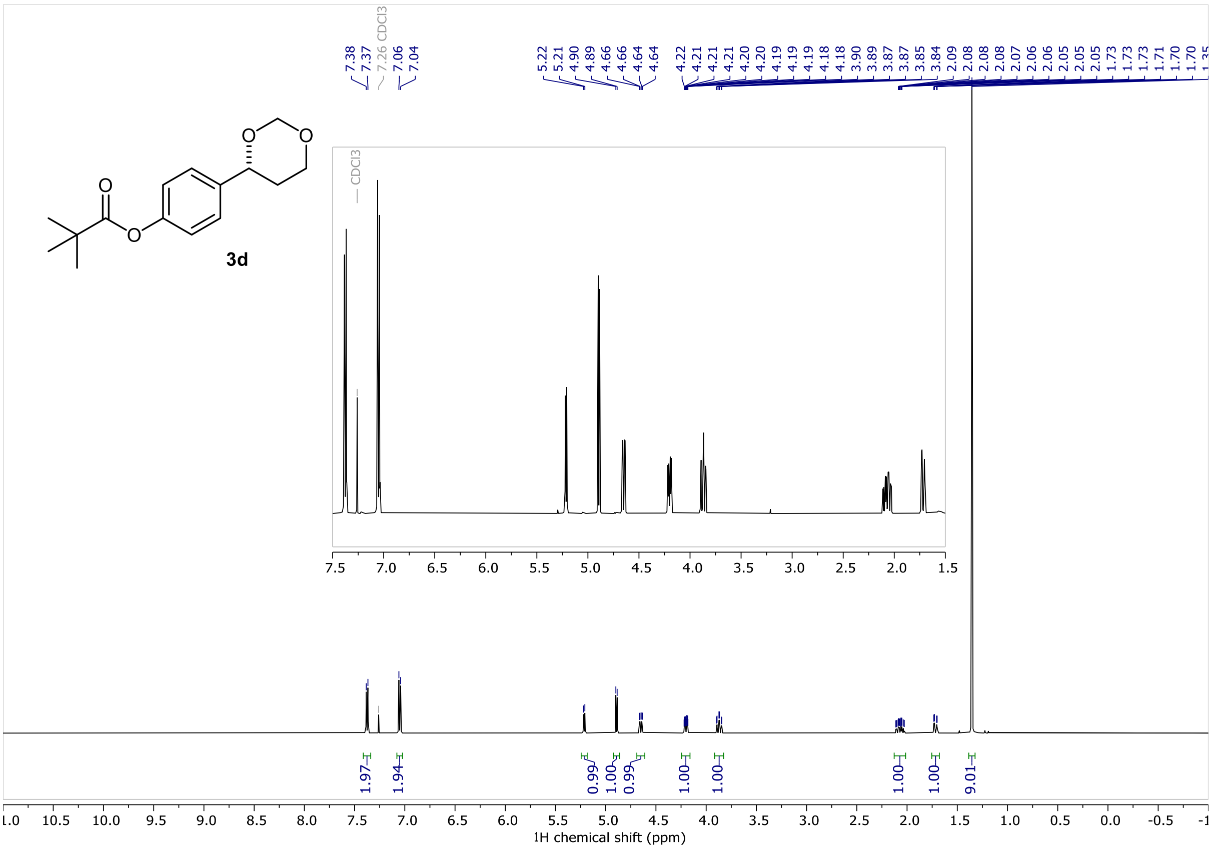

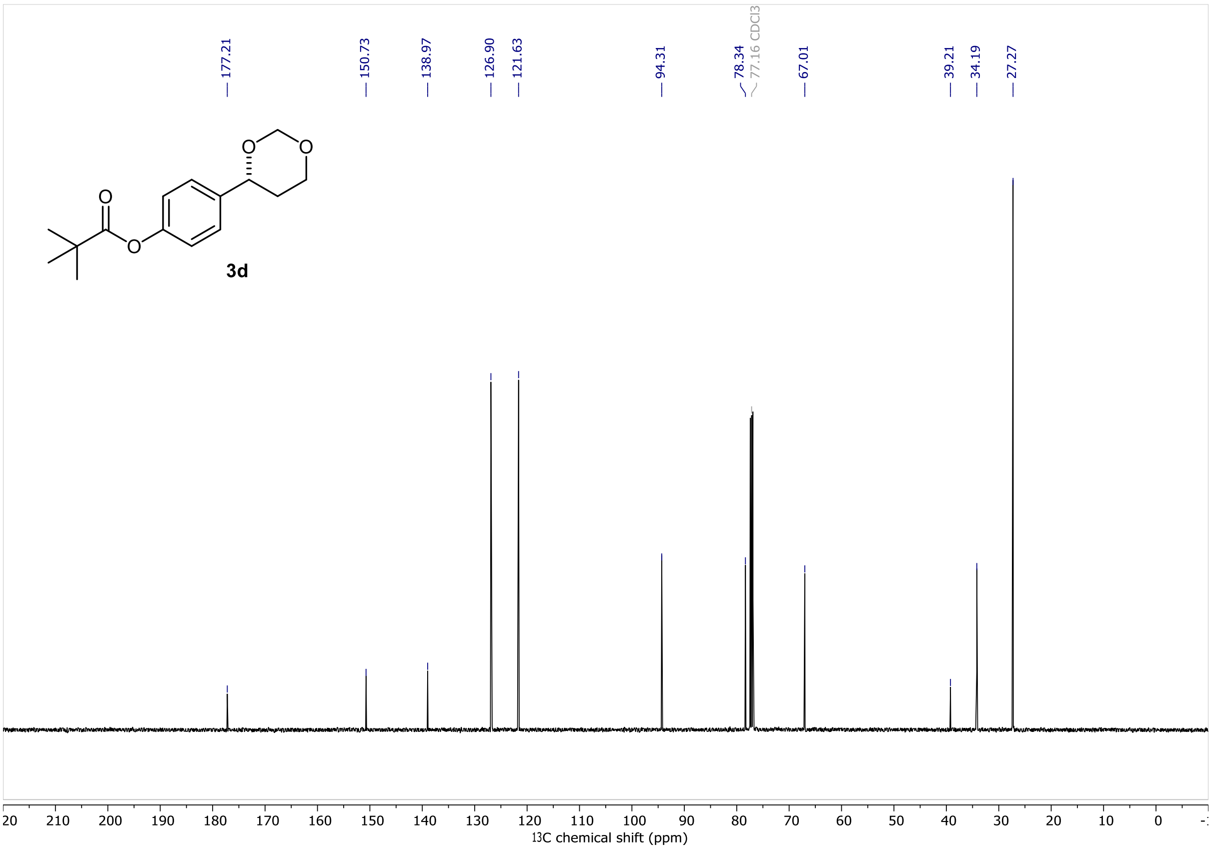

NMR spectra for compound **3d**: ^{1}H (501 MHz) and ^{13}C (126 MHz), in $CDCl_3$.

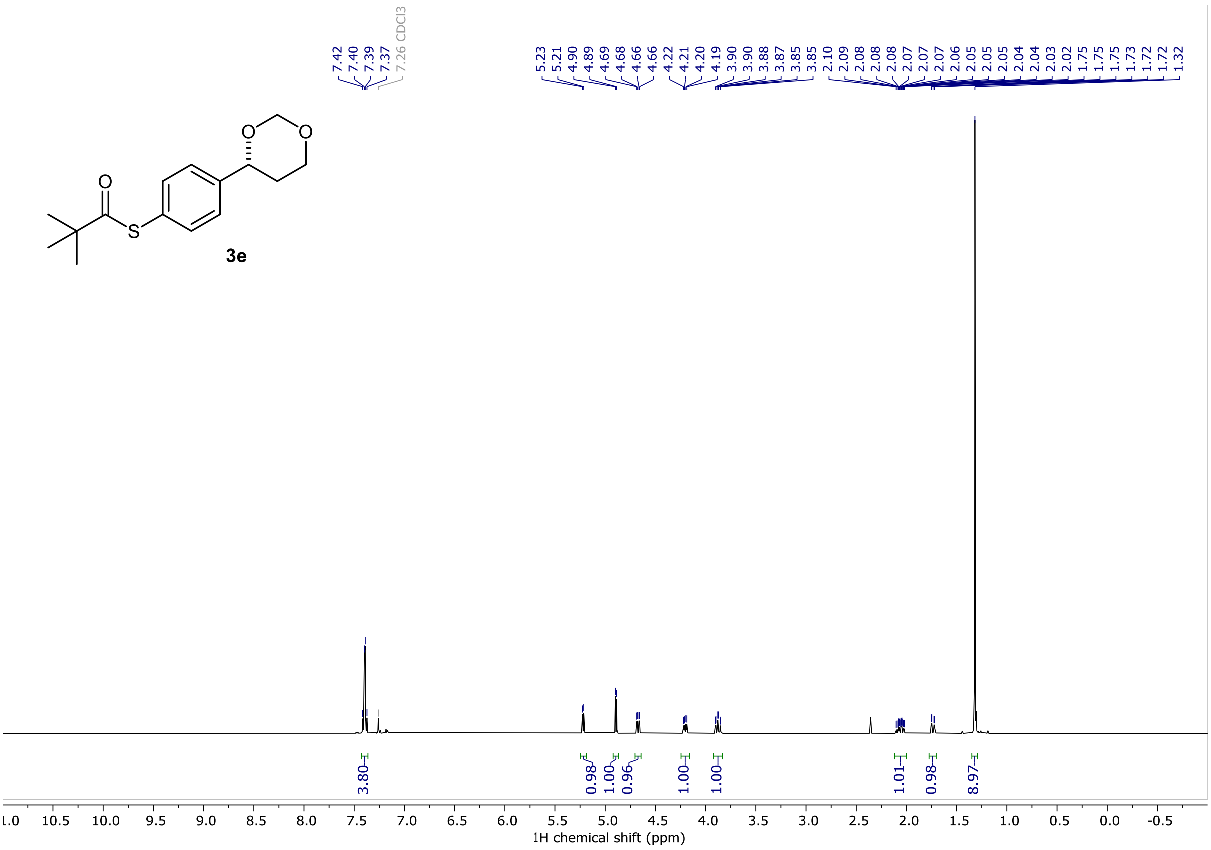

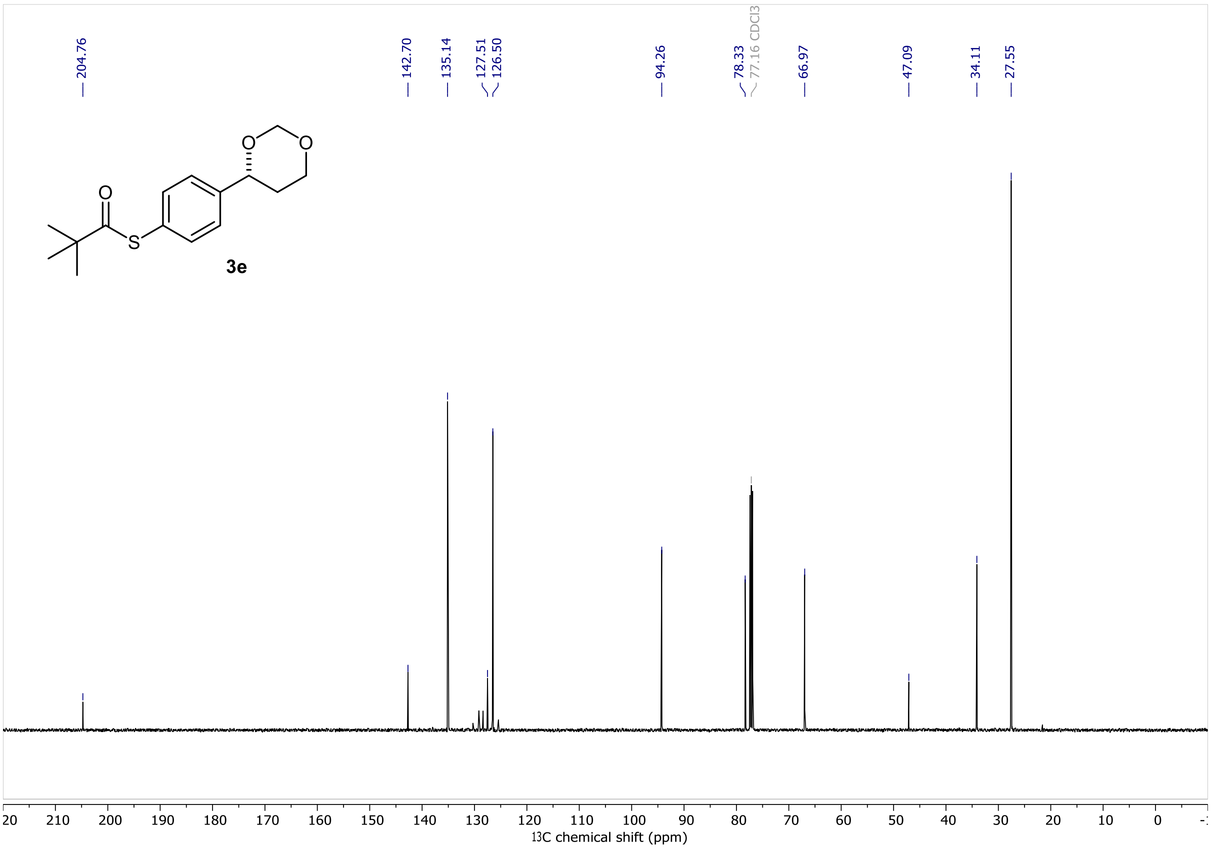

NMR spectra for compound **3e**: ^{1}H (501 MHz) and ^{13}C (126 MHz), in $CDCl_3$.

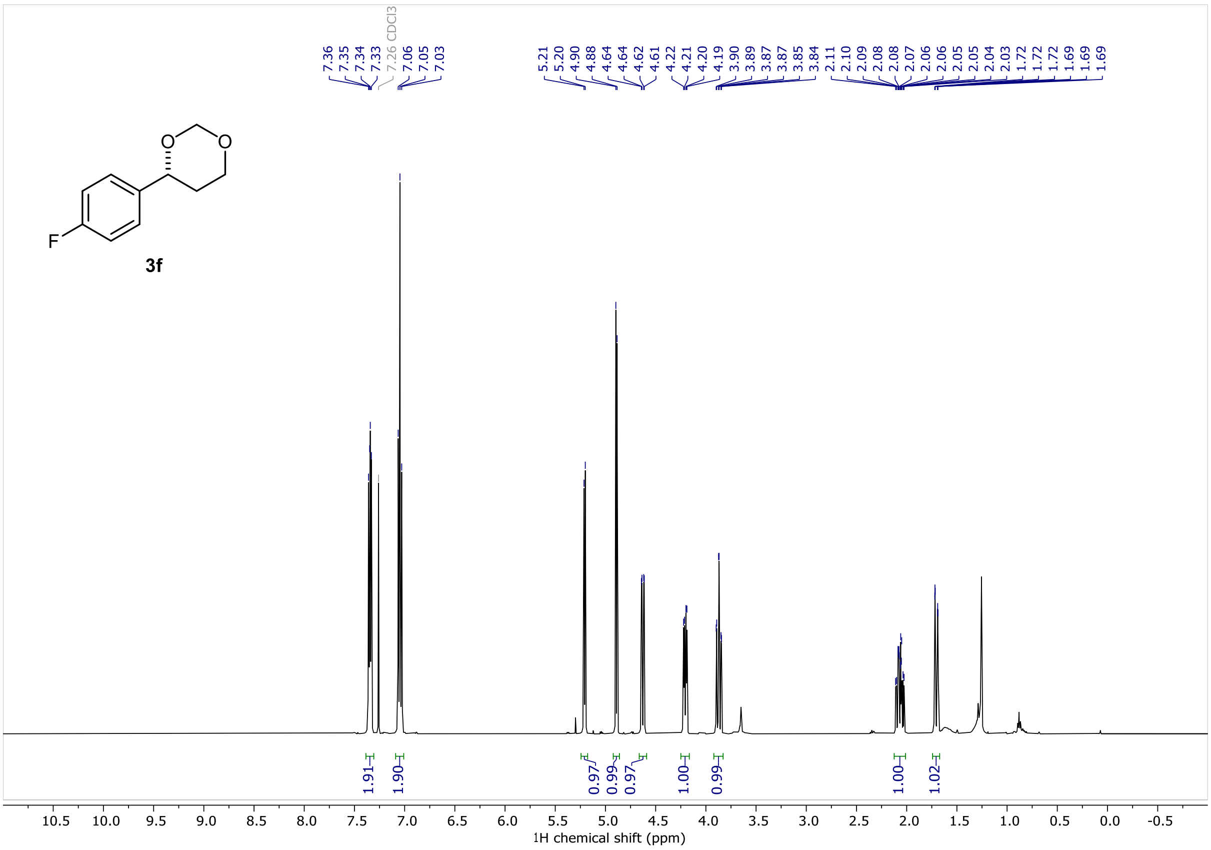

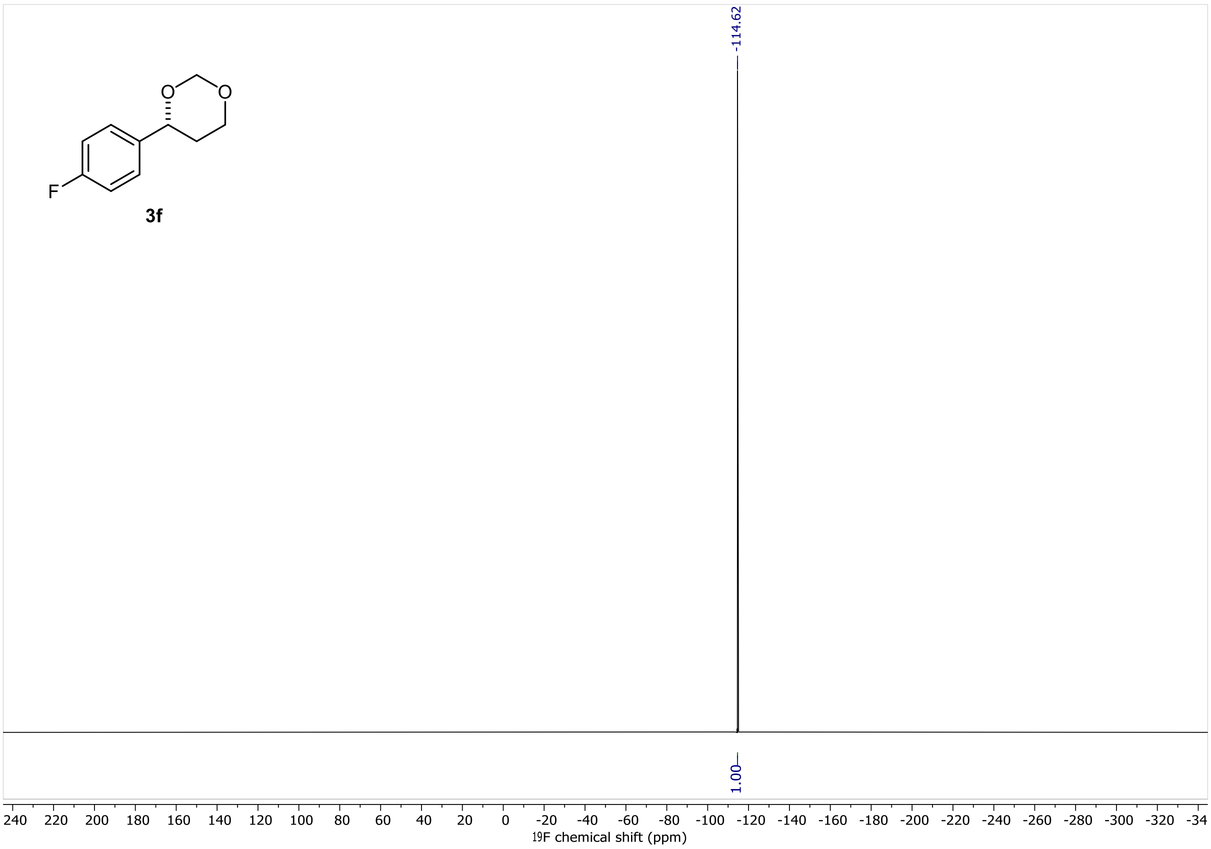

NMR spectra for compound **3f**: ^{1}H (501 MHz) and ^{19}F (471 MHz), in $CDCl_3$.

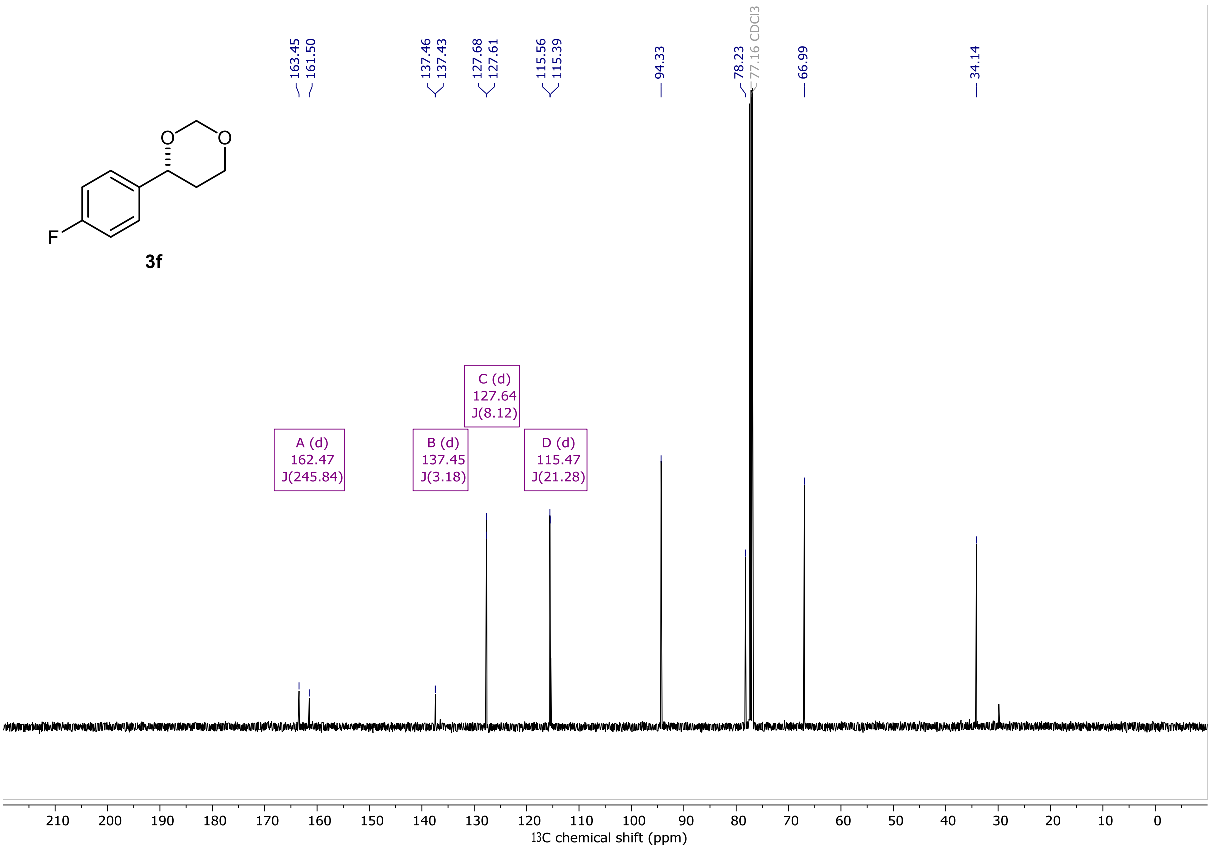

NMR spectra for compound **3f** *(continuation)*: ^{13}C (126 MHz), in $CDCl_3$.

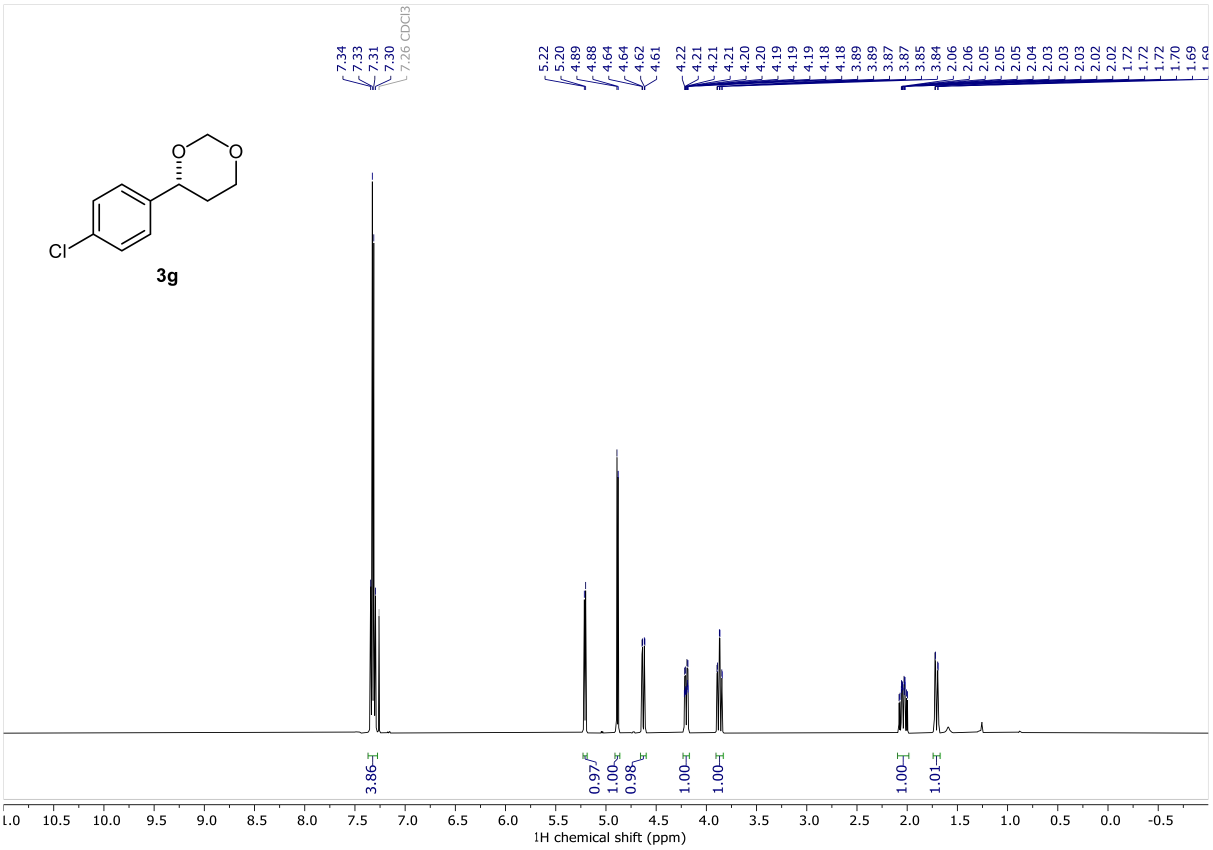

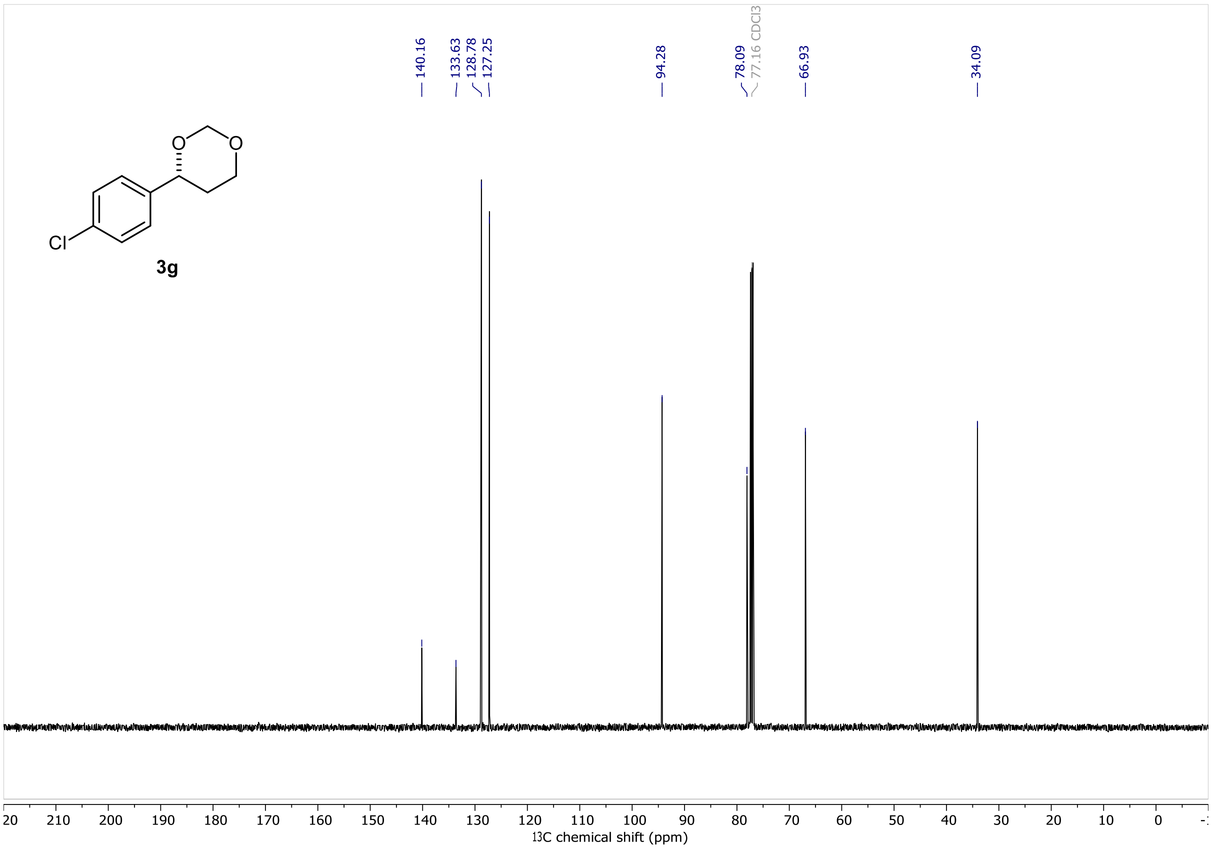

NMR spectra for compound **3g**: ^{1}H (501 MHz) and ^{13}C (126 MHz), in $CDCl_3$.

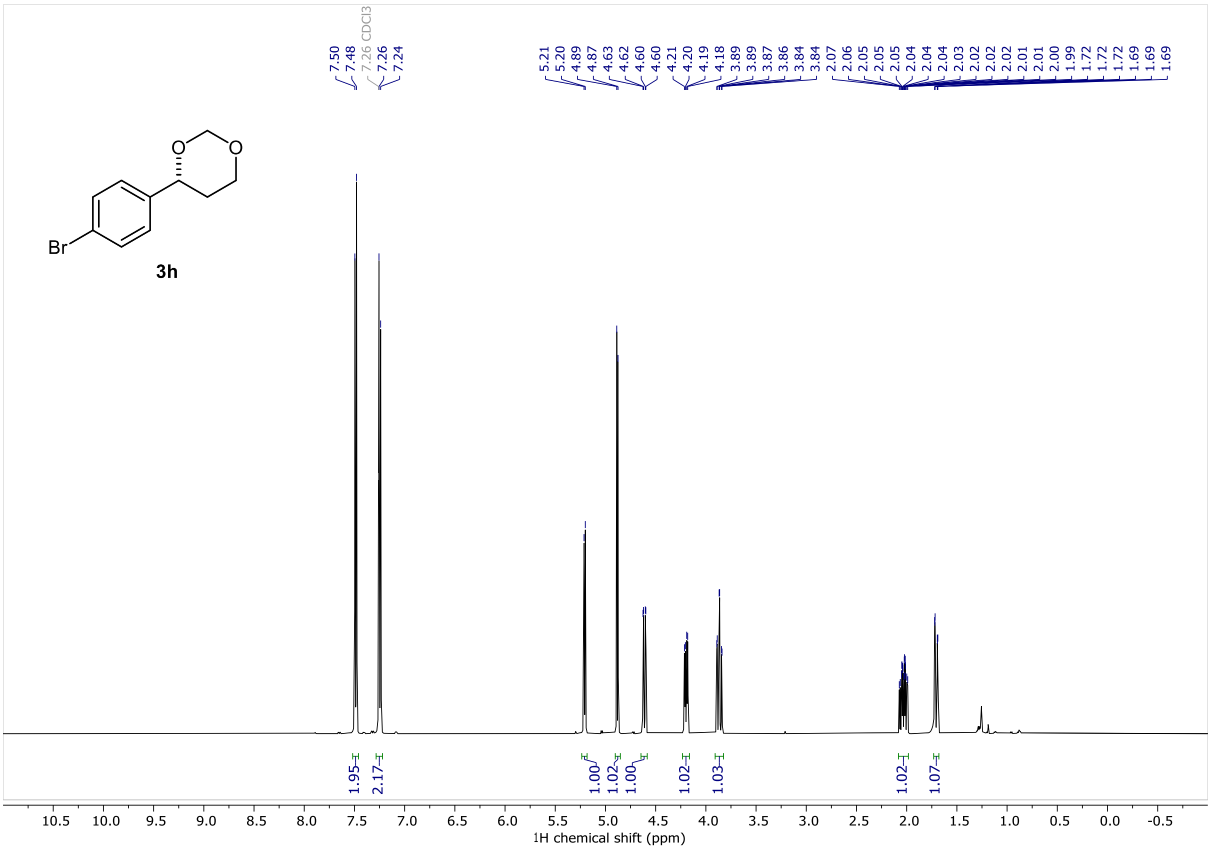

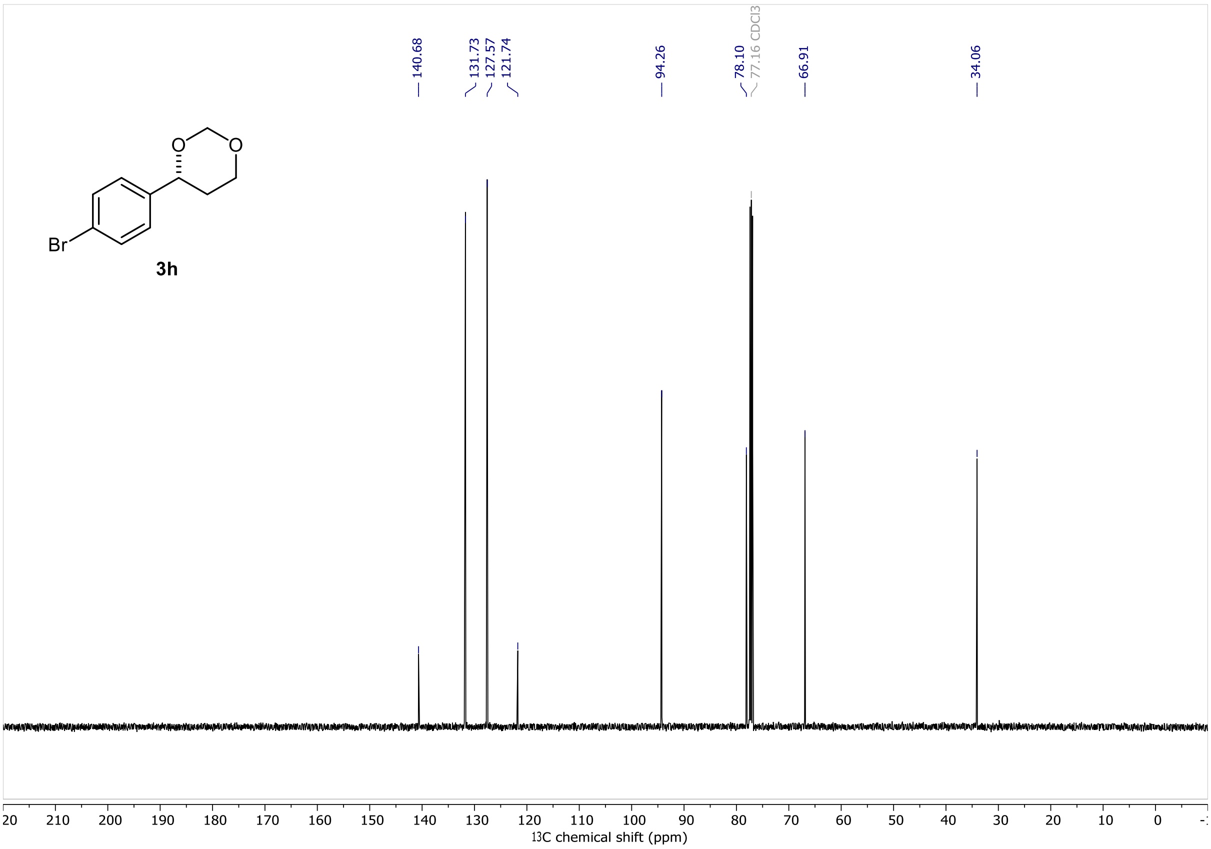

NMR spectra for compound **3h**: ^{1}H (501 MHz) and ^{13}C (126 MHz), in $CDCl_3$.

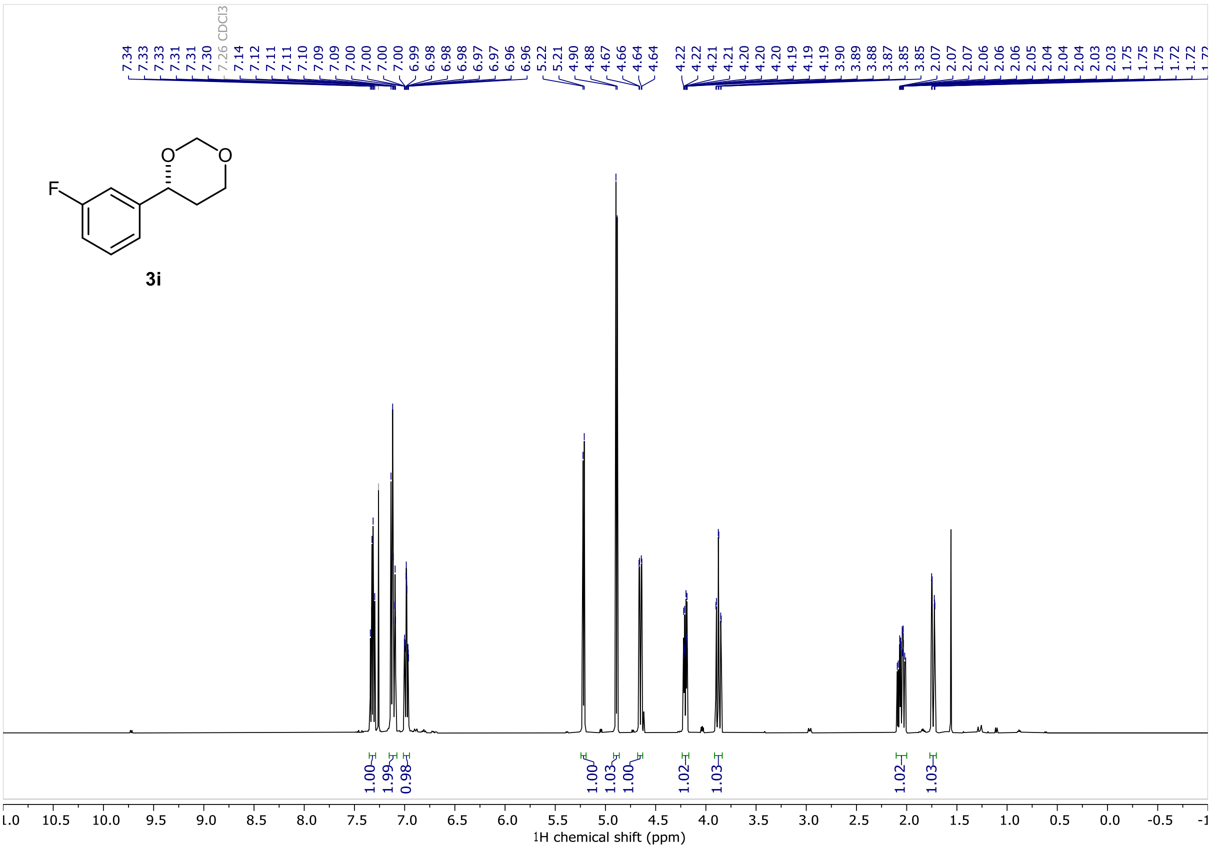

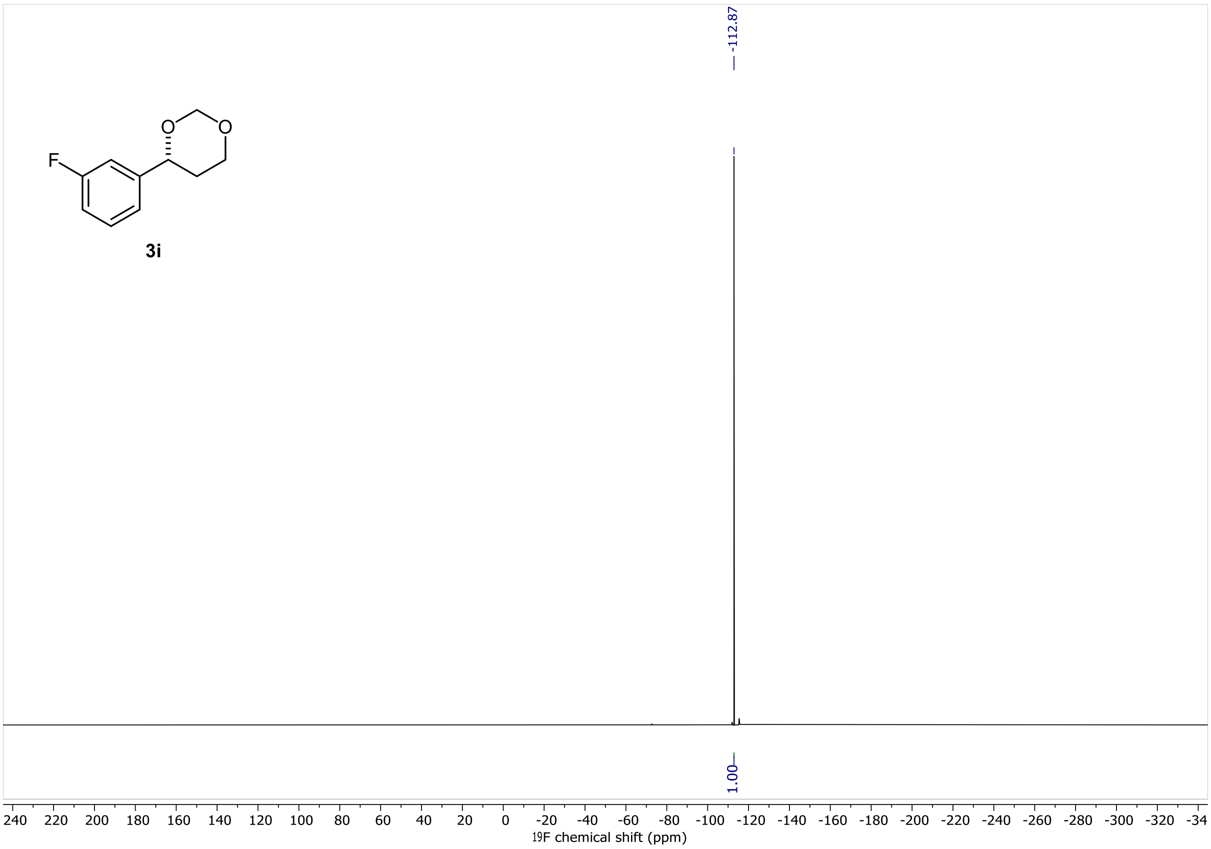

NMR spectra for compound **3i**: ^{1}H (501 MHz) and ^{19}F (471 MHz), in $CDCl_3$.

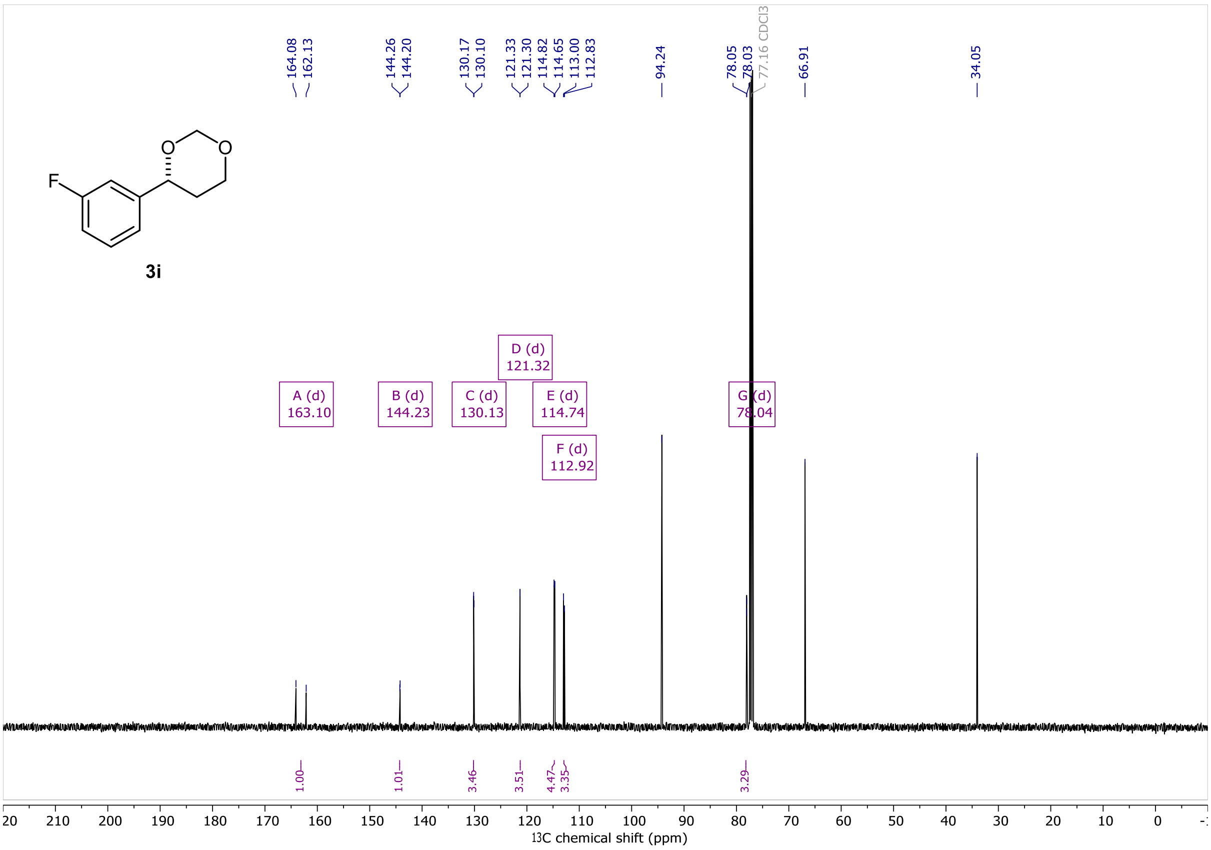

NMR spectra for compound **3i** *(continuation)*: ^{13}C (126 MHz), in $CDCl_3$.

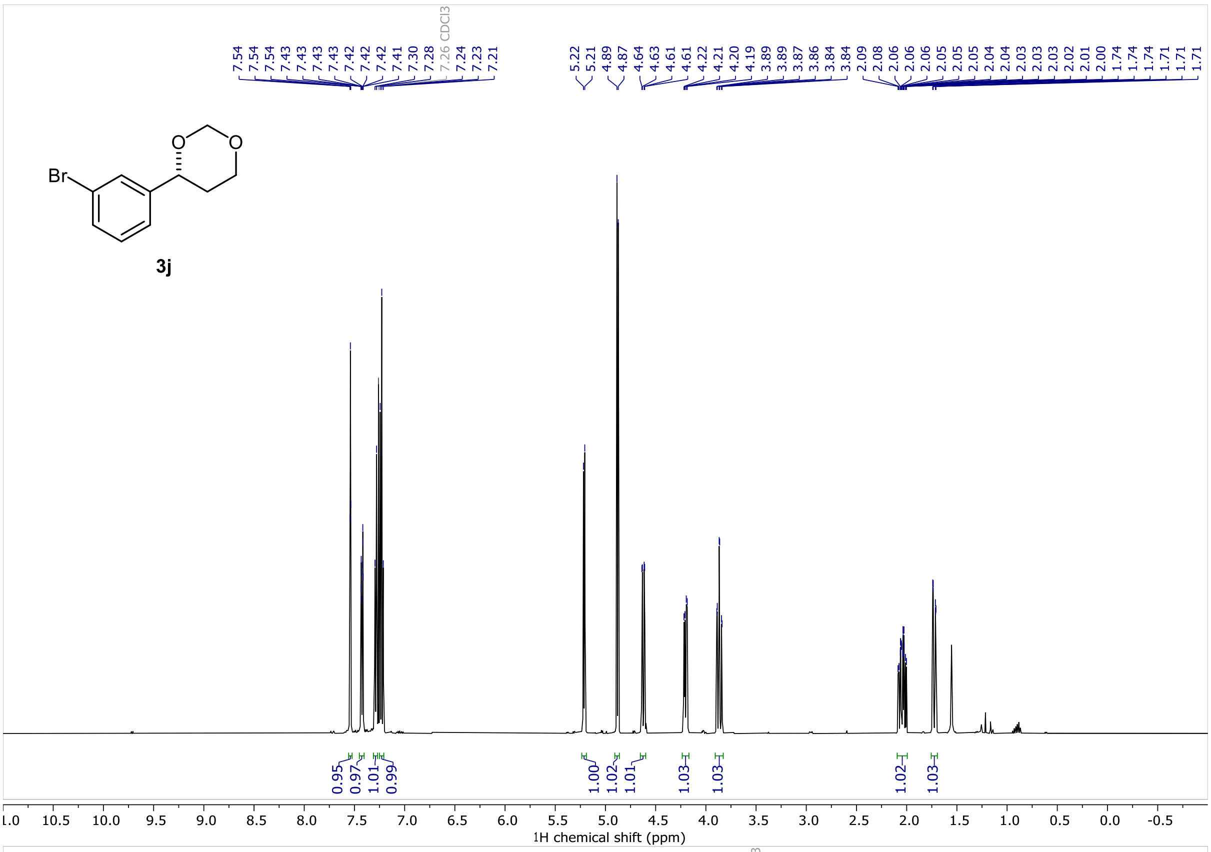

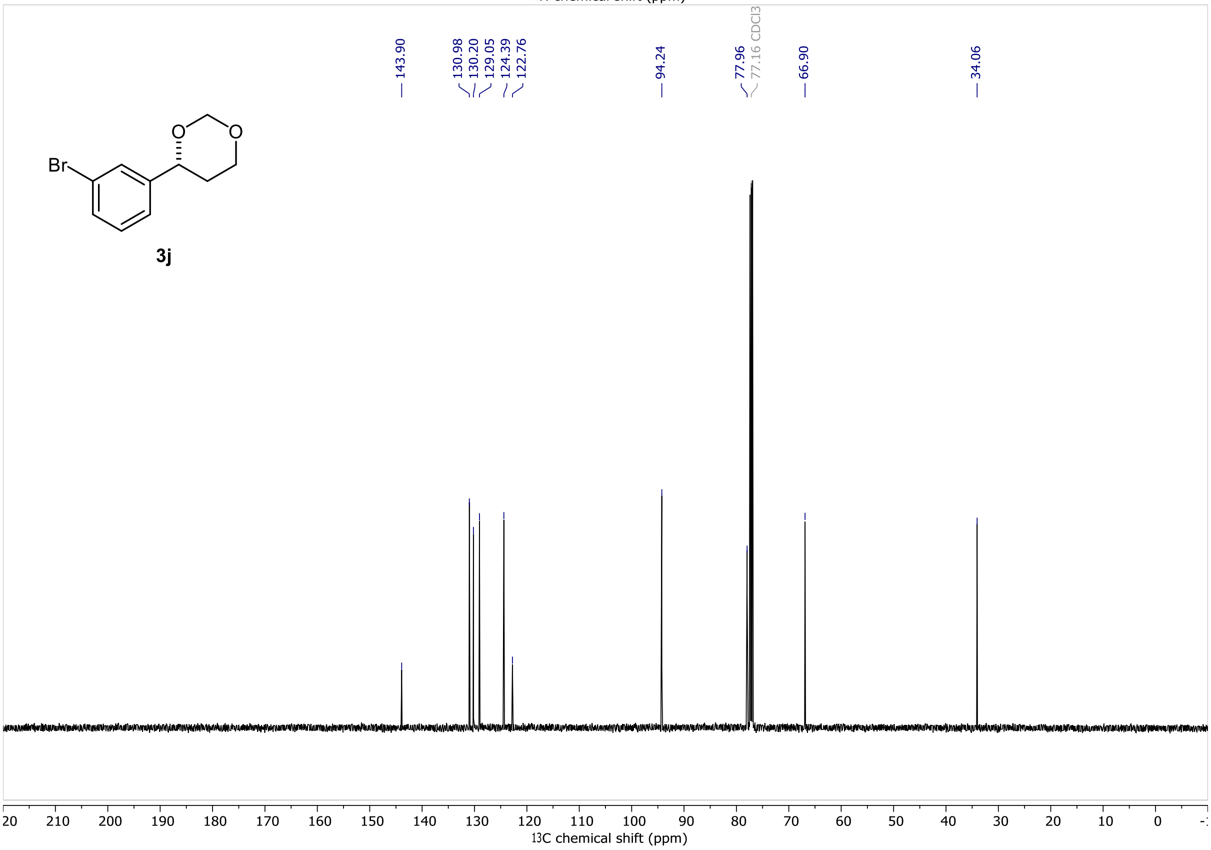

NMR spectra for compound **3j**: ^{1}H (501 MHz) and ^{13}C (126 MHz), in $CDCl_3$.

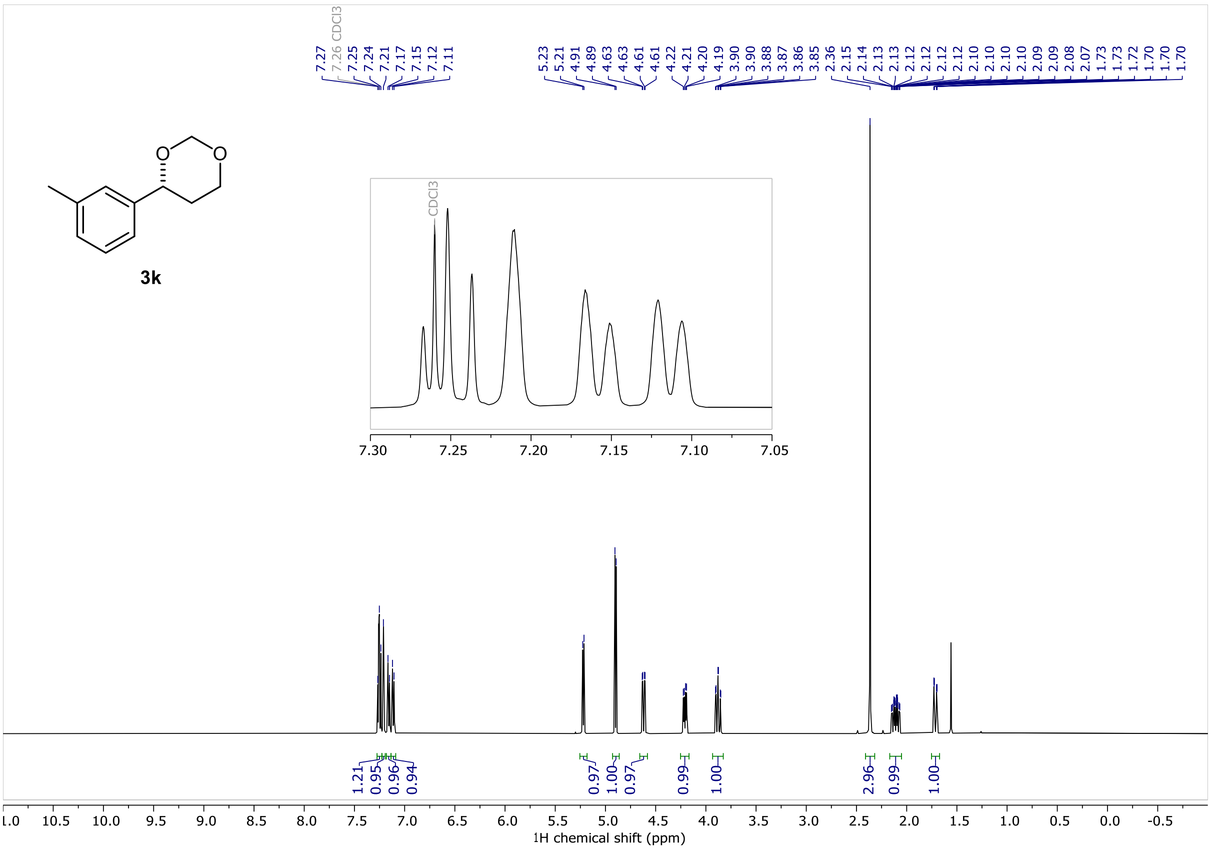

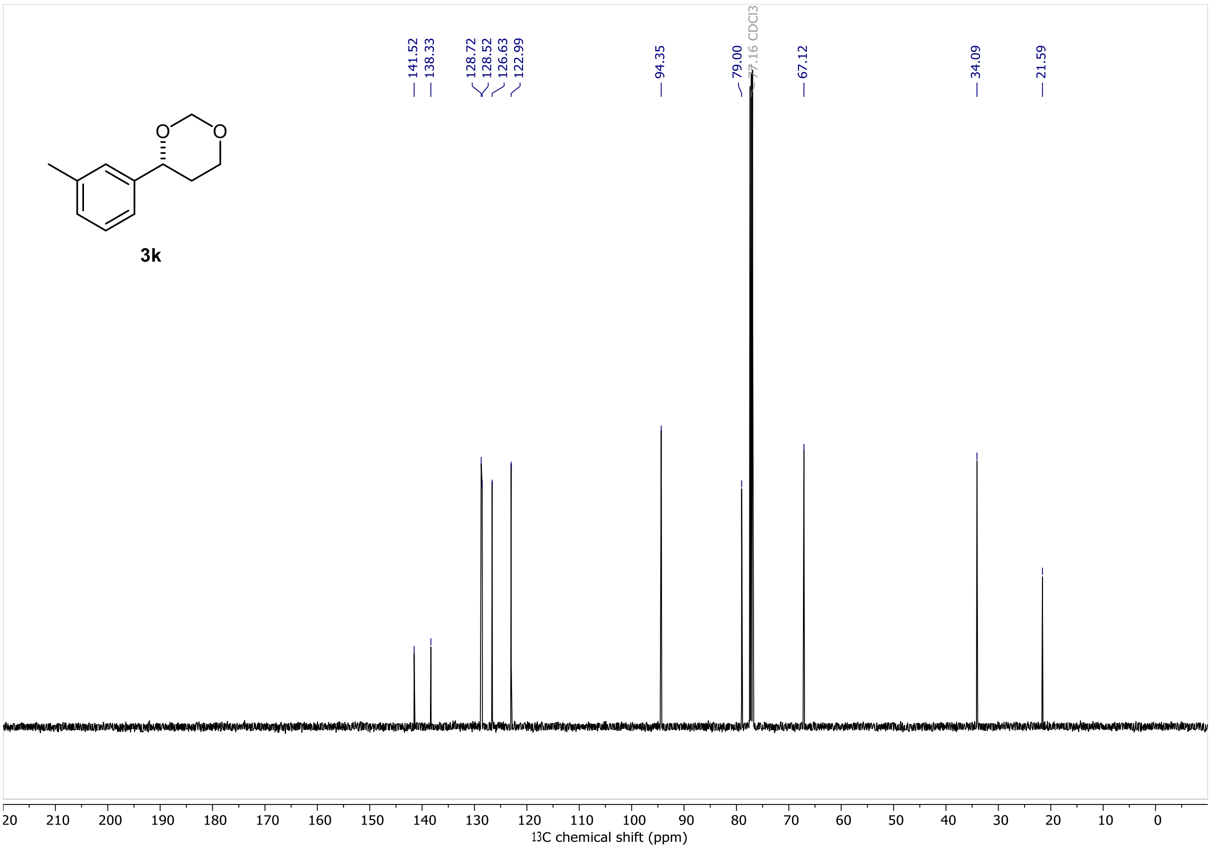

NMR spectra for compound **3k**: ^{1}H (501 MHz) and ^{13}C (126 MHz), in $CDCl_3$.

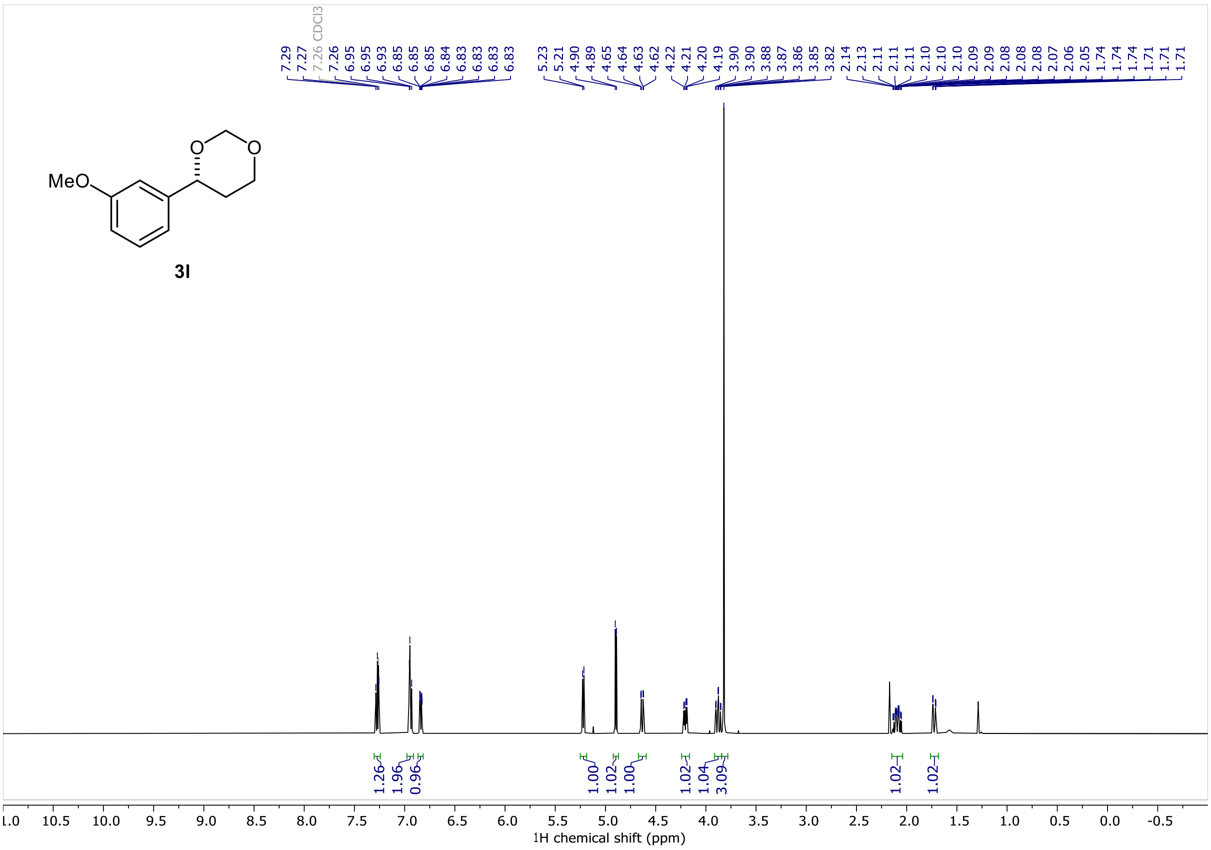

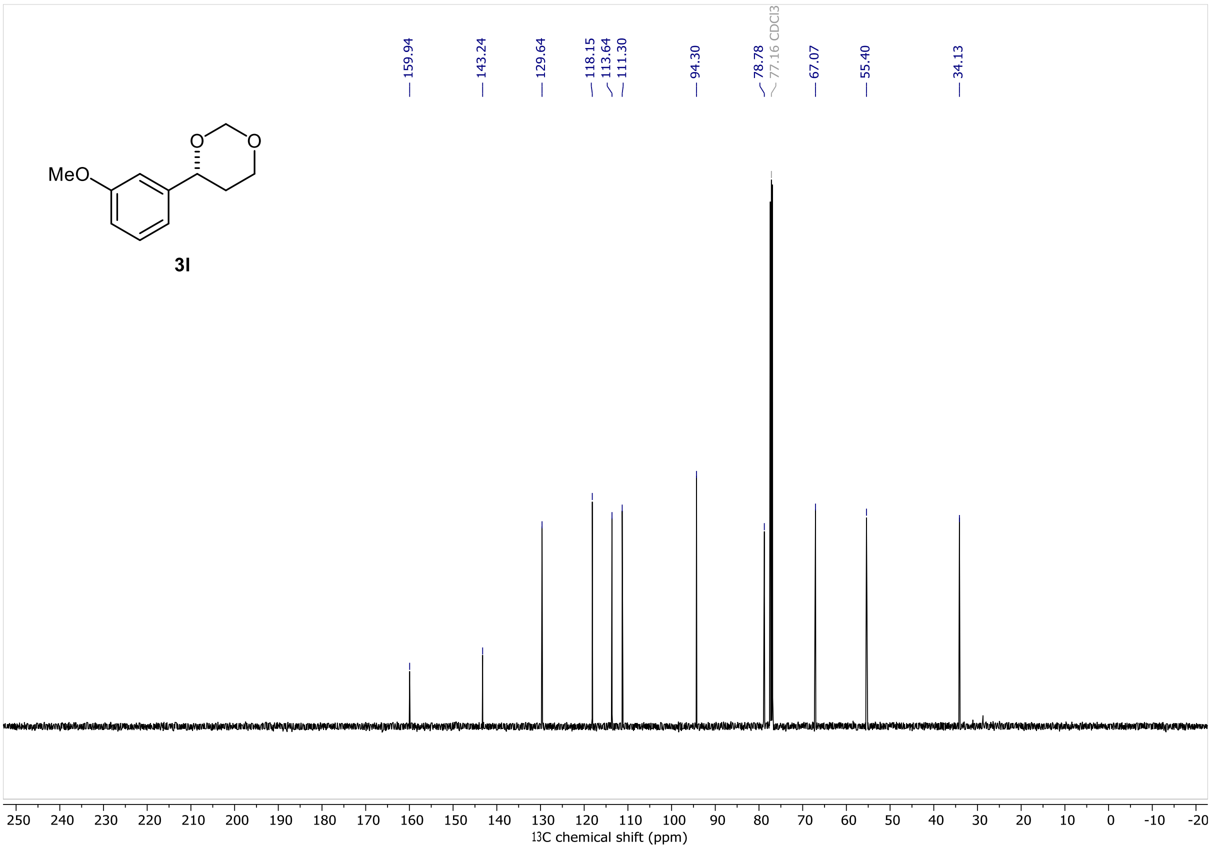

NMR spectra for compound **3l**: ^{1}H (501 MHz) and ^{13}C (126 MHz), in $CDCl_3$.

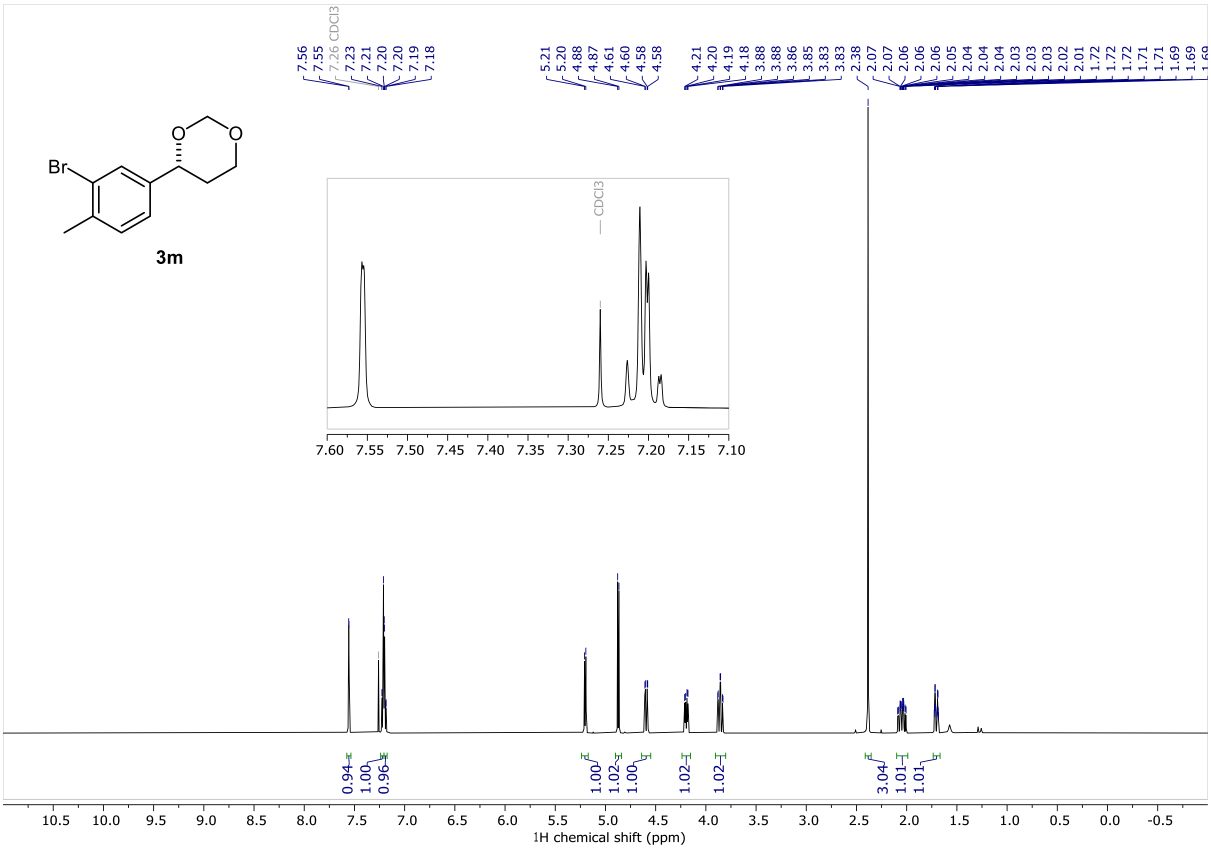

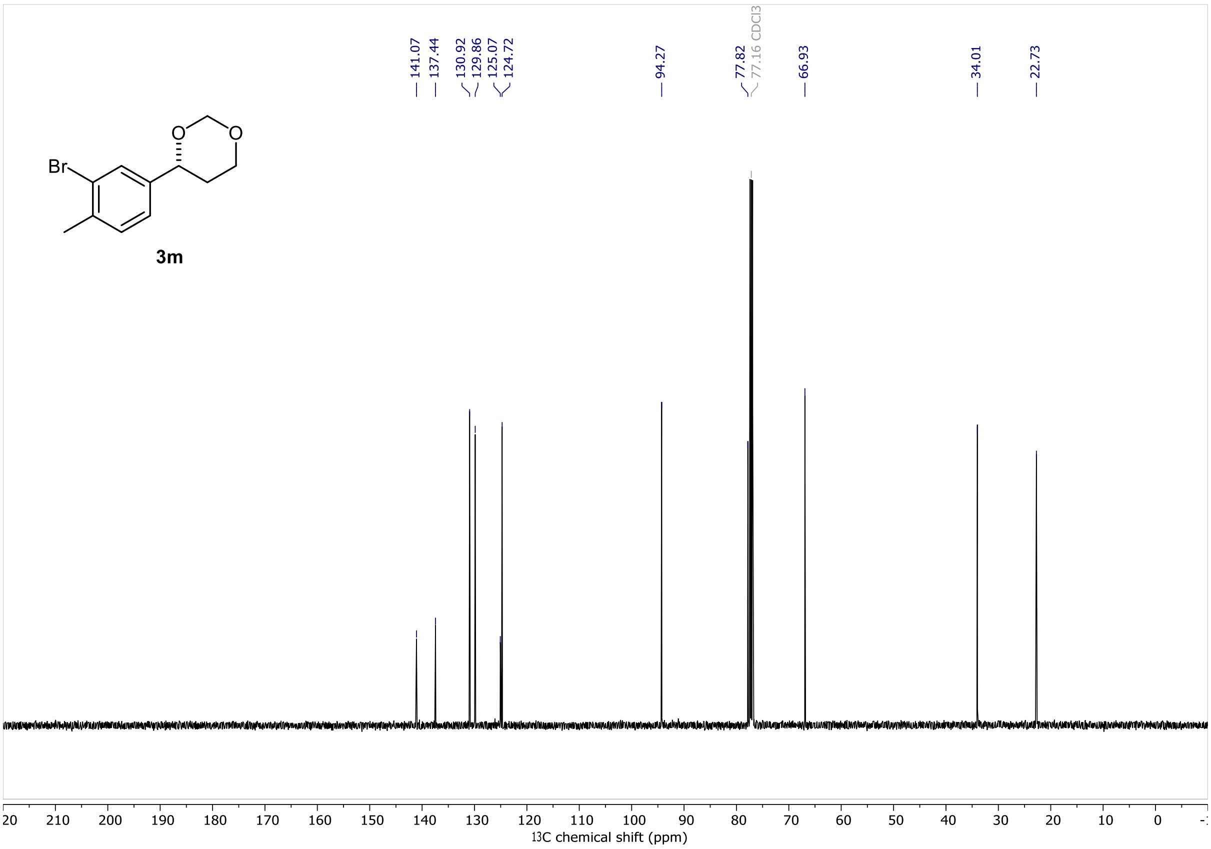

NMR spectra for compound **3m**: ^{1}H (501 MHz) and ^{13}C (126 MHz), in $CDCl_3$.

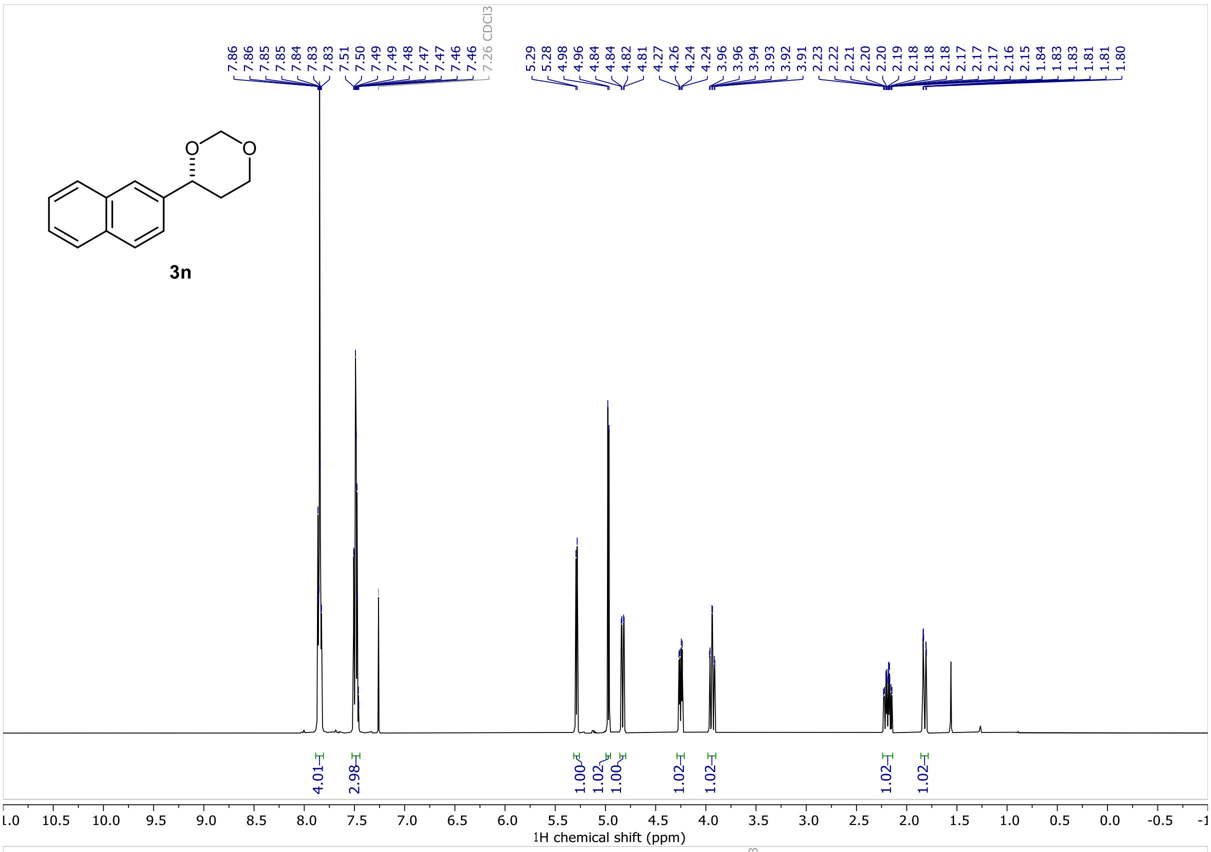

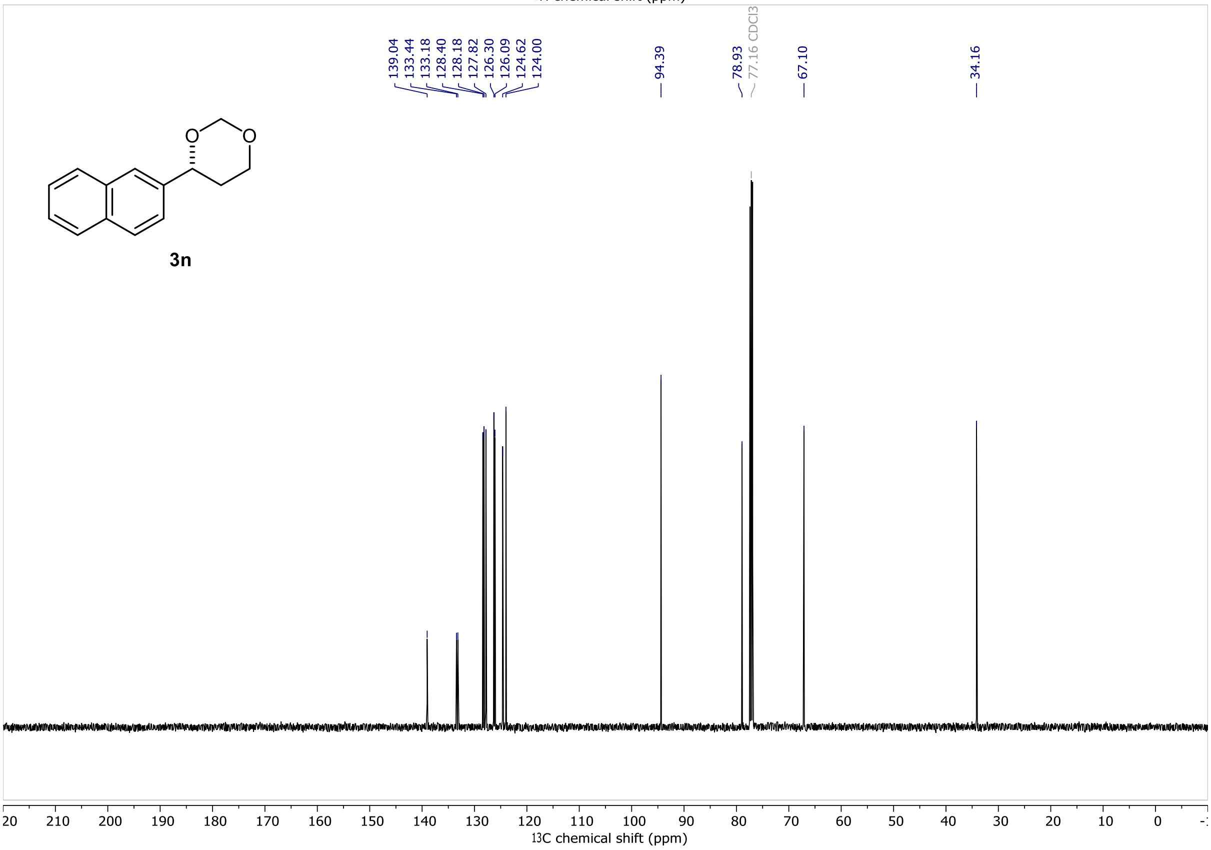

NMR spectra for compound **3n**: ^{1}H (501 MHz) and ^{13}C (126 MHz), in $CDCl_3$.

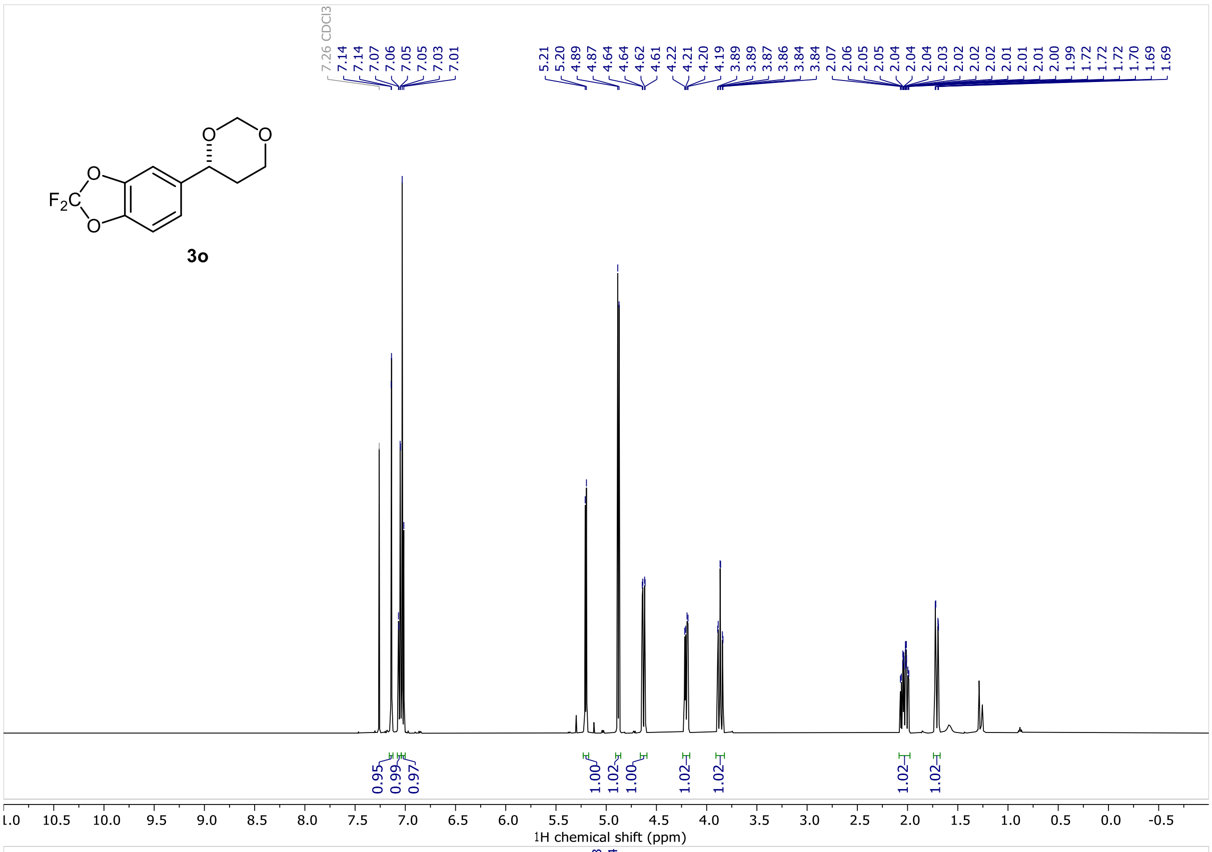

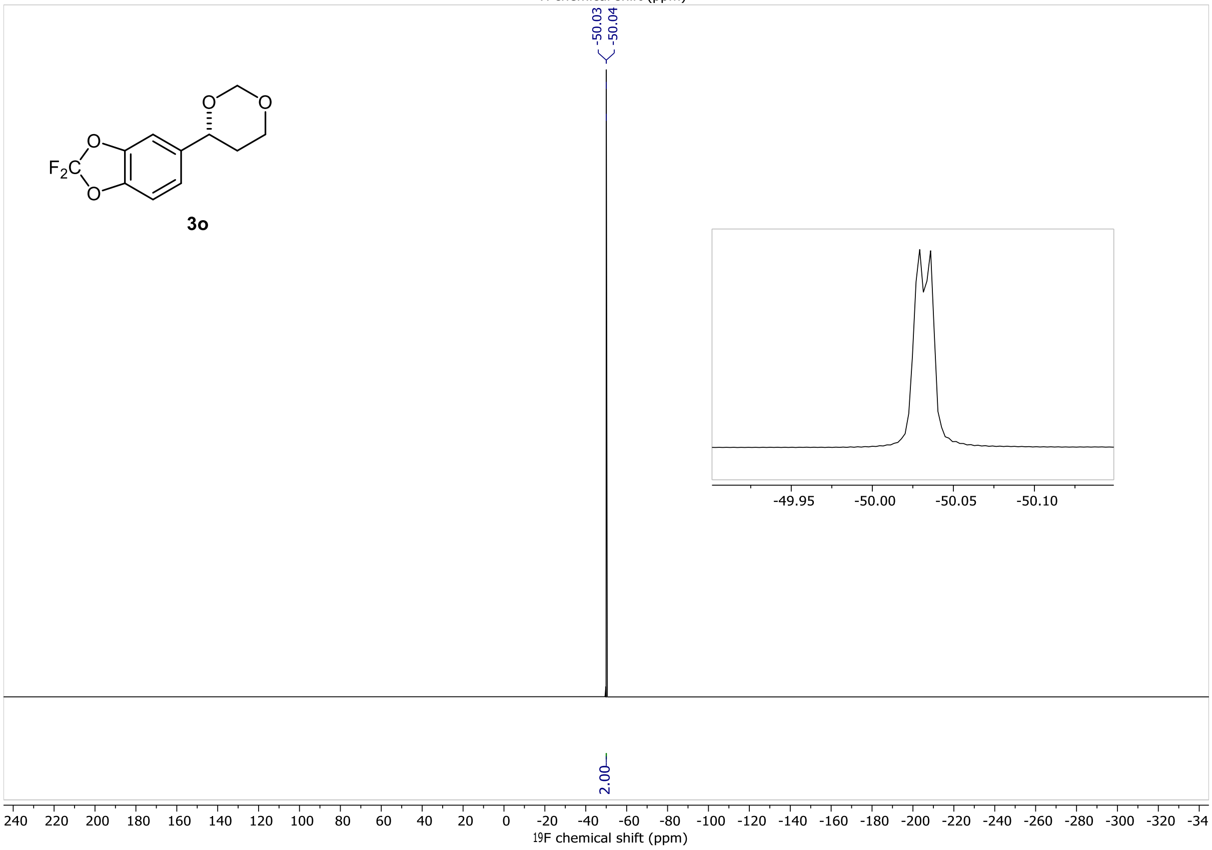

NMR spectra for compound **3o**: ^{1}H (501 MHz) and ^{19}F (471 MHz), in $CDCl_3$.

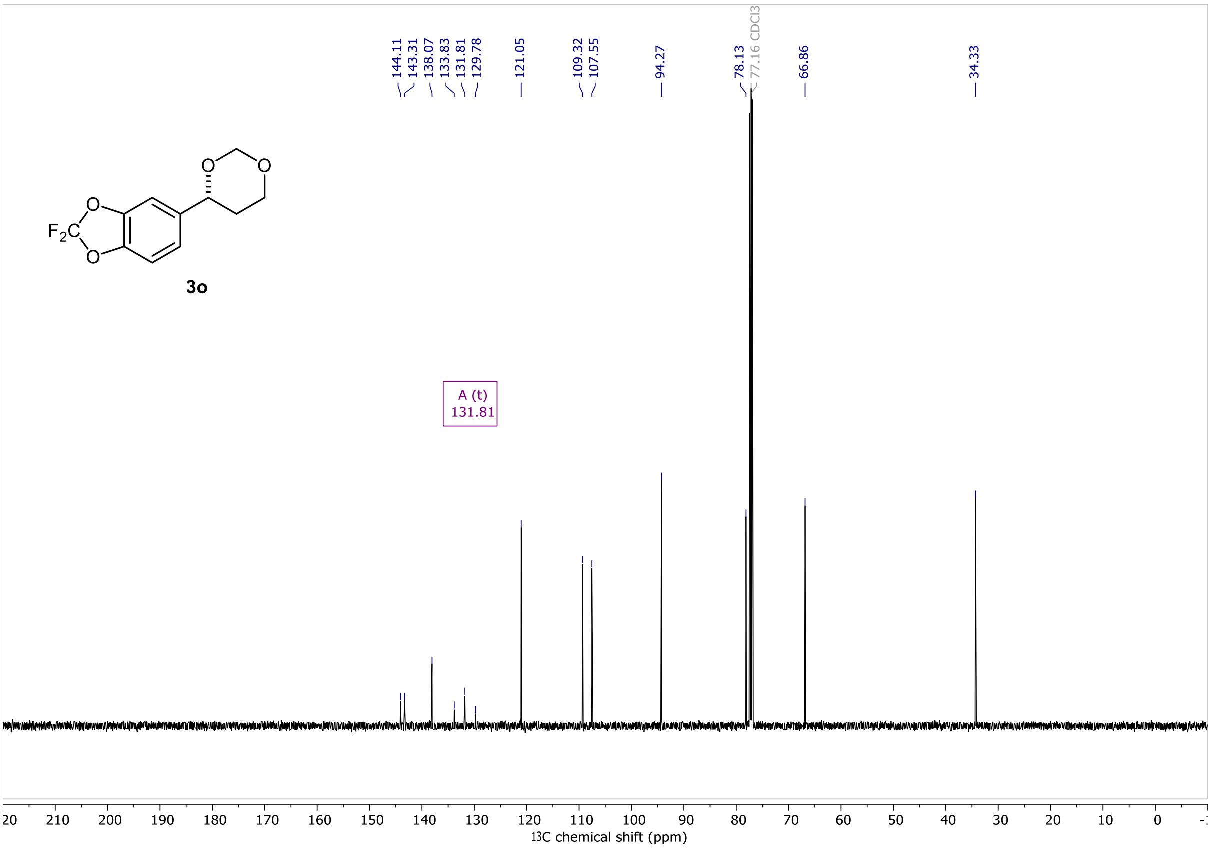

NMR spectra for compound **3o** *(continuation)*: ^{13}C (126 MHz), in $CDCl_3$.

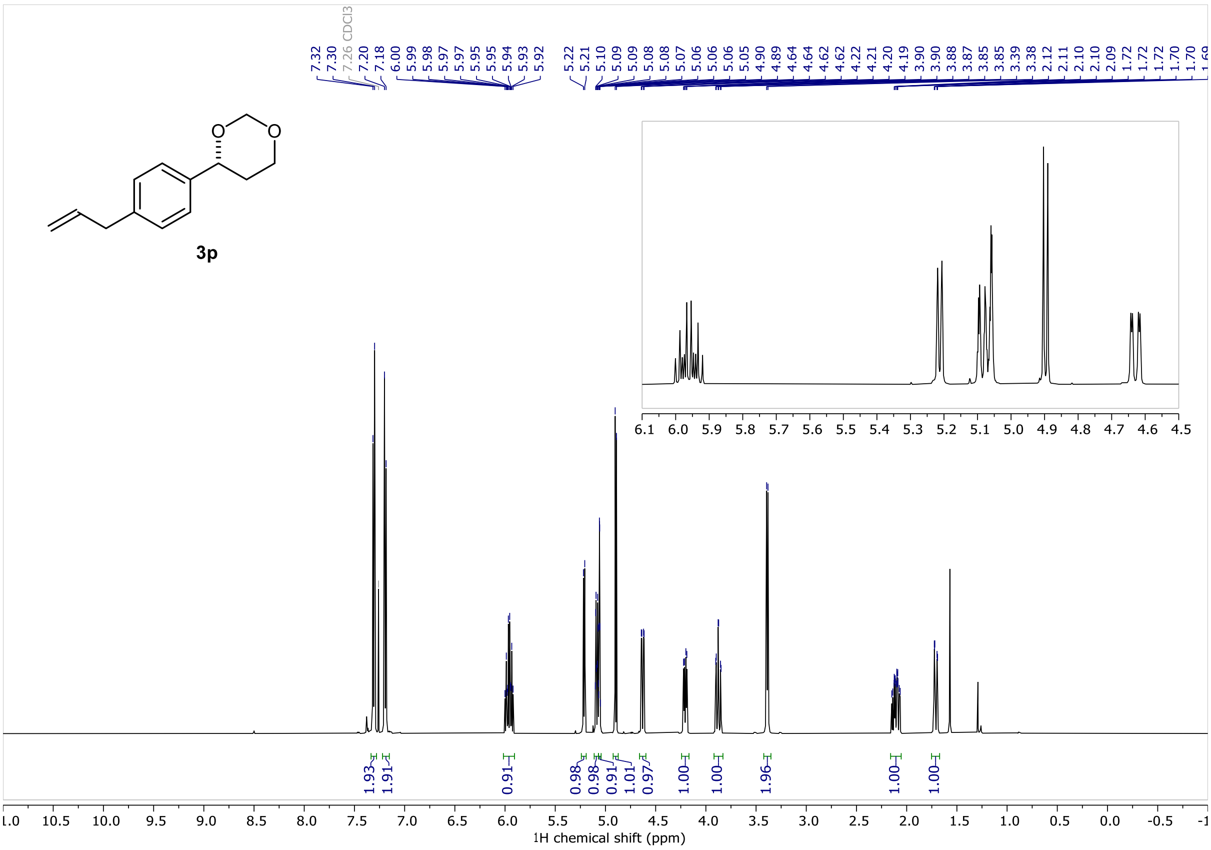

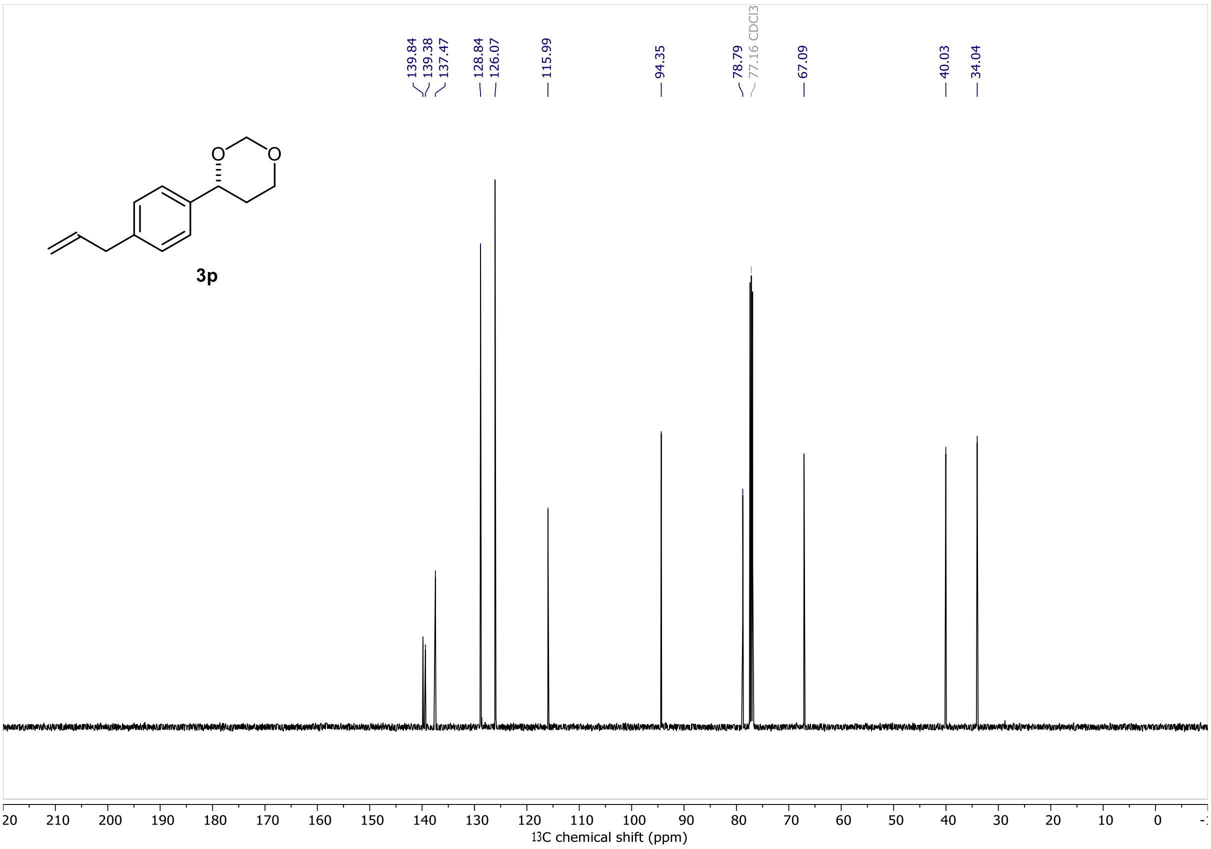

NMR spectra for compound **3p**: ^{1}H (501 MHz) and ^{13}C (126 MHz), in $CDCl_3$.

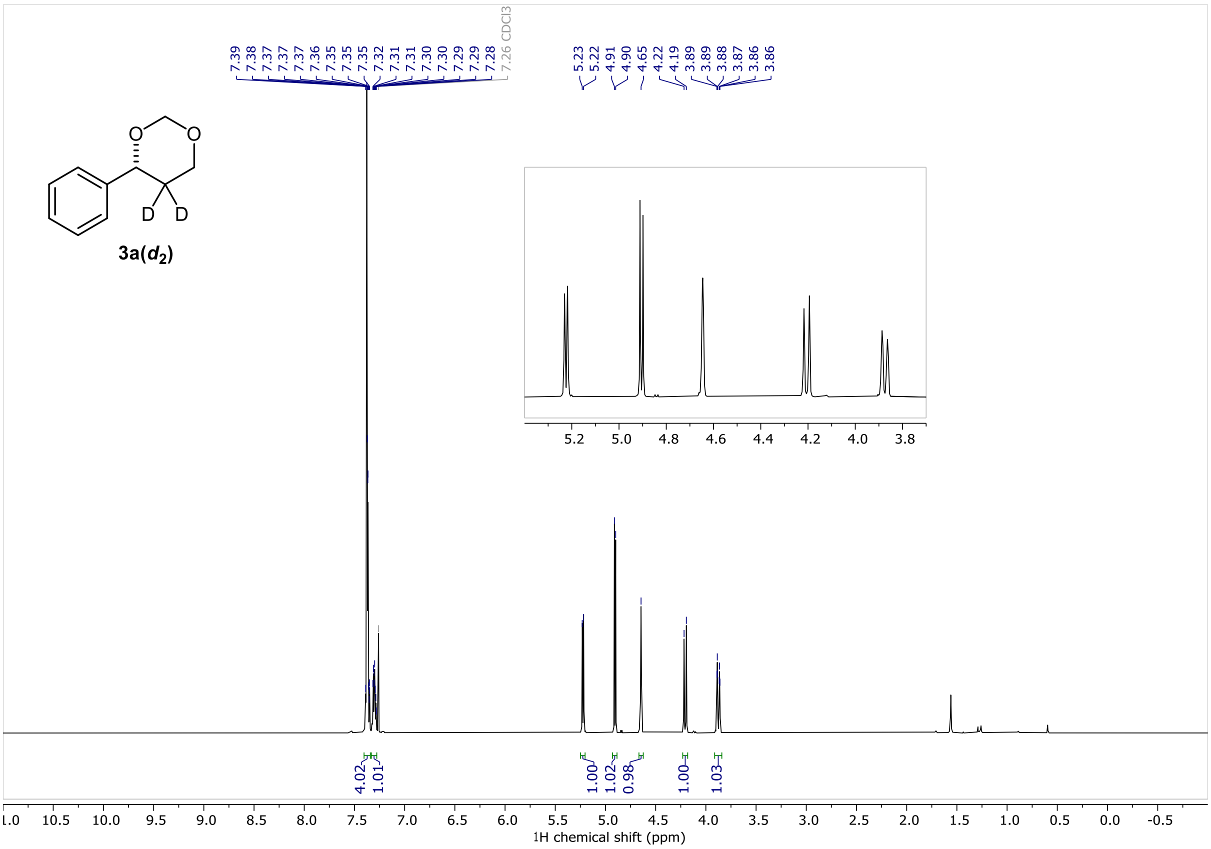

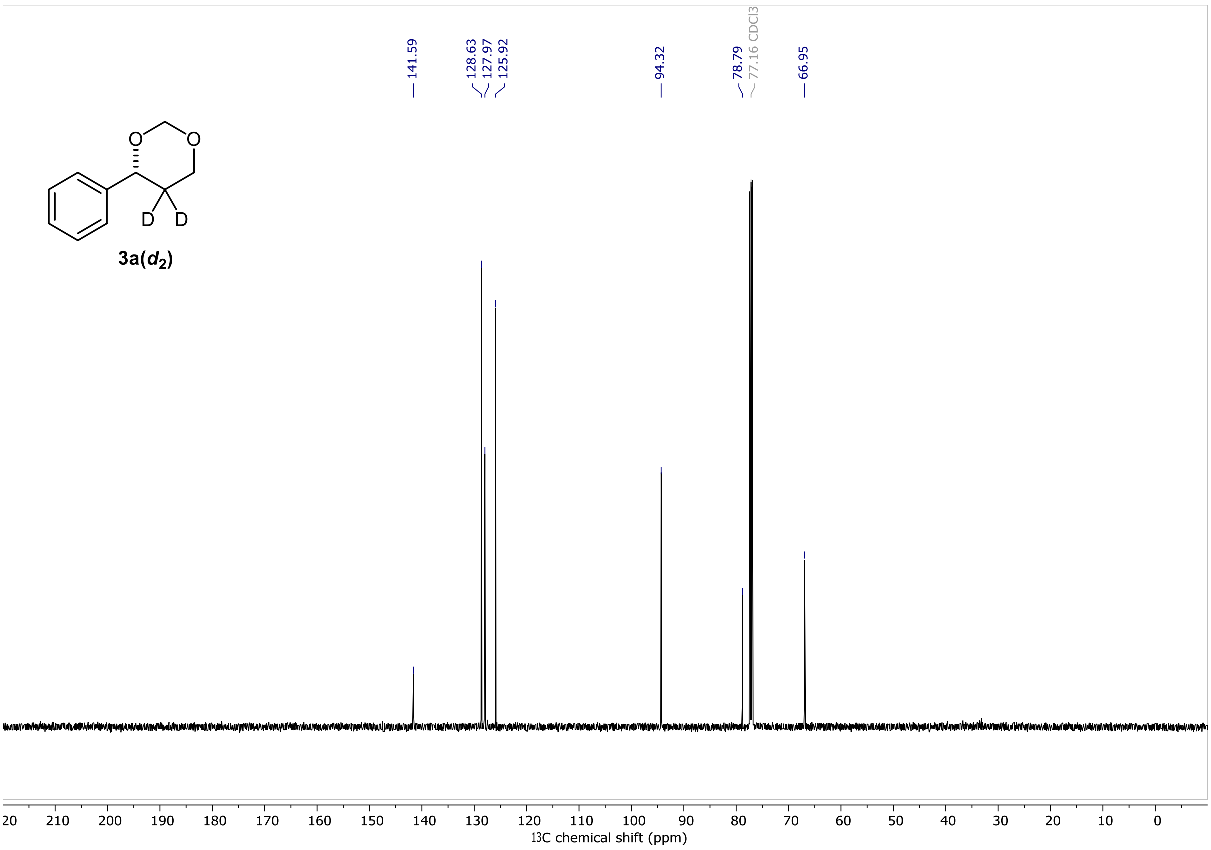

NMR spectra for compound **3a(*d*$_2$)**: ^{1}H (501 MHz) and ^{13}C (126 MHz), in $CDCl_3$.

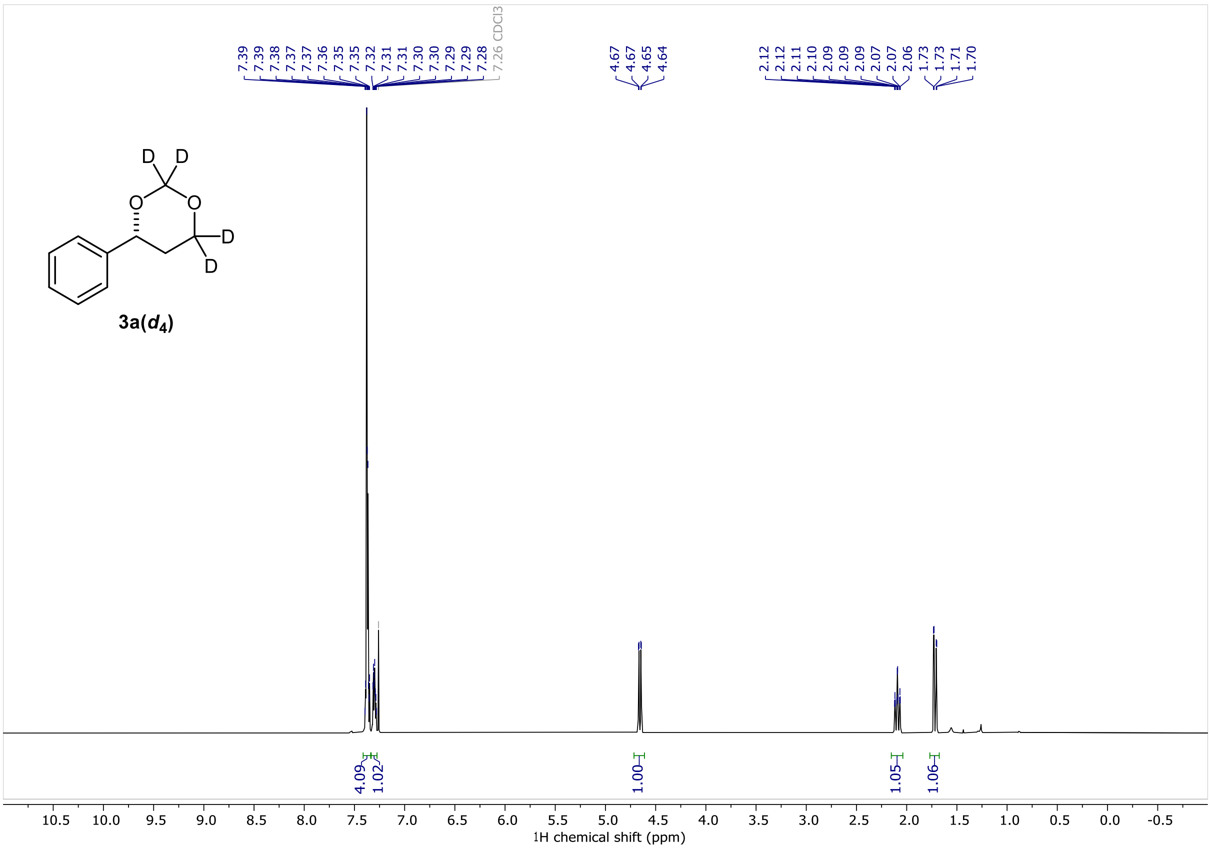

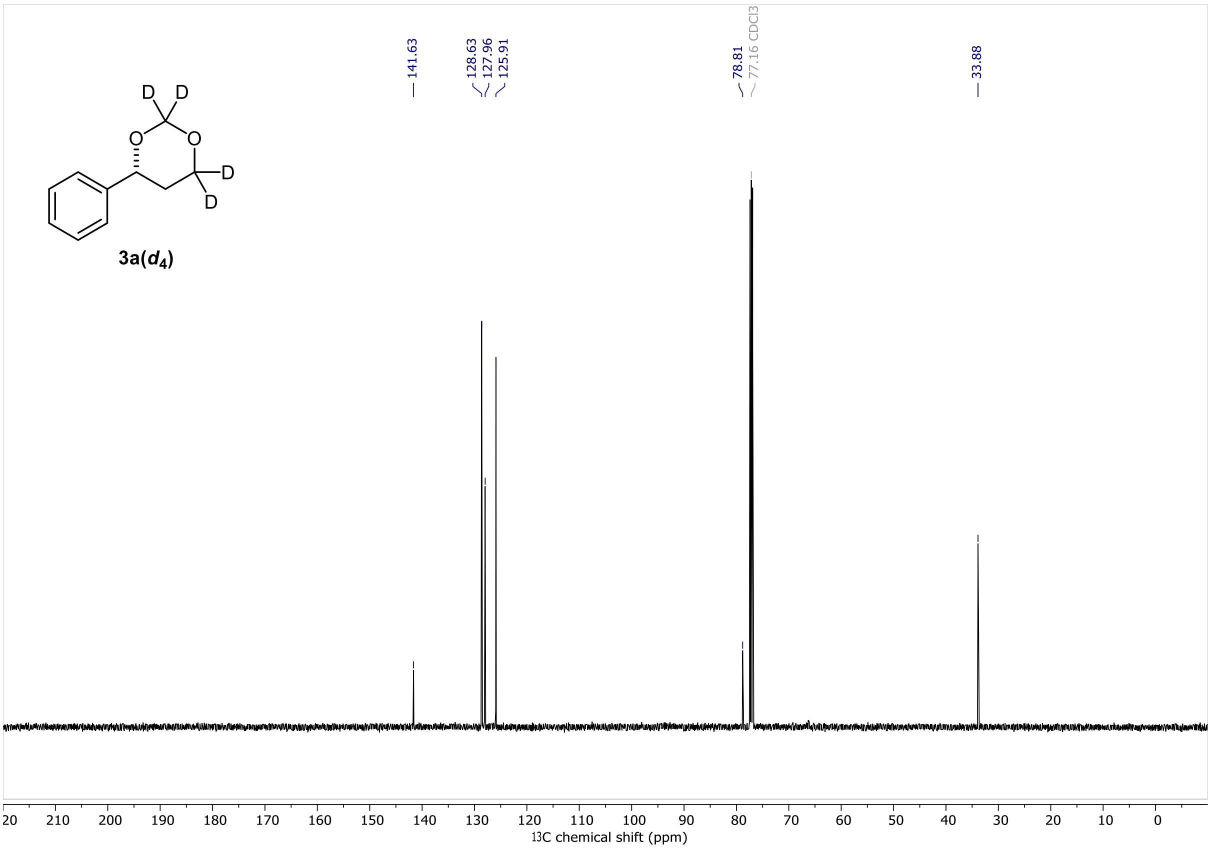

NMR spectra for compound **3a(*d*$_4$)**: ^{1}H (501 MHz) and ^{13}C (126 MHz), in $CDCl_3$.

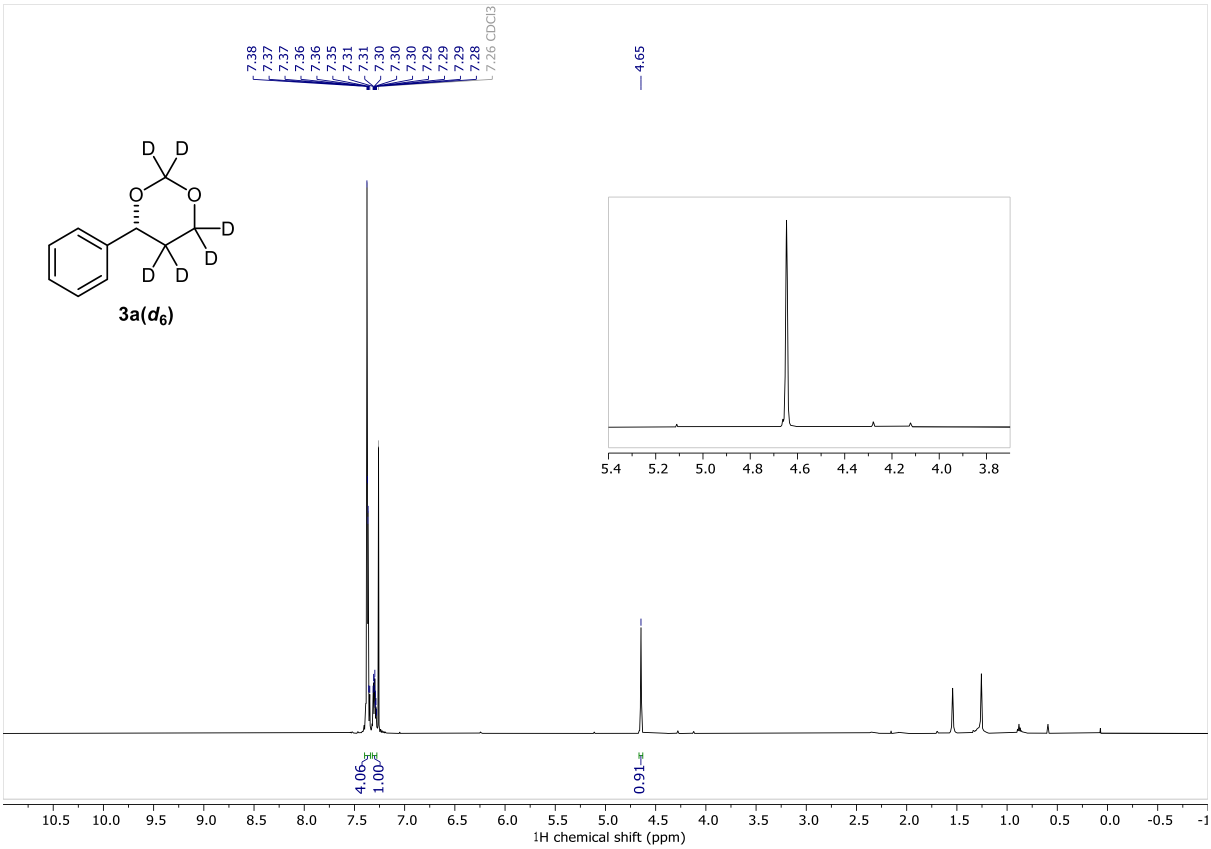

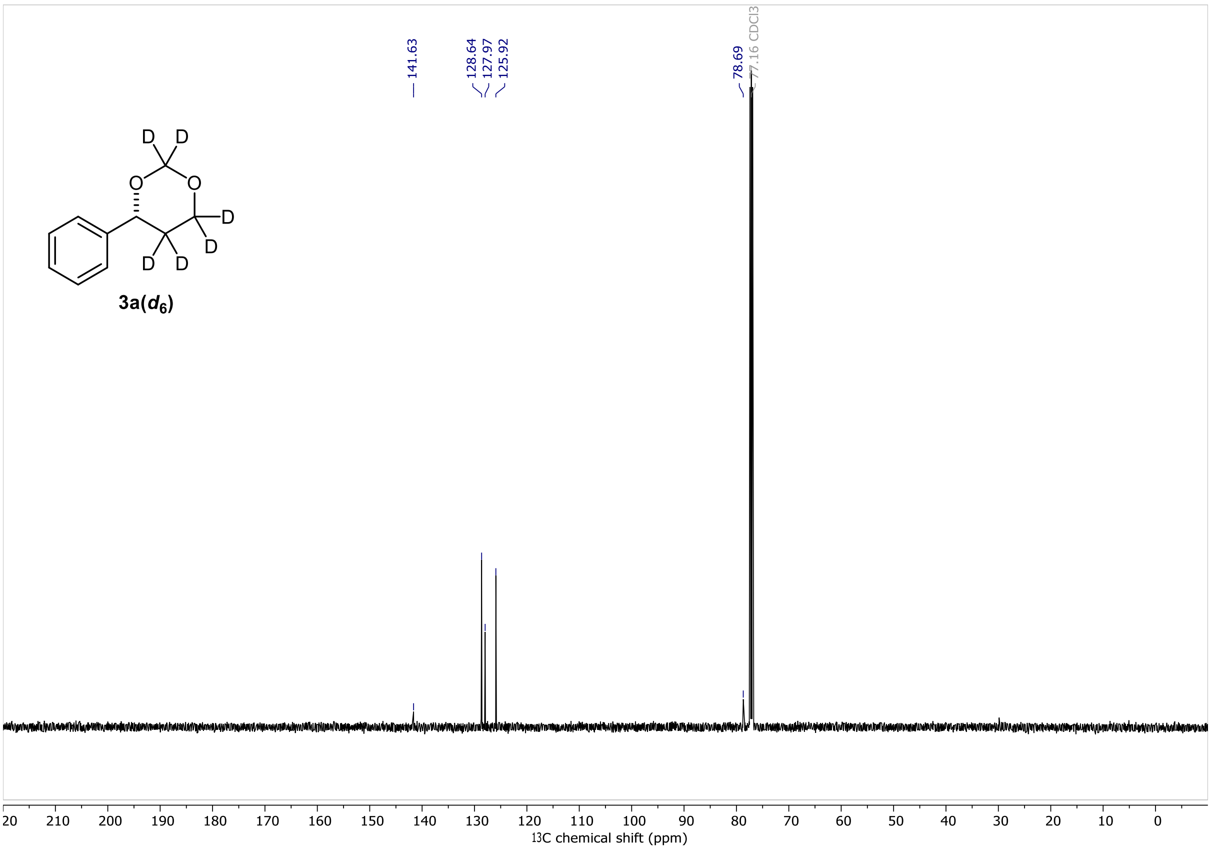

NMR spectra for compound **3a(*d*$_6$)**: ^{1}H (501 MHz) and ^{13}C (126 MHz), in $CDCl_3$.

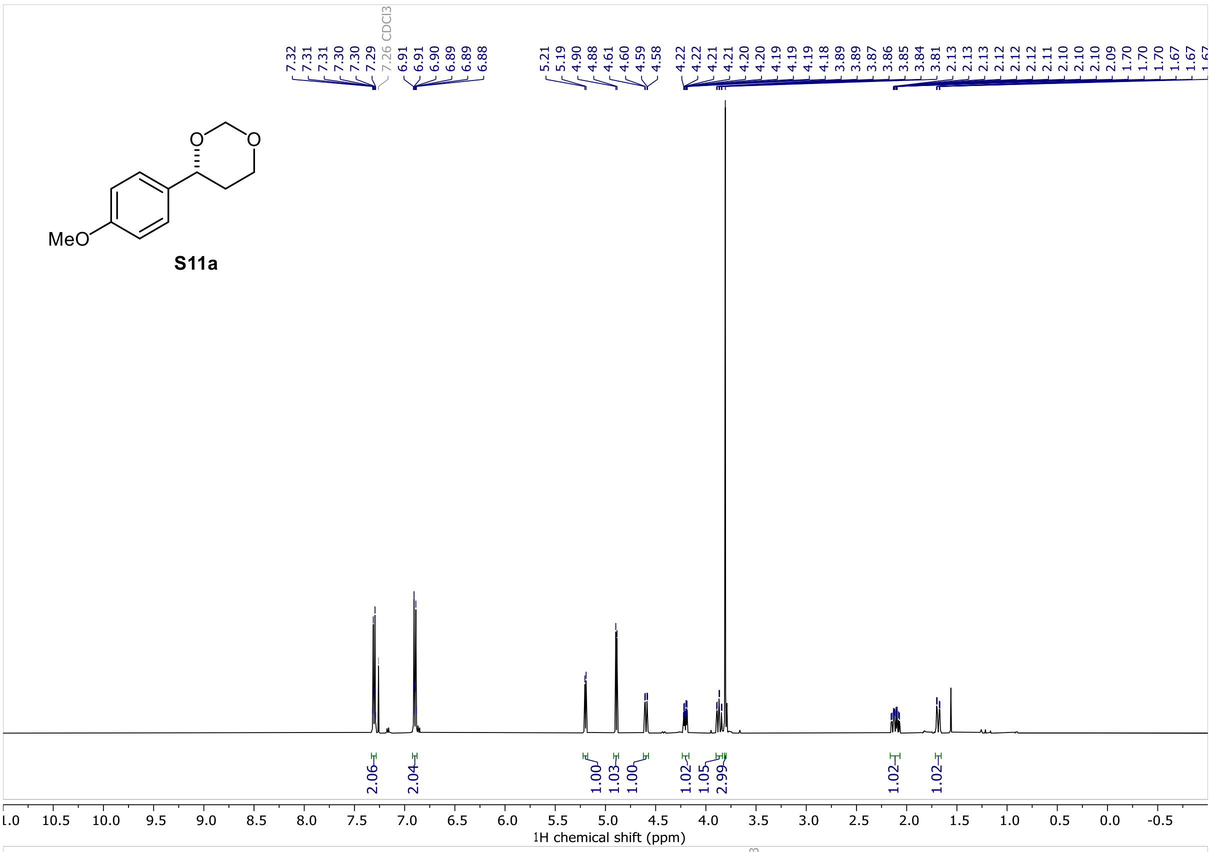

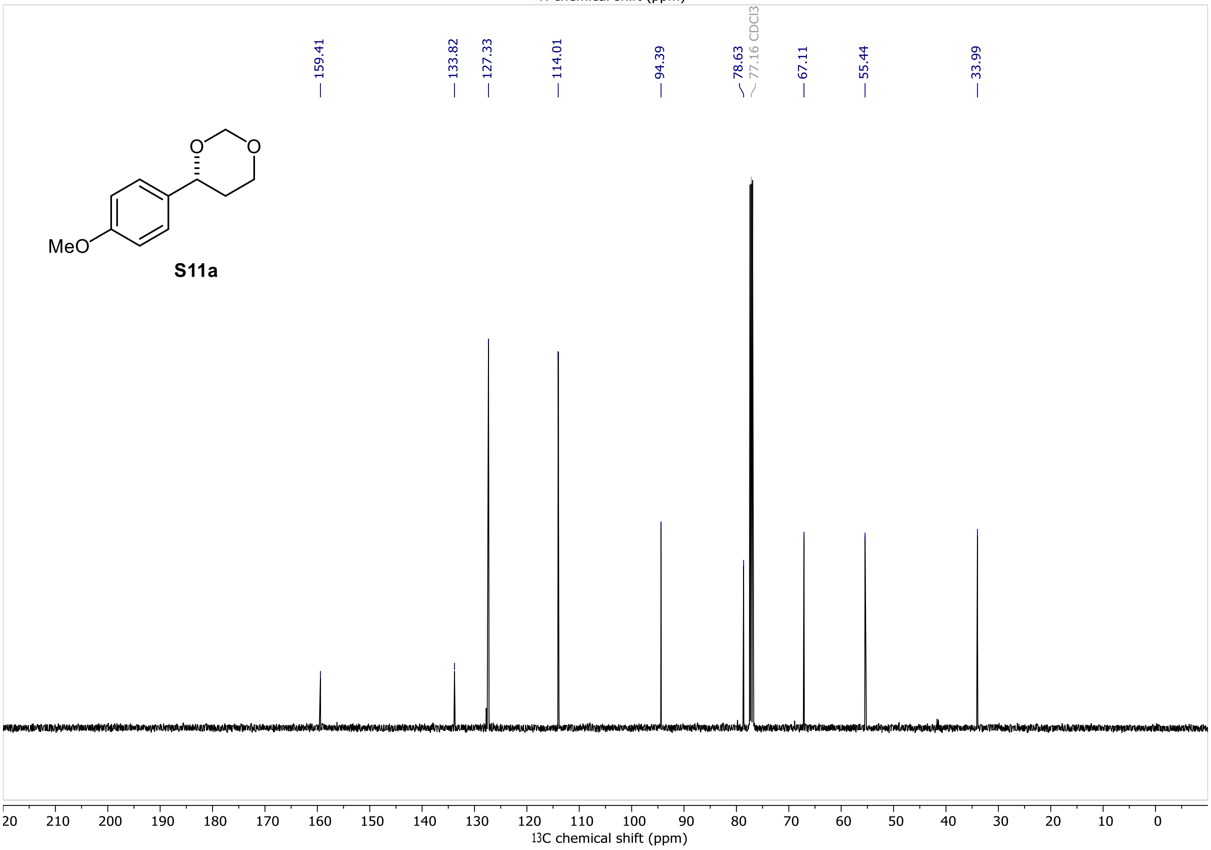

NMR spectra for compound **S11a**: ^{1}H (501 MHz) and ^{13}C (126 MHz), in $CDCl_3$.

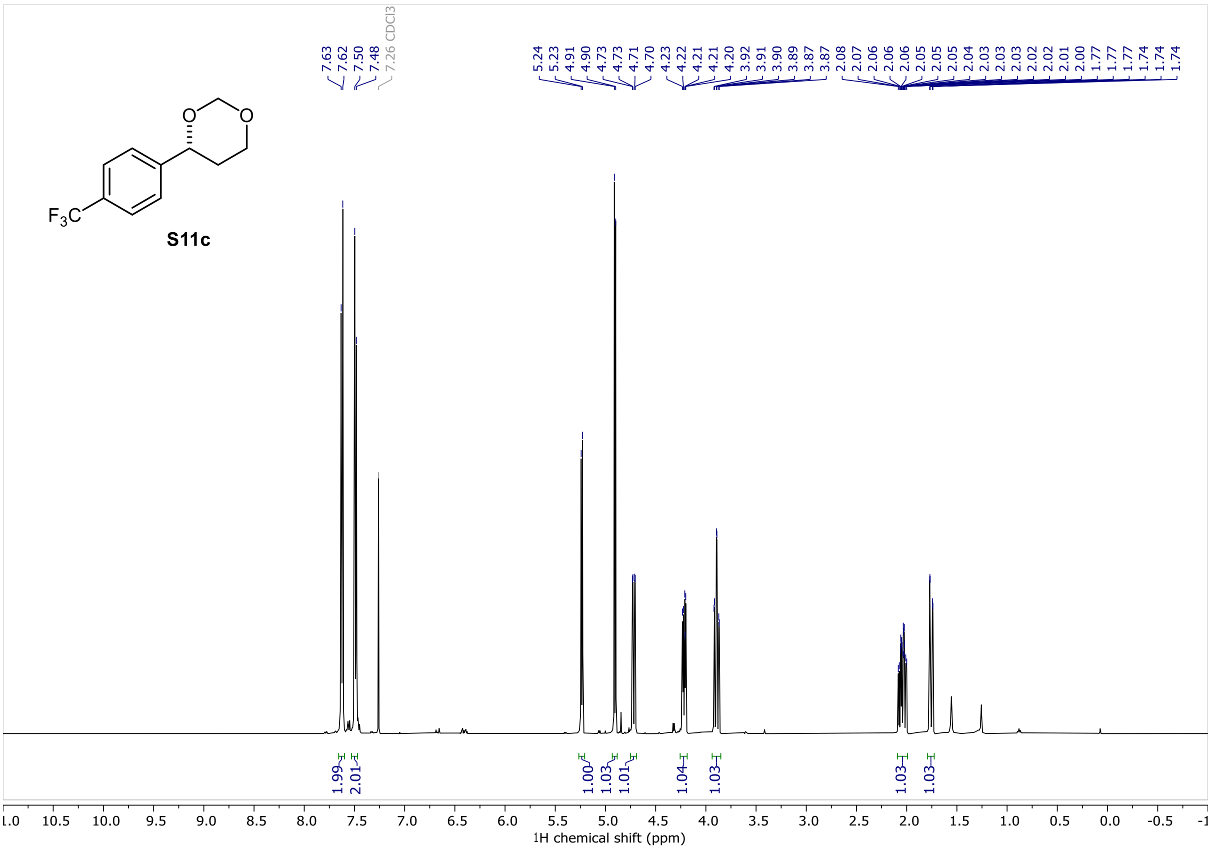

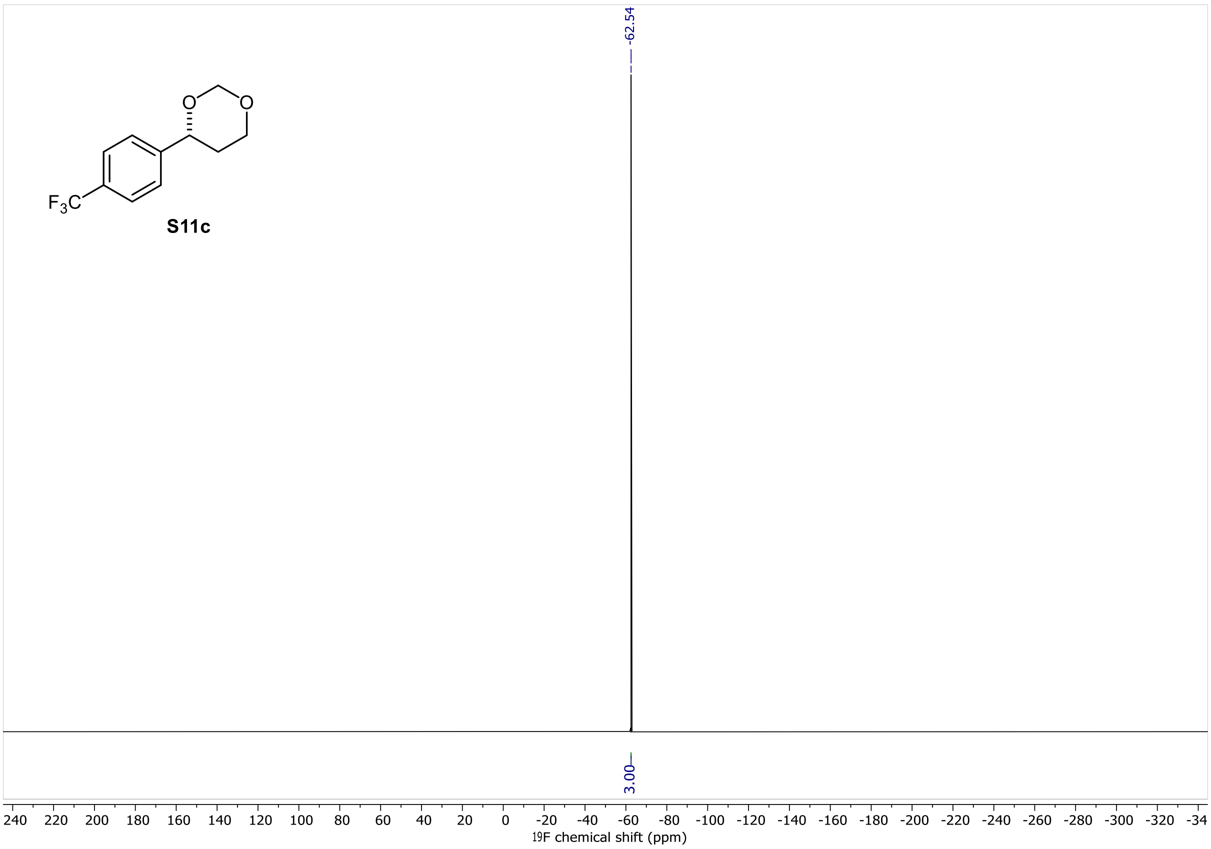

NMR spectra for compound **S11c**: ^{1}H (501 MHz) and ^{19}F (471 MHz), in $CDCl_3$.

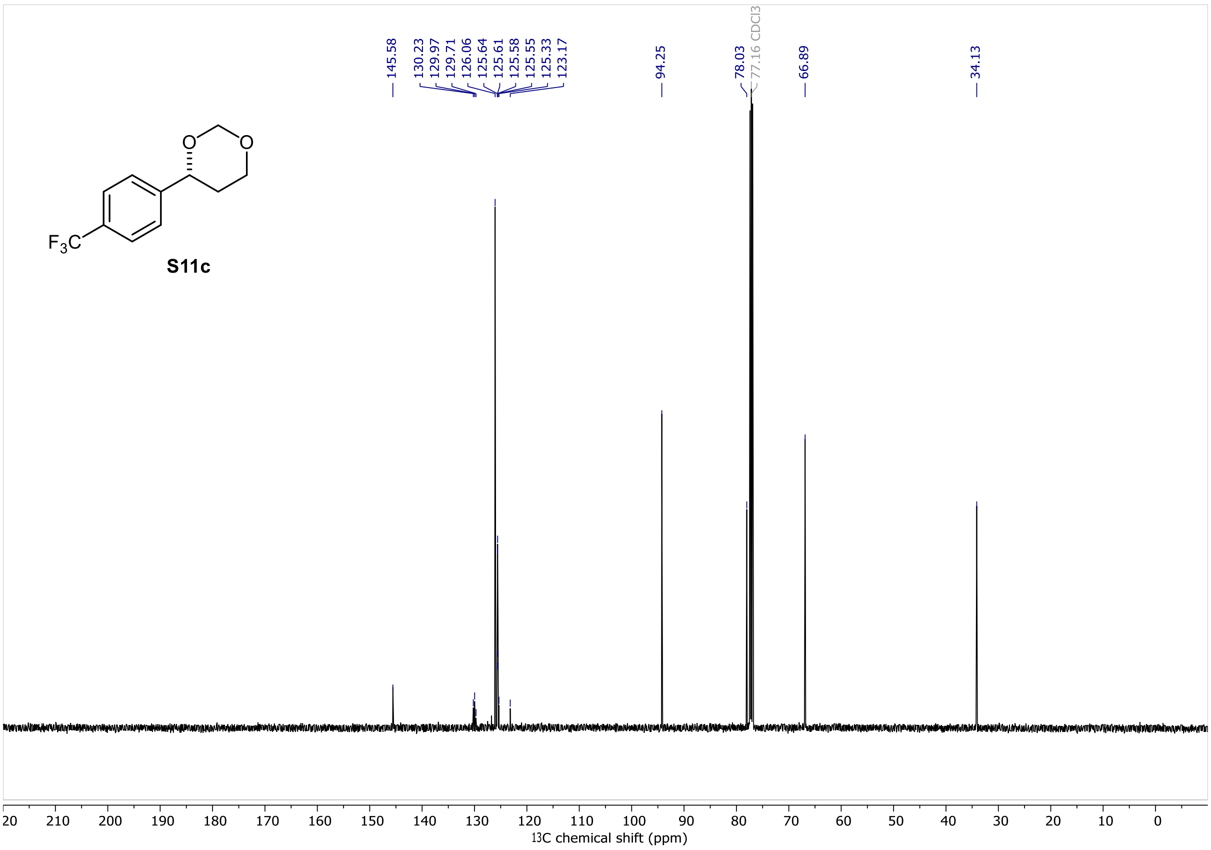

NMR spectra for compound **S11c** *(continuation)*: ^{13}C (126 MHz), in $CDCl_3$.

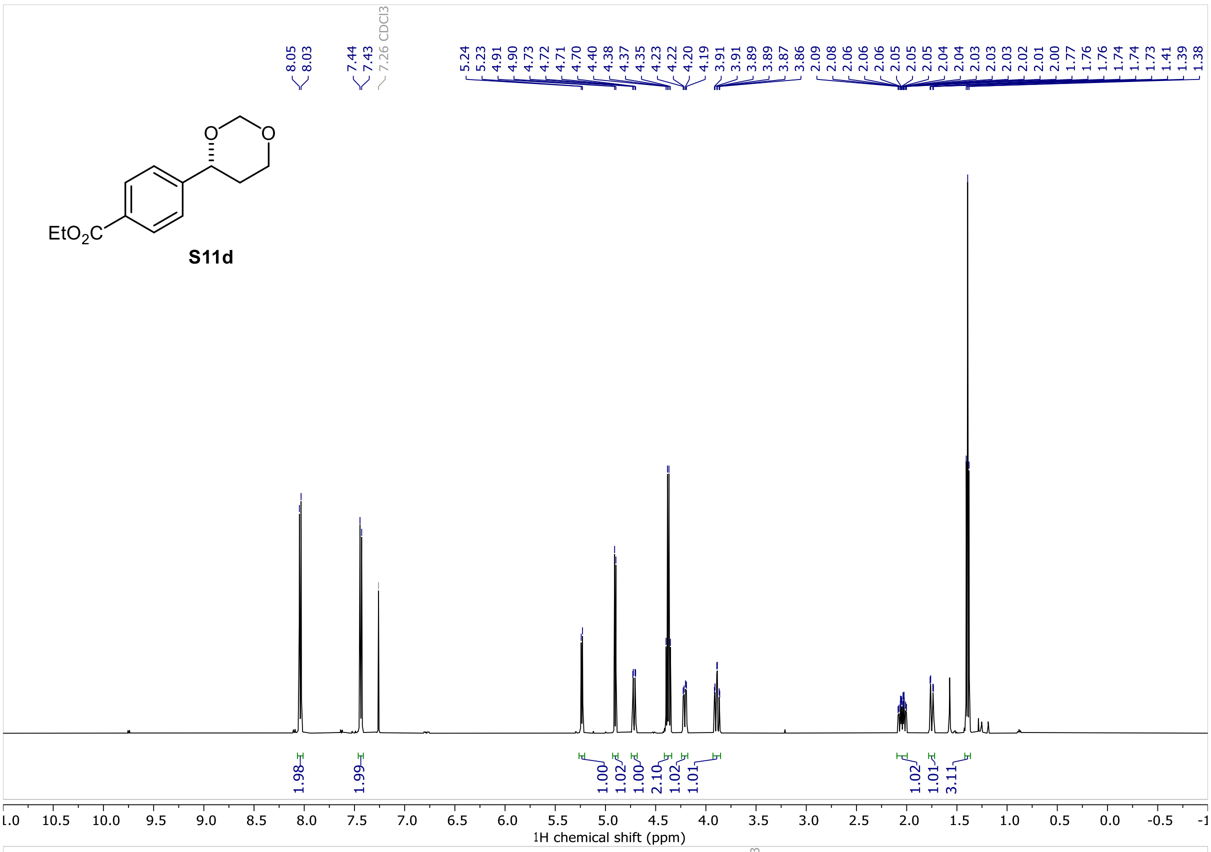

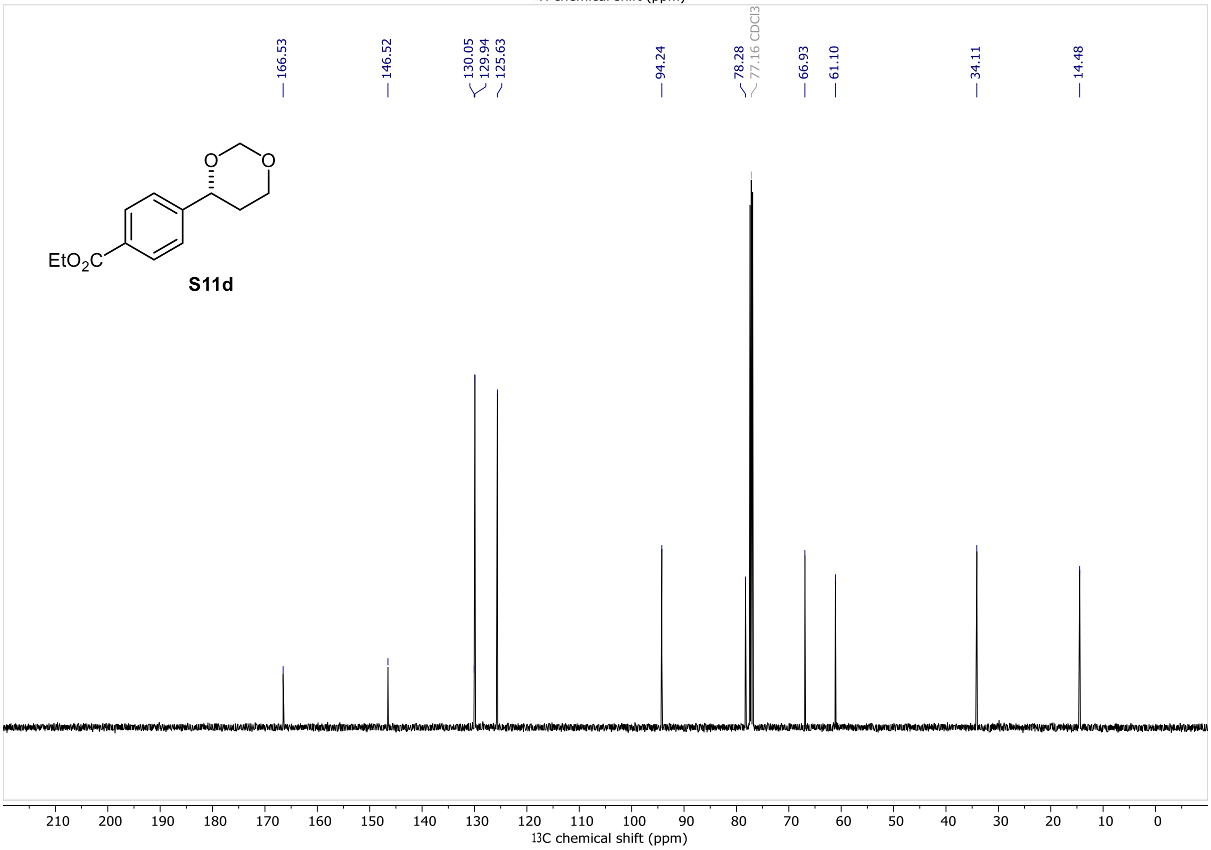

NMR spectra for compound **S11d**: ^{1}H (501 MHz) and ^{13}C (126 MHz), in $CDCl_3$.

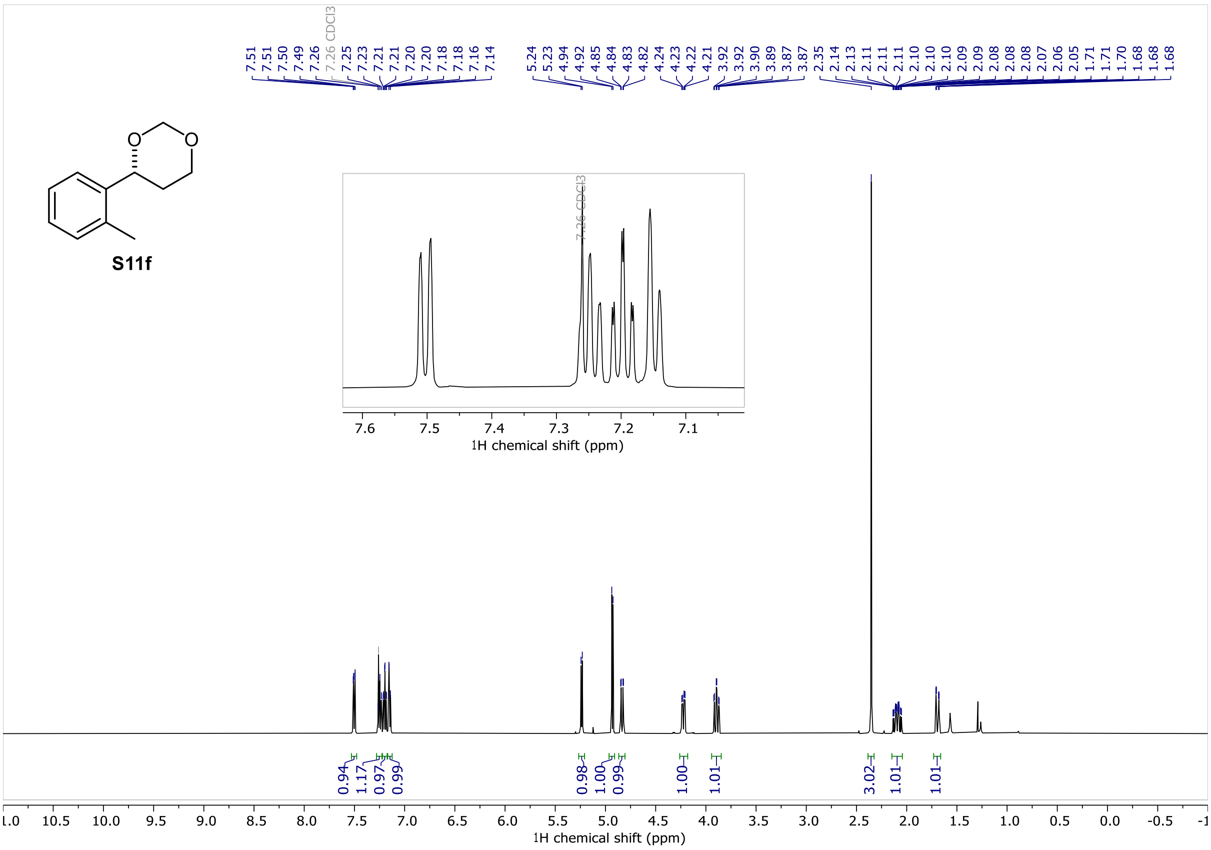

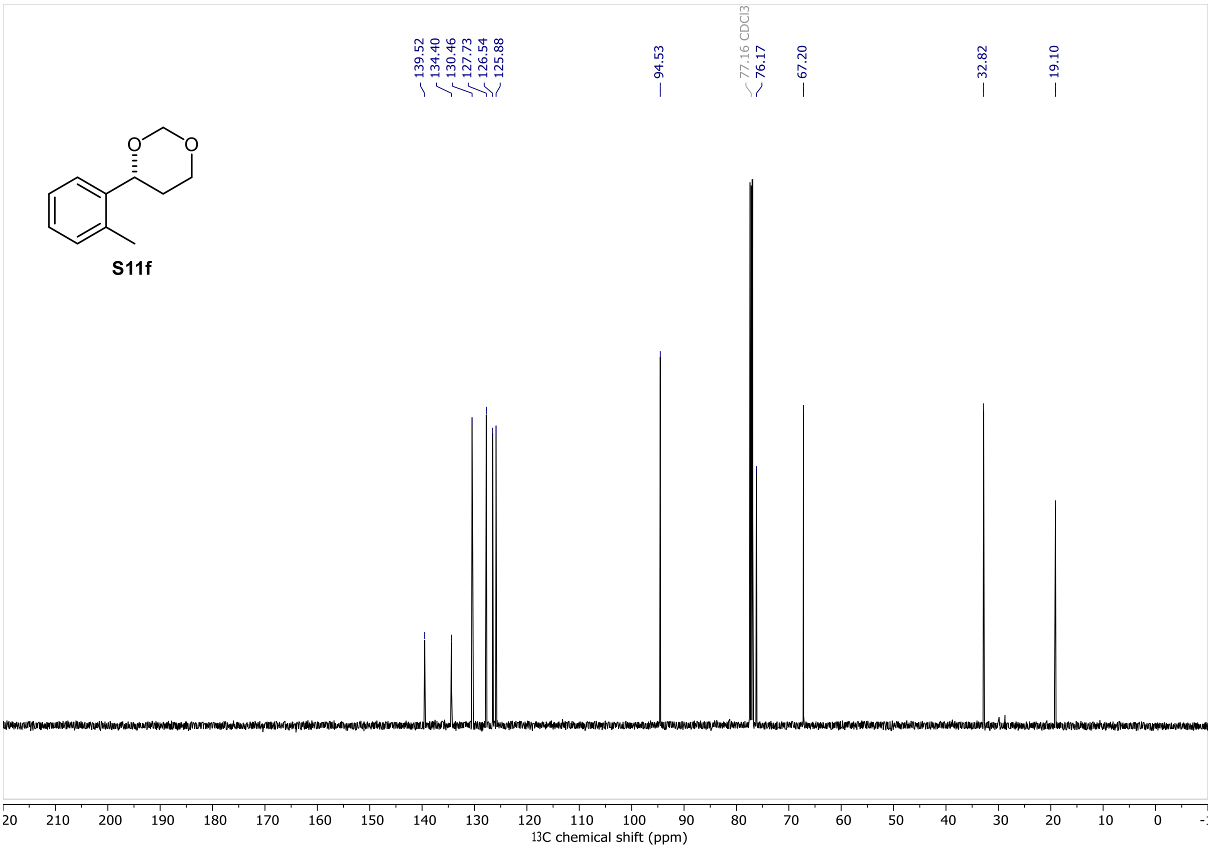

NMR spectra for compound **S11f**: ^{1}H (501 MHz) and ^{13}C (126 MHz), in $CDCl_3$.

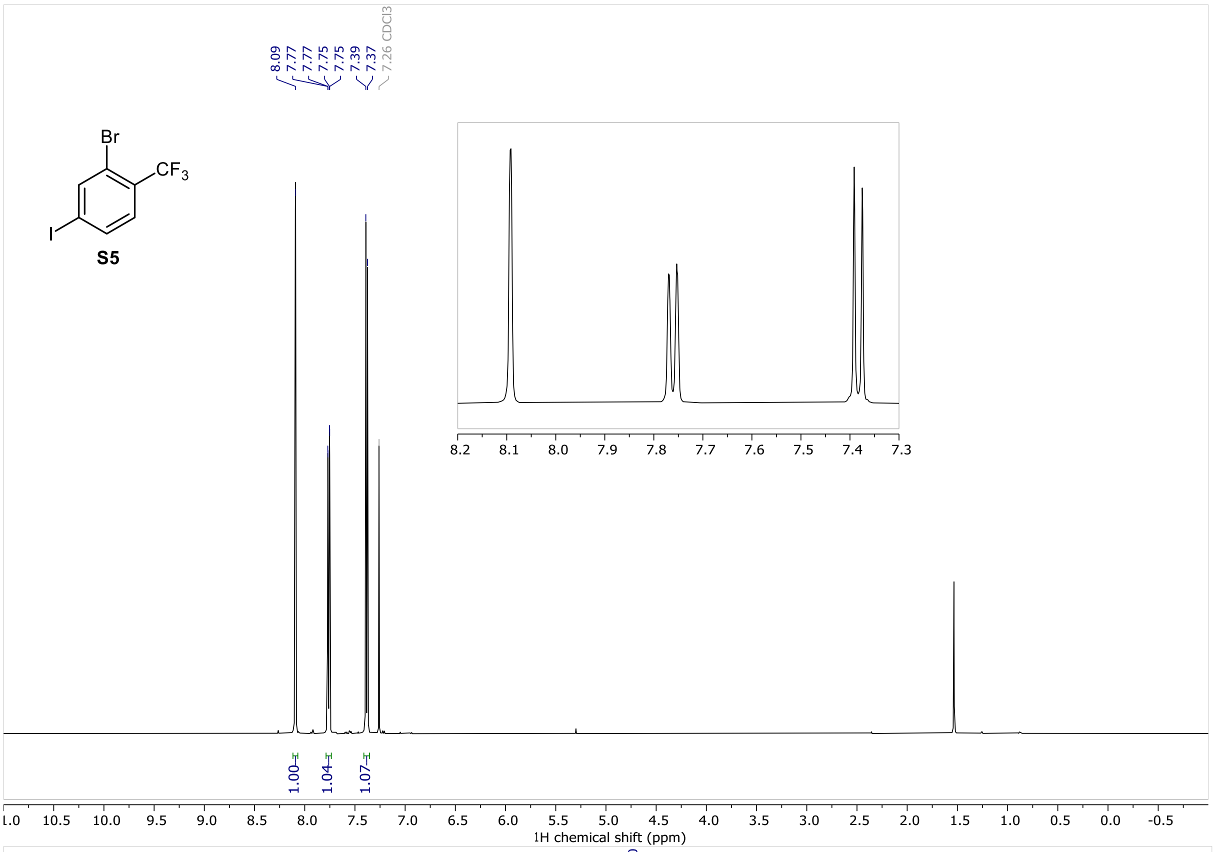

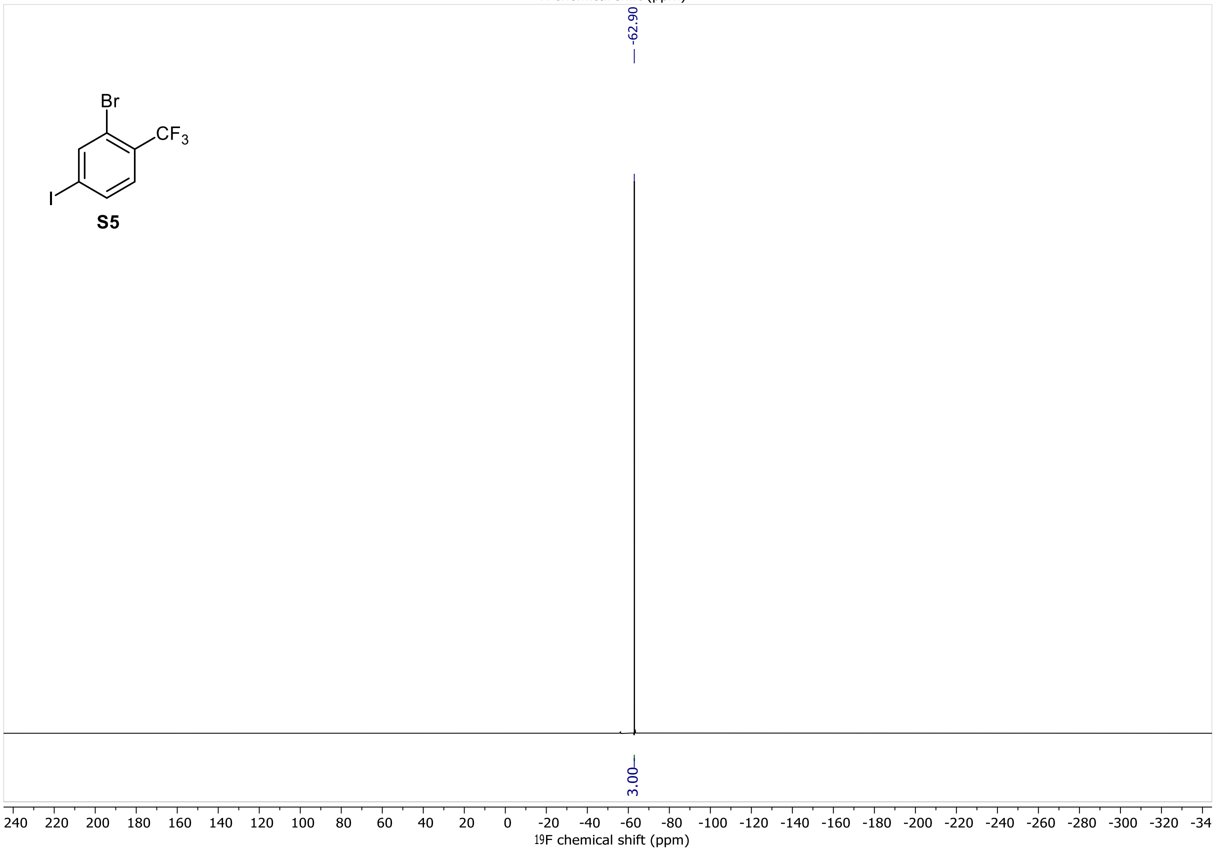

NMR spectra for compound **S5**: ^{1}H (501 MHz) and ^{19}F (471 MHz), in $CDCl_3$.

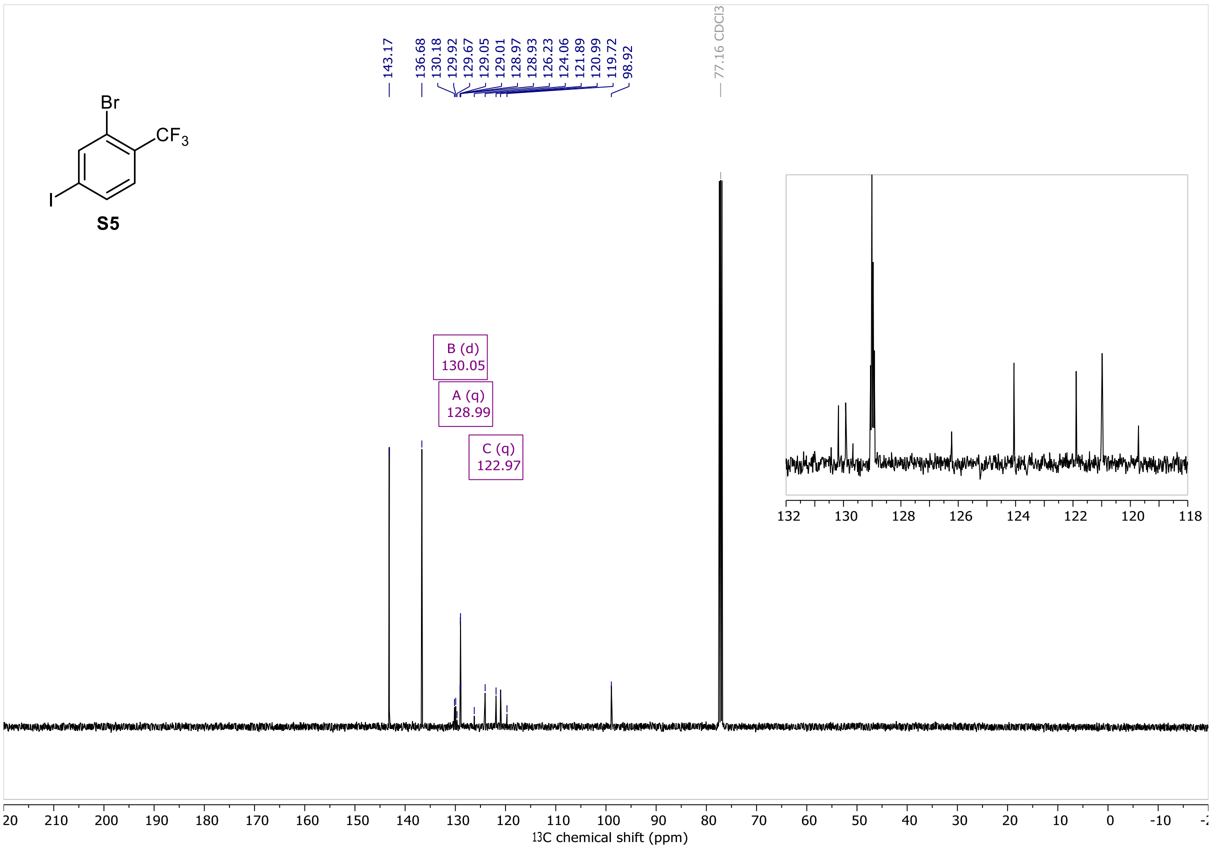

NMR spectra for compound **S5** *(continuation)*: ^{13}C (126 MHz), in $CDCl_3$.

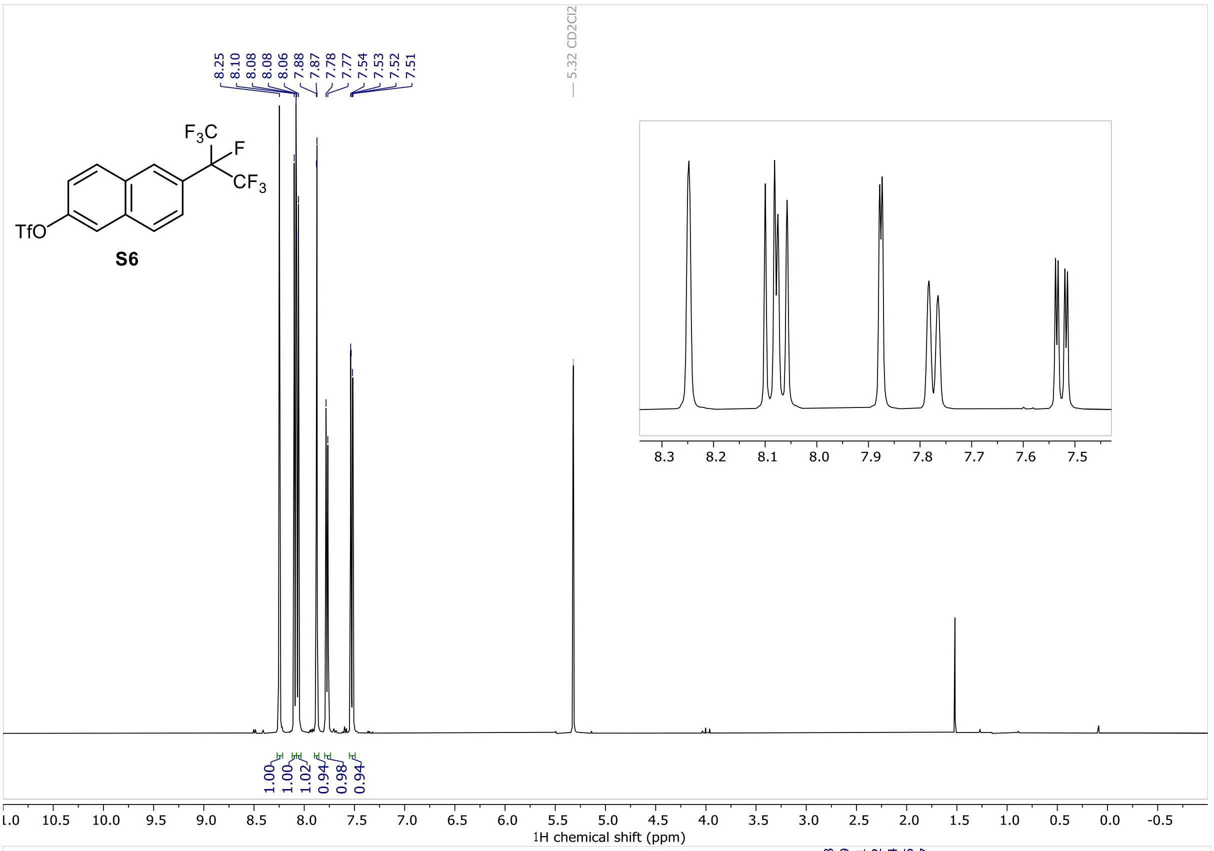

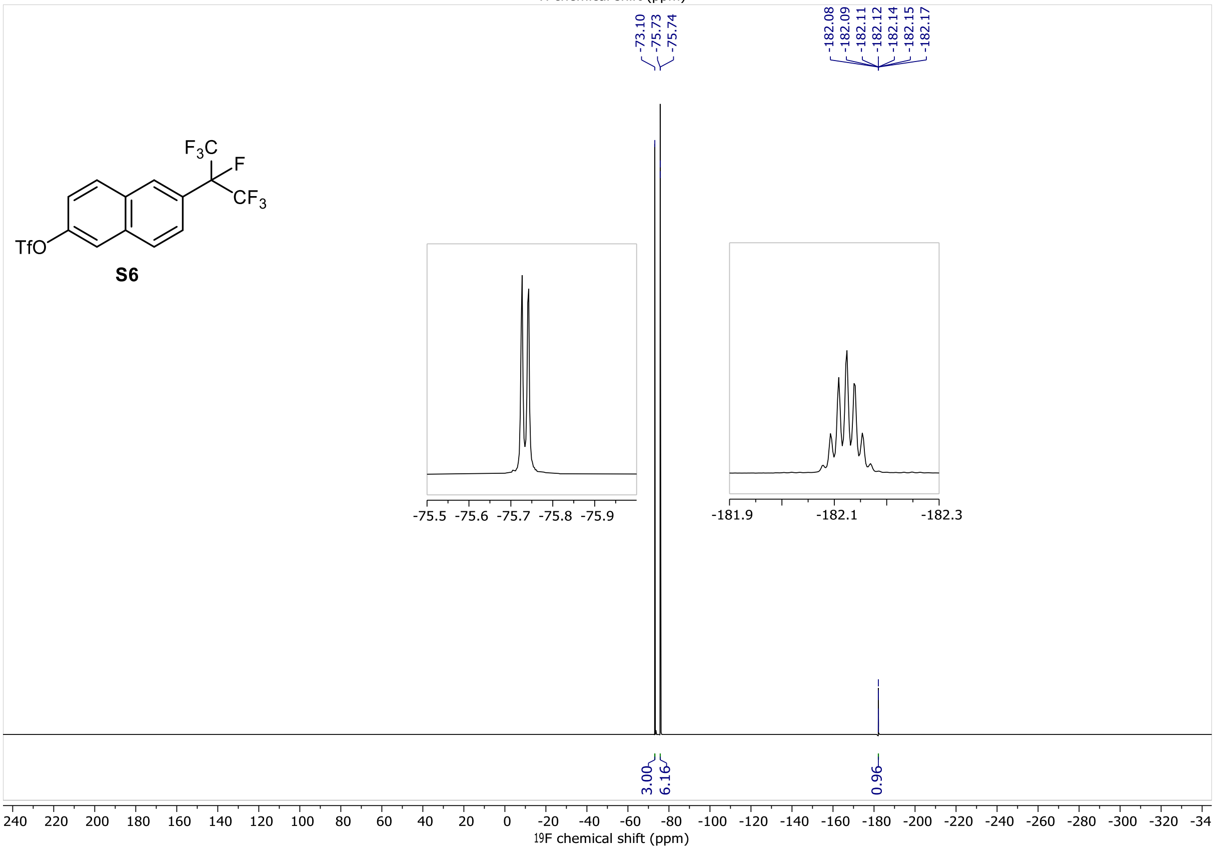

NMR spectra for compound **S6**: ^{1}H (501 MHz) and ^{19}F (471 MHz), in CD_2Cl_2.

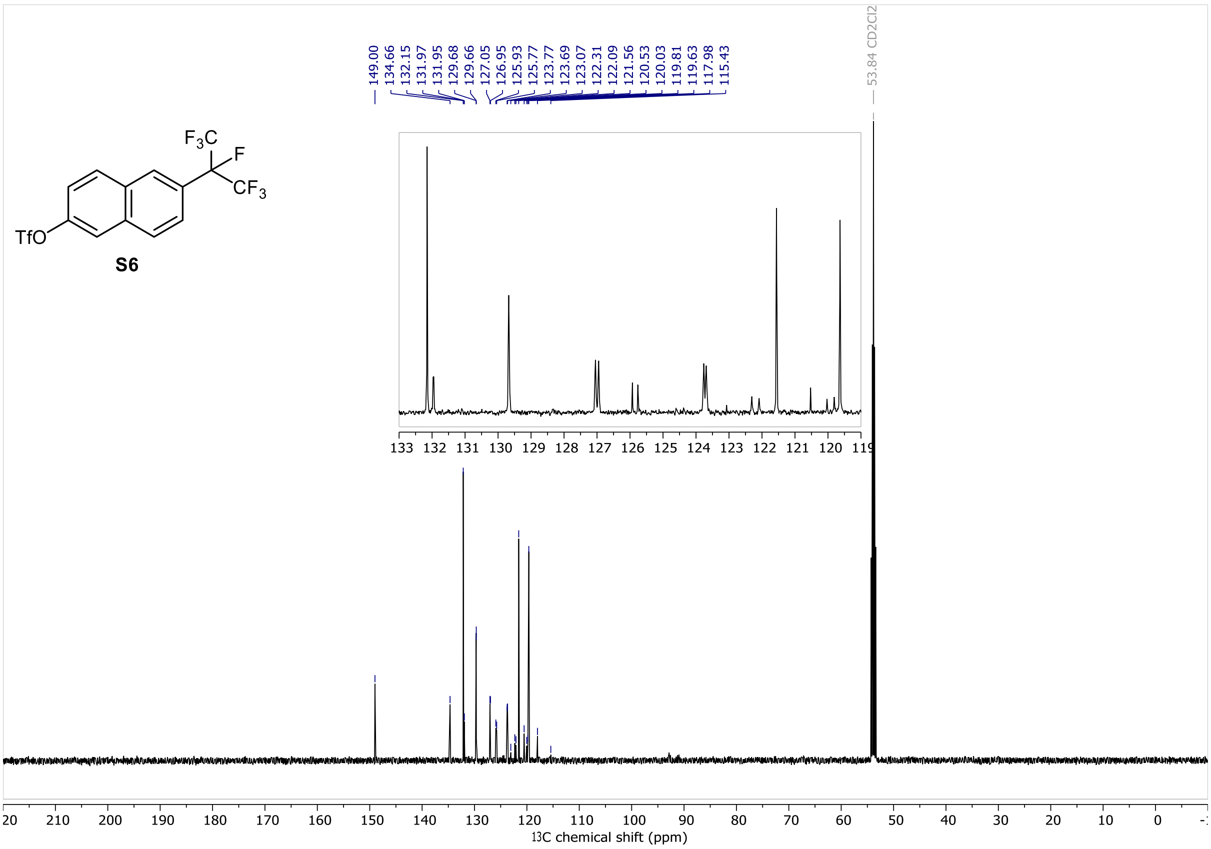

NMR spectra for compound **S6** *(continuation)*: ^{13}C (126 MHz), in CD_2Cl_2.

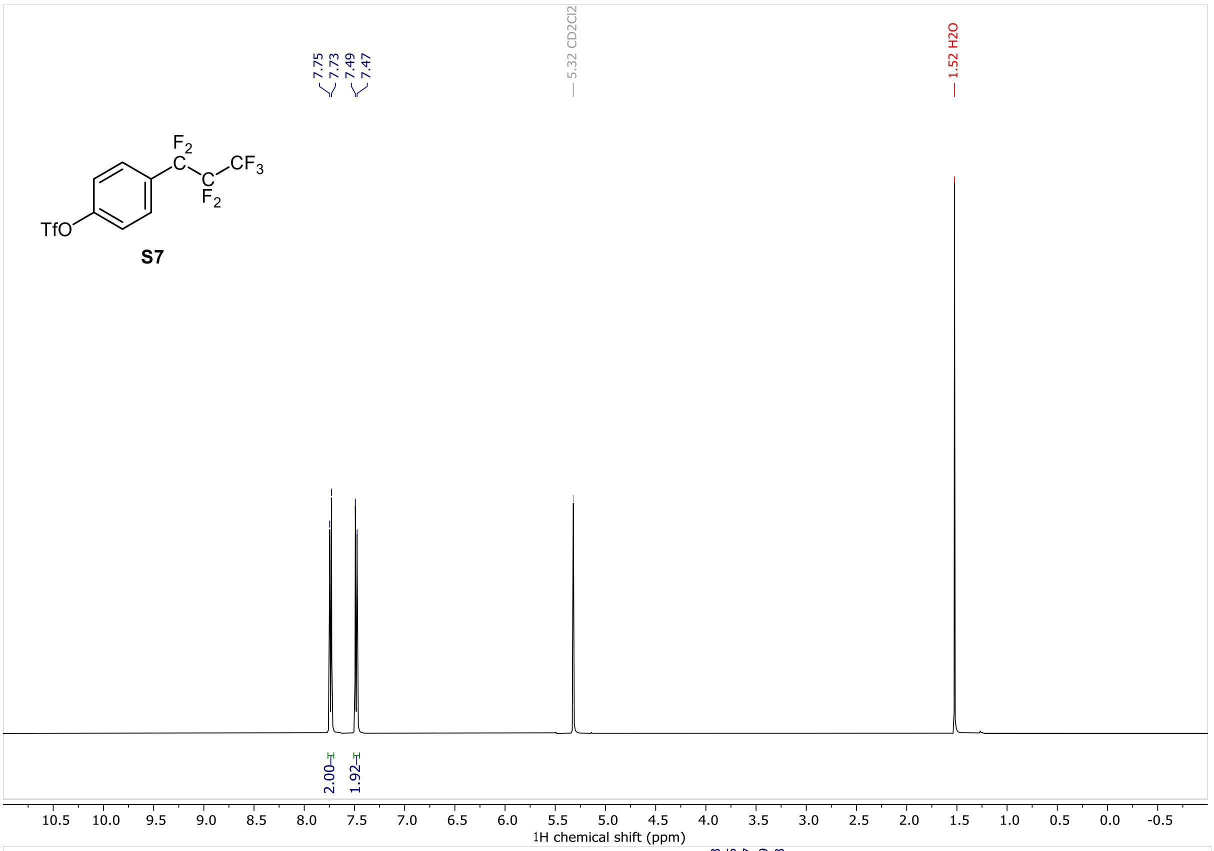

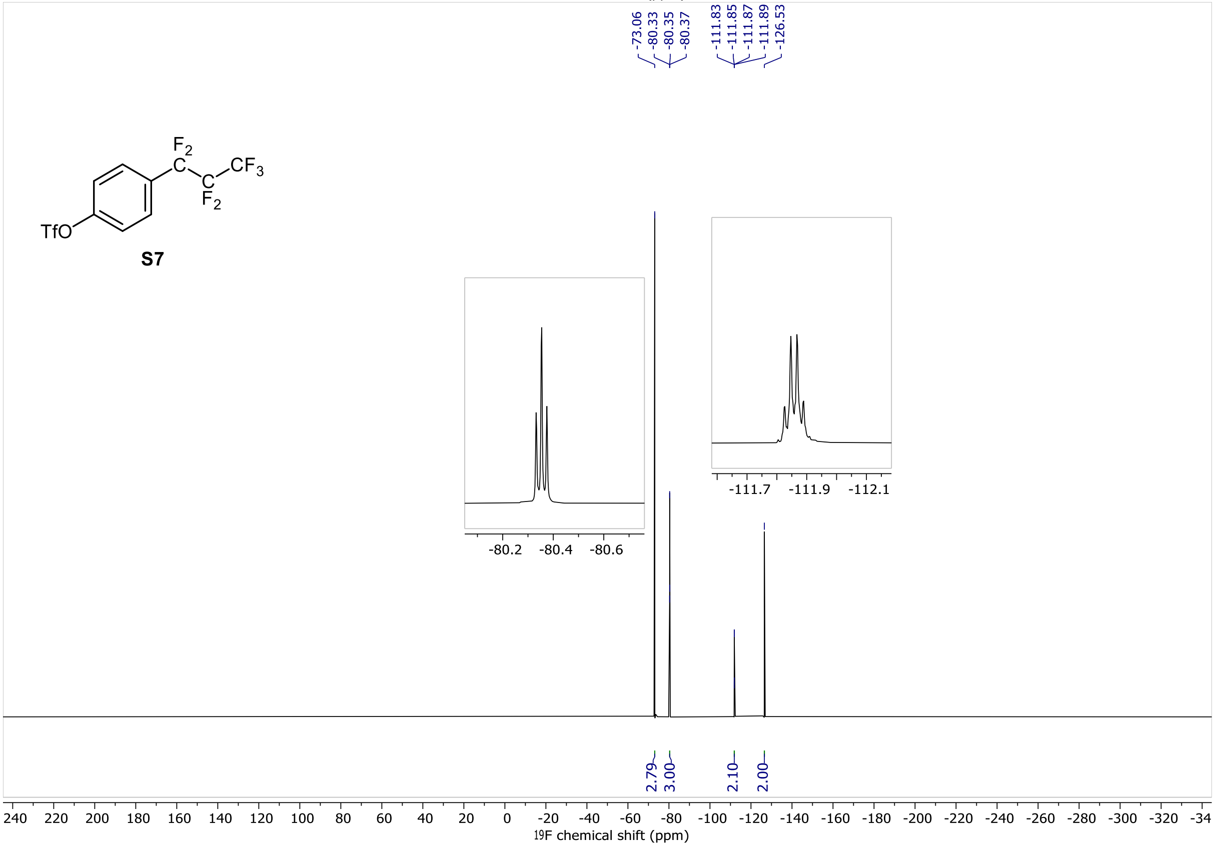

NMR spectra for compound **S7**: ^{1}H (501 MHz) and ^{19}F (471 MHz), in CD_2Cl_2.

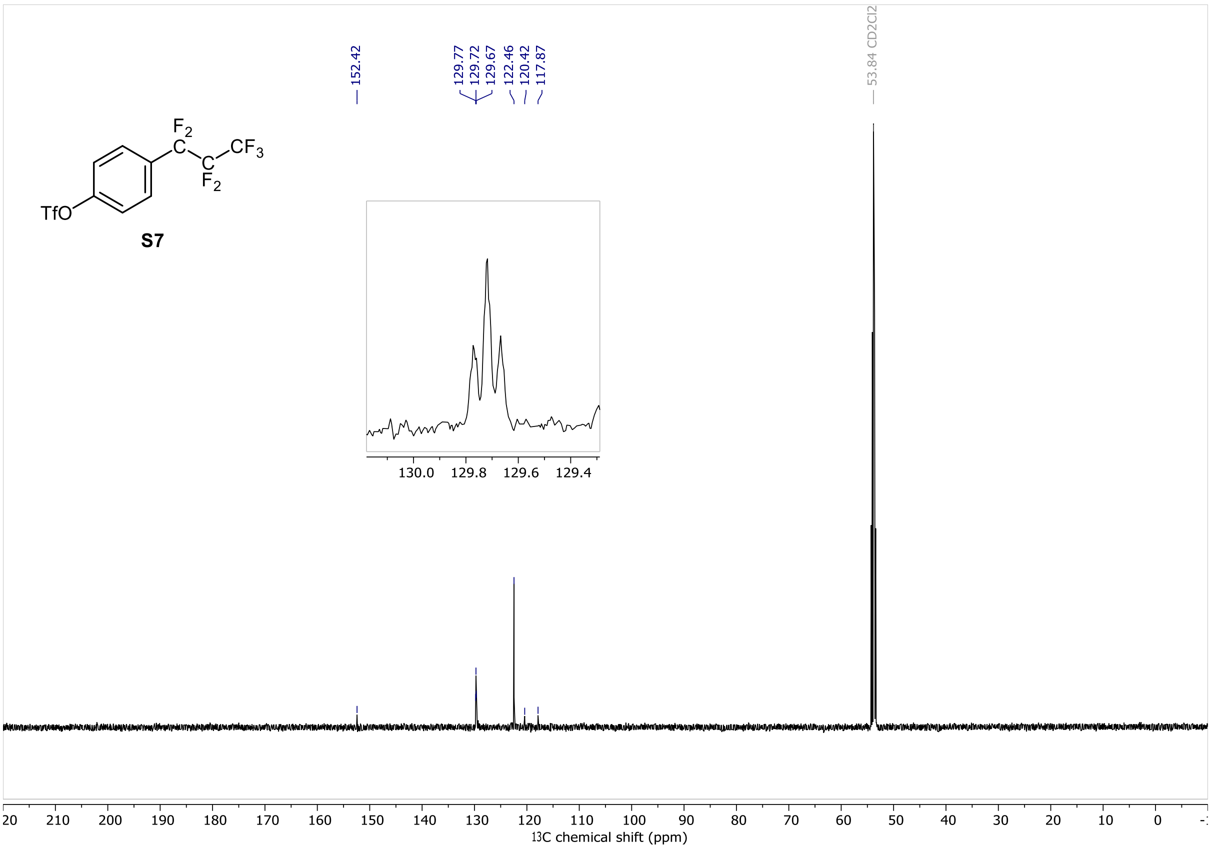

NMR spectra for compound **S7** *(continuation)*: ^{13}C (126 MHz), in CD_2Cl_2.

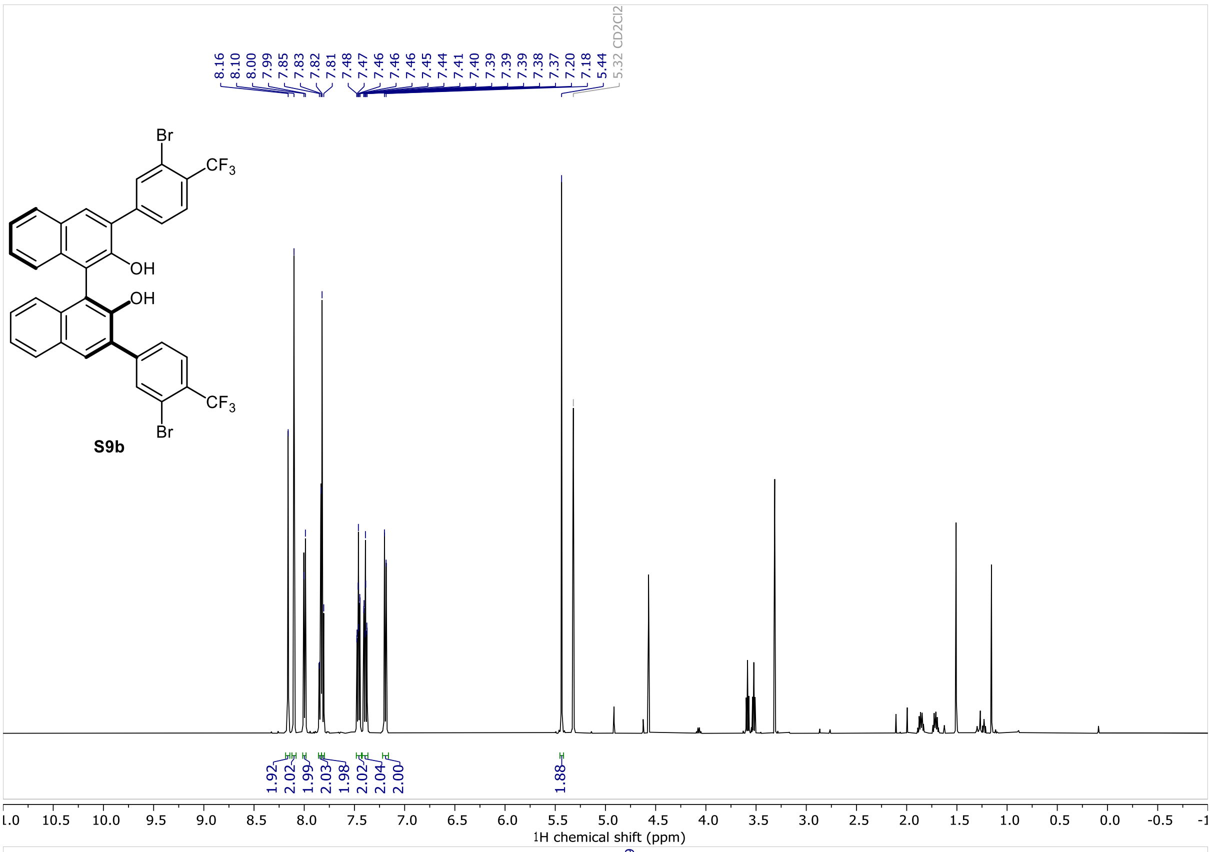

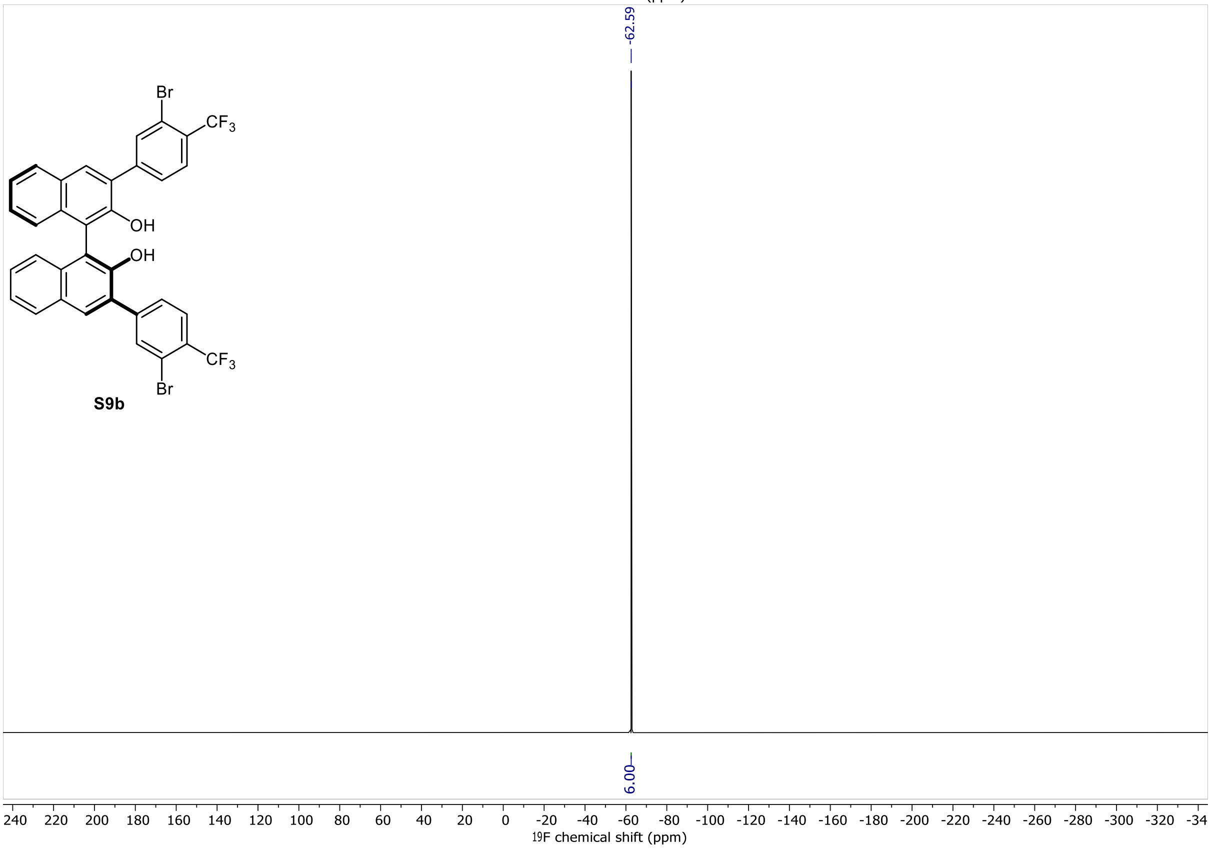

NMR spectra for compound **S9b**: ^{1}H (501 MHz) and ^{19}F (471 MHz), in CD_2Cl_2.

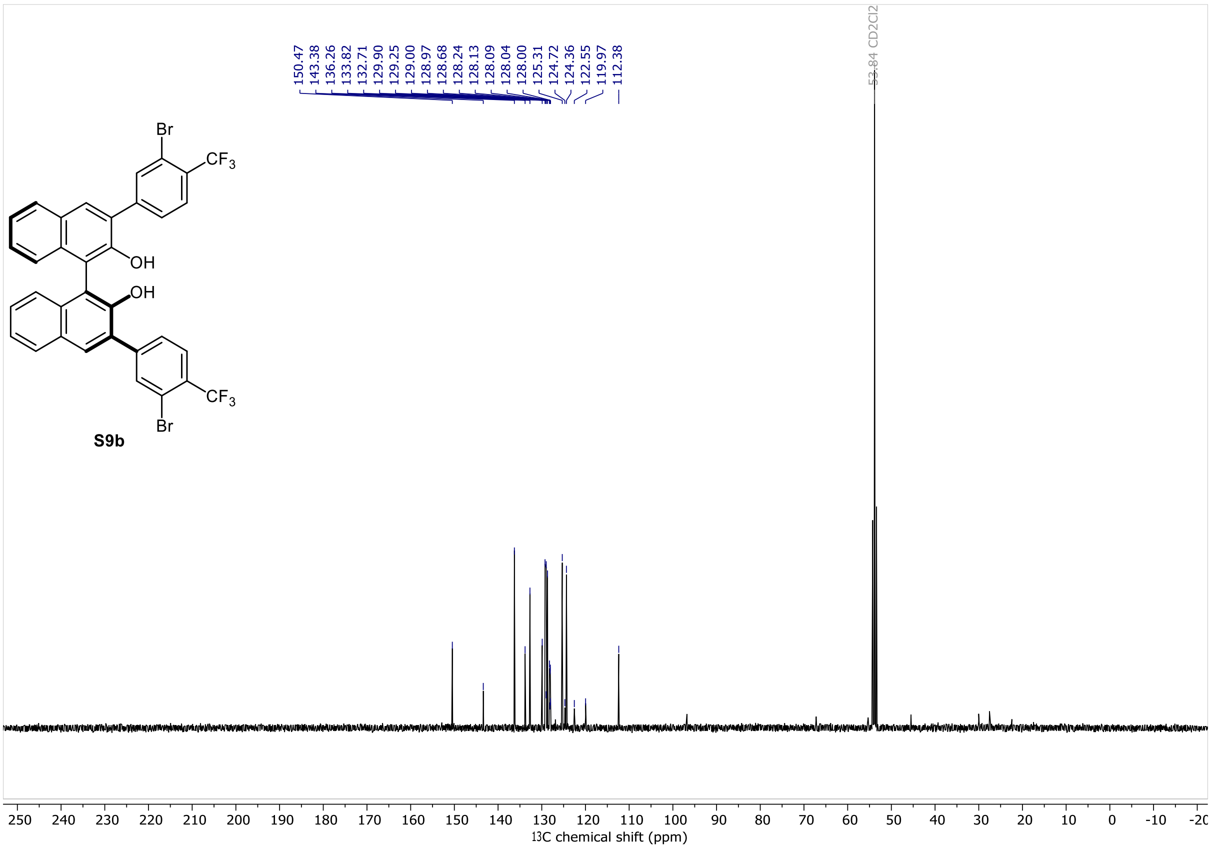

NMR spectra for compound **S9b** *(continuation)*: ^{13}C (126 MHz), in CD_2Cl_2.

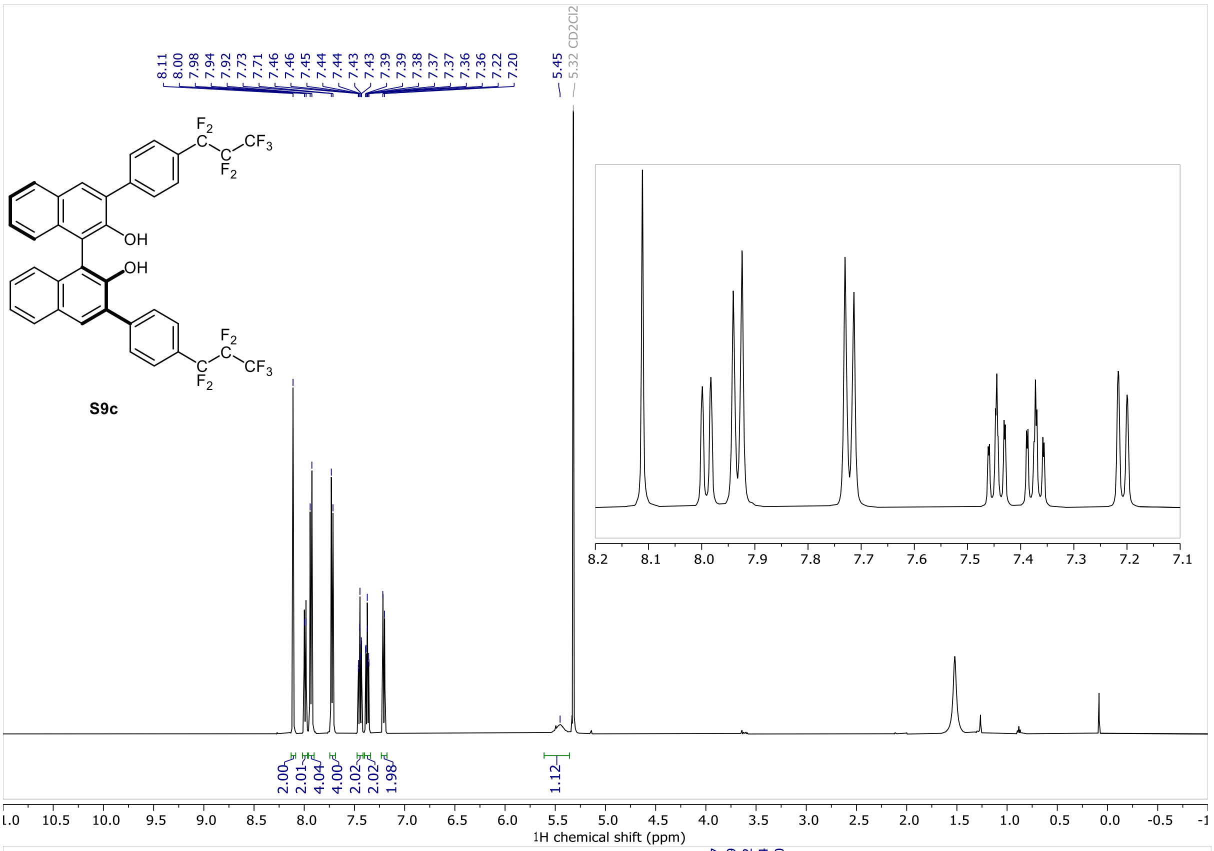

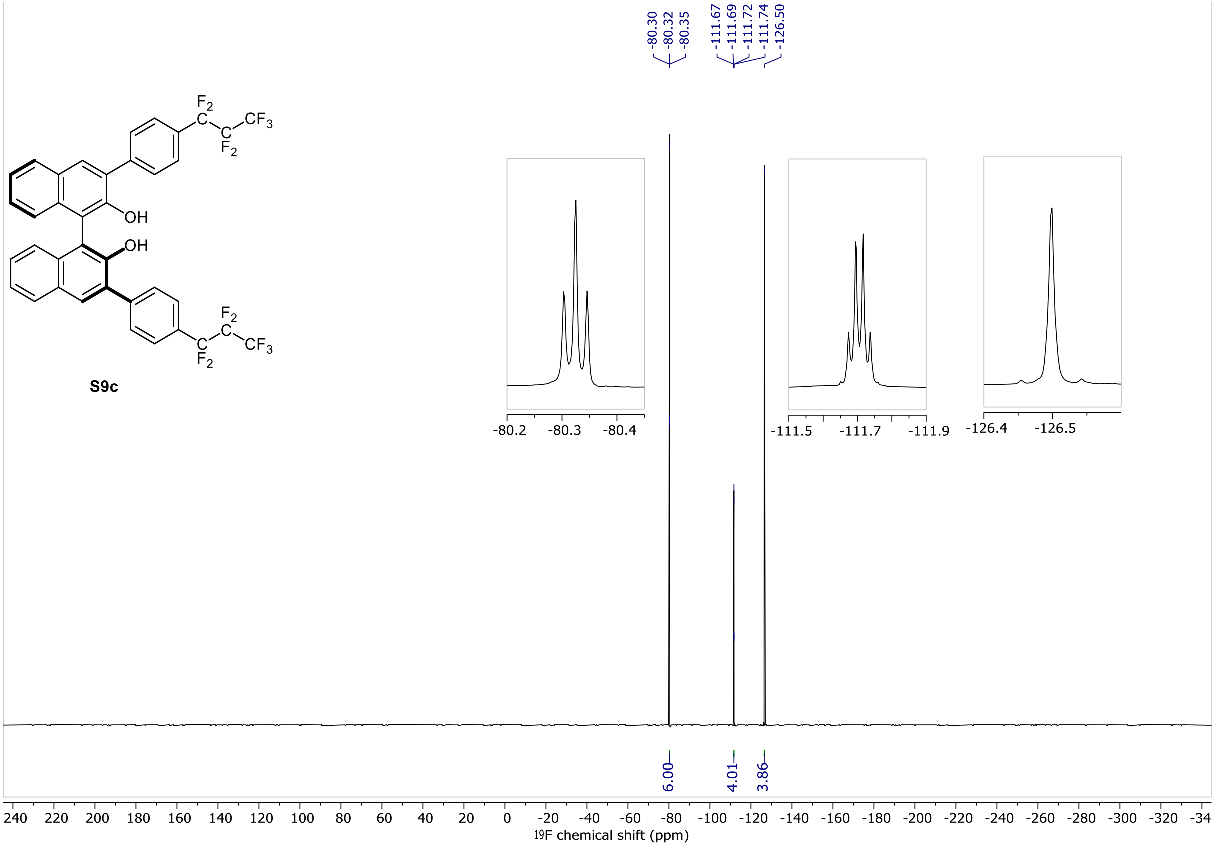

NMR spectra for compound **S9c**: ^{1}H (501 MHz) and ^{19}F (471 MHz), in CD_2Cl_2.

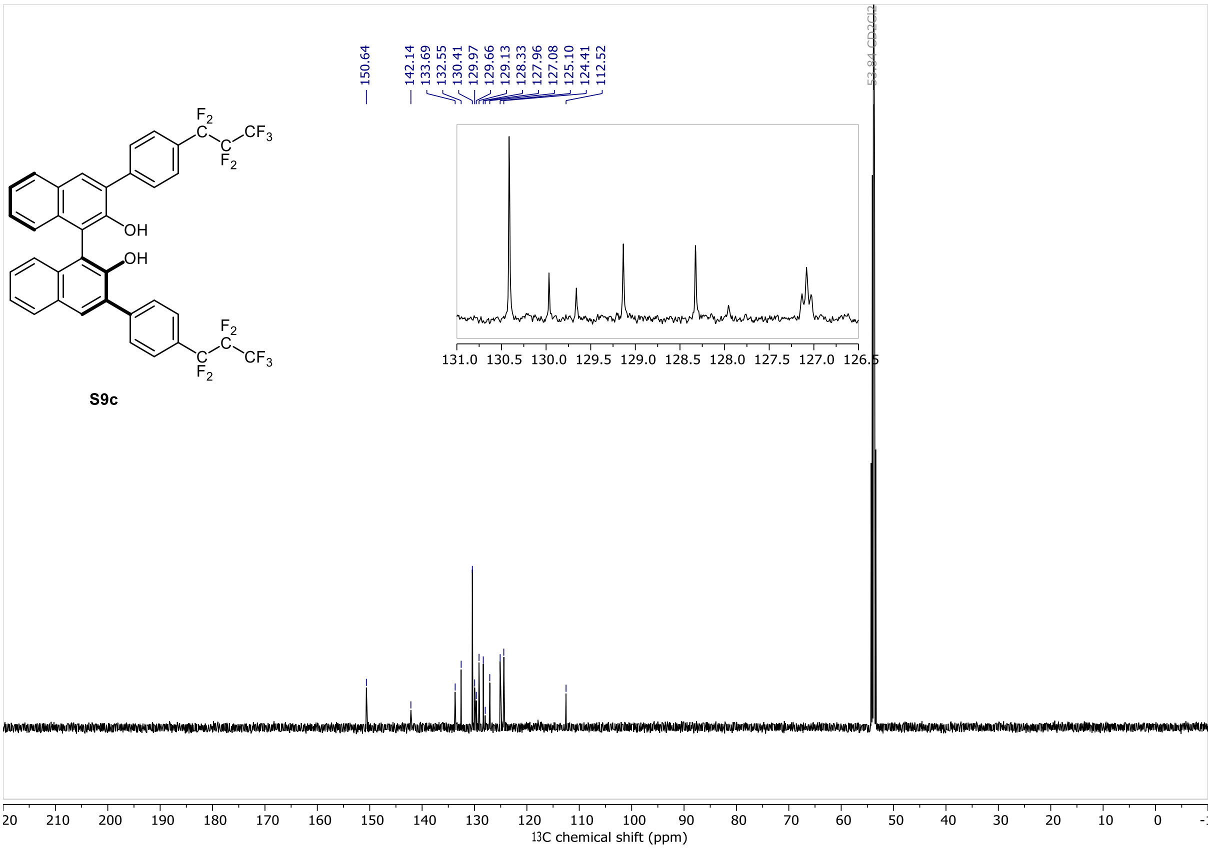

NMR spectra for compound **S9c** *(continuation)*: ^{13}C (126 MHz), in CD_2Cl_2.

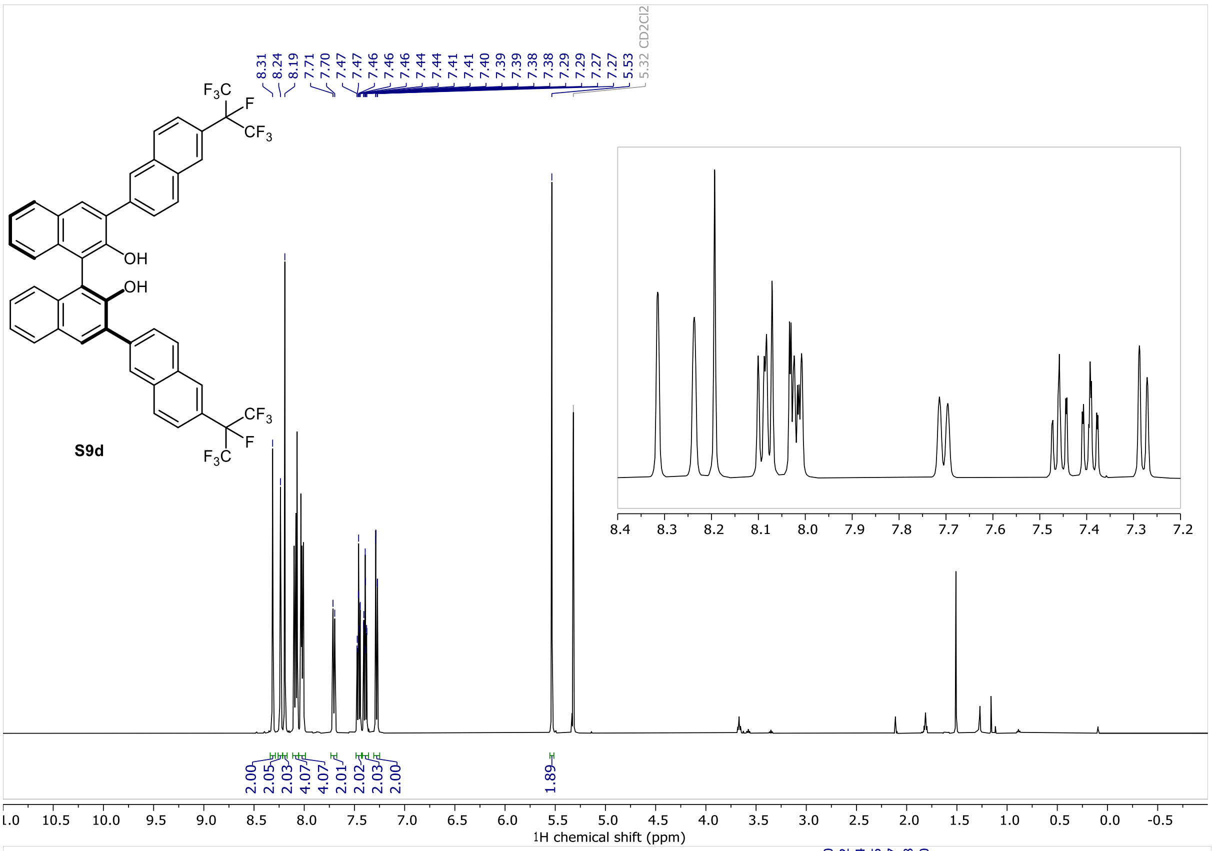

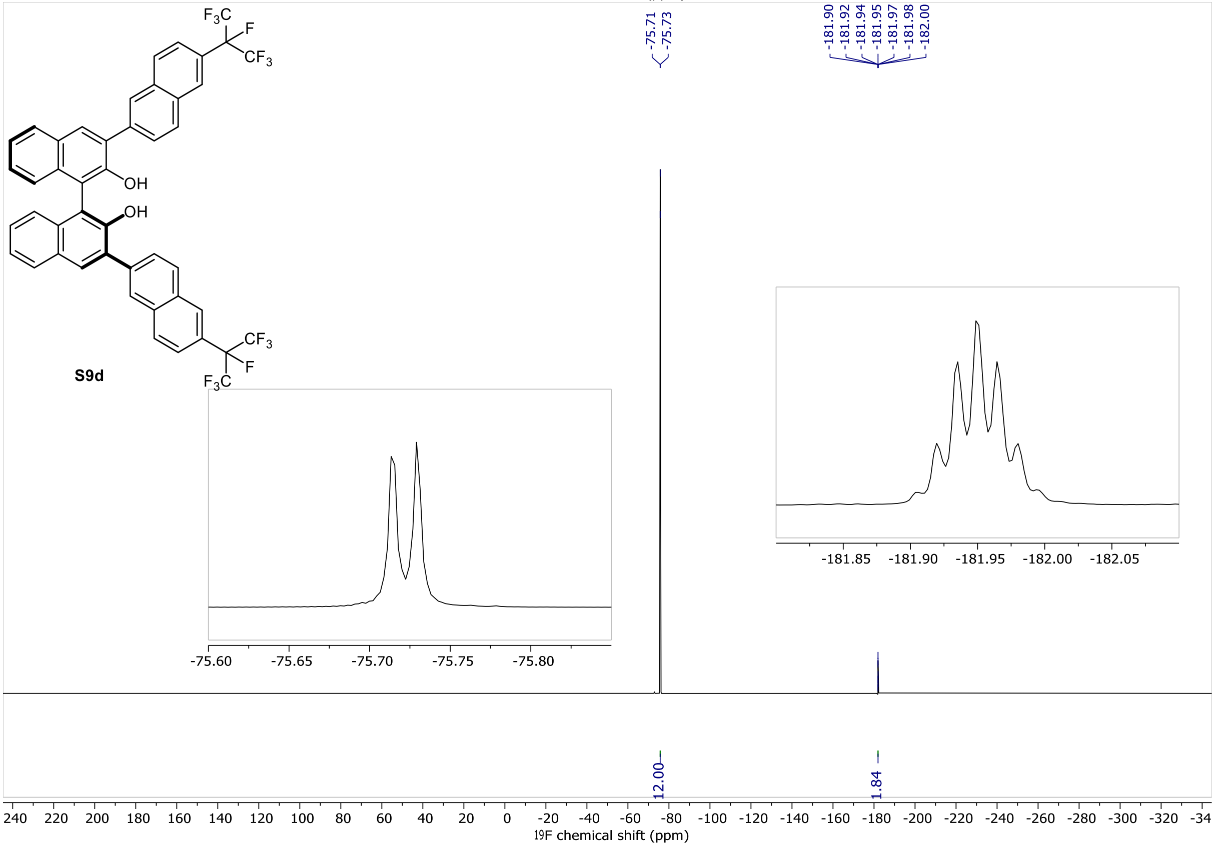

NMR spectra for compound **S9d**: ^{1}H (501 MHz) and ^{19}F (471 MHz), in CD_2Cl_2.

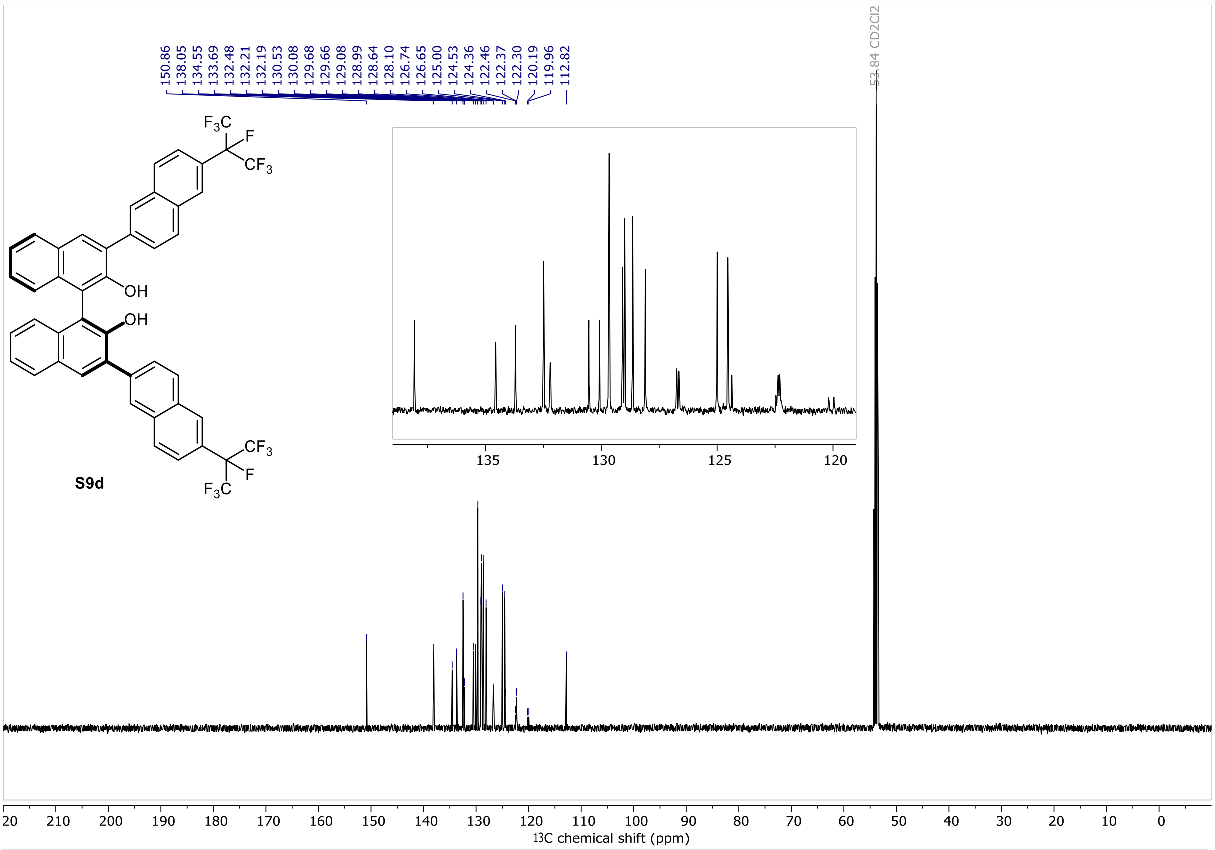

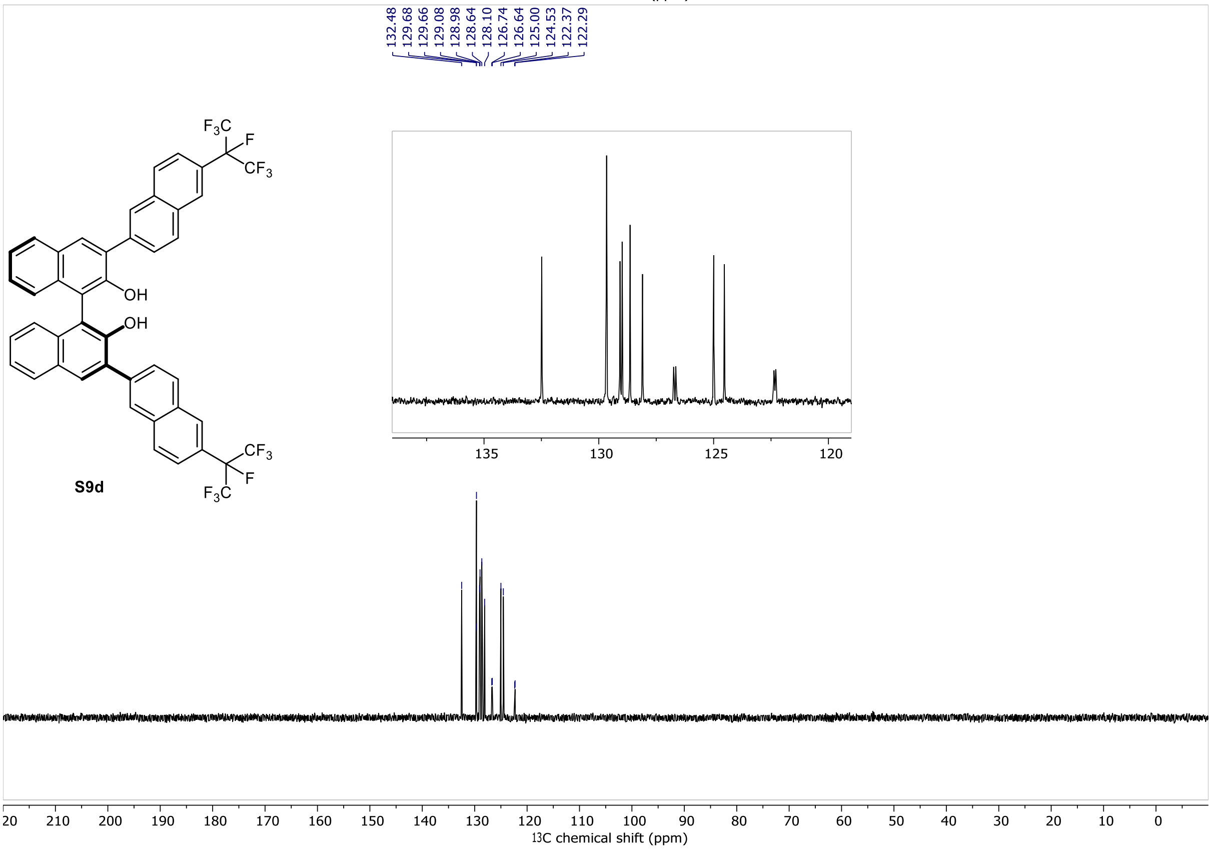

NMR spectra for compound **S9d** *(continuation)*: ^{13}C (126 MHz) and DEPT-135 (126 MHz), in CD_2Cl_2.

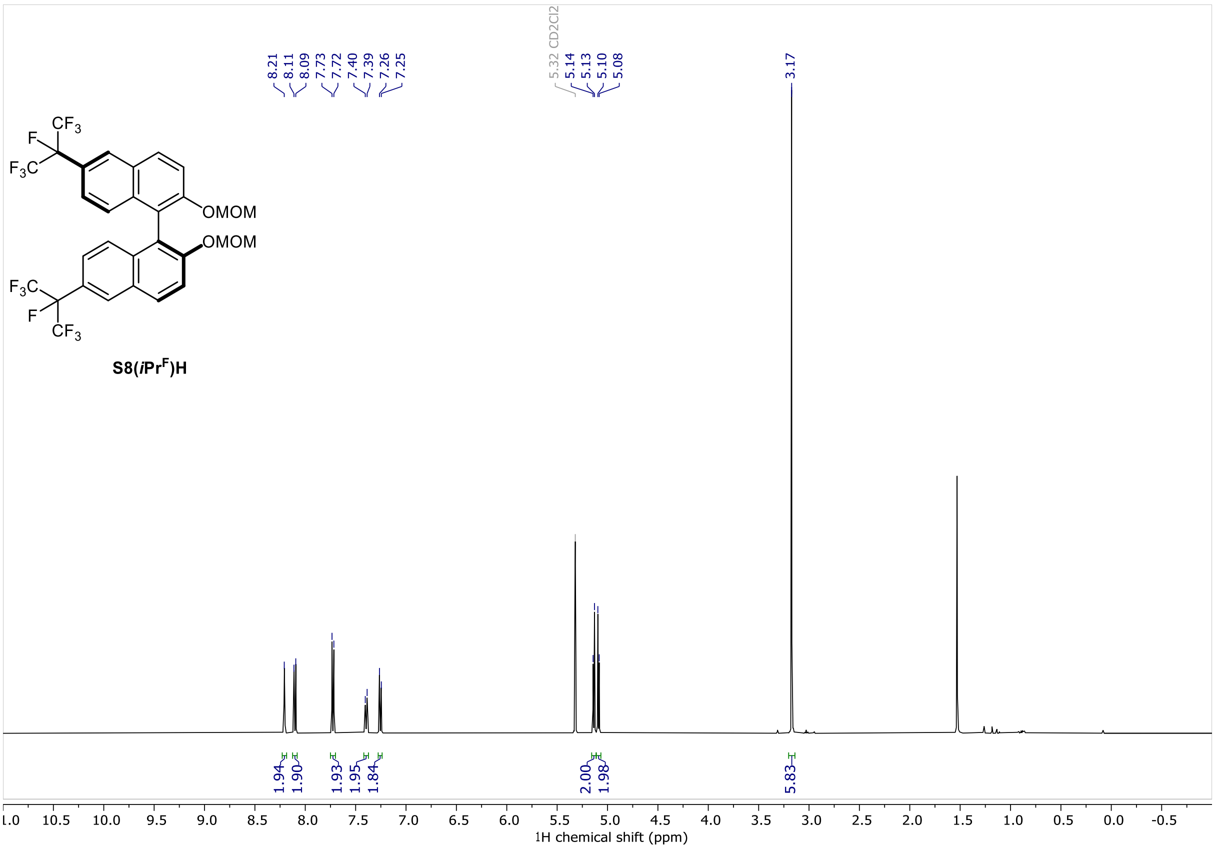

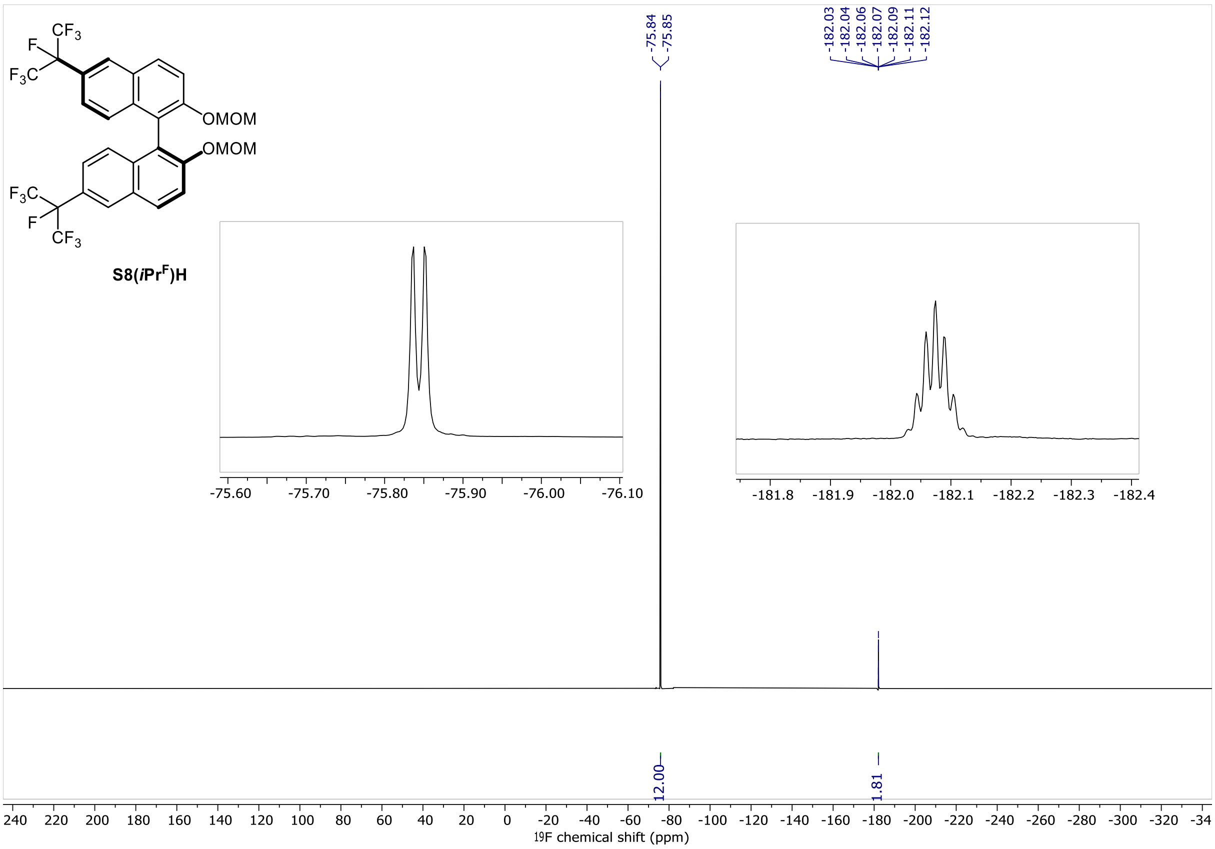

NMR spectra for compound **S8(*i*Pr^F)H**: ^{1}H (501 MHz) and ^{19}F (471 MHz), in CD_2Cl_2.

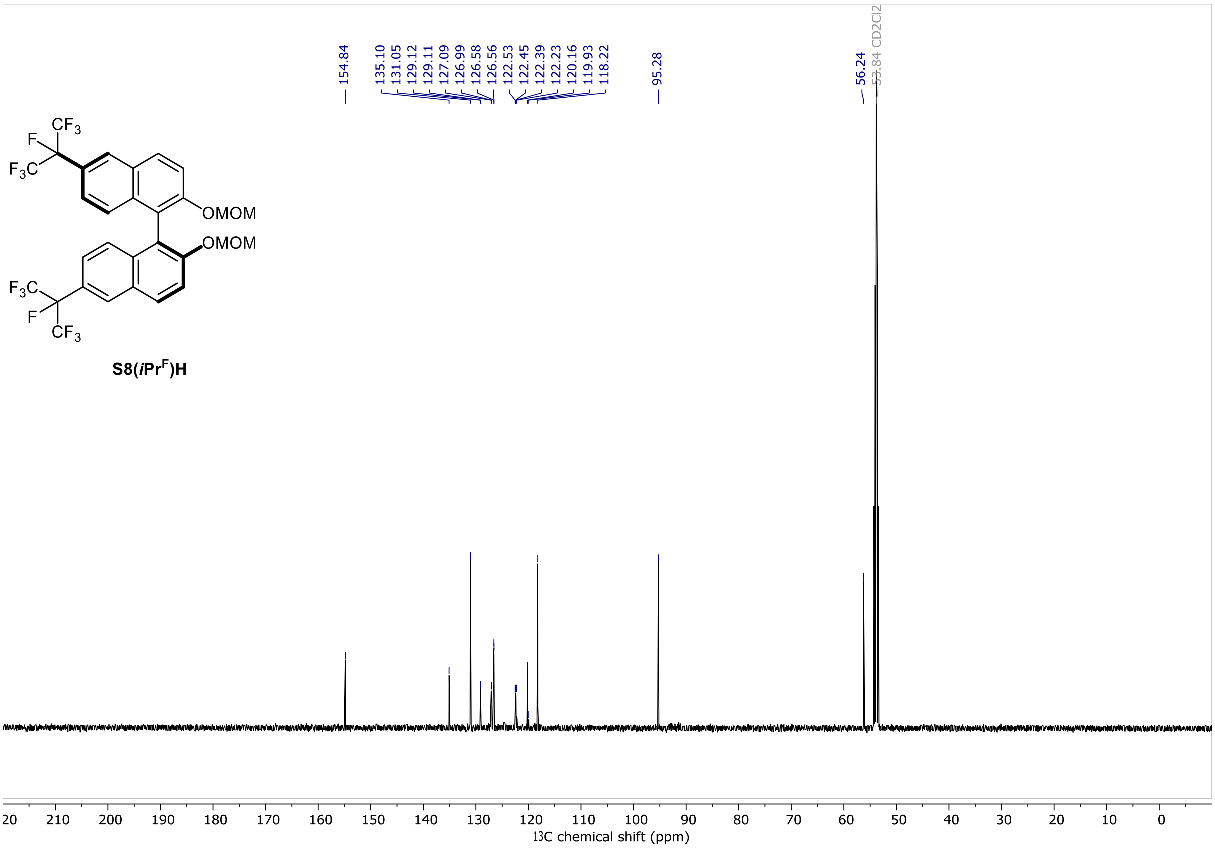
S8(*i*Pr[F])H

NMR spectra for compound **S8(*i*Pr[F])H** *(continuation)*: ^{13}C (126 MHz), in CD_2Cl_2.

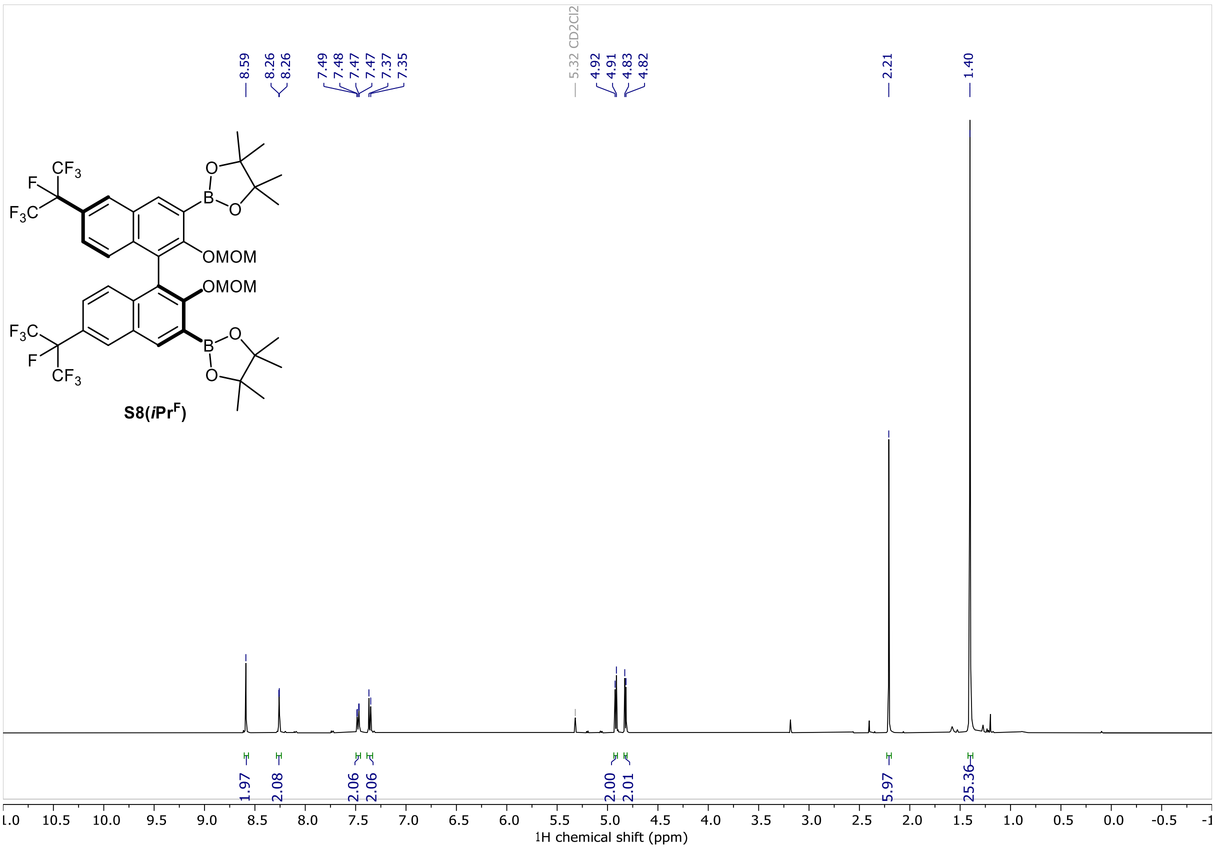

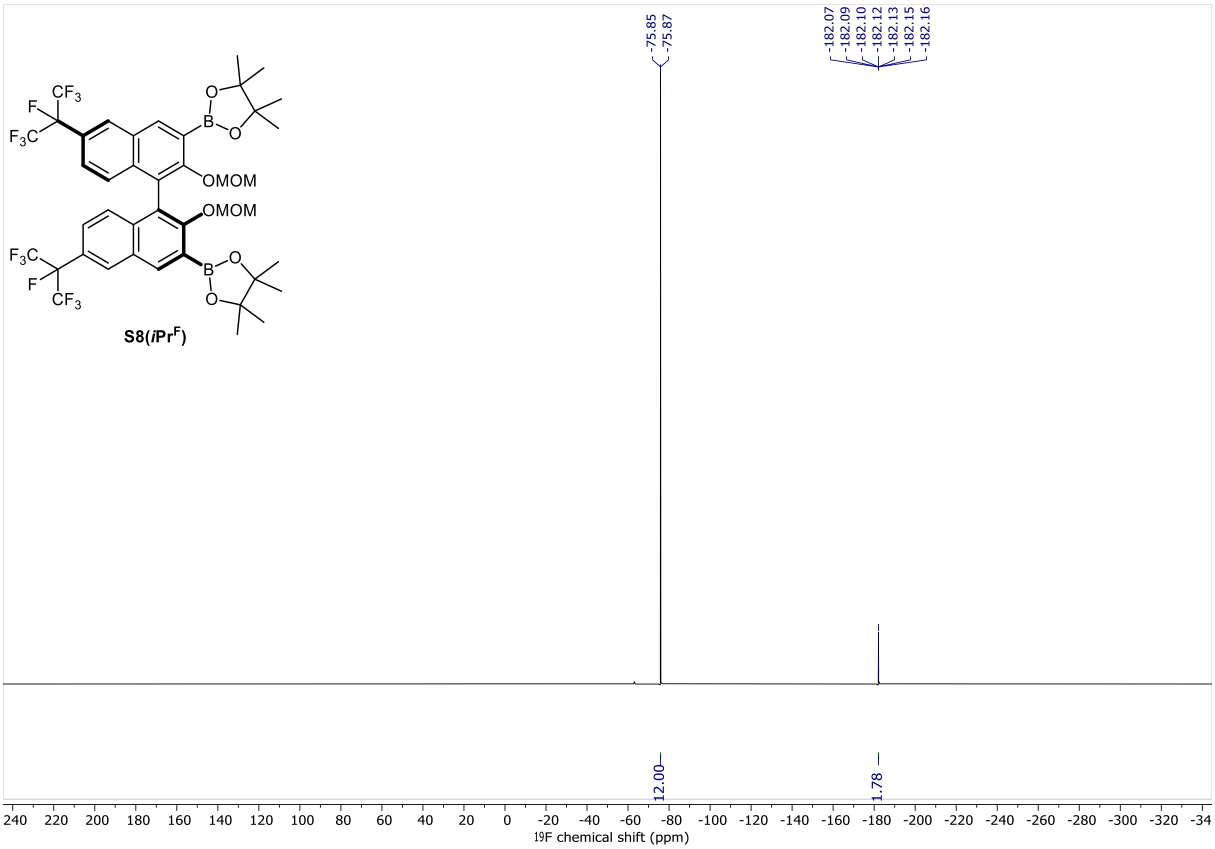

NMR spectra for compound **S8(*i*Pr^F)**: ^{1}H (501 MHz) and ^{19}F (471 MHz), in CD_2Cl_2.

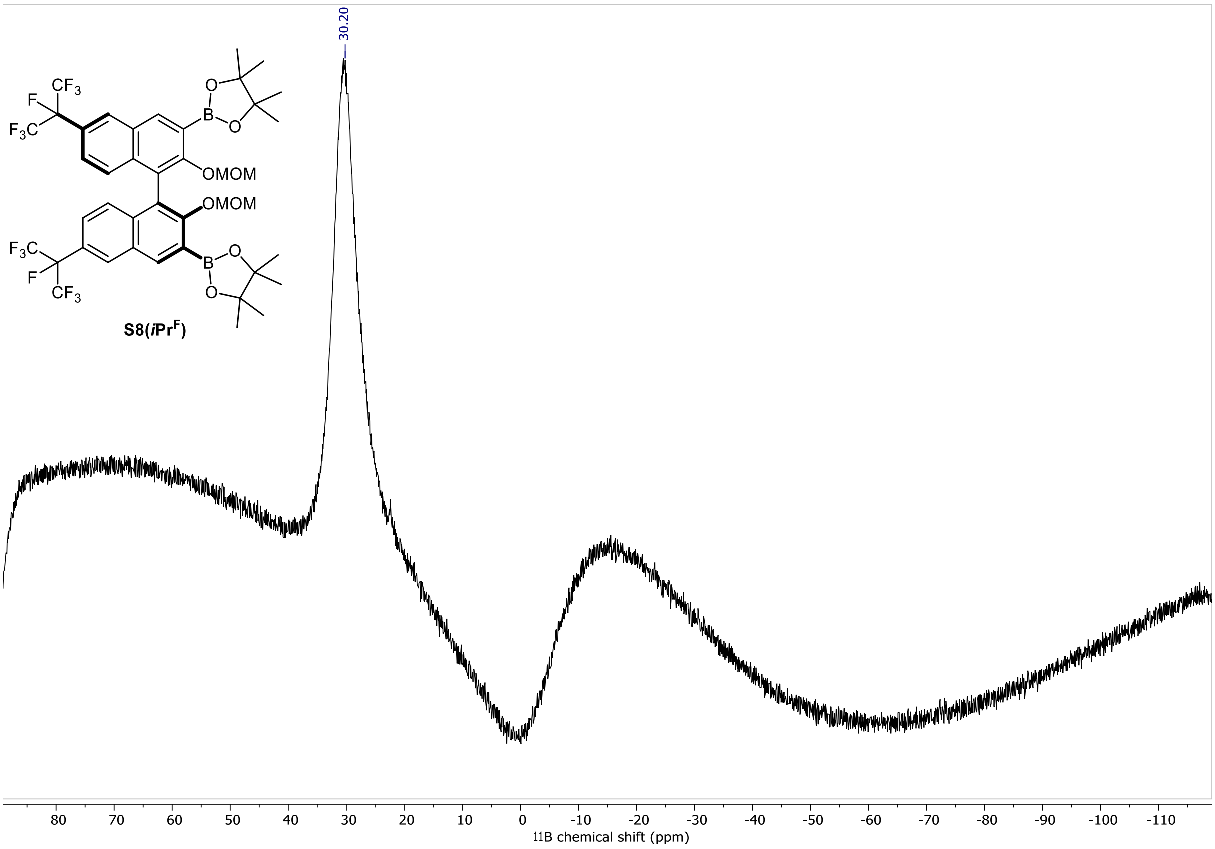

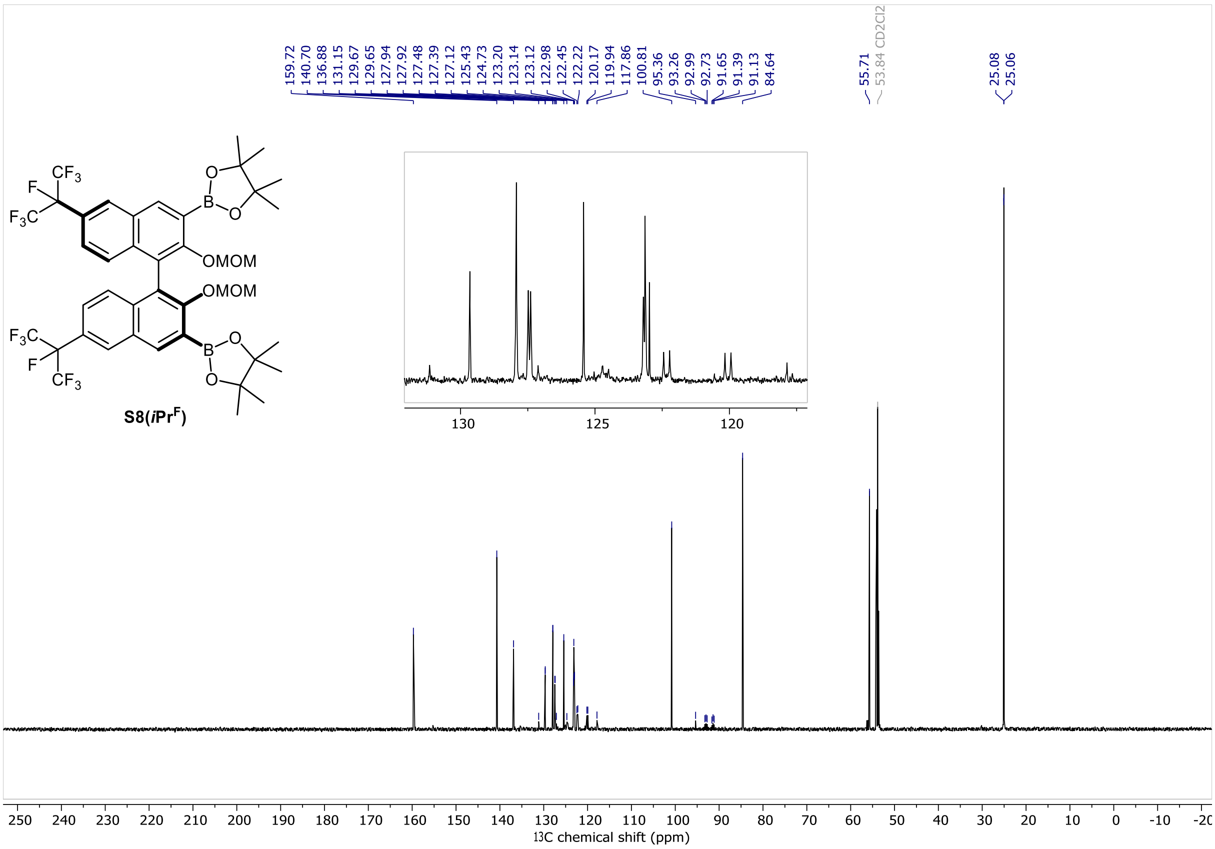

NMR spectra for compound **S8(*i*Pr^F)** *(continuation)*: ^{11}B (161 MHz) and ^{13}C (126 MHz), in CD_2Cl_2.

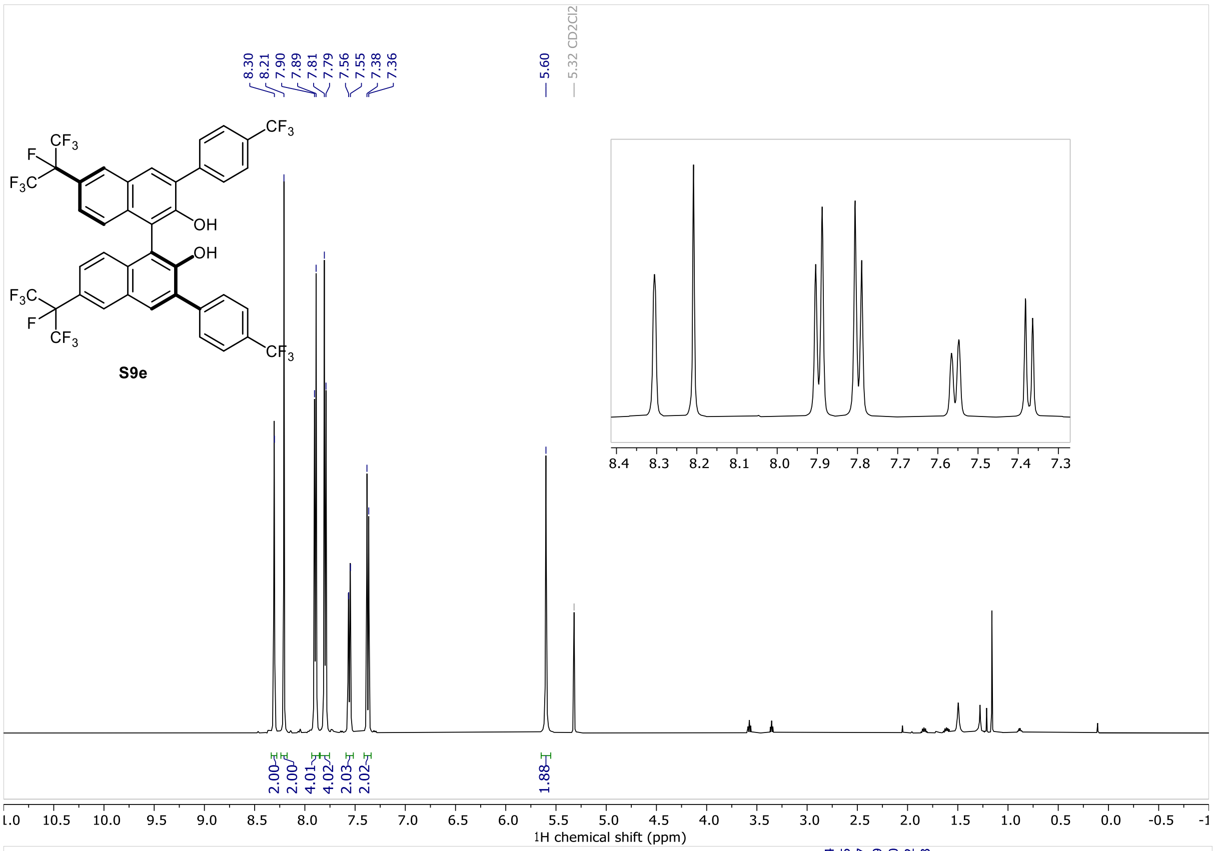

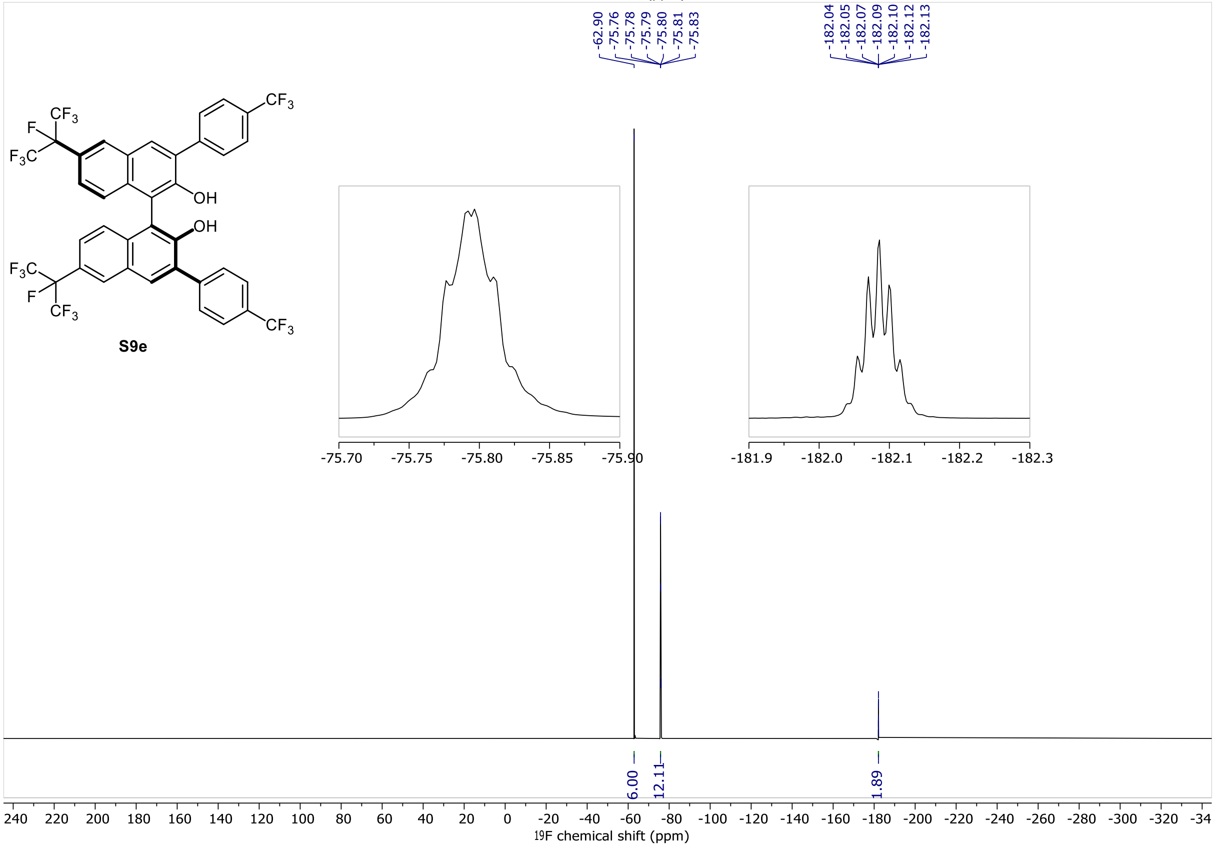

NMR spectra for compound **S9e**: ^{1}H (501 MHz) and ^{19}F (471 MHz), in CD_2Cl_2.

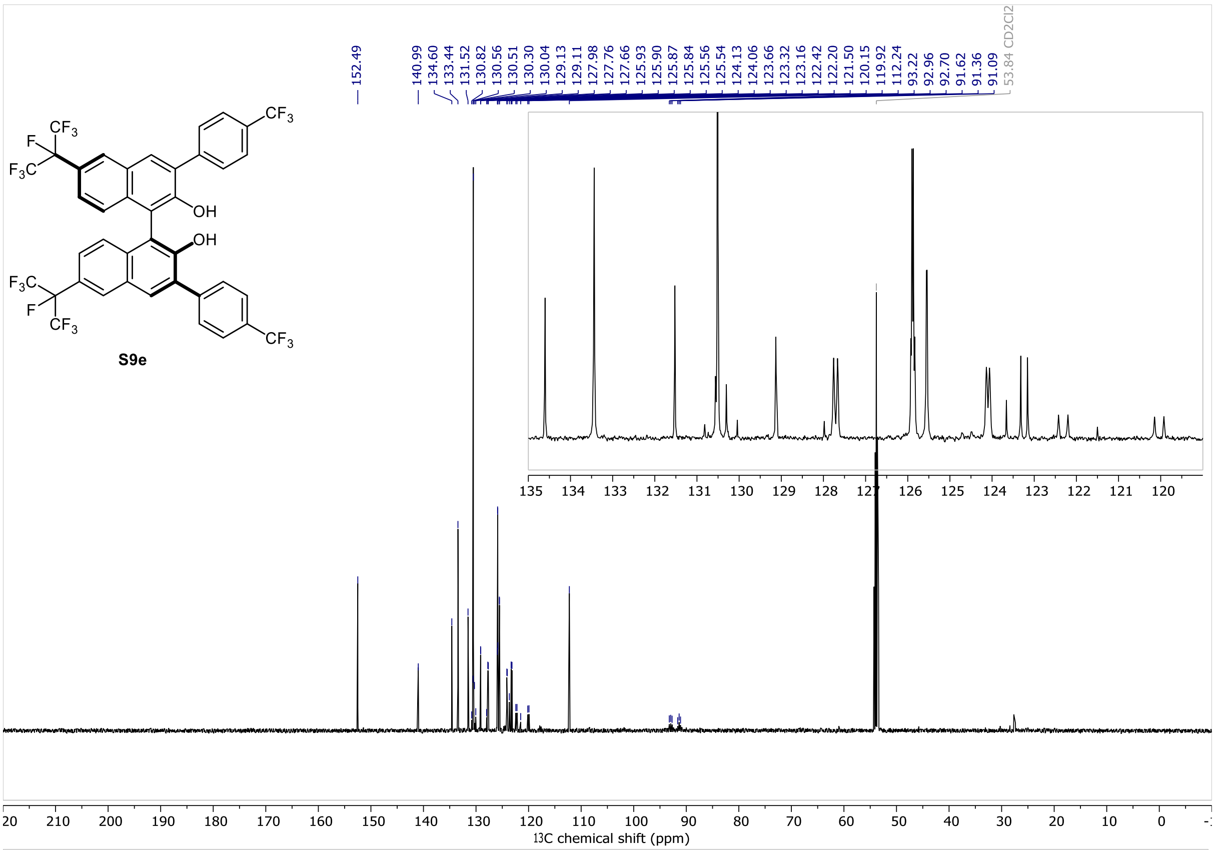

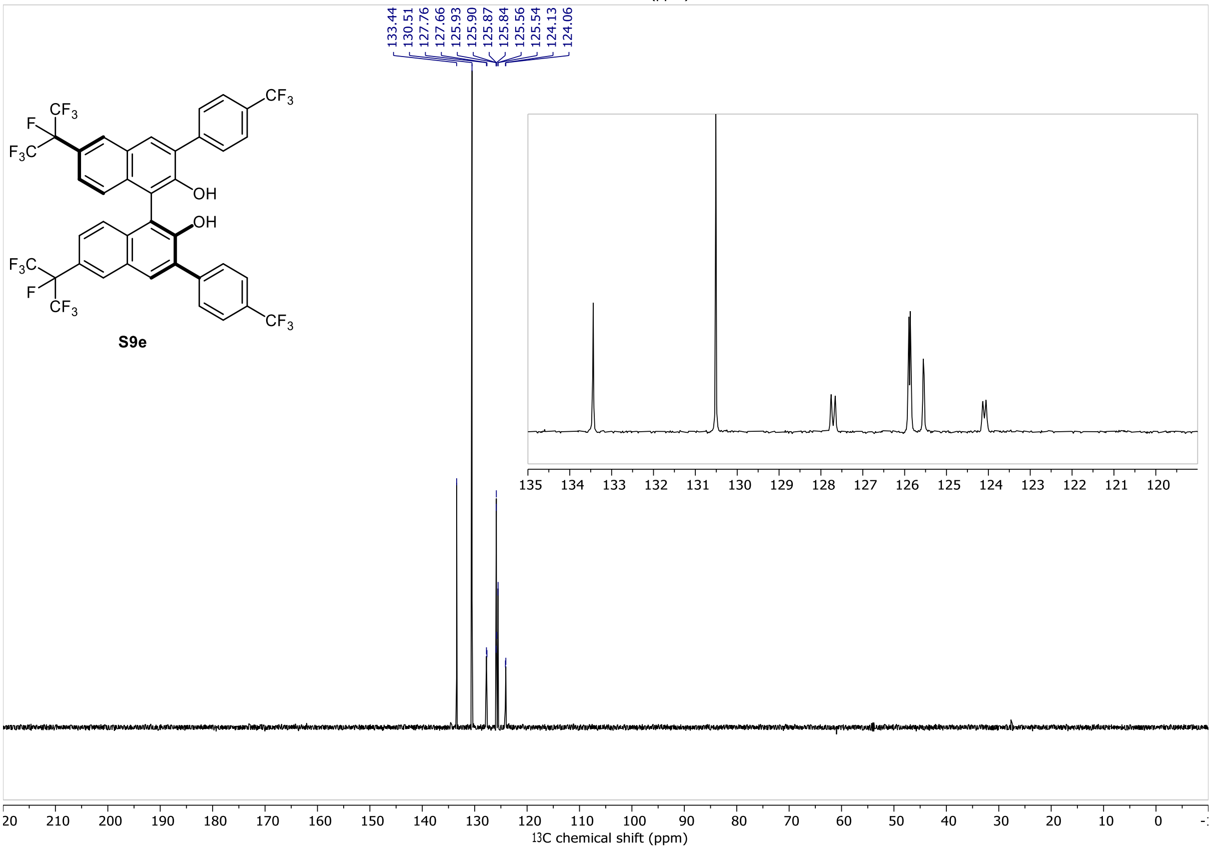

NMR spectra for compound **S9e** *(continuation)*: ^{13}C (126 MHz) and DEPT-135 (126 MHz), in CD_2Cl_2.

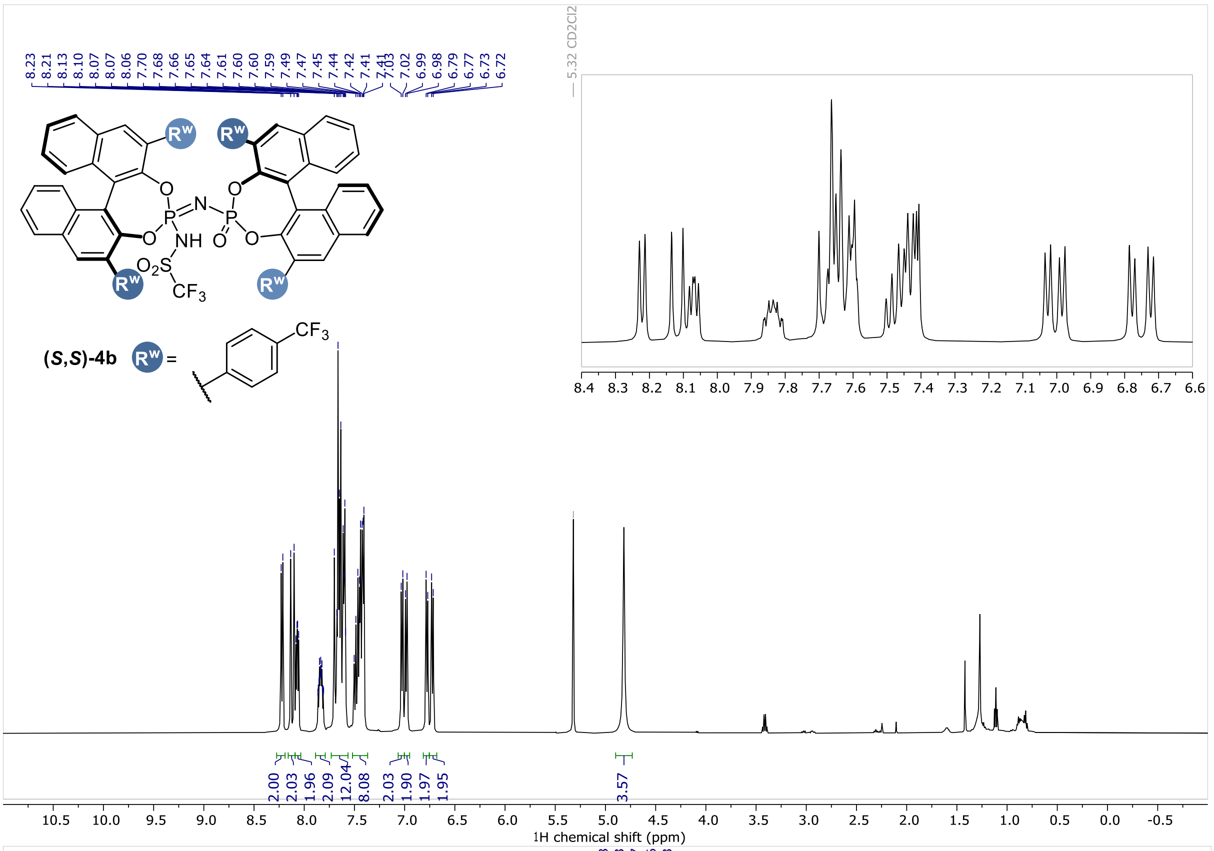

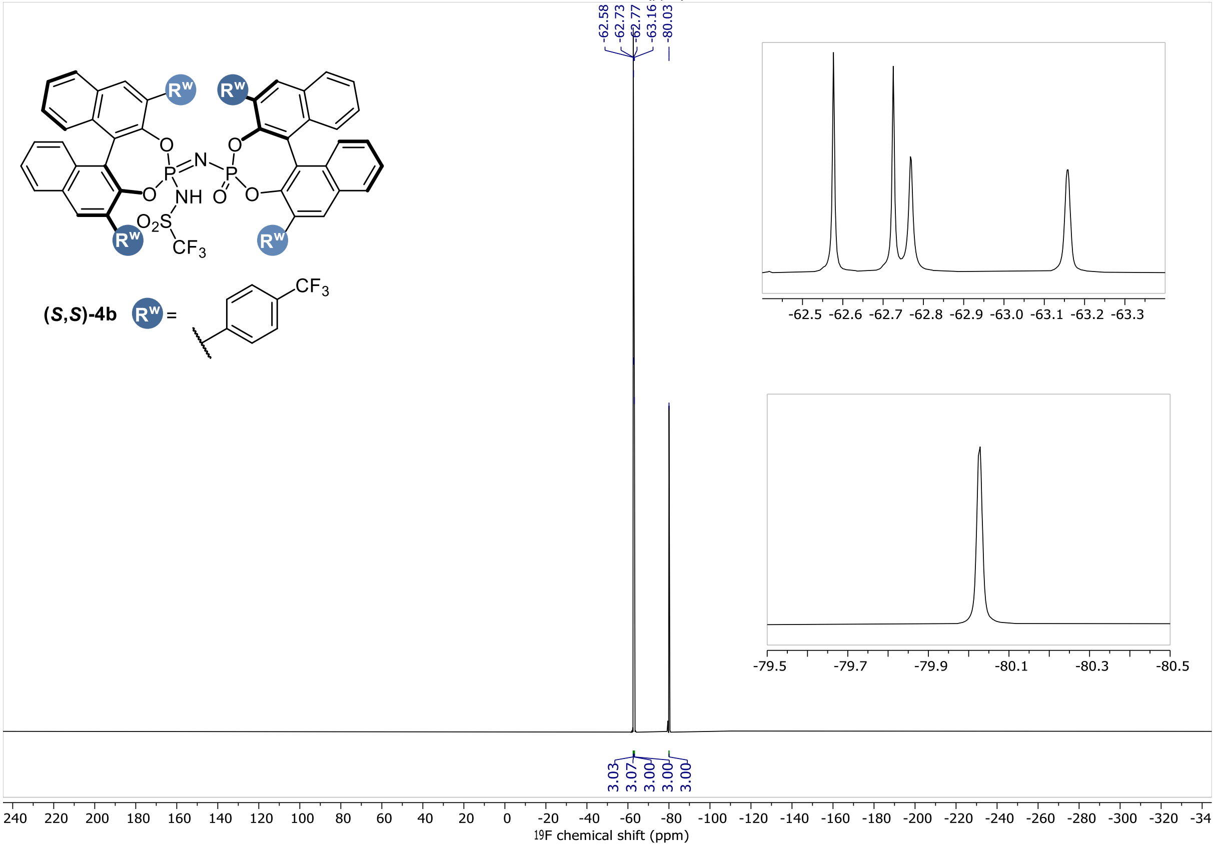

NMR spectra for compound **4b**: ^{1}H (501 MHz) and ^{19}F (471 MHz), in CD_2Cl_2.

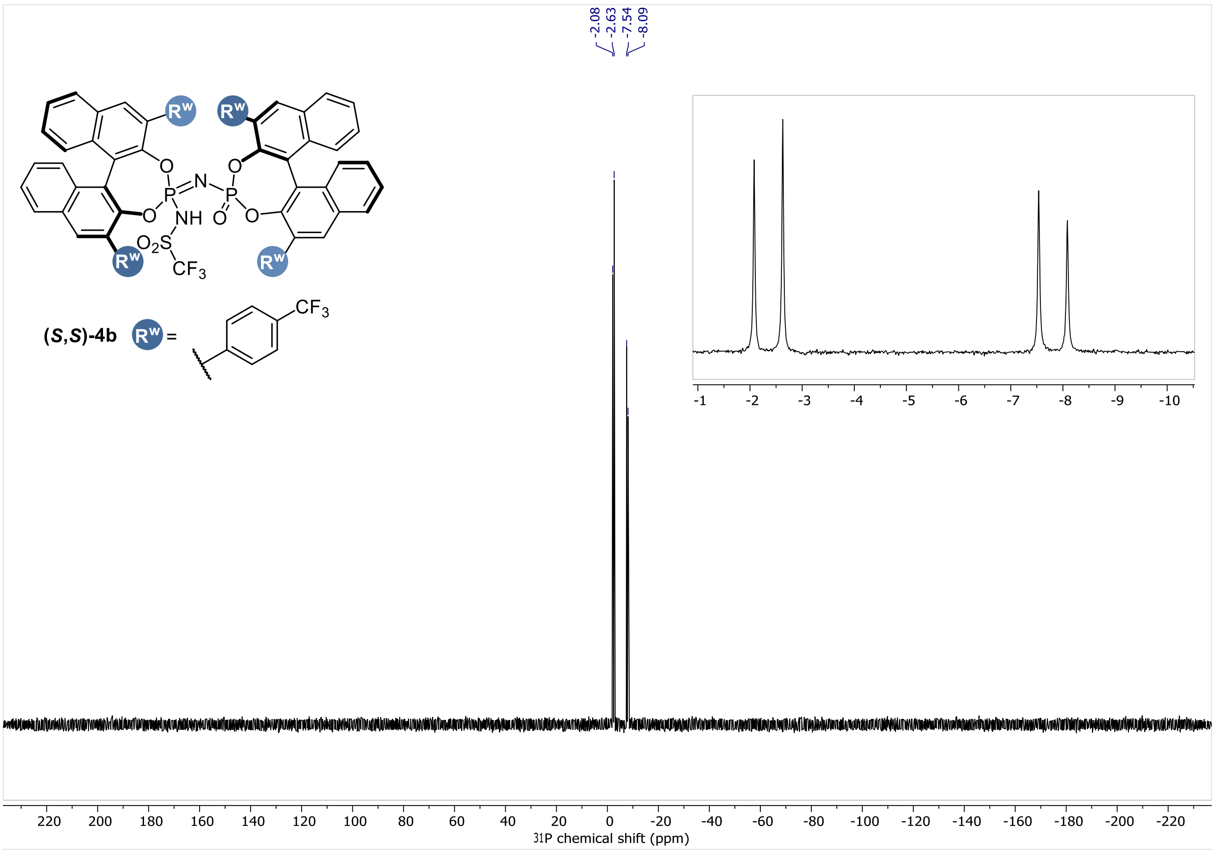

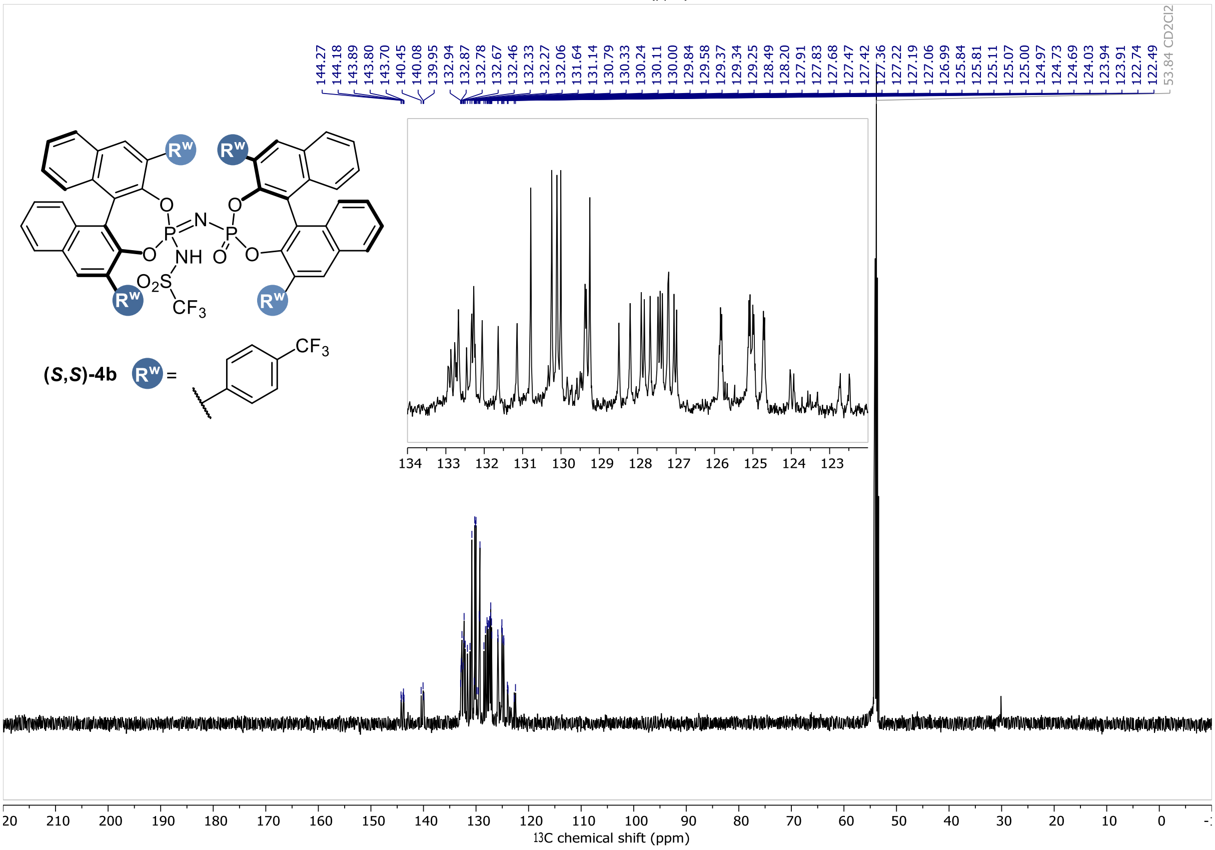

NMR spectra for compound **4b** *(continuation)*: ^{31}P (203 MHz) and ^{13}C (126 MHz), in CD_2Cl_2.

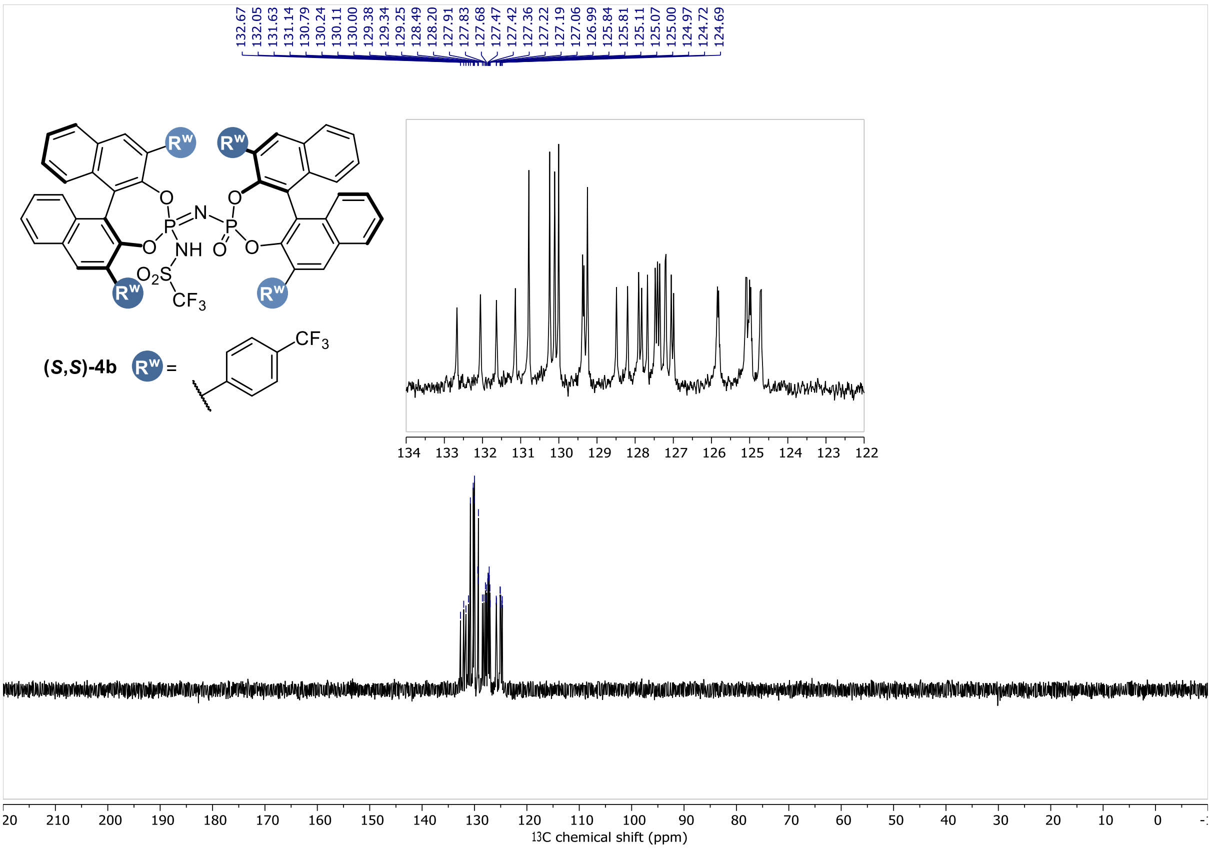

NMR spectra for compound **4b** *(continuation)*: DEPT-135 (126 MHz), in CD_2Cl_2.

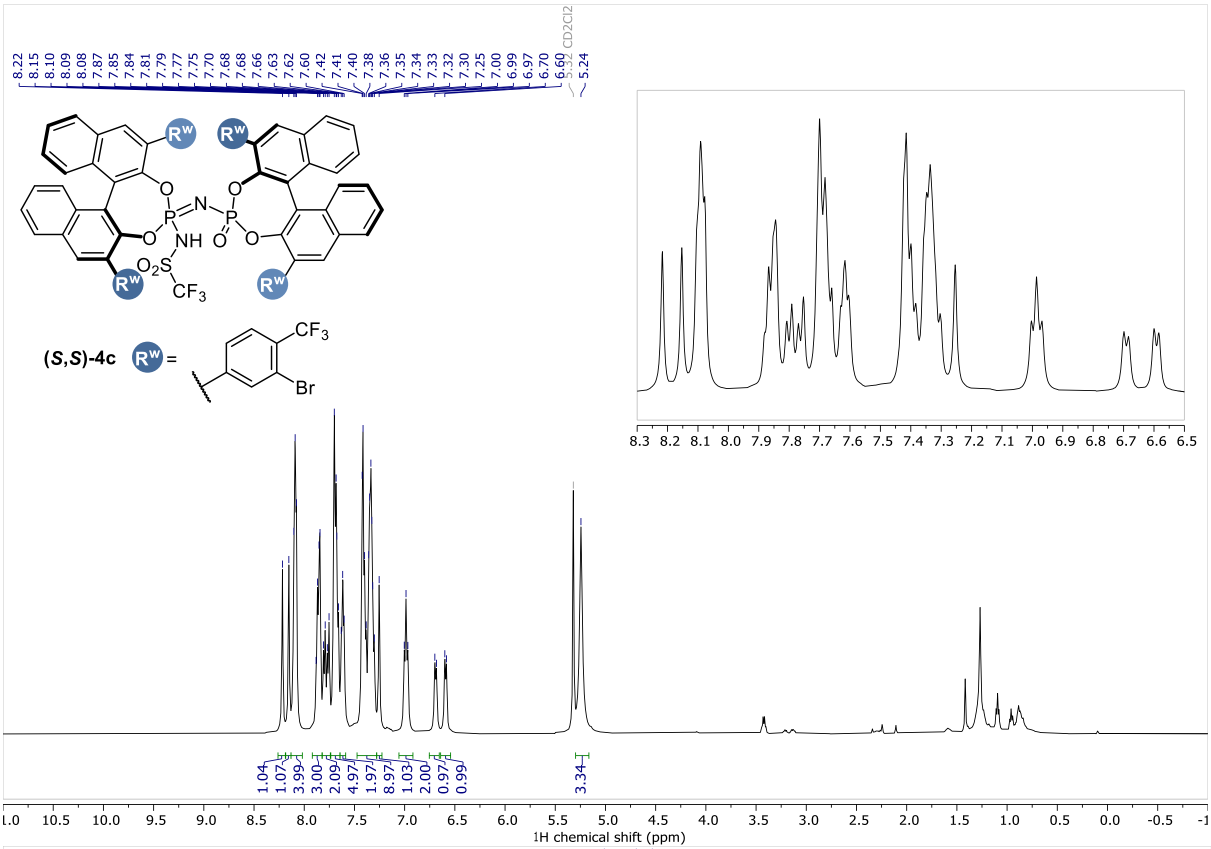

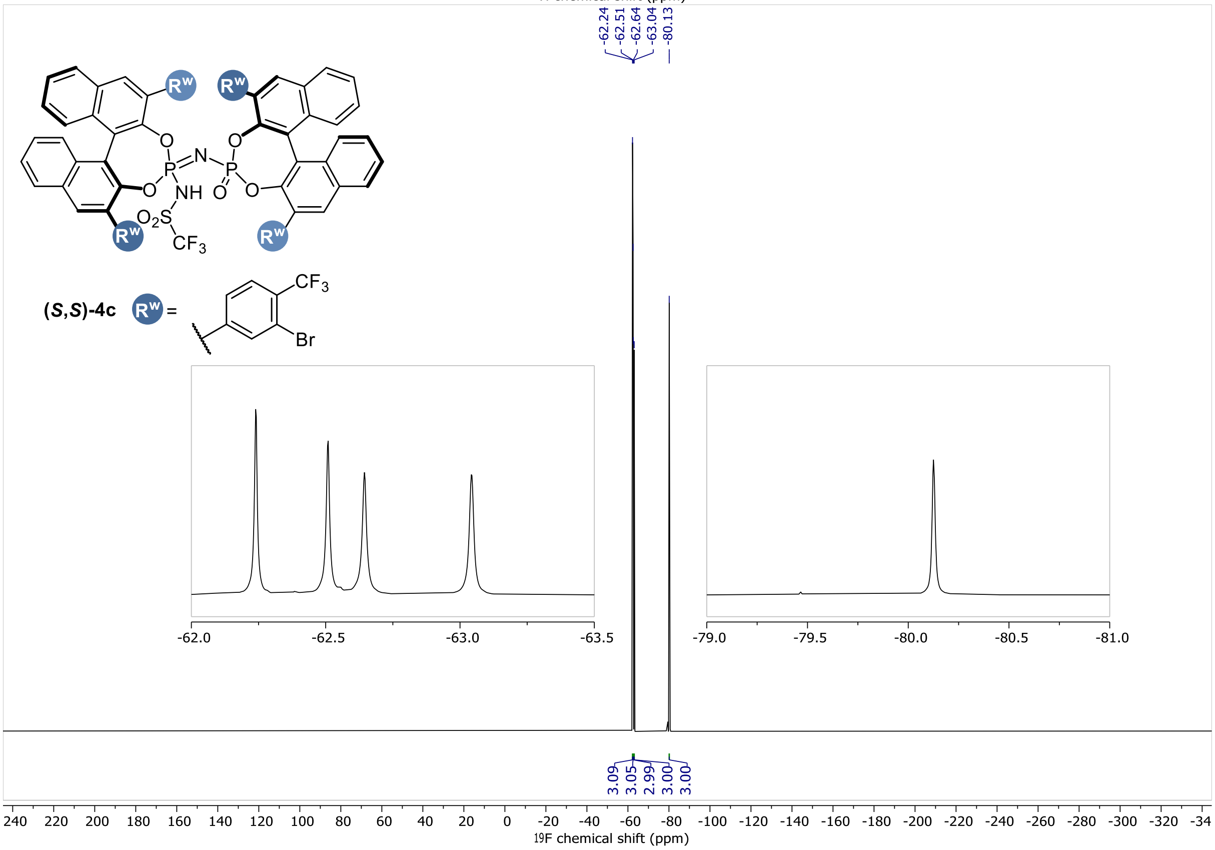

NMR spectra for compound **4c**: ^{1}H (501 MHz) and ^{19}F (471 MHz), in CD_2Cl_2.

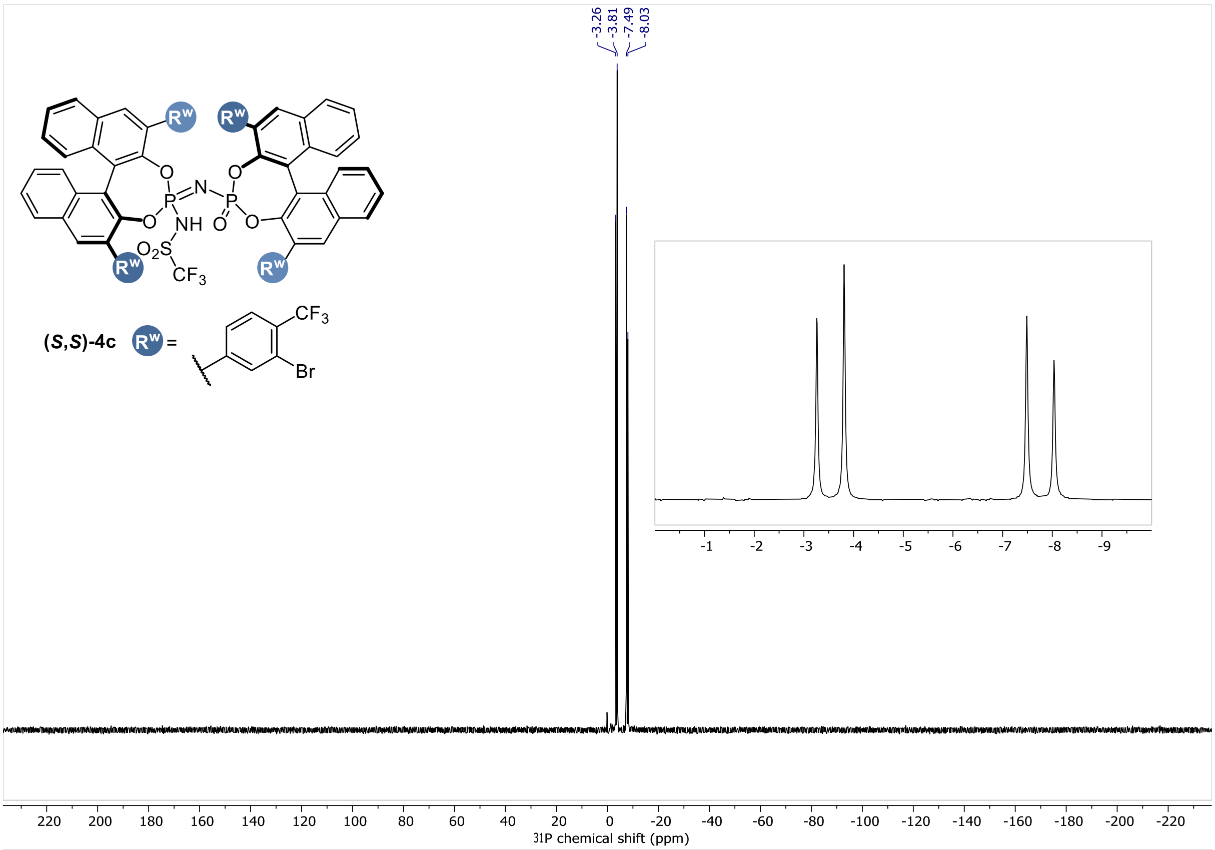

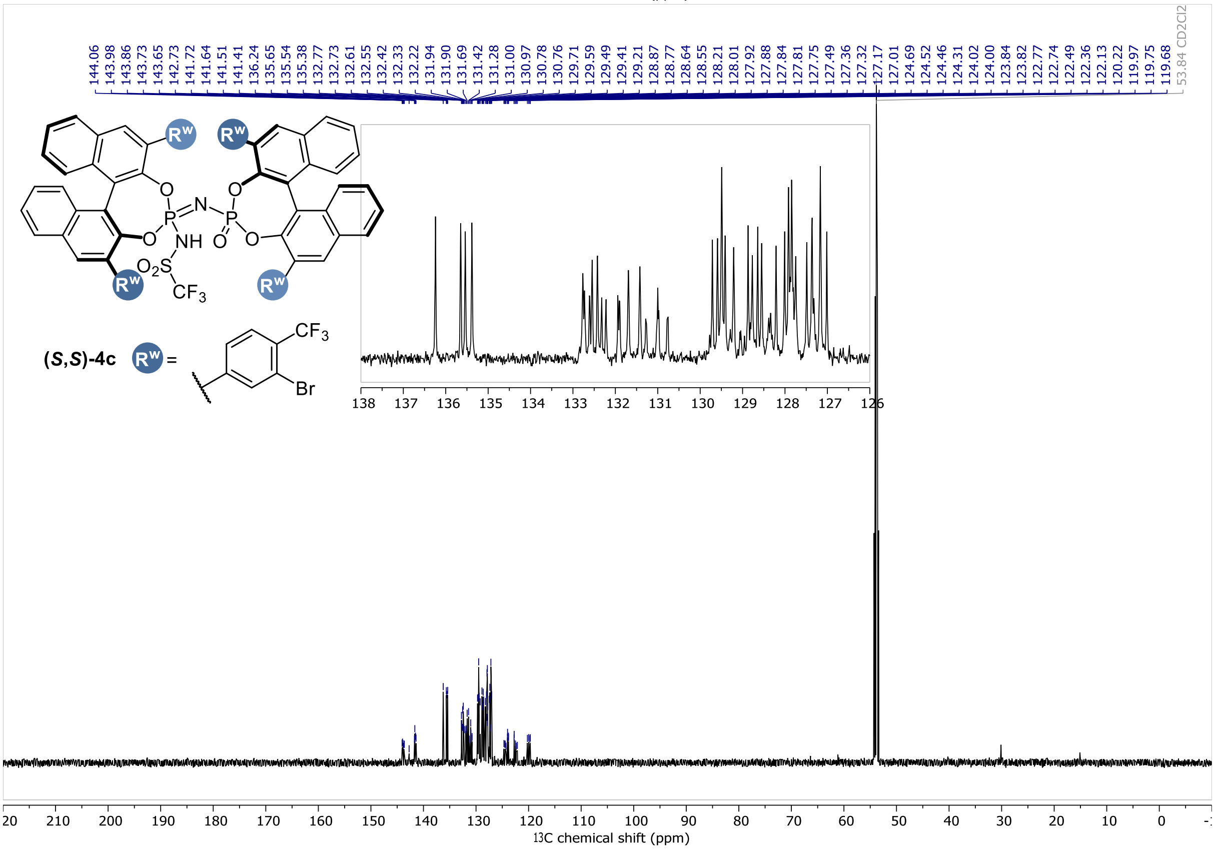

NMR spectra for compound **4c** *(continuation)*: ^{31}P (203 MHz) and ^{13}C (126 MHz), in CD_2Cl_2.

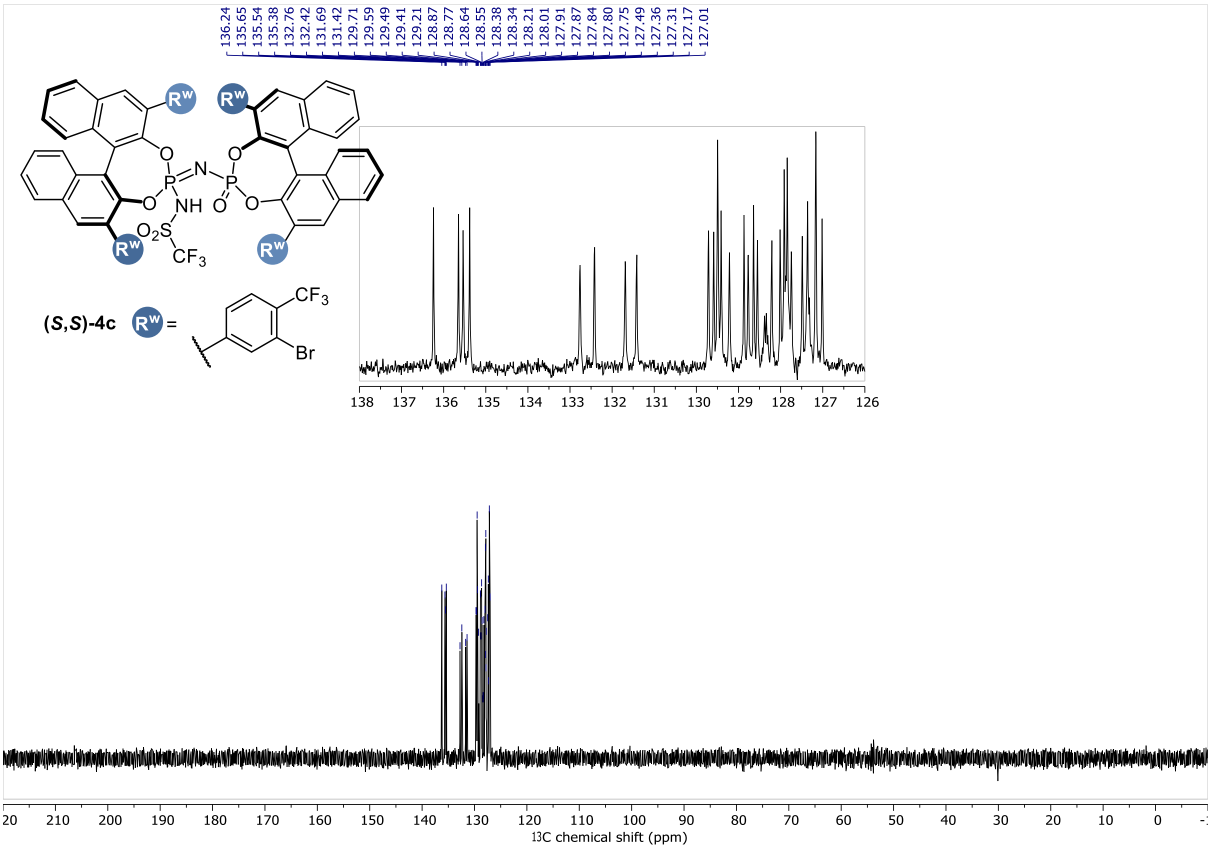

NMR spectra for compound **4c** *(continuation)*: DEPT-135 (126 MHz), in CD_2Cl_2.

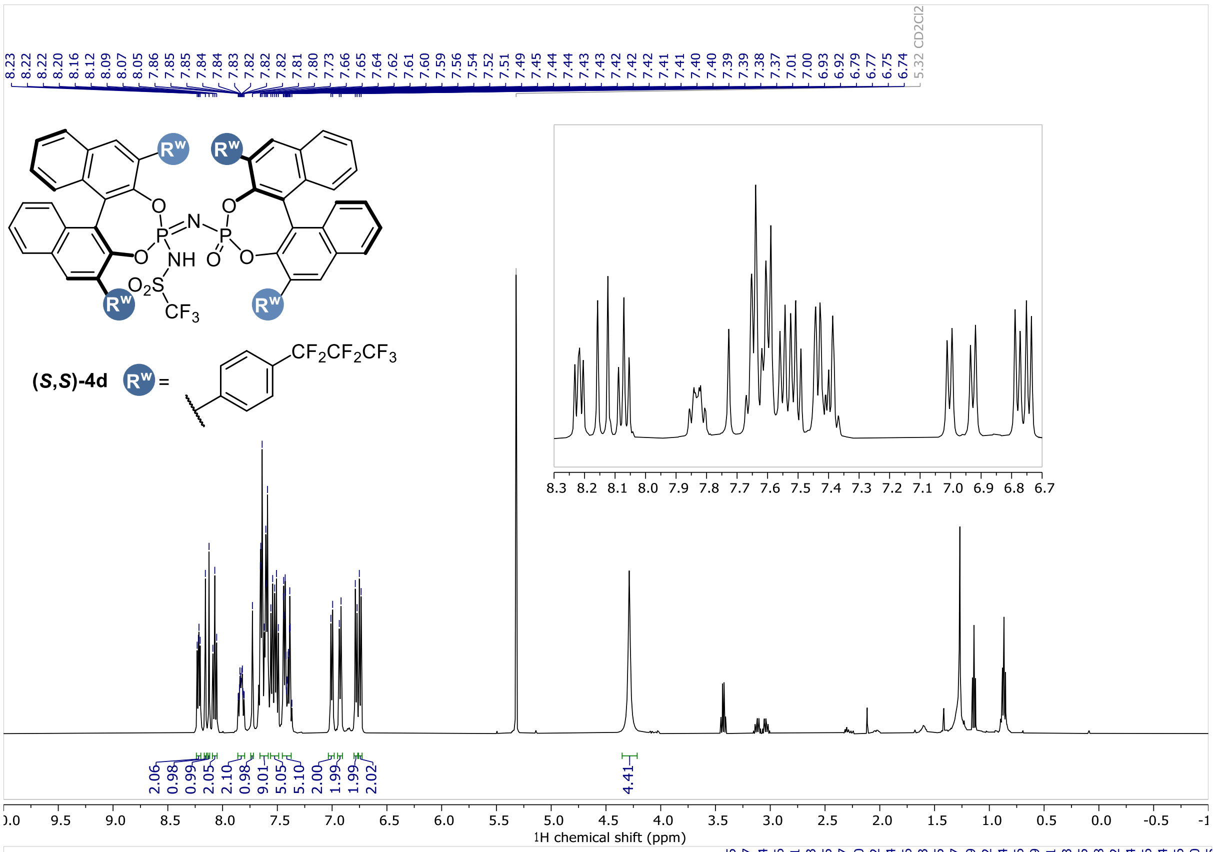

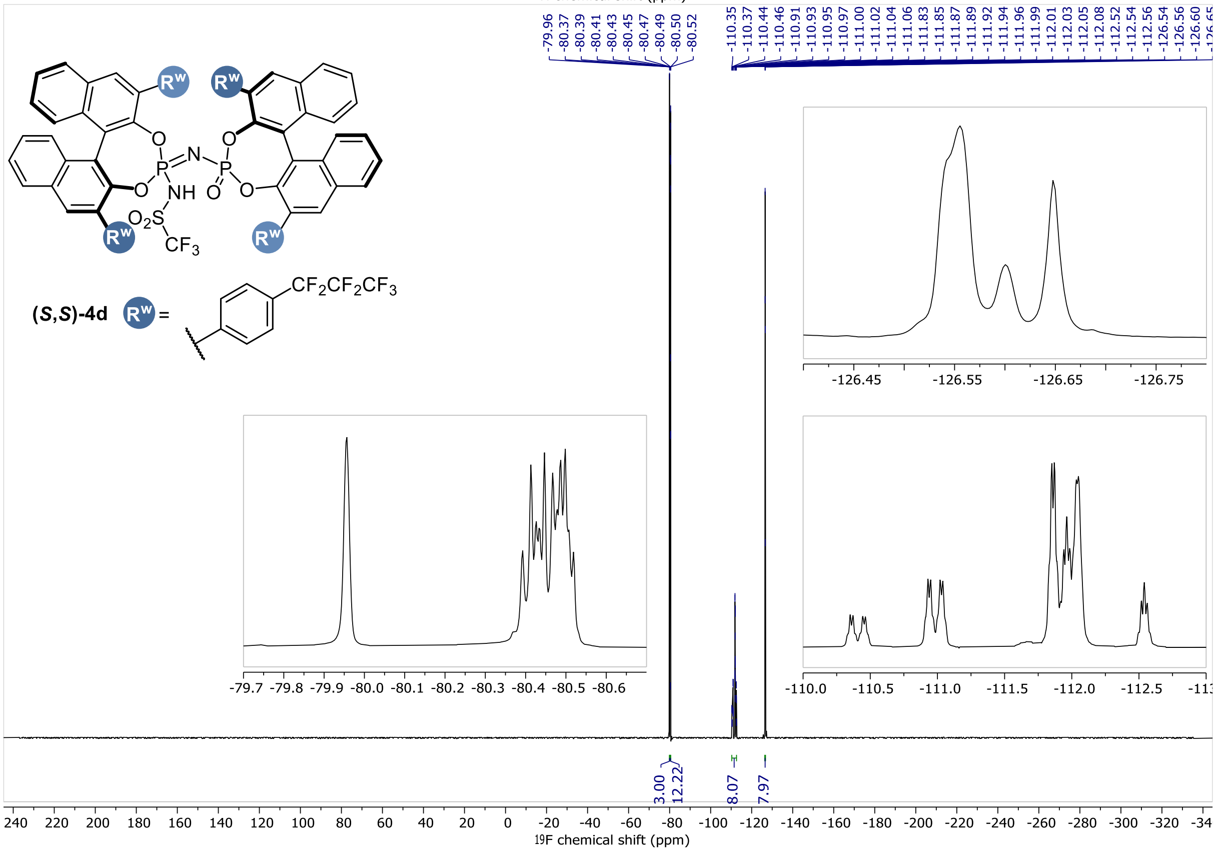

NMR spectra for compound **4d**: ^1H (501 MHz) and ^{19}F (471 MHz), in CD_2Cl_2.

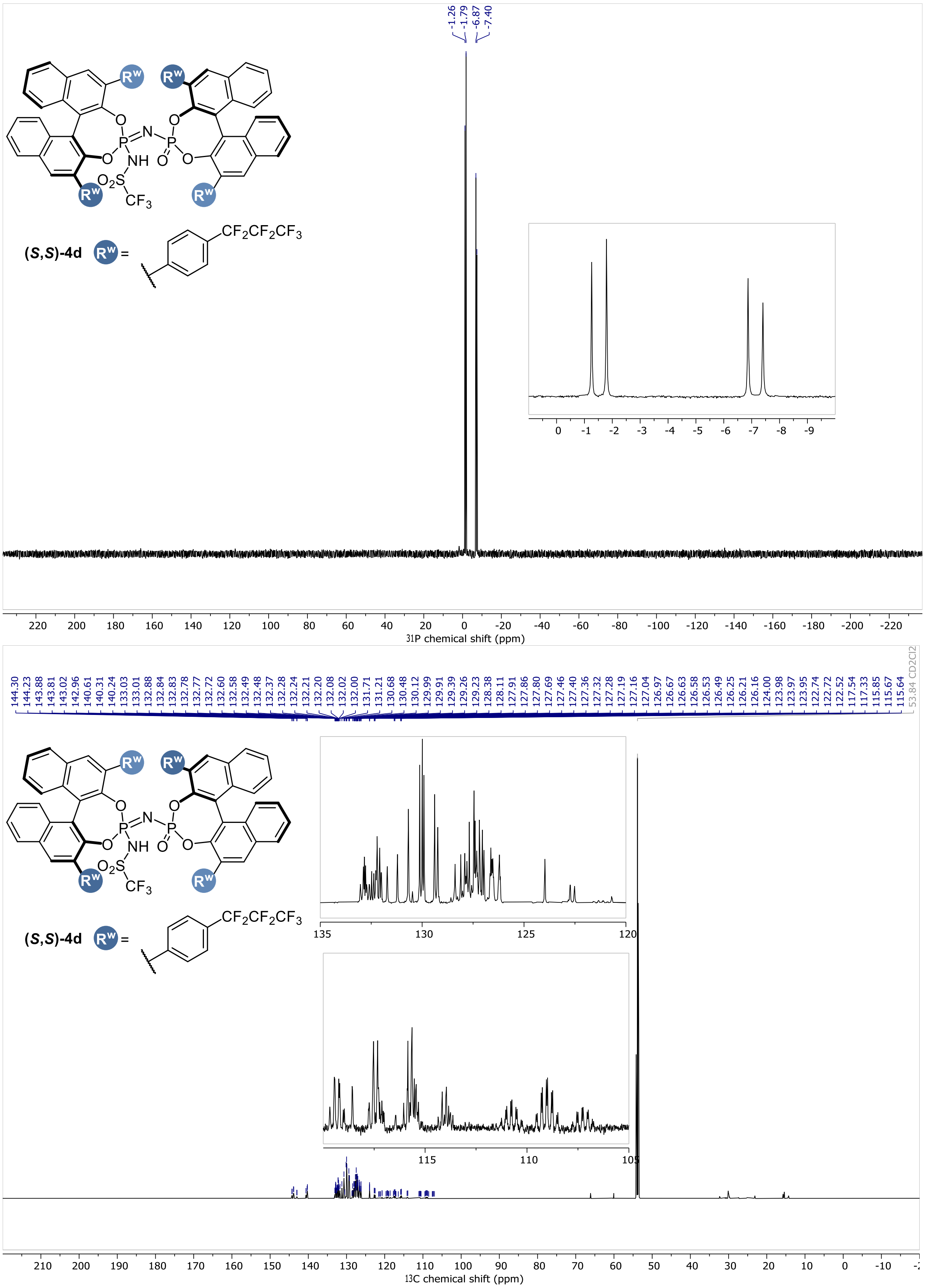

NMR spectra for compound **4d** *(continuation)*: ^{31}P (203 MHz), and ^{13}C (151 MHz), in CD_2Cl_2.

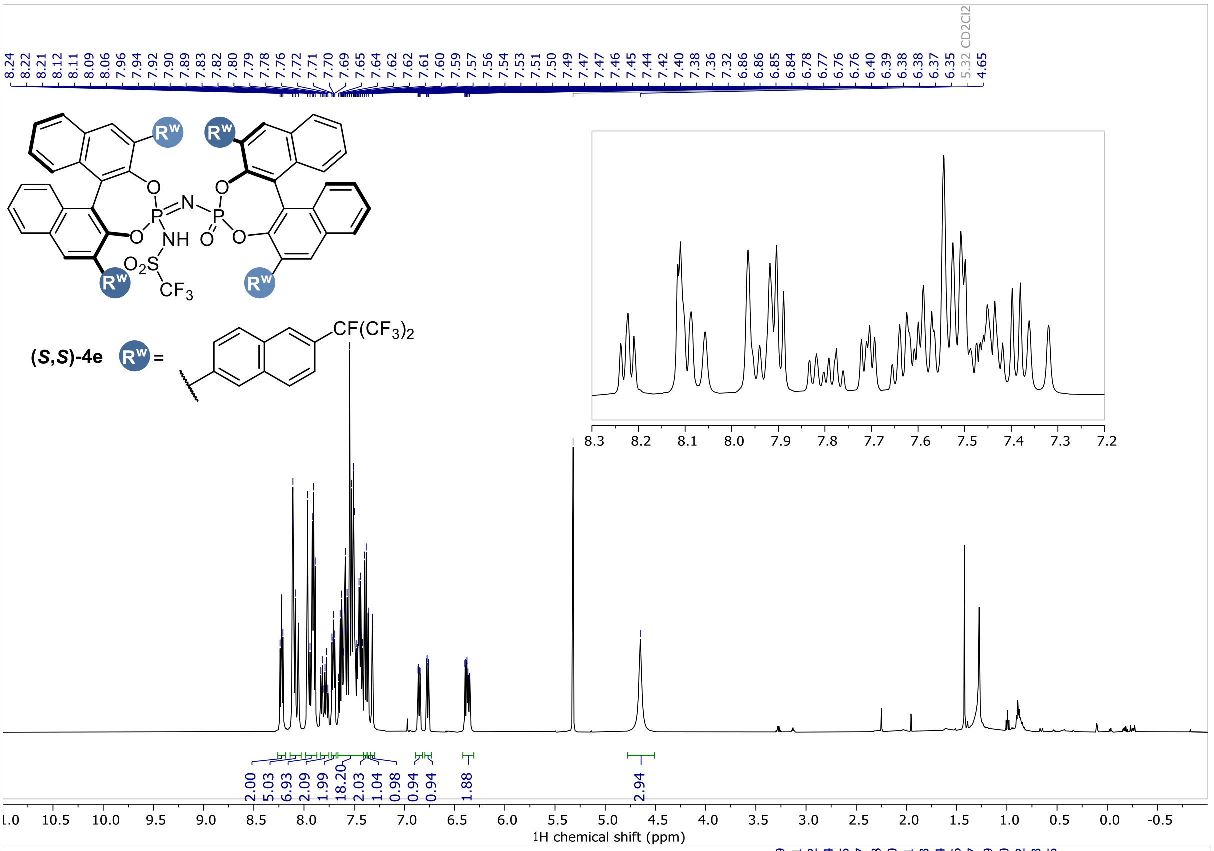

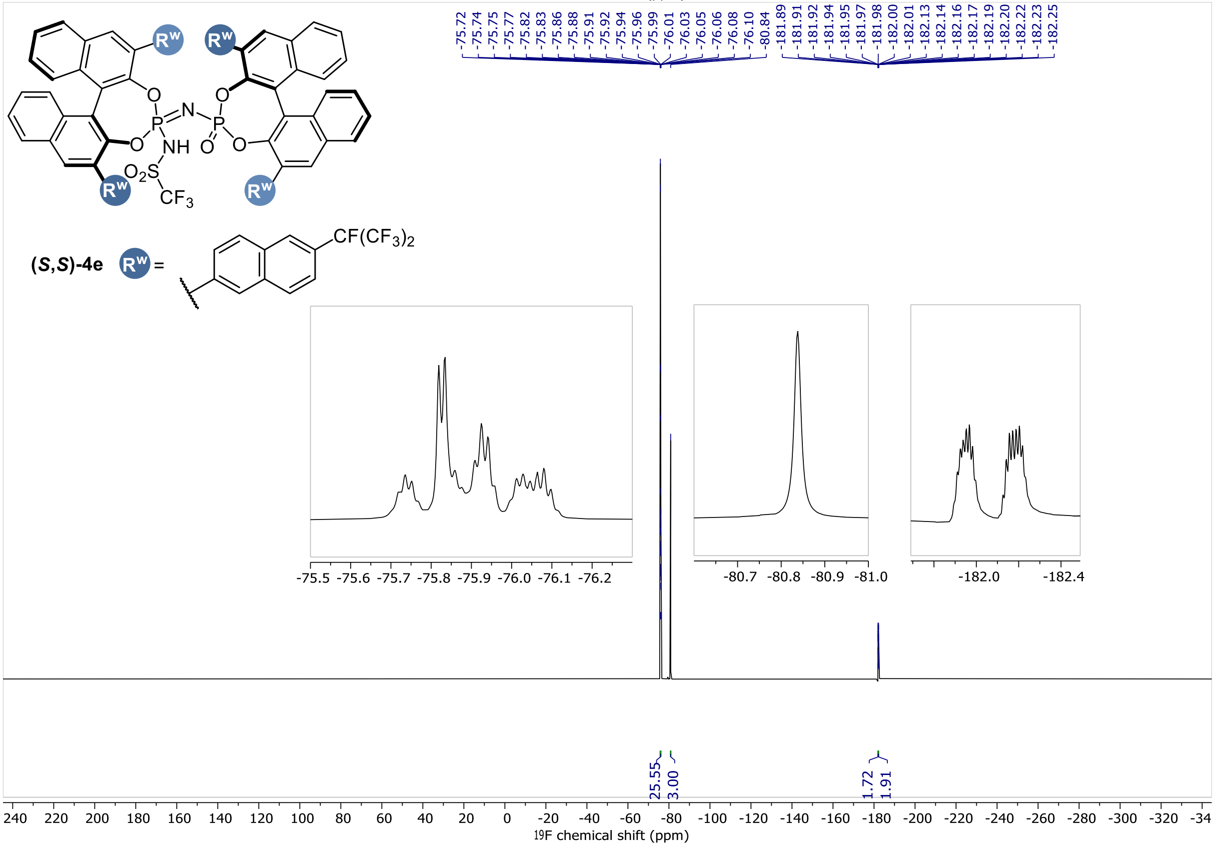

NMR spectra for compound **4e**: ^{1}H (501 MHz) and ^{19}F (471 MHz), in CD_2Cl_2.

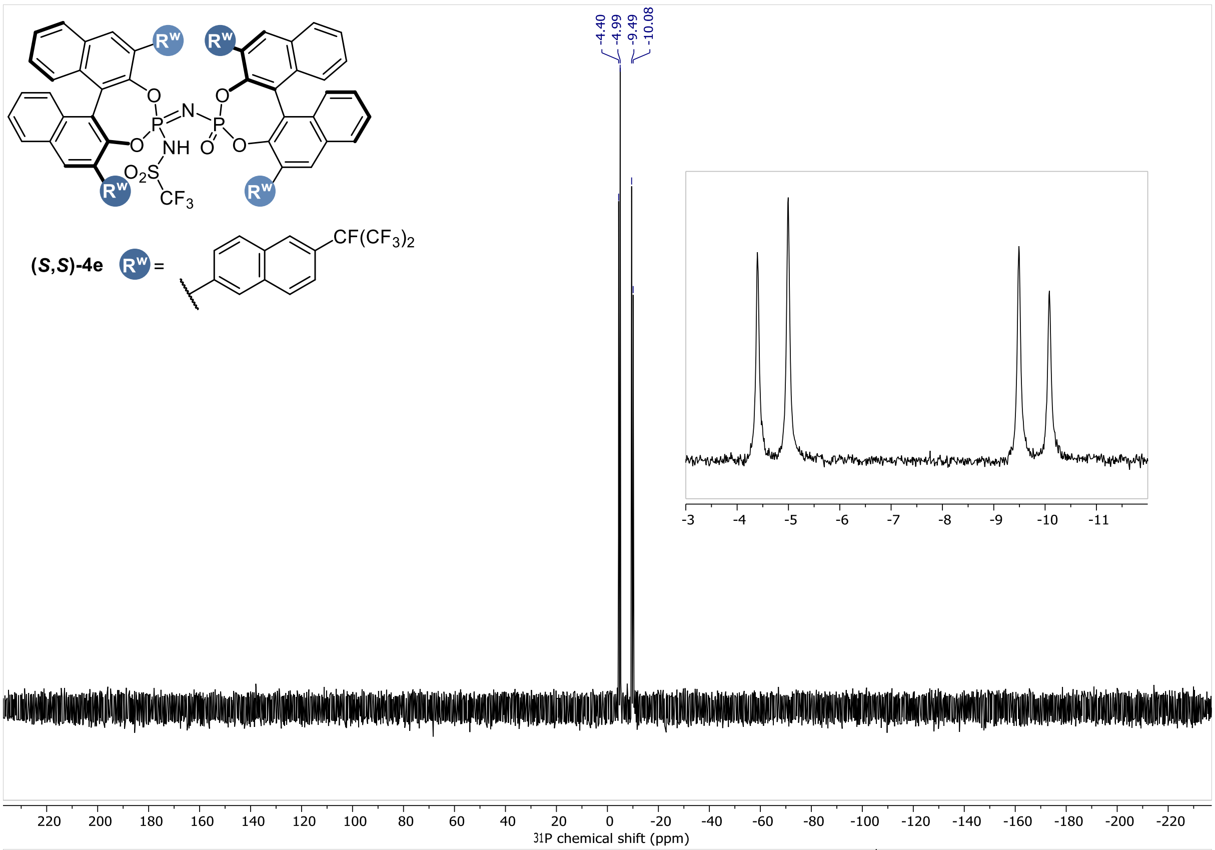

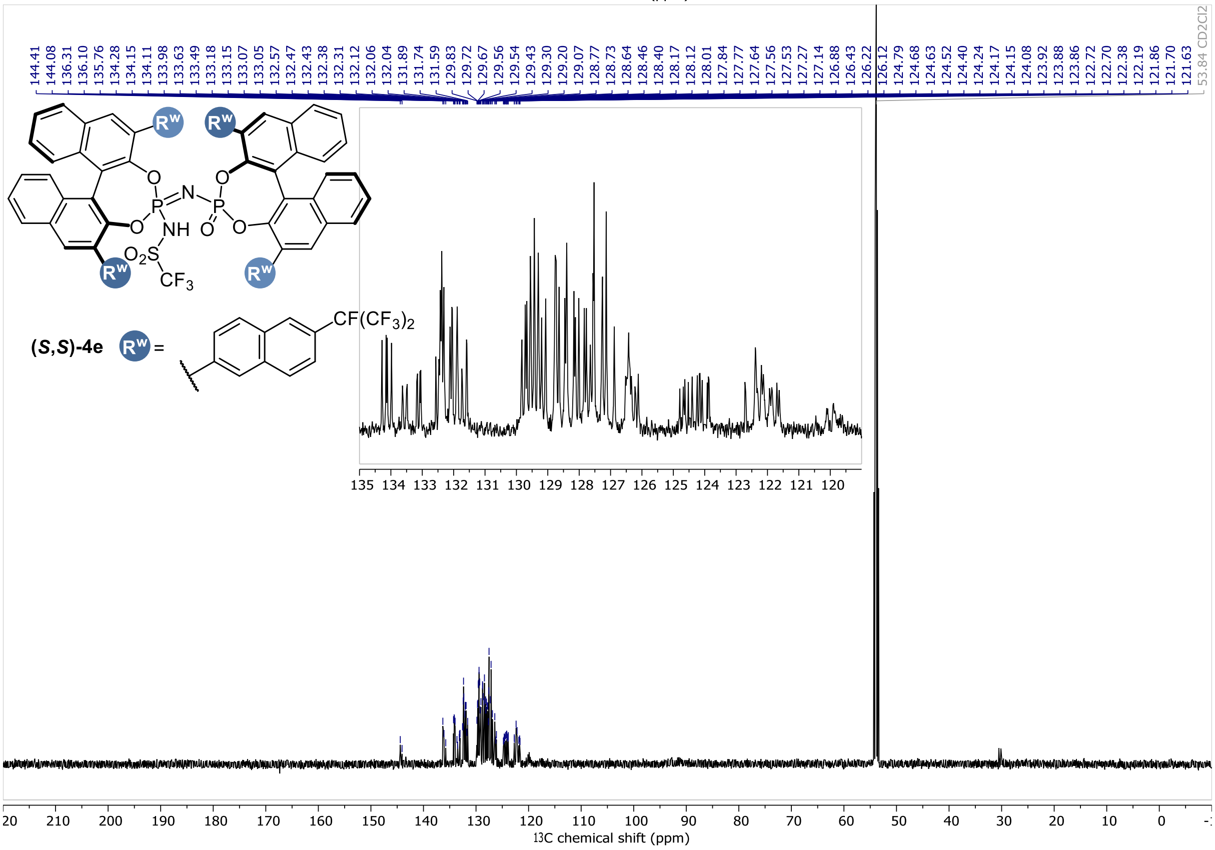

NMR spectra for compound **4e** *(continuation)*: ^{31}P (203 MHz) and ^{13}C (126 MHz), in CD_2Cl_2.

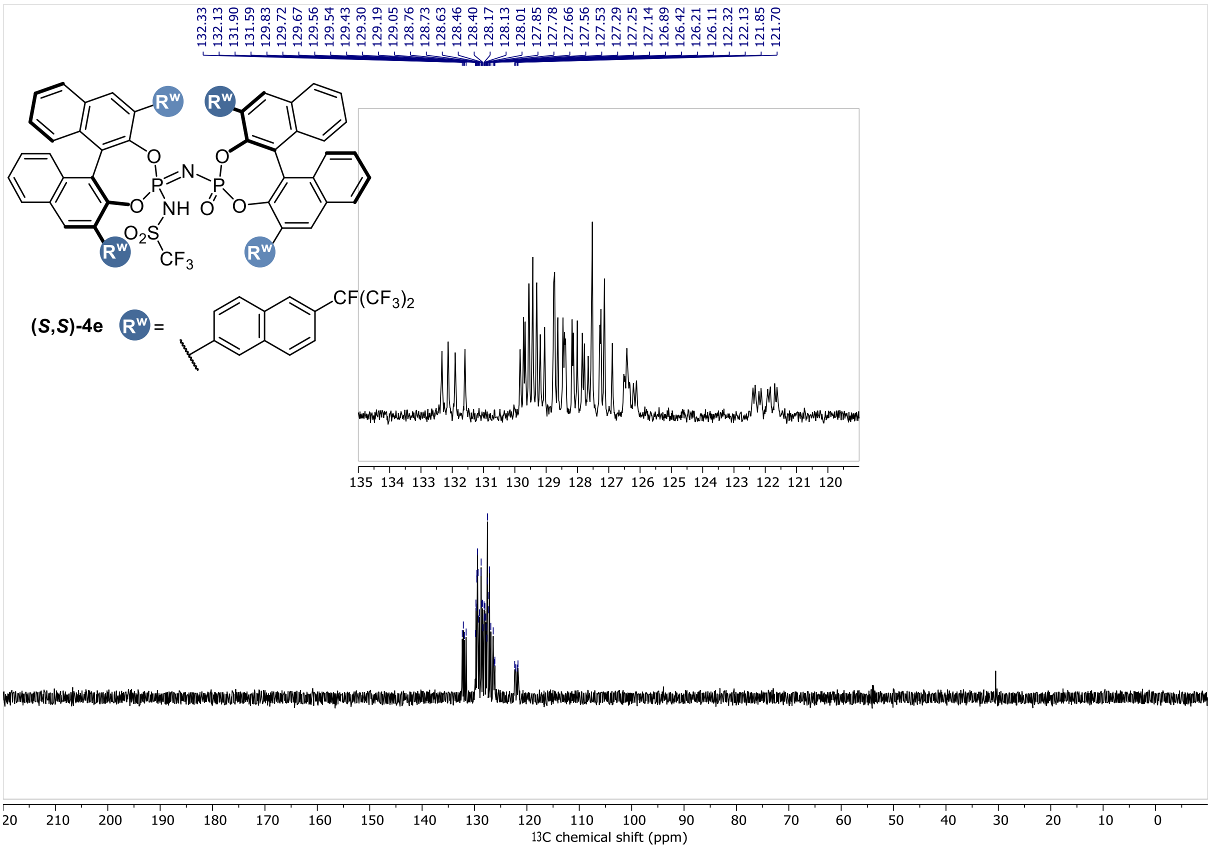

NMR spectra for compound **4e** *(continuation)*: DEPT-135 (126 MHz), in CD_2Cl_2.

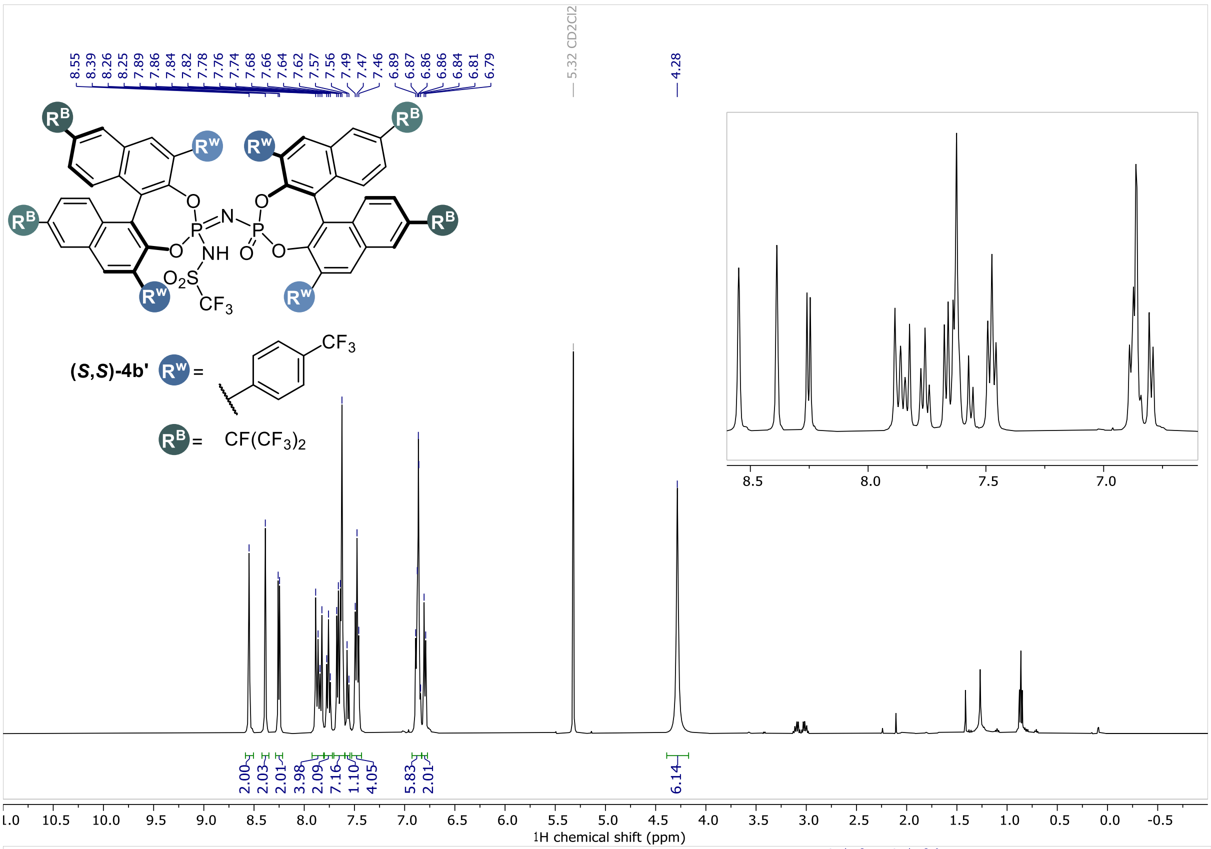

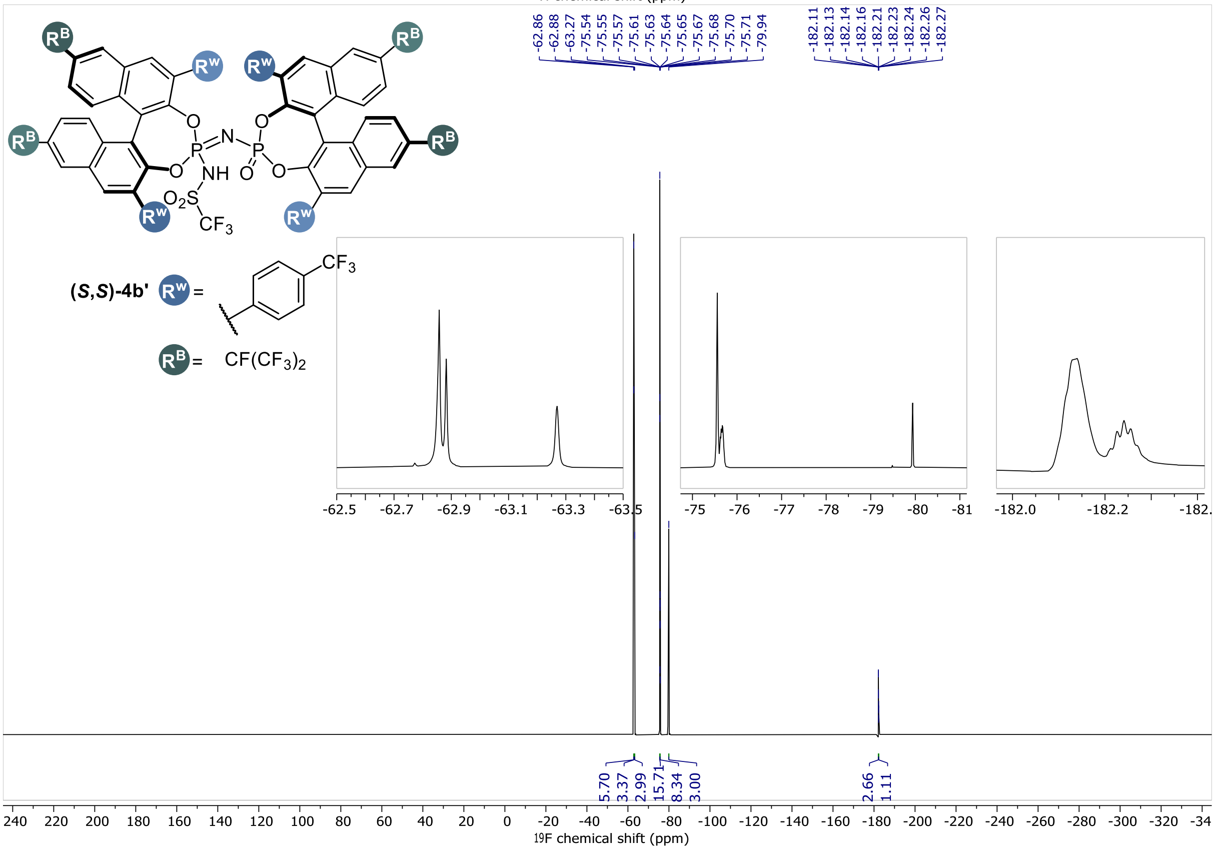

NMR spectra for compound **4b’**: ^{1}H (501 MHz) and ^{19}F (471 MHz), in CD_2Cl_2.

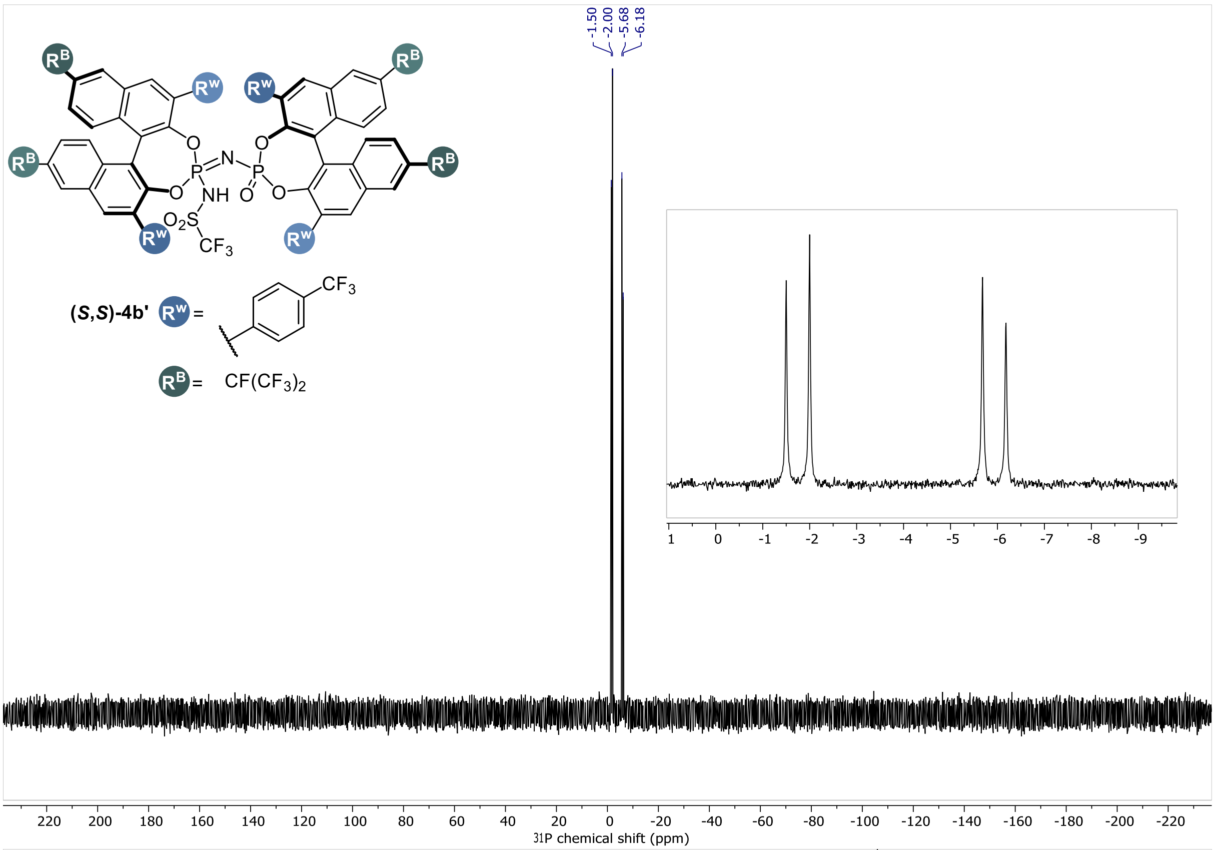

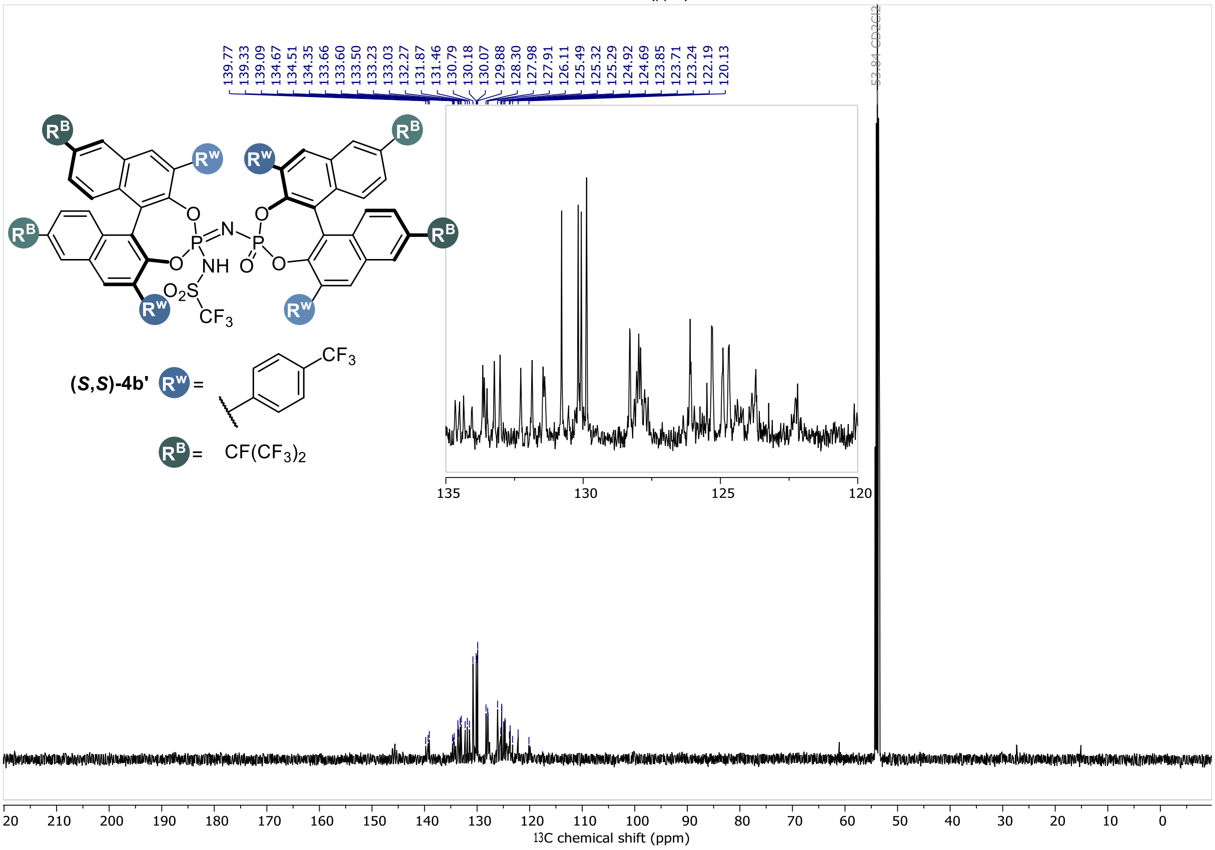

NMR spectra for compound **4b'** *(continuation)*: ^{31}P (203 MHz) and ^{13}C (126 MHz), in CD_2Cl_2.

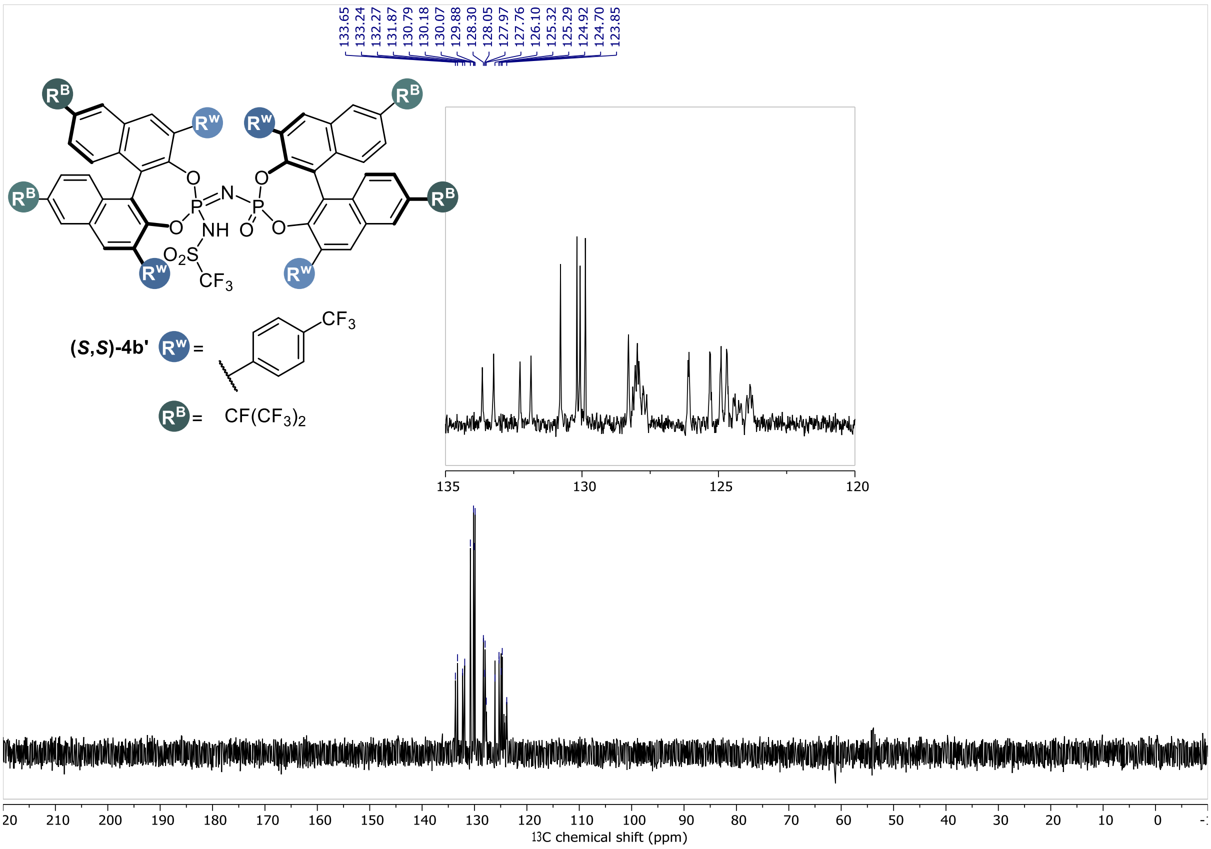

NMR spectra for compound **4b’** *(continuation)*: DEPT-135 (126 MHz), in CD_2Cl_2.

10. Copies of HPLC traces

(Chiralpak IB-3 column, Heptane/*i*-PrOH 95:5, 1 mL/min, 25 °C, 220 nm)

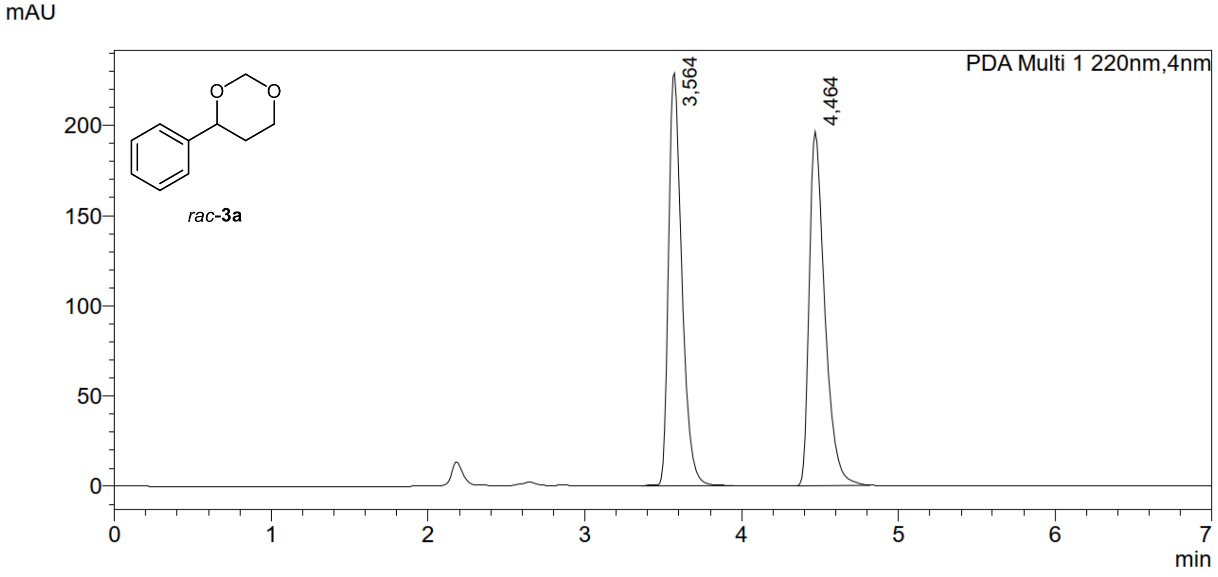

Peak#	Ret. Time	Area%
1	3,564	49,886
2	4,464	50,114
Total		100,000

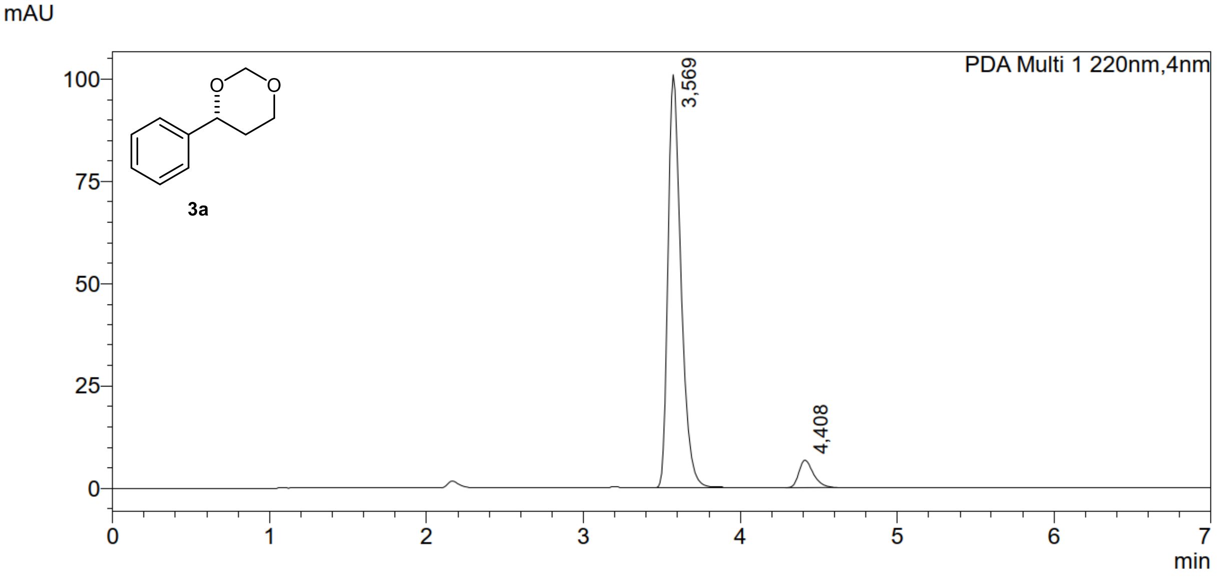

Peak#	Ret. Time	Area%
1	3,569	95,421
2	4,408	4,579
Total		100,000

(Chiralpak IC-3 column, Heptane/*i*-PrOH 98:2, 1 mL/min, 25 °C, 220 nm)

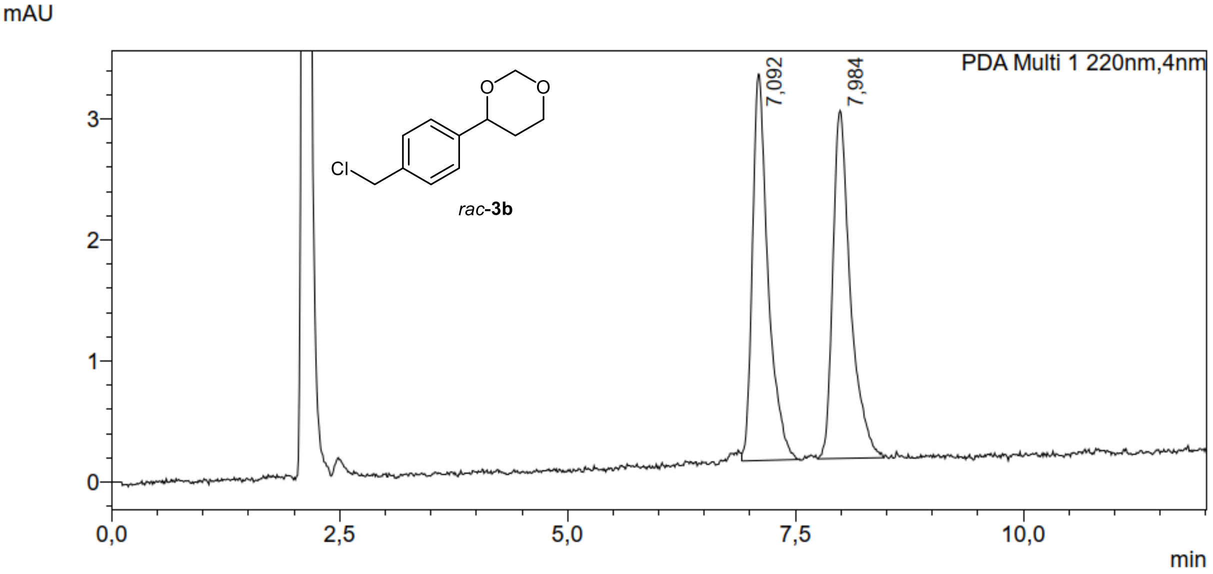

Peak#	Ret. Time	Area%
1	7,092	49,583
2	7,984	50,417
Total		100,000

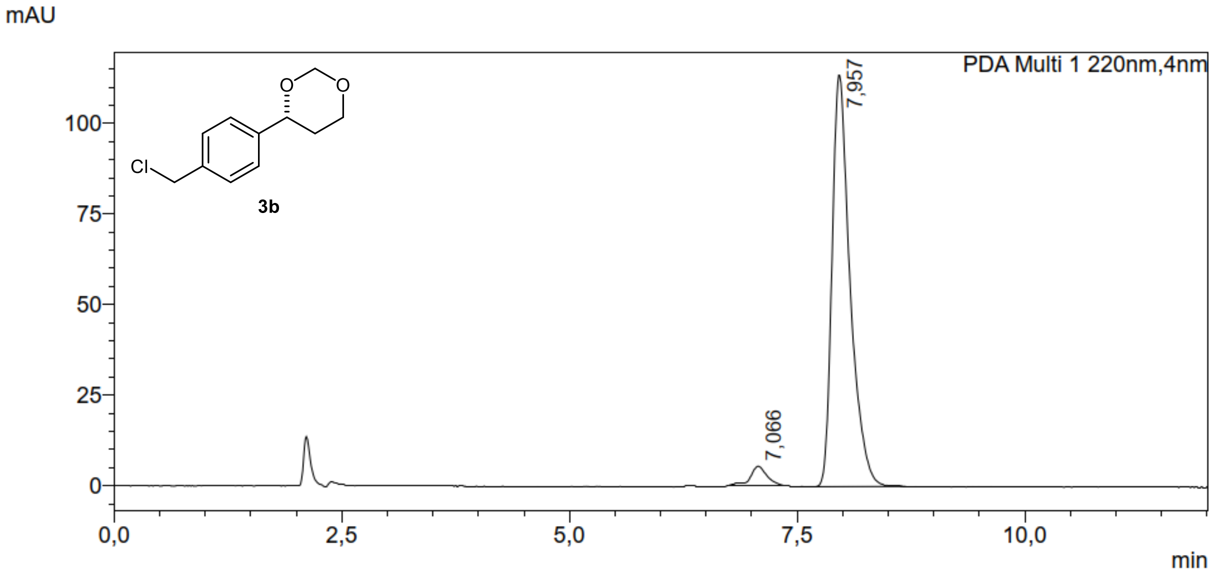

Peak#	Ret. Time	Area%
1	7,066	4,551
2	7,957	95,449
Total		100,000

(Chiralpak AD-3 column, Heptane/*i*-PrOH 97:3, 0.5 mL/min, 25 °C, 220 nm)

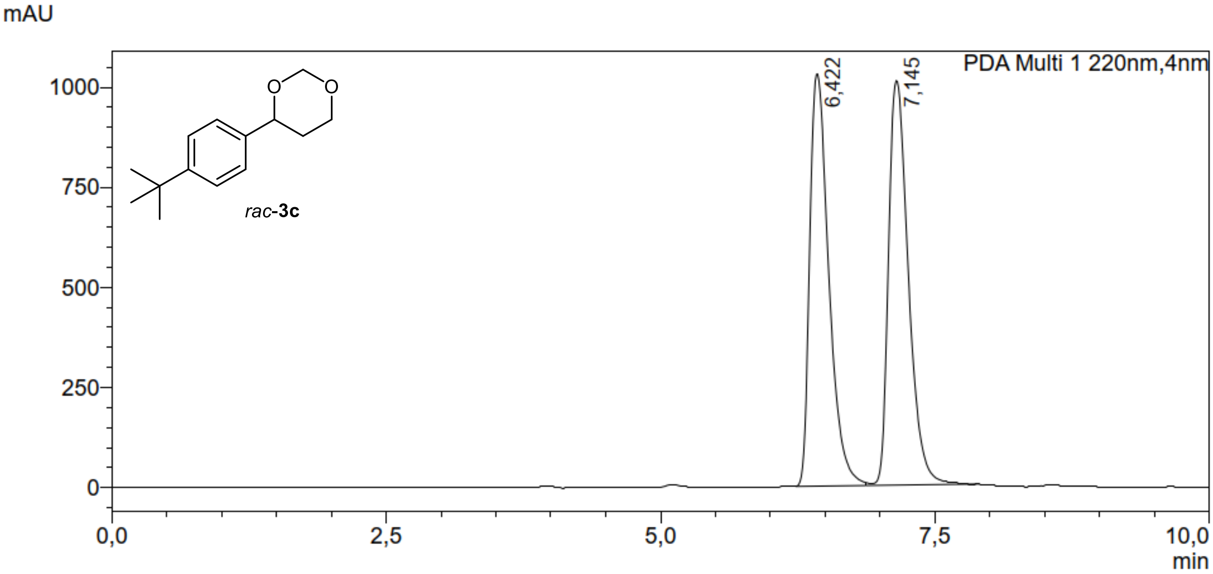

Peak#	Ret. Time	Area%
1	6,422	49,484
2	7,145	50,516
Total		100,000

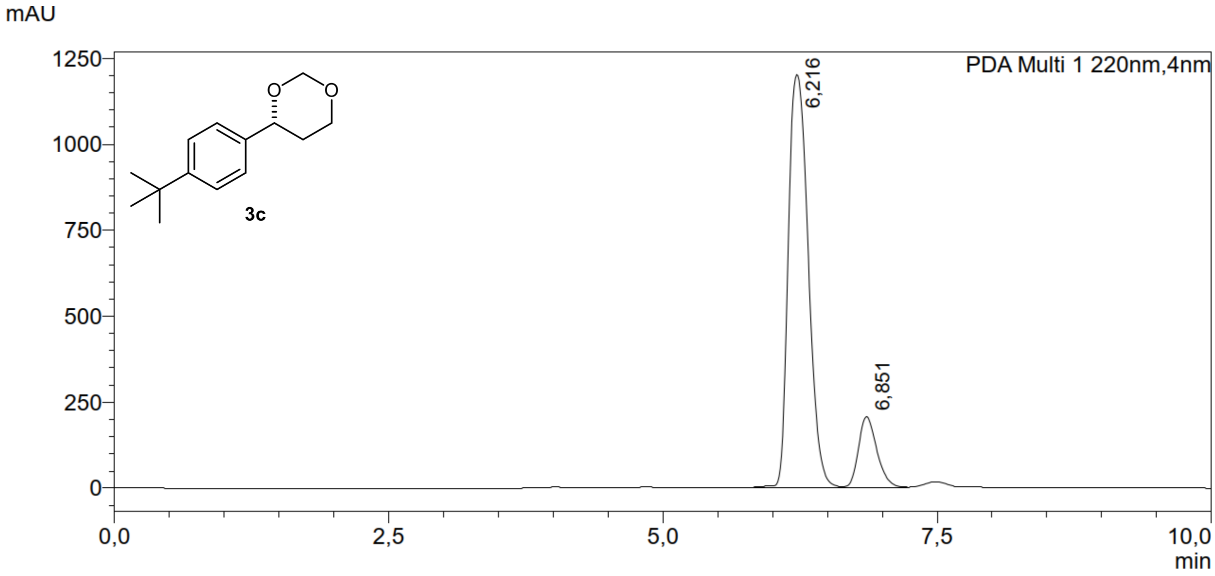

Peak#	Ret. Time	Area%
1	6,216	91,308
2	6,851	8,692
Total		100,000

(Chiralpak IB-3 column, Heptane/*i*-PrOH 98:2, 1 mL/min, 25 °C, 220 nm)

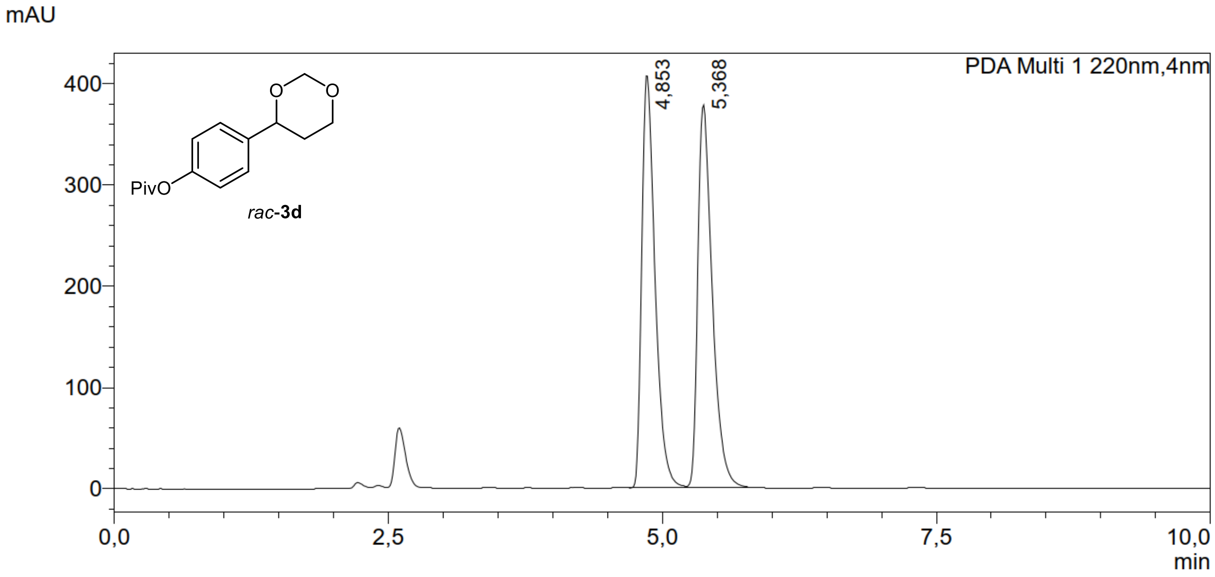

Peak#	Ret. Time	Area%
1	4,853	49,709
2	5,368	50,291
Total		100,000

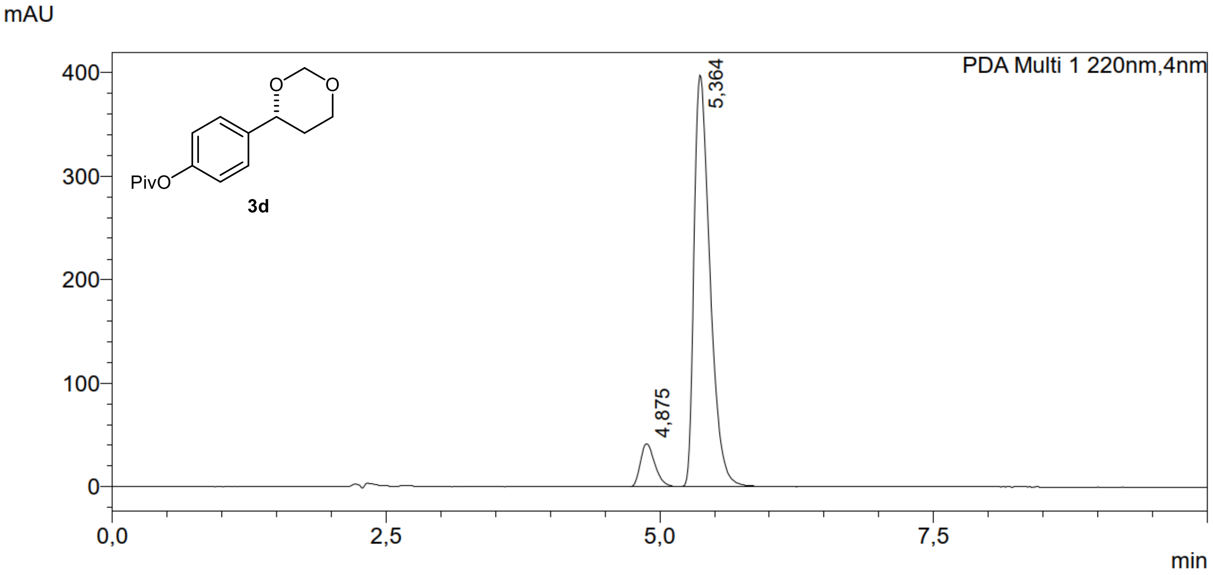

Peak#	Ret. Time	Area%
1	4,875	8,803
2	5,364	91,197
Total		100,000

(Chiralcel OJ-3 column, Heptane/*i*-PrOH 95:5, 1 mL/min, 25 °C, 220 nm)

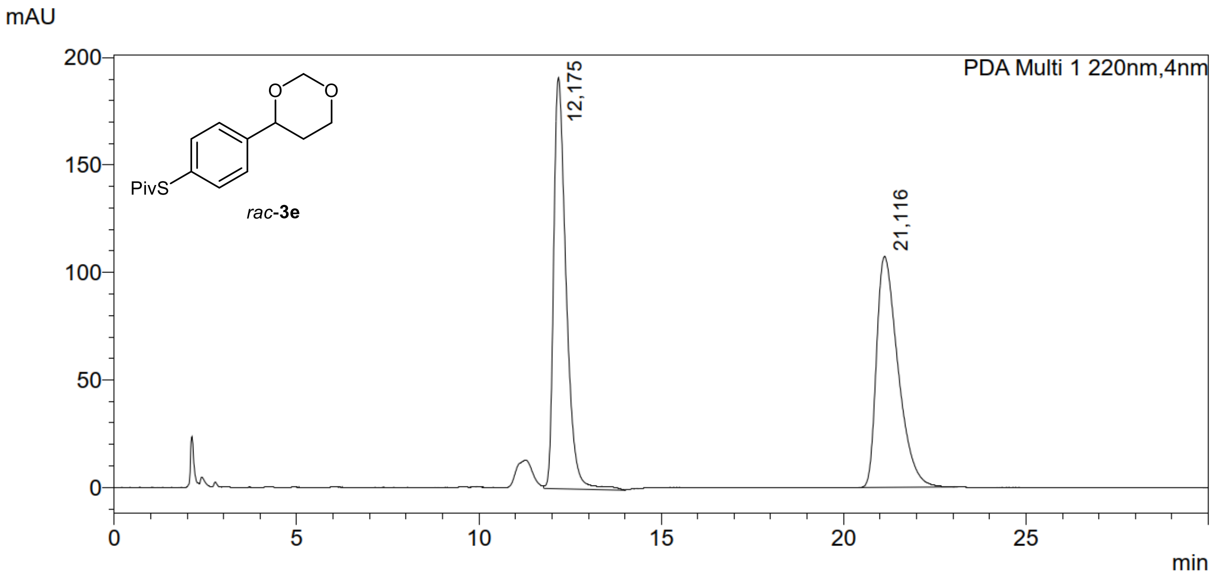

Peak#	Ret. Time	Area%
1	12,175	50,650
2	21,116	49,350
Total		100,000

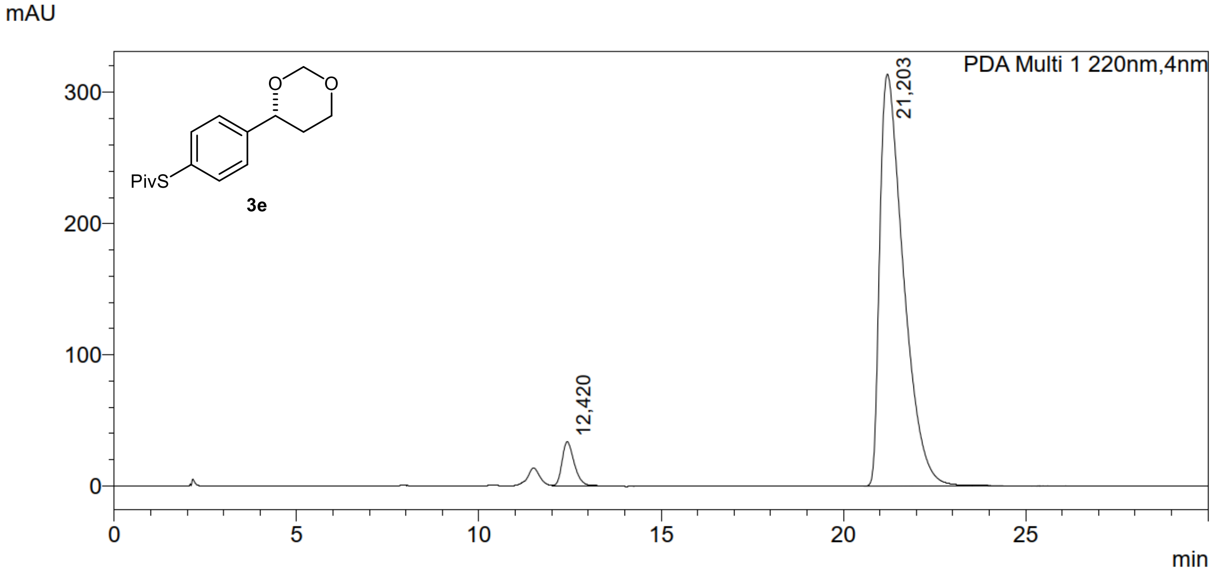

Peak#	Ret. Time	Area%
1	12,420	5,193
2	21,203	94,807
Total		100,000

(Chiralpak IC-3 column, Heptane/*i*-PrOH 98:2, 1 mL/min, 25 °C, 220 nm)

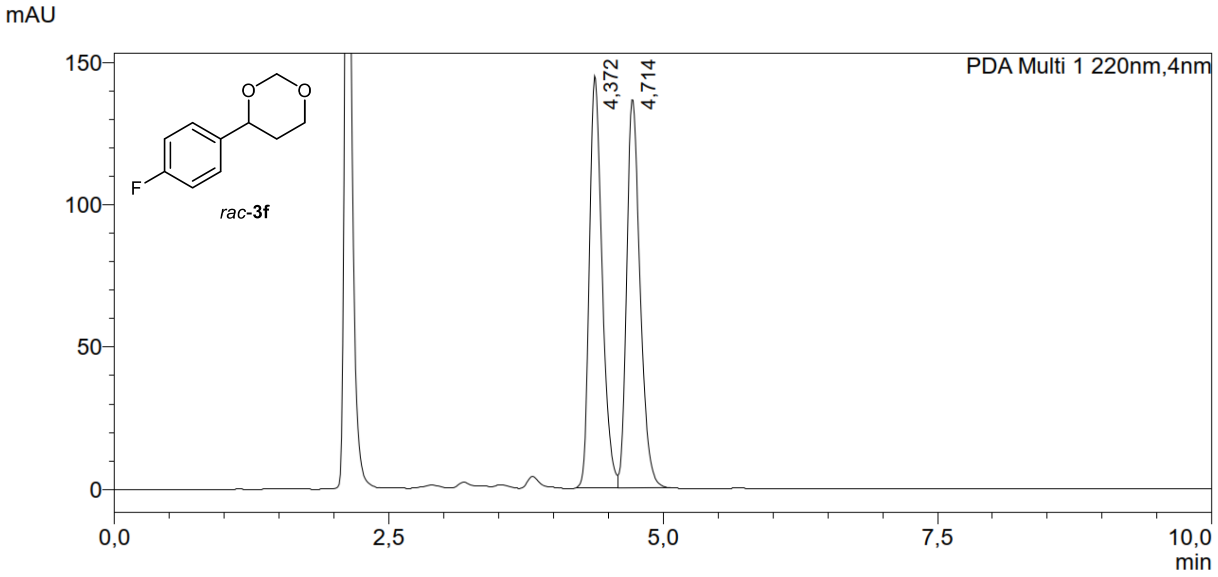

Peak#	Ret. Time	Area%
1	4,372	50,187
2	4,714	49,813
Total		100,000

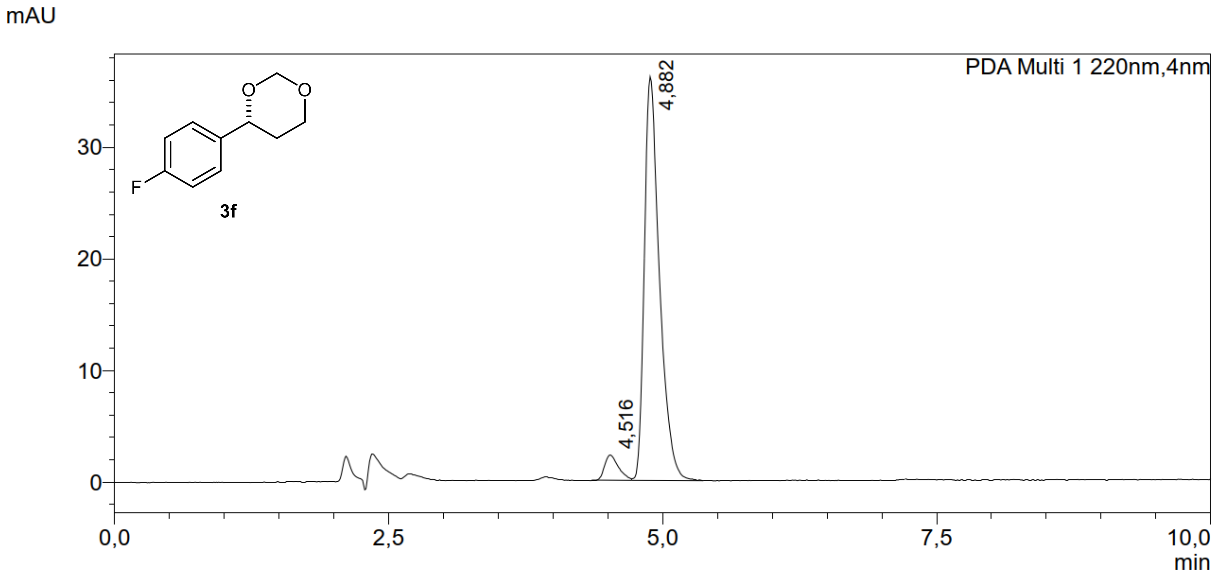

Peak#	Ret. Time	Area%
1	4,516	5,441
2	4,882	94,559
Total		100,000

(Chiralpak IC-3 column, Heptane/*i*-PrOH 97:3, 0.5 mL/min, 25 °C, 220 nm)

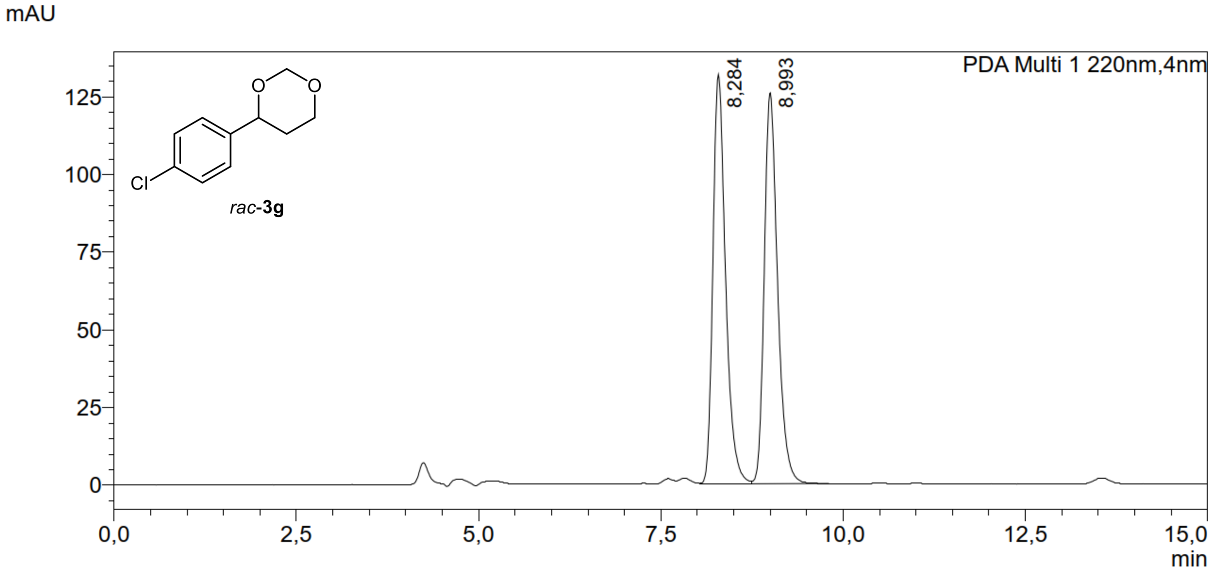

Peak#	Ret. Time	Area%
1	8,284	49,852
2	8,993	50,148
Total		100,000

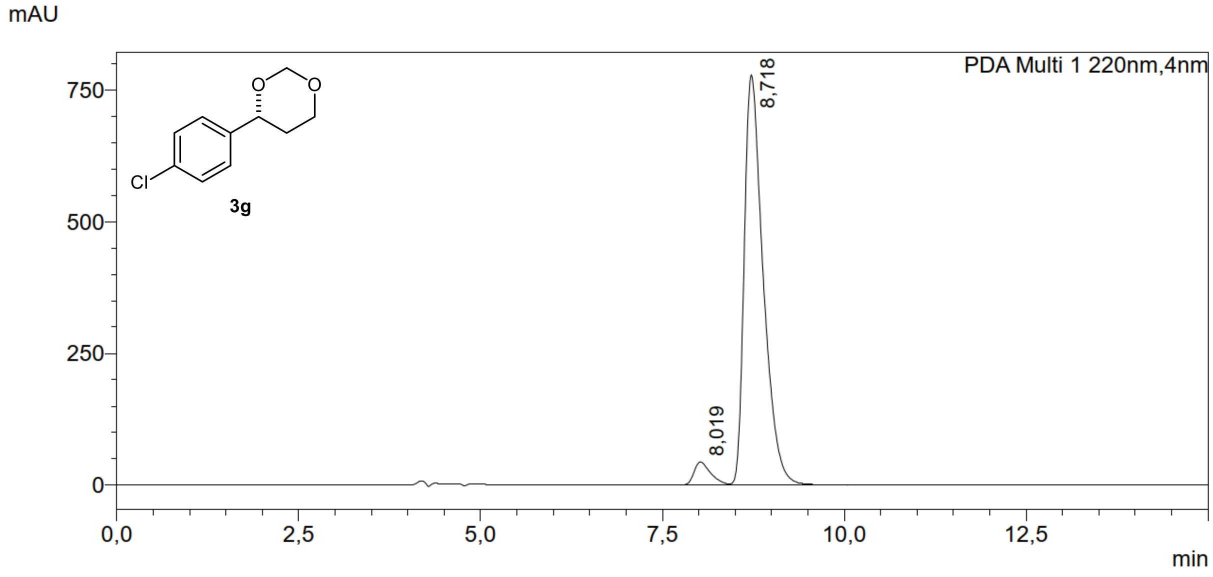

Peak#	Ret. Time	Area%
1	8,019	4,765
2	8,718	95,235
Total		100,000

(Chiralpak IC-3 column, Heptane/*i*-PrOH 97:3, 0.5 mL/min, 25 °C, 220 nm)

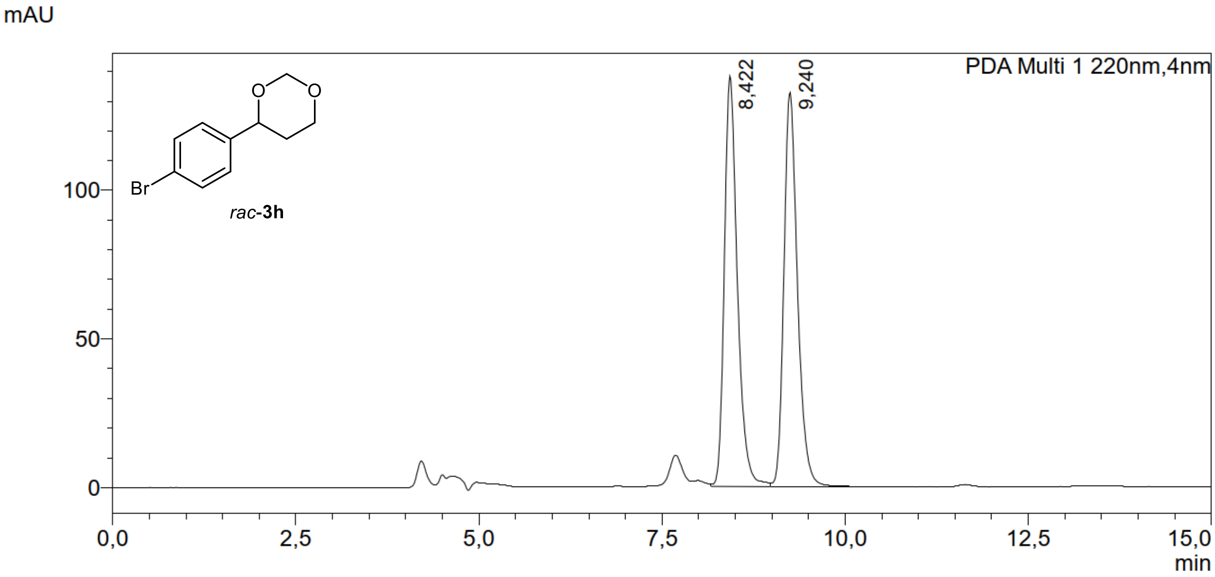

Peak#	Ret. Time	Area%
1	8,422	49,904
2	9,240	50,096
Total		100,000

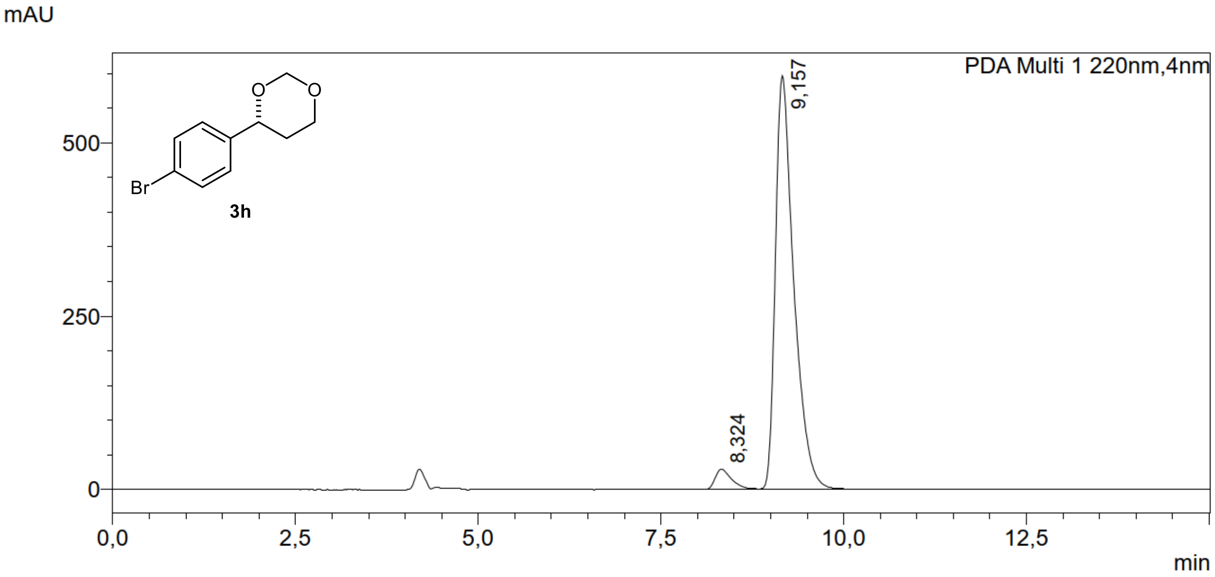

Peak#	Ret. Time	Area%
1	8,324	4,399
2	9,157	95,601
Total		100,000

(Chiralcel OJ-3 column, Heptane/*i*-PrOH 95:5, 1 mL/min, 25 °C, 220 nm)

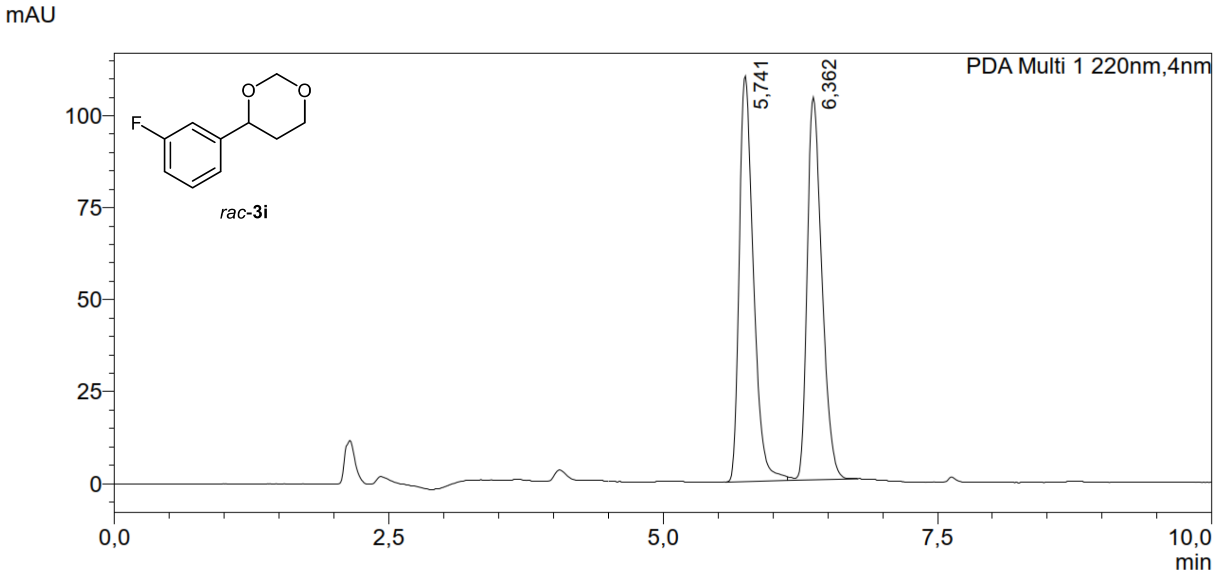

Peak#	Ret. Time	Area%
1	5,741	50,513
2	6,362	49,487
Total		100,000

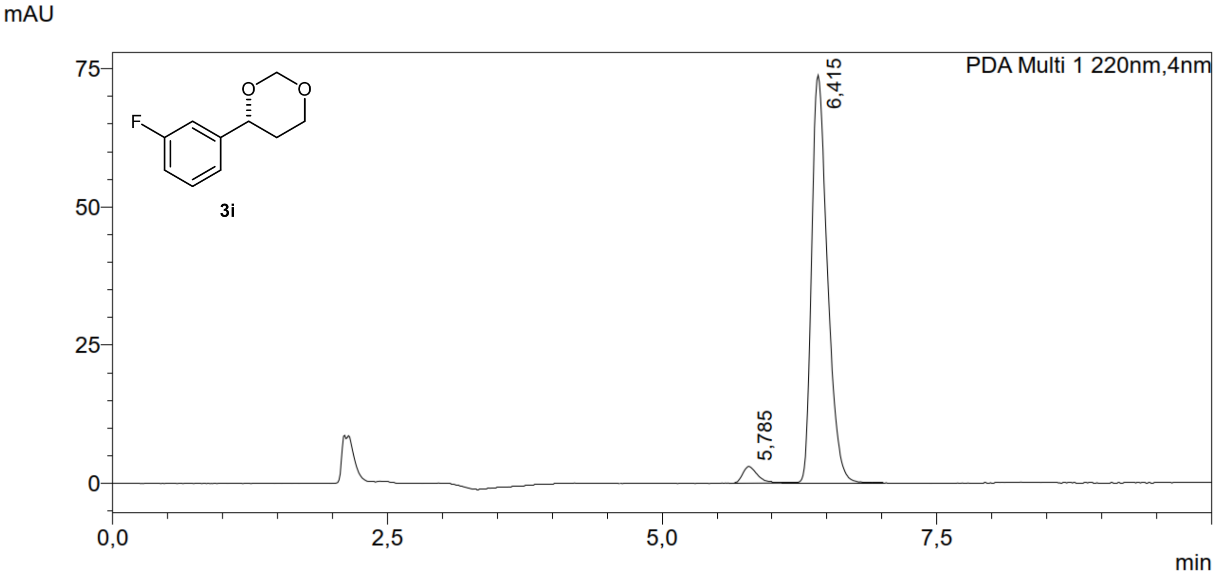

Peak#	Ret. Time	Area%
1	5,785	3,741
2	6,415	96,259
Total		100,000

(Chiralpak IC-3 column, Heptane/*i*-PrOH 97:3, 0.5 mL/min, 25 °C, 220 nm)

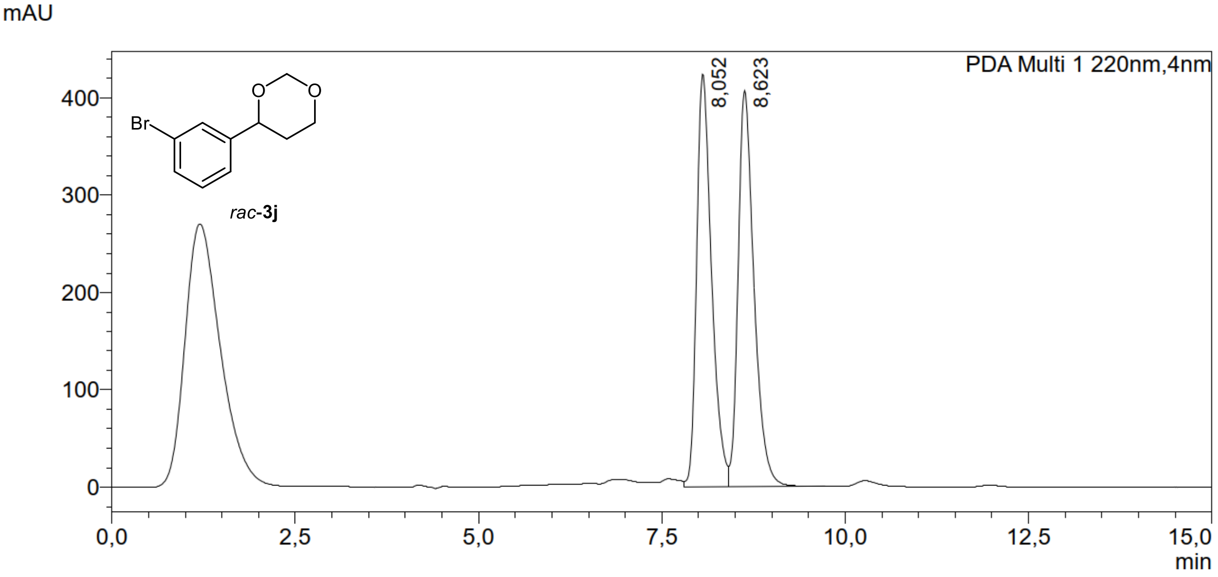

Peak#	Ret. Time	Area%
1	8,052	49,431
2	8,623	50,569
Total		100,000

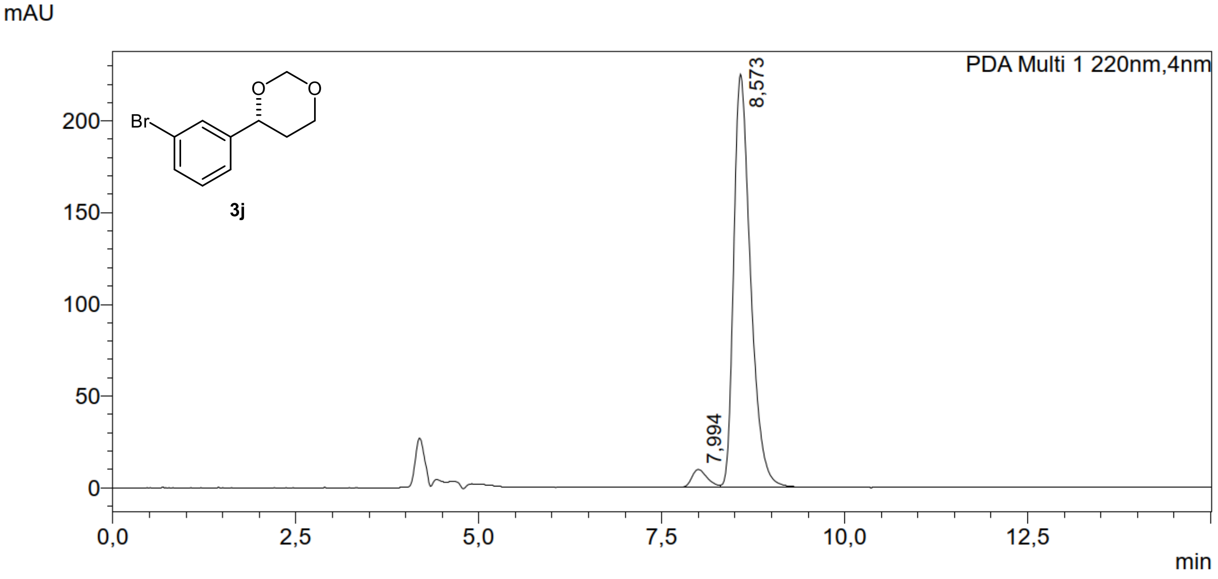

Peak#	Ret. Time	Area%
1	7,994	3,945
2	8,573	96,055
Total		100,000

(Chiralpak IB-3 column, Heptane/*i*-PrOH 95:5, 1 mL/min, 25 °C, 220 nm)

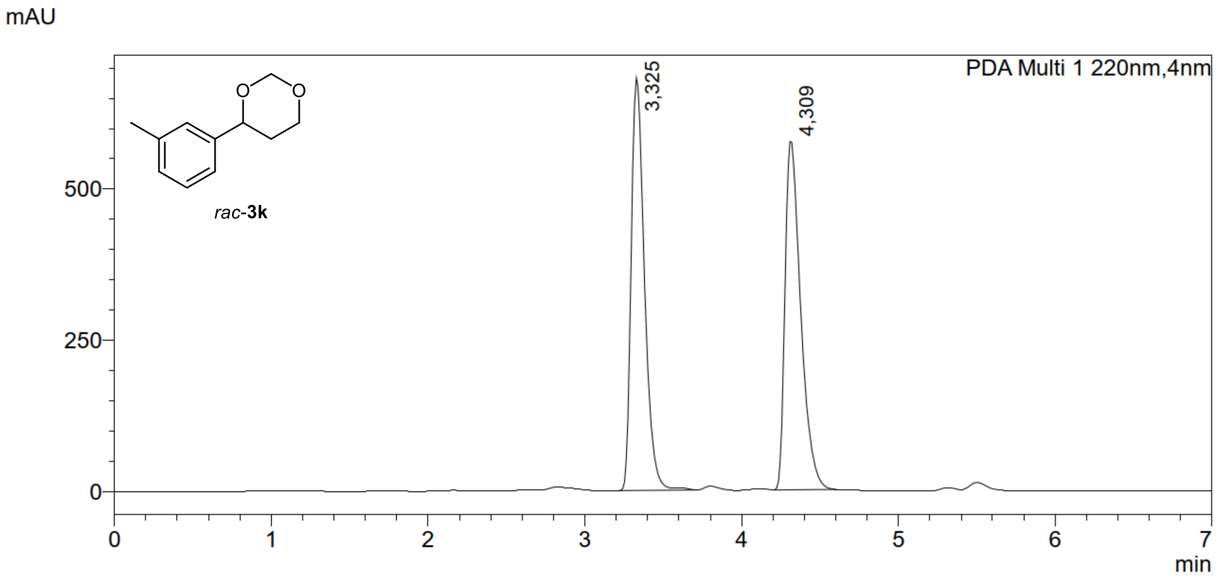

Peak#	Ret. Time	Area%
1	3,325	49,867
2	4,309	50,133
Total		100,000

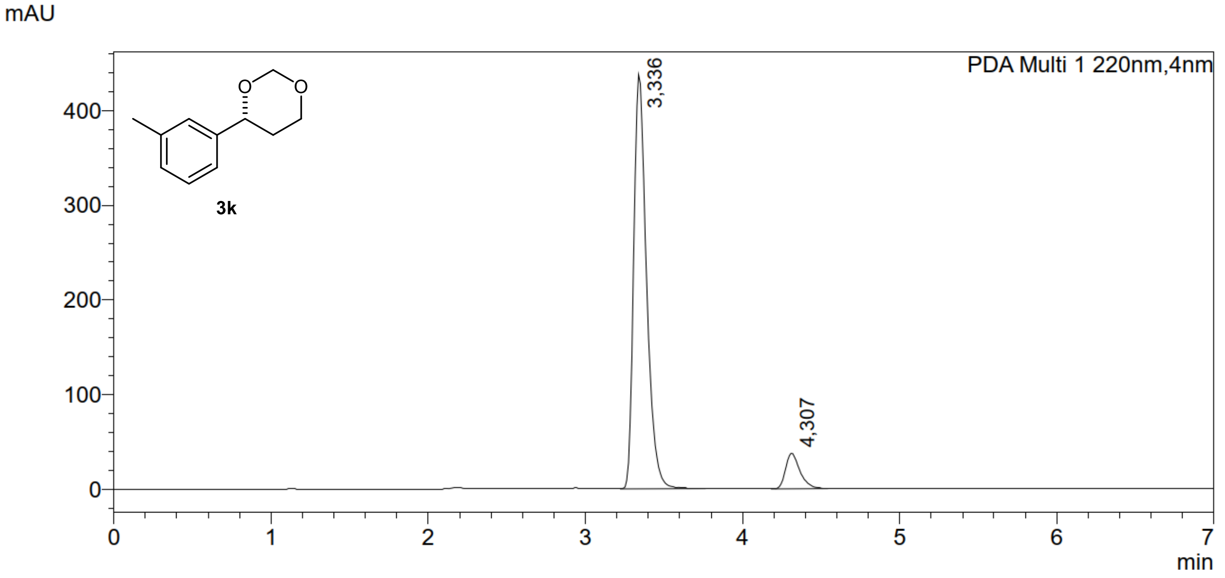

Peak#	Ret. Time	Area%
1	3,336	93,151
2	4,307	6,849
Total		100,000

(Chiralpak IB-3 column, Heptane/*i*-PrOH 95:5, 1 mL/min, 25 °C, 220 nm)

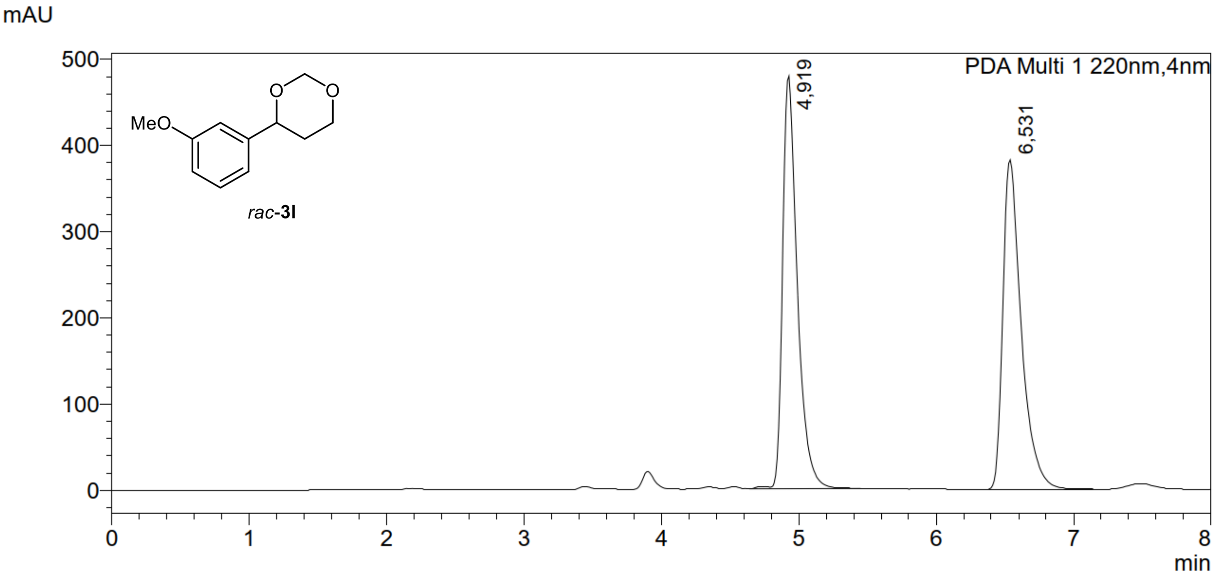

Peak#	Ret. Time	Area%
1	4,919	50,028
2	6,531	49,972
Total		100,000

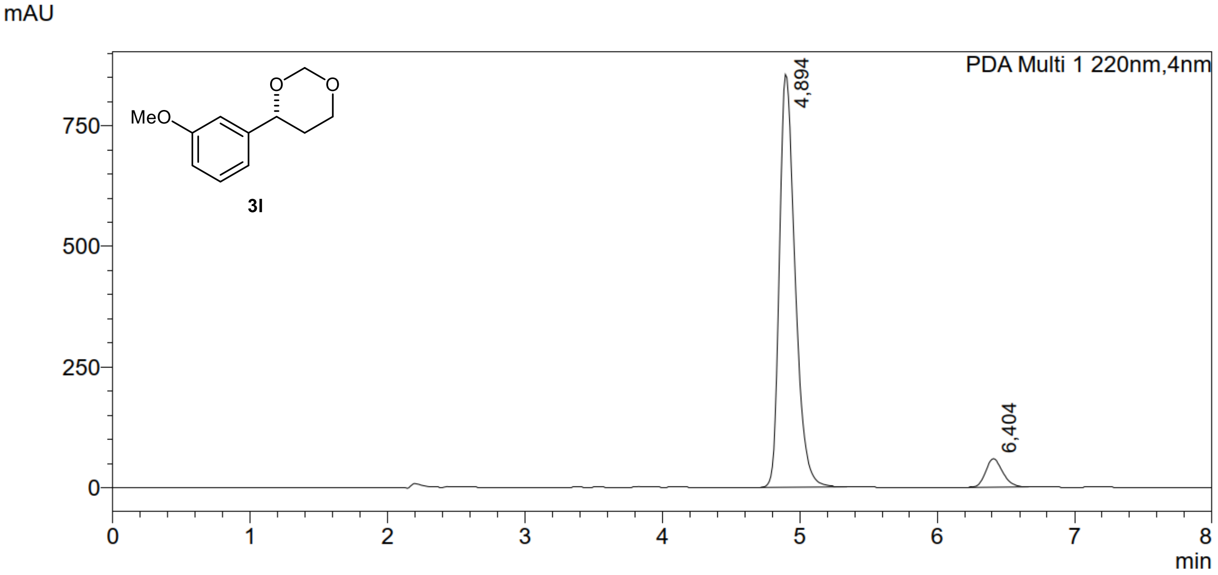

Peak#	Ret. Time	Area%
1	4,894	93,044
2	6,404	6,956
Total		100,000

(Chiralcel OJ-3 column, Heptane/*i*-PrOH 95:5, 1 mL/min, 25 °C, 220 nm)

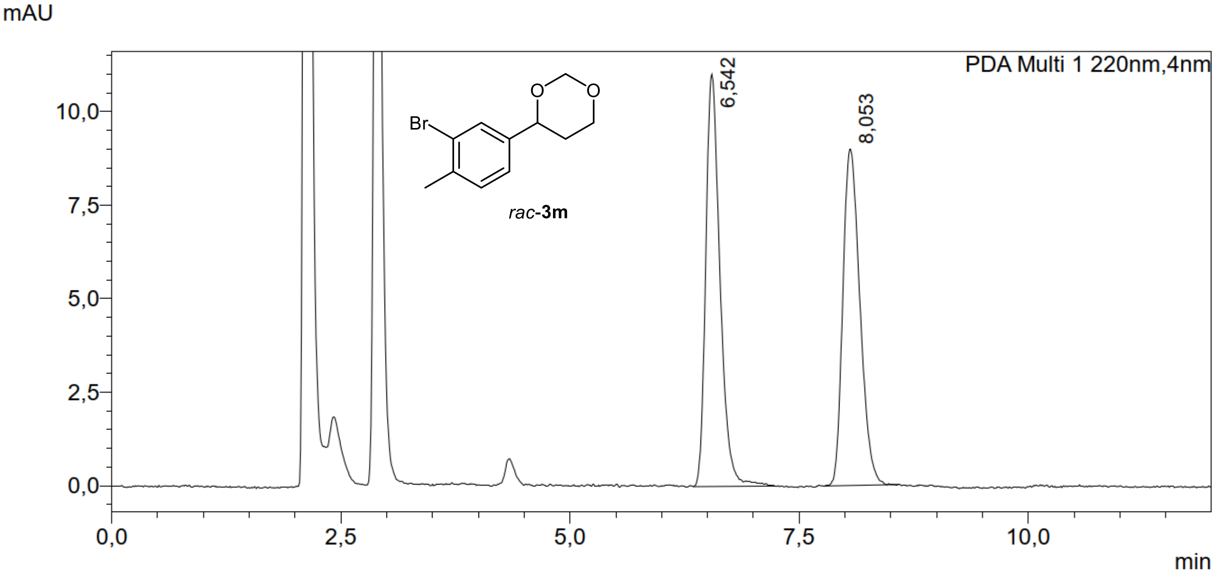

Peak#	Ret. Time	Area%
1	6,542	50,512
2	8,053	49,488
Total		100,000

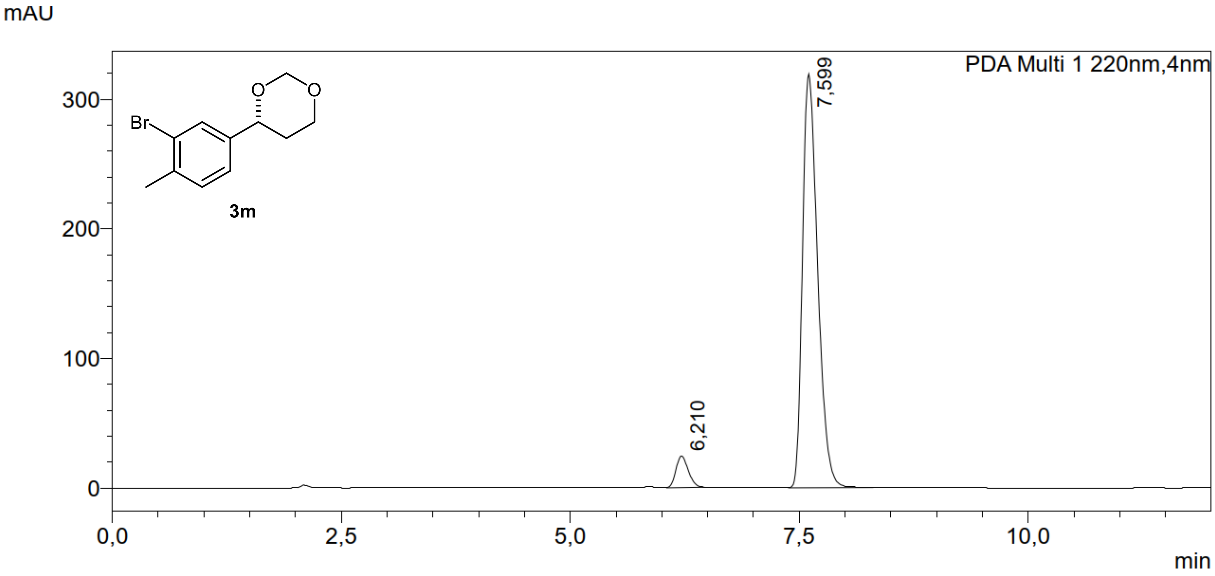

Peak#	Ret. Time	Area%
1	6,210	5,849
2	7,599	94,151
Total		100,000

(Chiralpak IB-3 column, Heptane/*i*-PrOH 80:20, 1 mL/min, 25 °C, 274 nm)

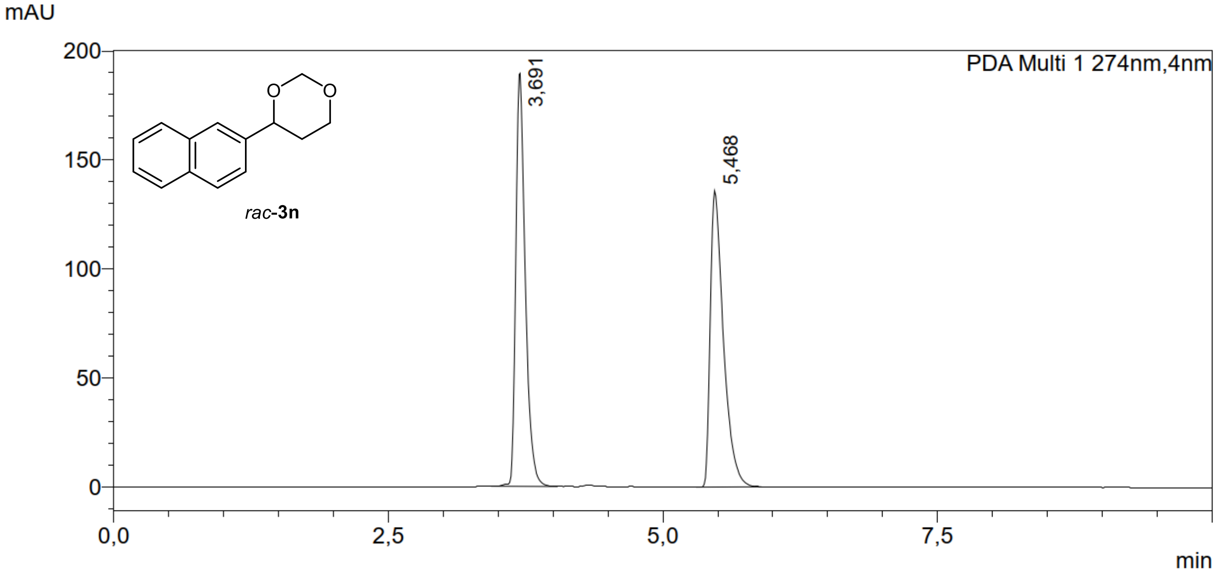

Peak#	Ret. Time	Area%
1	3,691	50,072
2	5,468	49,928
Total		100,000

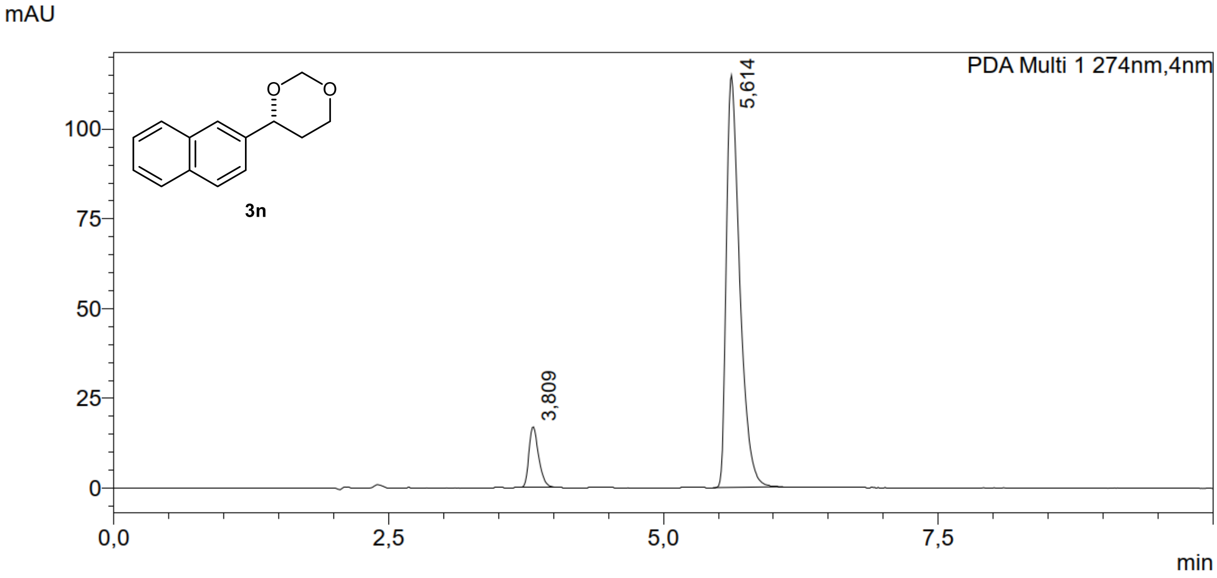

Peak#	Ret. Time	Area%
1	3,809	9,601
2	5,614	90,399
Total		100,000

(Chiralcel OJ-3 column, Heptane/*i*-PrOH 95:5, 1 mL/min, 25 °C, 220 nm)

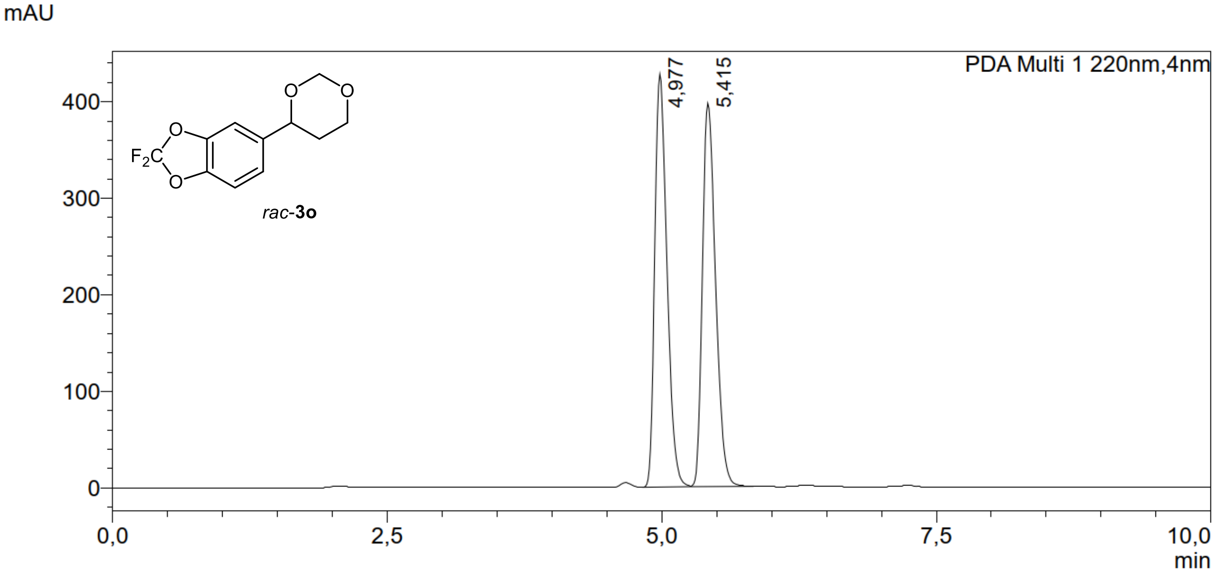

Peak#	Ret. Time	Area%
1	4,977	49,855
2	5,415	50,145
Total		100,000

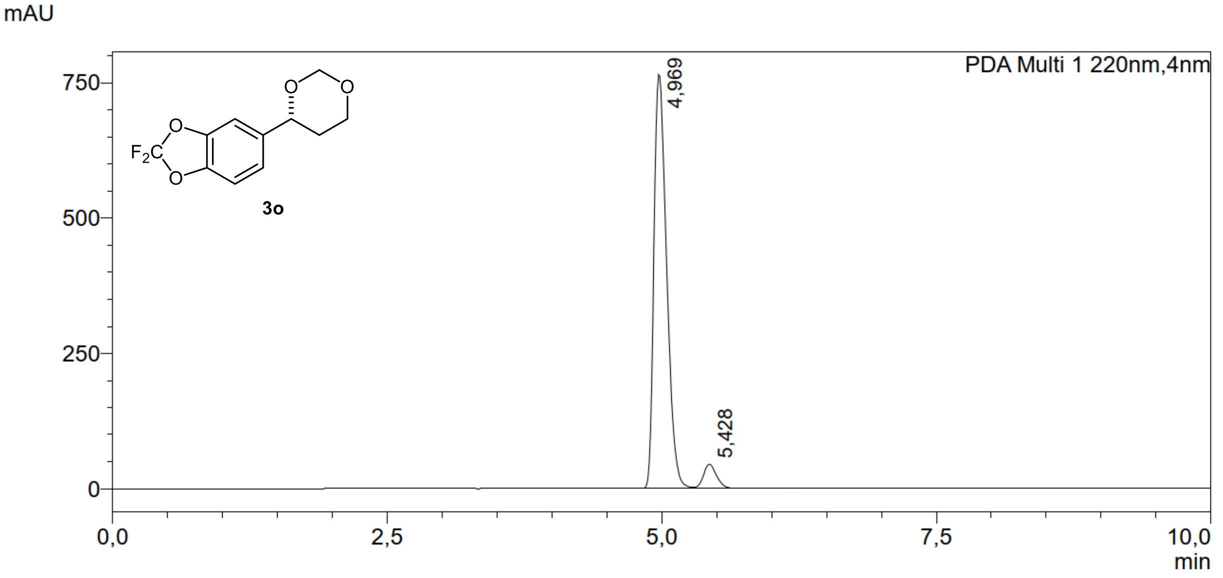

Peak#	Ret. Time	Area%
1	4,969	94,335
2	5,428	5,665
Total		100,000

(Chiralpak IC-3 column, Heptane/*i*-PrOH 97:3, 0.5 mL/min, 25 °C, 220 nm)

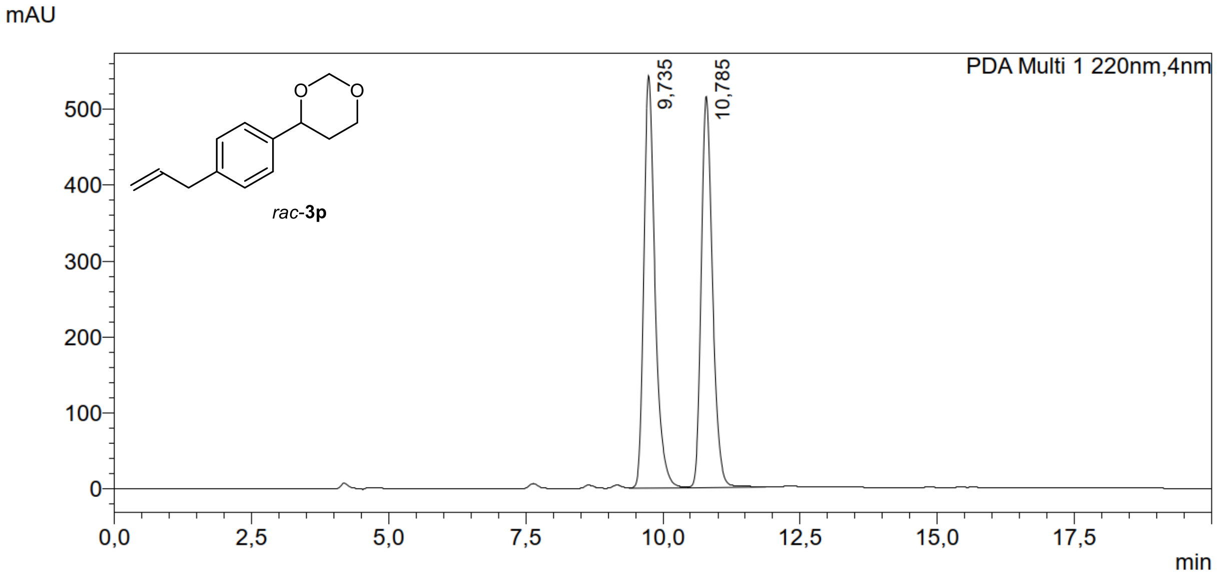

Peak#	Ret. Time	Area%
1	9,735	50,958
2	10,785	49,042
Total		100,000

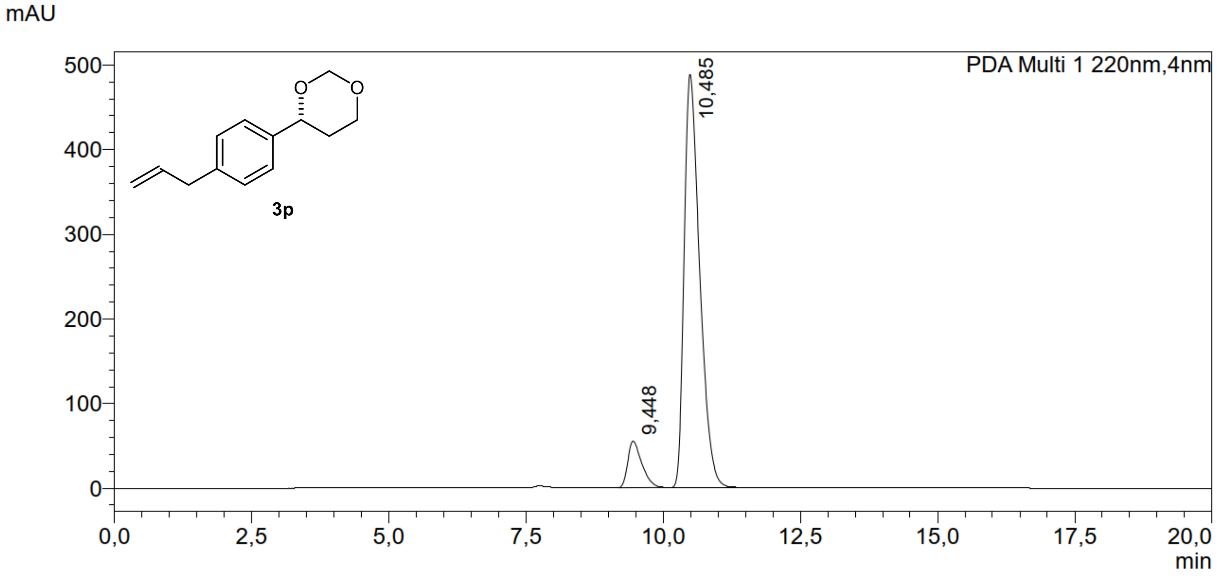

Peak#	Ret. Time	Area%
1	9,448	9,220
2	10,485	90,780
Total		100,000

(Chiralpak IB-3 column, Heptane/*i*-PrOH 95:5, 1 mL/min, 25 °C, 220 nm)

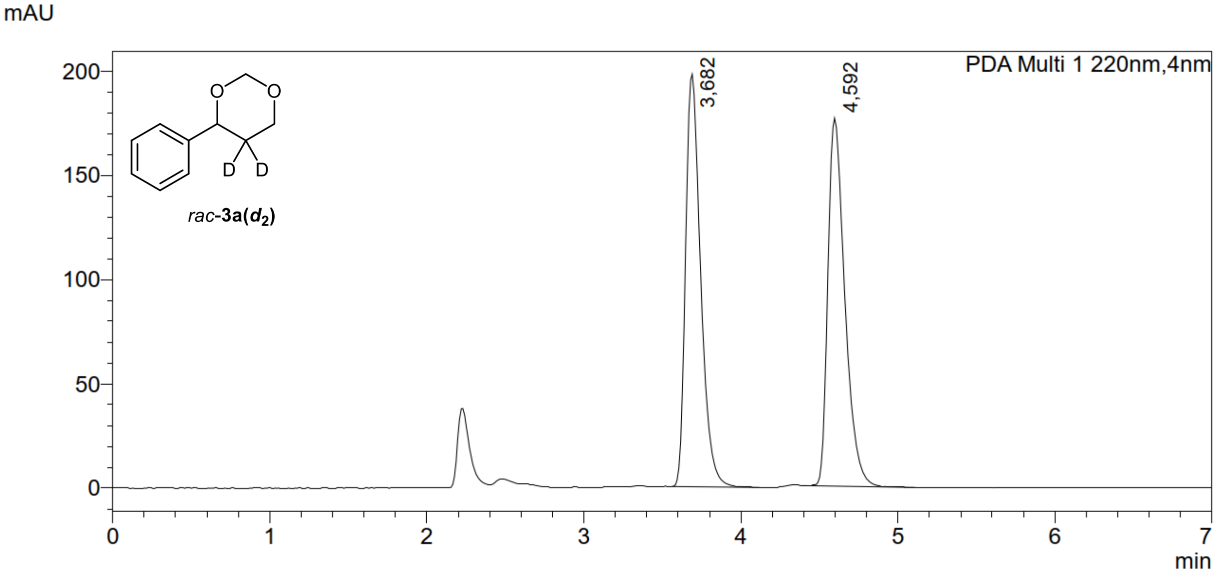

Peak#	Ret. Time	Area%
1	3,682	49,943
2	4,592	50,057
Total		100,000

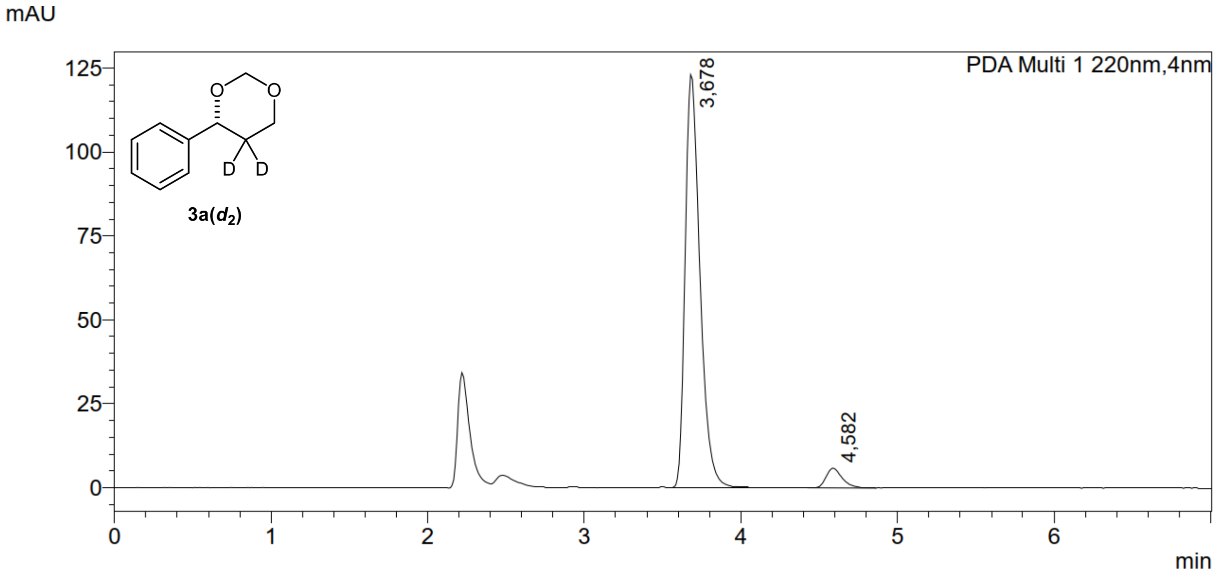

Peak#	Ret. Time	Area%
1	3,678	95,071
2	4,582	4,929
Total		100,000

(Chiralpak IB-3 column, Heptane/*i*-PrOH 95:5, 1 mL/min, 25 °C, 220 nm)

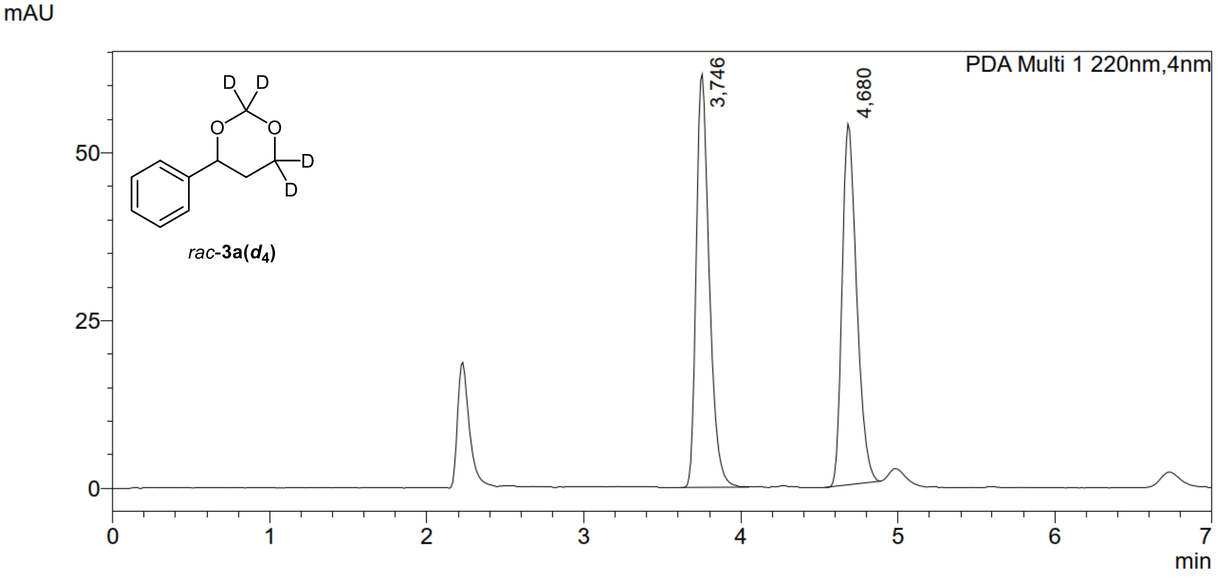

Peak#	Ret. Time	Area%
1	3,746	50,735
2	4,680	49,265
Total		100,000

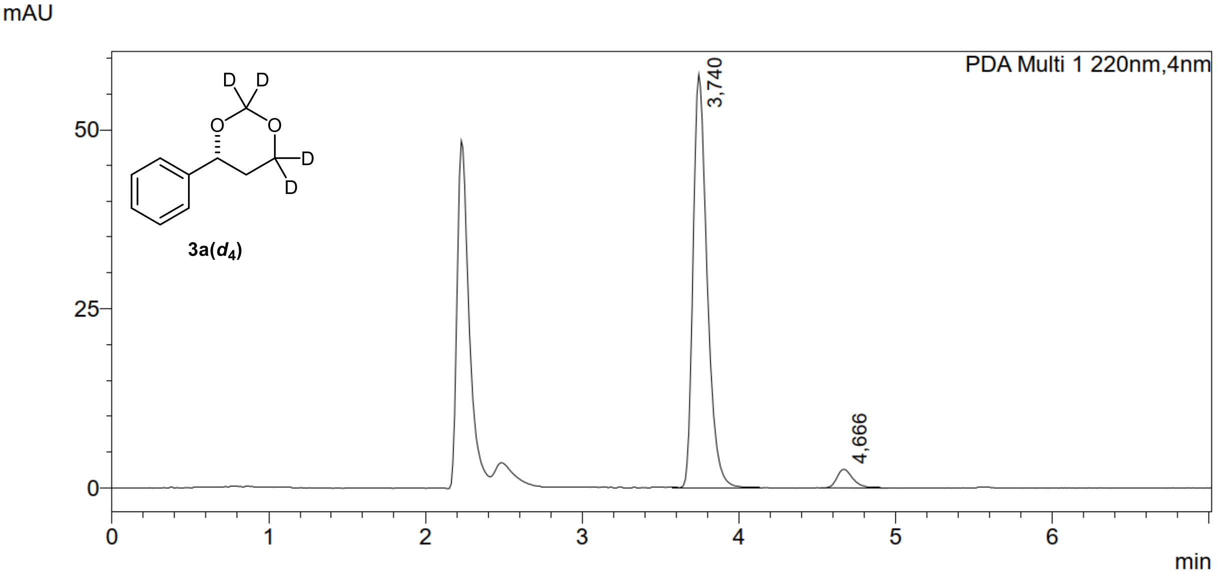

Peak#	Ret. Time	Area%
1	3,740	95,143
2	4,666	4,857
Total		100,000

(Chiralpak IB-3 column, Heptane/*i*-PrOH 95:5, 1 mL/min, 25 °C, 220 nm)

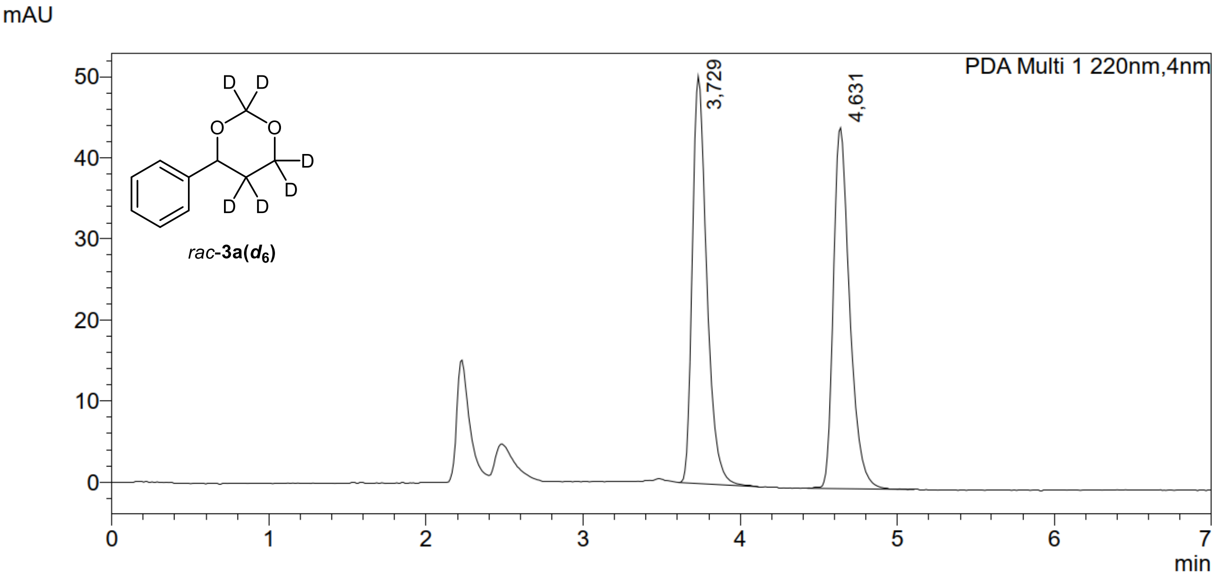

Peak#	Ret. Time	Area%
1	3,729	50,014
2	4,631	49,986
Total		100,000

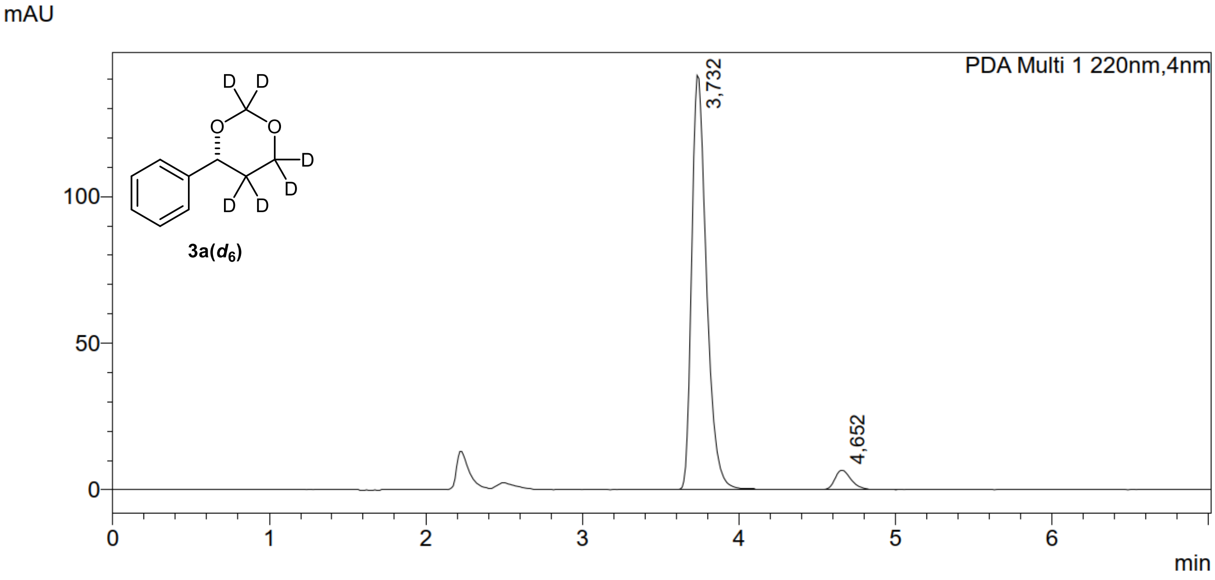

Peak#	Ret. Time	Area%
1	3,732	95,040
2	4,652	4,960
Total		100,000

(Chiralpak AD-3 column, Heptane/*i*-PrOH 97:3, 0.5 mL/min, 25 °C, 220 nm)

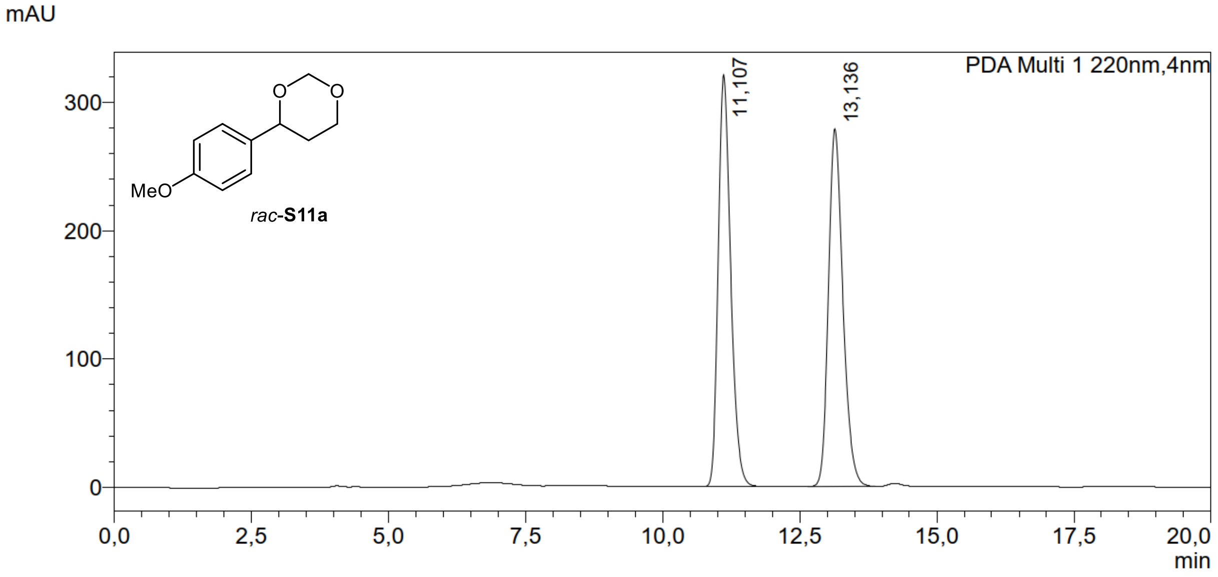

Peak#	Ret. Time	Area%
1	11,107	49,748
2	13,136	50,252
Total		100,000

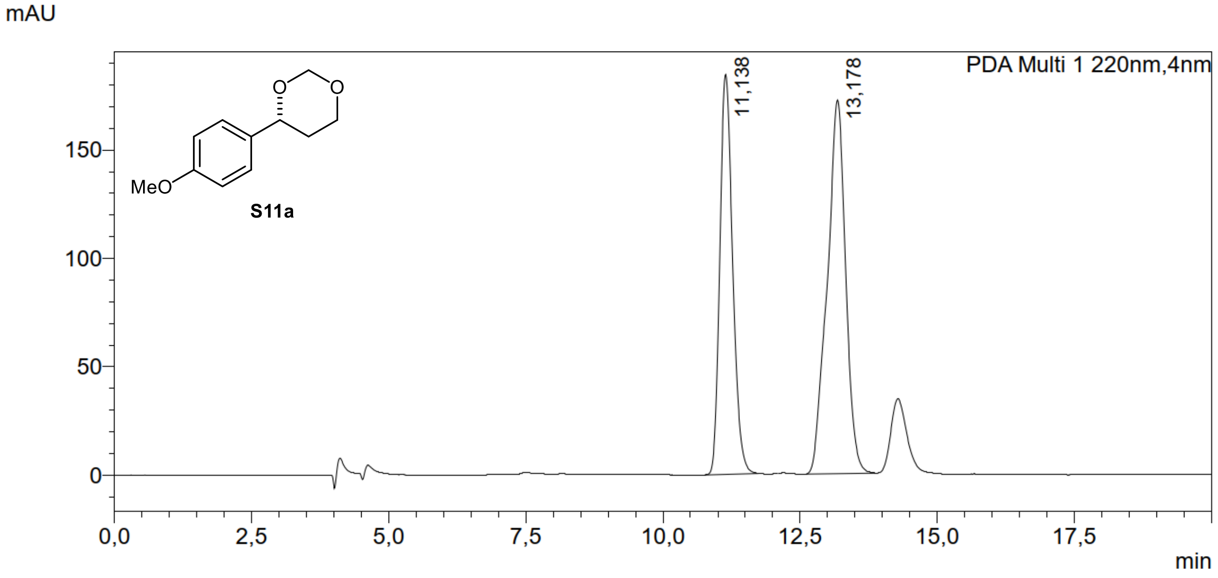

Peak#	Ret. Time	Area%
1	11,138	42,616
2	13,178	57,384
Total		100,000

(Kromasil Amycoat RP column, CH_3CN/H_2O 50:50, 1 mL/min, 25 °C, 220 nm)

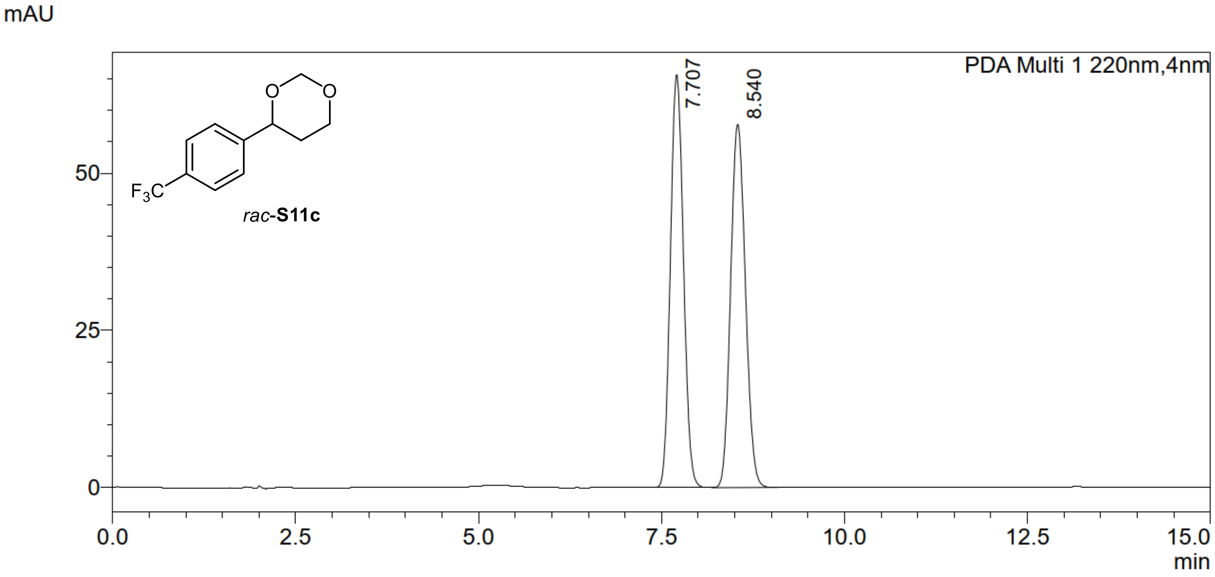

Peak#	Ret. Time	Area%
1	7.707	50.077
2	8.540	49.923
Total		100.000

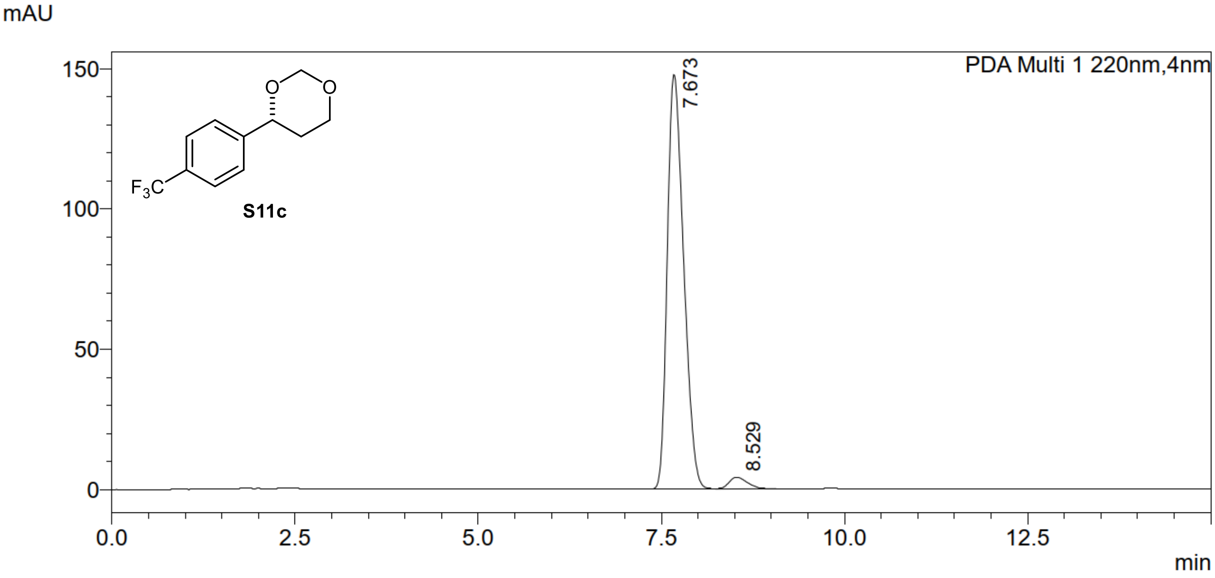

Peak#	Ret. Time	Area%
1	7.673	96.943
2	8.529	3.057
Total		100.000

(Chiralpak AD-3 column, Heptane/*i*-PrOH 95:5, 1 mL/min, 25 °C, 220 nm)

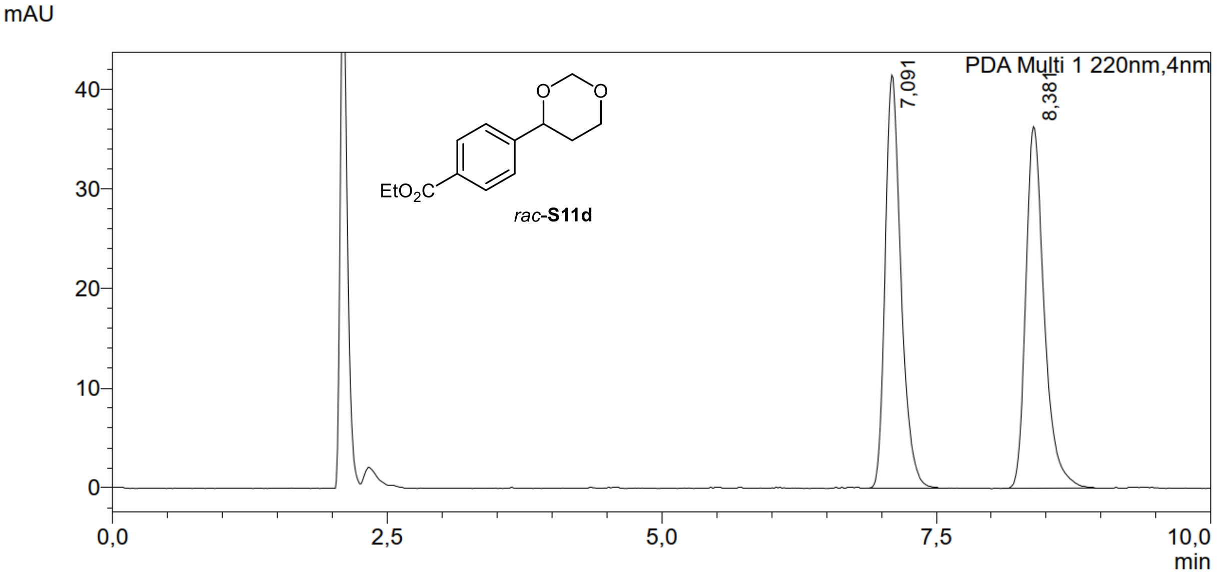

Peak#	Ret. Time	Area%
1	7,091	49,614
2	8,381	50,386
Total		100,000

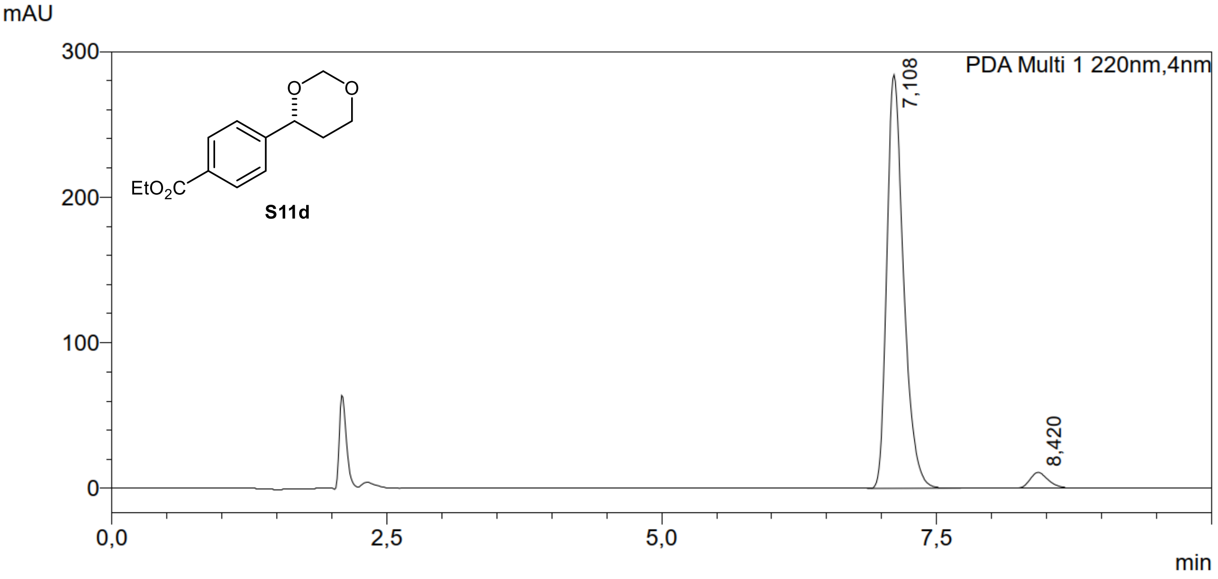

Peak#	Ret. Time	Area%
1	7,108	95,849
2	8,420	4,151
Total		100,000

(Chiralpak IC-3 column, Heptane/*i*-PrOH 97:3, 0.5 mL/min, 25 °C, 220 nm)

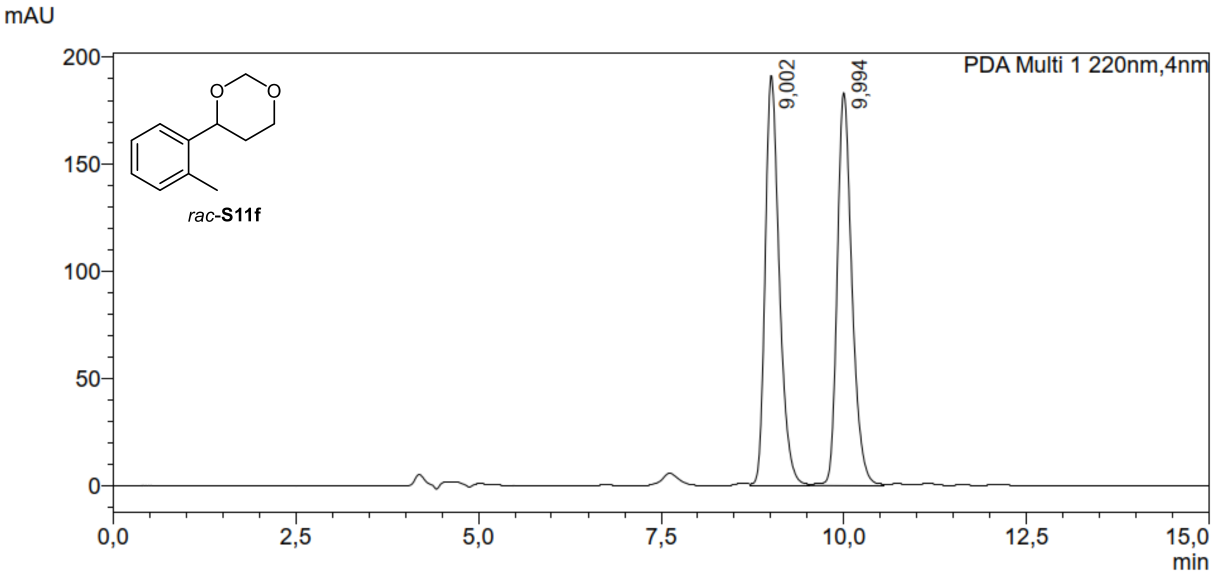

Peak#	Ret. Time	Area%
1	9,002	49,919
2	9,994	50,081
Total		100,000

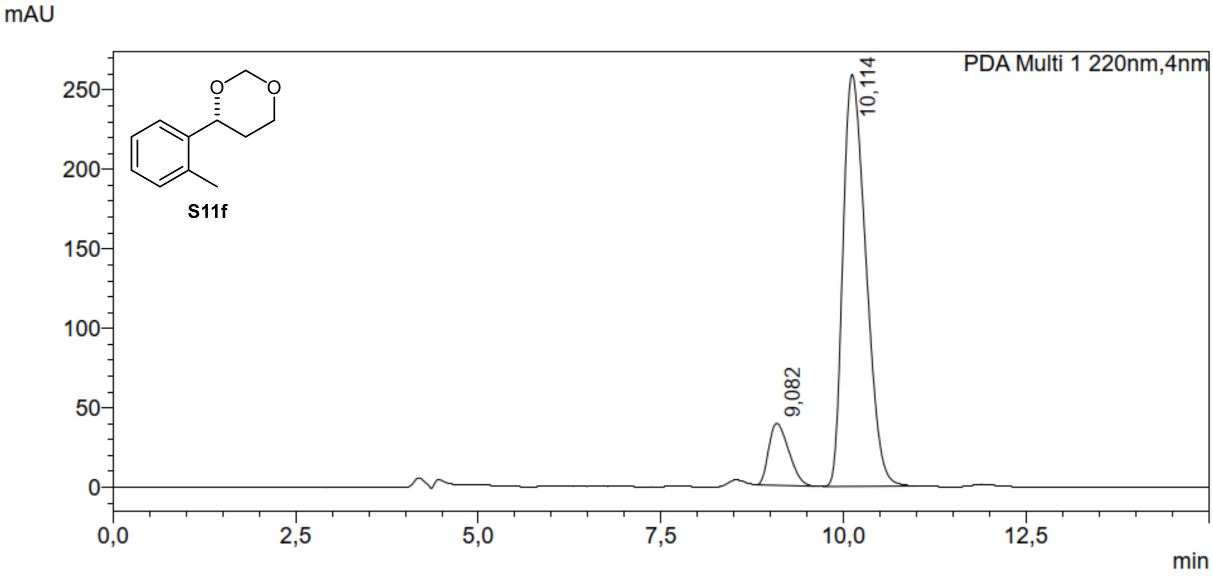

Peak#	Ret. Time	Area%
1	9,082	11,643
2	10,114	88,357
Total		100,000

(Chiralpak IC-3 column, Heptane/*i*-PrOH 95:5, 1 mL/min, 25 °C, 220 nm)

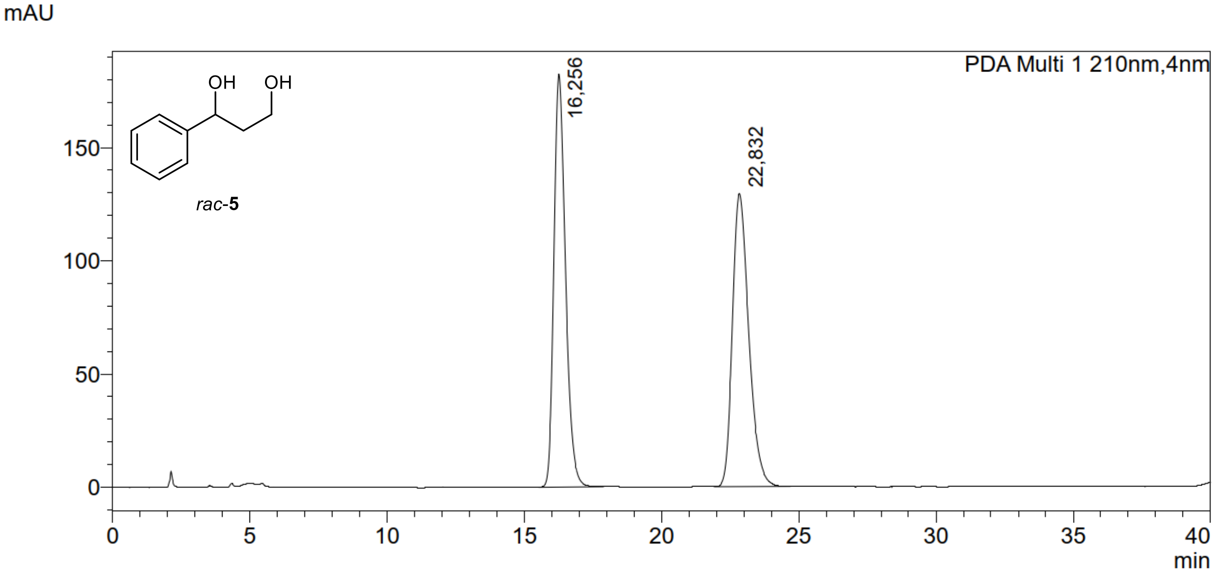

Peak#	Ret. Time	Area%
1	16,256	49,974
2	22,832	50,026
Total		100,000

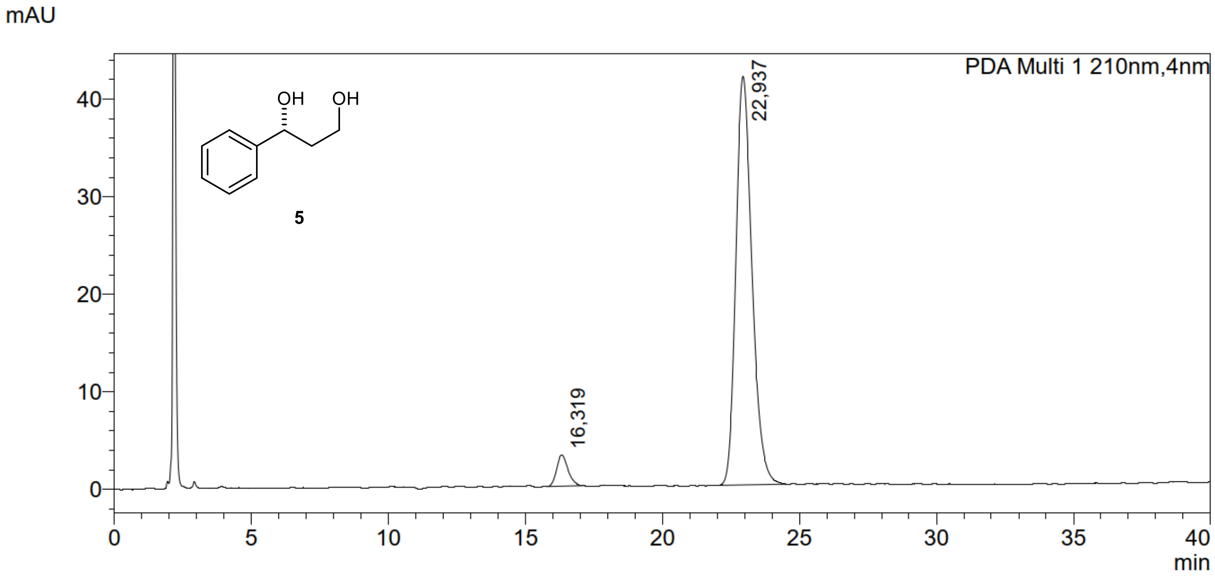

Peak#	Ret. Time	Area%
1	16,319	5,133
2	22,937	94,867
Total		100,000

11. References

1. Armarego, W. L. F., *Purification of laboratory chemicals*. 8th ed.; Butterworth-Heinemann: Woburn, MA, 2003.
2. Harris, R. K.; Becker, E. D.; Cabral de Menezes, S. M.; Granger, P.; Hoffman, R. E.; Zilm, K. W. Further conventions for NMR shielding and chemical shifts (IUPAC Recommendations 2008). *Pure Appl. Chem.* **2008,** *80* (1), 59–84.
3. Dong, X.-Y.; Zhang, Y.-F.; Ma, C.-L.; Gu, Q.-S.; Wang, F.-L.; Li, Z.-L.; Jiang, S.-P.; Liu, X.-Y. A general asymmetric copper-catalysed Sonogashira C(sp^3)–C(*sp*) coupling. *Nat. Chem.* **2019,** *11* (12), 1158–1166.
4. Jiménez-González, L.; García-Muñoz, S.; Álvarez-Corral, M.; Muñoz-Dorado, M.; Rodríguez-García, I. Diastereoselective Synthesis of 2-Aryl-3-vinyl-2,3-dihydrobenzo[*b*]furans through a Sakurai Reaction: A Mechanistic Proposal. *Chem-Eur. J.* **2007,** *13* (2), 557–568.
5. Al-Hourani, B. J.; Bravo-Vasquez, J. P.; High, L. R. H.; Fenniri, H. Synthesis and characterization of aryl thioacetyl styrene monomers: towards a new generation of SERS-active polymers. *Tetrahedron Lett.* **2007,** *48* (52), 9144–9147.
6. Luo, F.-T.; Xue, C.; Ko, S.-L.; Shao, Y.-D.; Wu, C.-J.; Kuo, Y.-M. Preparation of polystyrene-supported soluble palladacycle catalyst for Heck and Suzuki reactions. *Tetrahedron* **2005,** *61* (25), 6040–6045.
7. Tang, M.; Han, S.; Huang, S.; Huang, S.; Xie, L.-G. Carbosulfenylation of Alkenes with Organozinc Reagents and Dimethyl(methylthio)sulfonium Trifluoromethanesulfonate. *Org. Lett.* **2020,** *22* (24), 9729–9734.
8. Kayaki, Y.; Koda, T.; Ikariya, T. A Highly Effective (Triphenyl phosphite)palladium Catalyst for a Cross−Coupling Reaction of Allylic Alcohols with Organoboronic Acids. *Eur. J. Org. Chem.* **2004,** *2004* (24), 4989–4993.
9. Cívicos, J. F.; Alonso, D. A.; Nájera, C. Oxime Palladacycle-Catalyzed Suzuki–Miyaura Alkenylation of Aryl, Heteroaryl, Benzyl, and Allyl Chlorides under Microwave Irradiation Conditions. *Adv. Synth. Catal.* **2011,** *353* (10), 1683–1687.
10. Kohler, D. G.; Gockel, S. N.; Kennemur, J. L.; Waller, P. J.; Hull, K. L. Palladium-catalysed *anti*-Markovnikov selective oxidative amination. *Nat. Chem.* **2018,** *10* (3), 333–340.
11. Xiao, J.; Li, Q.; Shen, R.; Shimada, S.; Han, L. B. Phosphonium Phenolate Zwitterion vs Phosphonium Ylide: Synthesis, Characterization and Reactivity Study of a Trimethylphosphonium Phenolate Zwitterion. *Adv. Synth. Catal.* **2019,** *361* (24), 5715–5720.
12. Kapeller, D.; Barth, R.; Mereiter, K.; Hammerschmidt, F. Preparation of Chiral α-Oxy-[$^2H_1$]methyllithiums of 99% ee and Determination of Their Configurational Stability. *J. Am. Chem. Soc.* **2007,** *129* (4), 914–923.
13. Liu, L.; Kaib, P. S. J.; Tap, A.; List, B. A General Catalytic Asymmetric Prins Cyclization. *J. Am. Chem. Soc.* **2016,** *138* (34), 10822–10825.
14. Maegawa, T.; Koutani, Y.; Otake, K.; Fujioka, H. Methylene Acetal Formation from 1,2- and 1,3-Diols Using an O,S-Acetal, 1,3-Dibromo-5,5-dimethylhydantoin, and BHT. *J. Org. Chem.* **2013,** *78* (7), 3384–3390.
15. Zhang, J.; Hua, L.; Li, F.; Wu, X.; Tian, S.; Yang, J. Prins Cyclization of Styrenes or Acetophenone Catalyzed by DBSA in Water. *Synthetic Commun.* **2011,** *42* (8), 1234–1242.
16. Kalkhambkar, R. G.; Jeong, Y. T. Highly Efficient Synthesis of 1,3-Dioxanes via Prins Reaction in Brønsted-Acidic Imidazolium Ionic Liquid. *Synthetic Commun.* **2014,** *44* (6), 762–771.
17. Fujioka, H.; Senami, K.; Kubo, O.; Yahata, K.; Minamitsuji, Y.; Maegawa, T. Mild Deprotection of Methylene Acetals in Combination with Trimethylsilyl Triflate–2,2′-Bipyridyl. *Chem. Pharm. Bull.* **2010,** *58* (3), 426–428.
18. Sood, D. E.; Champion, S.; Dawson, D. M.; Chabbra, S.; Bode, B. E.; Sutherland, A.; Watson, A. J. B. Deoxyfluorination with CuF_2: Enabled by Using a Lewis Base Activating Group. *Angew. Chem. Int. Ed.* **2020,** *59* (22), 8460–8463.
19. Tateiwa, J.-i.; Hashimoto, K.; Yamauchi, T.; Uemura, S. Cation-Exchanged Montmorillonite (M^{n+}-Mont)-Catalyzed Prins Reaction. *B. Chem. Soc. Jpn.* **1996,** *69* (8), 2361–2368.
20. Adam, J.-M.; Aebi, J.; Binggeli, A.; Green, L.; Hartmann, G.; Maerki, H. P.; Mattei, P.; Ricklin, F.; Roche, O. NOVEL HETEROCYCLYL COMPOUNDS. Patent US 2010/0022518 A1, January 28, 2010.
21. Trofymchuk, S.; Bugera, M. Y.; Klipkov, A. A.; Razhyk, B.; Semenov, S.; Tarasenko, K.; Starova, V. S.; Zaporozhets, O. A.; Tananaiko, O. Y.; Alekseenko, A. N.; Pustovit, Y.; Kiriakov, O.; Gerus, I. I.; Tolmachev, A. A.; Mykhailiuk, P. K. Deoxofluorination of (Hetero)aromatic Acids. *J. Org. Chem.* **2020,** *85* (5), 3110–3124.
22. Garg, G.; Forsberg, L. K.; Zhao, H.; Blagg, B. S. J. Development of Phenyl Cyclohexylcarboxamides as a Novel Class of Hsp90 C-terminal Inhibitors. *Chem-Eur. J.* **2017,** *23* (65), 16574–16585.

23. Yue, Y.; Turlington, M.; Yu, X. Q.; Pu, L. 3,3′-Anisyl-Substituted BINOL, H_4BINOL, and H_8BINOL Ligands: Asymmetric Synthesis of Diverse Propargylic Alcohols and Their Ring-Closing Metathesis to Chiral Cycloalkenes. *J. Org. Chem.* **2009,** *74* (22), 8681–8689.
24. Ahmed, I.; Clark, D. A. Rapid Synthesis of 3,3′ Bis-Arylated BINOL Derivatives Using a C–H Borylation *in Situ* Suzuki–Miyaura Coupling Sequence. *Org. Lett.* **2014,** *16* (16), 4332–4335.
25. Ishitani, H.; Ueno, M.; Kobayashi, S. Enantioselective Mannich-Type Reactions Using a Novel Chiral Zirconium Catalyst for the Synthesis of Optically Active β-Amino Acid Derivatives. *J. Am. Chem. Soc.* **2000,** *122* (34), 8180–8186.
26. Schwengers, S. A.; De, C. K.; Grossmann, O.; Grimm, J. A. A.; Sadlowski, N. R.; Gerosa, G. G.; List, B. Unified Approach to Imidodiphosphate-Type Brønsted Acids with Tunable Confinement and Acidity. *J. Am. Chem. Soc.* **2021,** *143* (36), 14835–14844.
27. Neese, F. The ORCA program system. *WIREs Comput. Mol. Sci.* **2011,** *2* (1), 73–78.
28. Zhang, Y.; Yang, W. Comment on “Generalized Gradient Approximation Made Simple”. *Phys. Rev. Lett.* **1998,** *80* (4), 890–890.
29. (a) Grimme, S.; Antony, J.; Ehrlich, S.; Krieg, H. A consistent and accurate *ab initio* parametrization of density functional dispersion correction (DFT-D) for the 94 elements H-Pu. *J. Chem. Phys.* **2010,** *132* (15), 154104; (b) Grimme, S.; Ehrlich, S.; Goerigk, L. Effect of the damping function in dispersion corrected density functional theory. *J. Comput. Chem.* **2011,** *32* (7), 1456–1465.
30. Weigend, F.; Ahlrichs, R. Balanced basis sets of split valence, triple zeta valence and quadruple zeta valence quality for H to Rn: Design and assessment of accuracy. *Phys. Chem. Chem. Phys.* **2005,** *7* (18), 3297–3305.
31. Barone, V.; Cossi, M. Quantum Calculation of Molecular Energies and Energy Gradients in Solution by a Conductor Solvent Model. *J. Phys. Chem. A.* **1998,** *102* (11), 1995–2001.
32. Zhao, Y.; Truhlar, D. G. The M06 suite of density functionals for main group thermochemistry, thermochemical kinetics, noncovalent interactions, excited states, and transition elements: two new functionals and systematic testing of four M06-class functionals and 12 other functionals. *Theor. Chem. Acc.* **2007,** *120* (1-3), 215–241.
33. Bickelhaupt, F. M.; Houk, K. N. Analyzing Reaction Rates with the Distortion/Interaction-Activation Strain Model. *Angew. Chem. Int. Ed.* **2017,** *56* (34), 10070–10086.
34. Legault, C. Y.: CYLview, 1.0b; Université de Sherbrooke, 2009 (http://www.cylview.org).
35. Humphrey, W.; Dalke, A.; Schulten, K. VMD: Visual molecular dynamics. *J. Mol. Graphics* **1996,** *14* (1), 33–38.
36. Kupova, O. Y.; Vakulin, I. V.; Talipov, R. F.; Morozkin, N. D.; Talipova, G. R. Theoretical investigation of the role of formaldehyde dimers in the Prins reaction. *React. Kinet. Mech. Cat.* **2013,** *110* (1), 41–52.
